# A graph clustering algorithm for detection and genotyping of structural variants from long reads

**DOI:** 10.1093/gigascience/giad112

**Published:** 2024-01-11

**Authors:** Nicolás Gaitán, Jorge Duitama

**Affiliations:** Systems and Computing Engineering Department, Universidad de Los Andes, Bogotá 111711, Colombia; Systems and Computing Engineering Department, Universidad de Los Andes, Bogotá 111711, Colombia

**Keywords:** structural variants, bioinformatics, genotyping, graph algorithms, genomics

## Abstract

**Background:**

Structural variants (SVs) are genomic polymorphisms defined by their length (>50 bp). The usual types of SVs are deletions, insertions, translocations, inversions, and copy number variants. SV detection and genotyping is fundamental given the role of SVs in phenomena such as phenotypic variation and evolutionary events. Thus, methods to identify SVs using long-read sequencing data have been recently developed.

**Findings:**

We present an accurate and efficient algorithm to predict germline SVs from long-read sequencing data. The algorithm starts collecting evidence (signatures) of SVs from read alignments. Then, signatures are clustered based on a Euclidean graph with coordinates calculated from lengths and genomic positions. Clustering is performed by the DBSCAN algorithm, which provides the advantage of delimiting clusters with high resolution. Clusters are transformed into SVs and a Bayesian model allows to precisely genotype SVs based on their supporting evidence. This algorithm is integrated into the single sample variants detector of the Next Generation Sequencing Experience Platform, which facilitates the integration with other functionalities for genomics analysis. We performed multiple benchmark experiments, including simulation and real data, representing different genome profiles, sequencing technologies (PacBio HiFi, ONT), and read depths.

**Conclusion:**

The results show that our approach outperformed state-of-the-art tools on germline SV calling and genotyping, especially at low depths, and in error-prone repetitive regions. We believe this work significantly contributes to the development of bioinformatic strategies to maximize the use of long-read sequencing technologies.

## Introduction

Structural variants (SV) are a type of genetic polymorphism, in both coding and noncoding sequences, which are usually defined by their length (>50 bp). The main types of SVs are deletions, insertions, translocations, inversions, and copy number variants [1]. The main genomic processes that cause the formation of structural variants are DNA recombination, replication, and repair-associated processes [2]. For example, one common mechanism is nonallelic homologous recombination (NAHR), which is a genetic repair mechanism in which misalignment of previously duplicated regions called low copy repeats (LCRs) occurs during meiosis. This subsequently causes a genomic rearrangement event on another locus that does not belong to the LCR gene, thus creating further deletions or duplications [3].

The interest in SVs comes mainly from the functional consequences of their genetic diversity. It has been proven that many SVs are involved in different gene expression patterns and influence different characteristics. SVs that are located adjacent to genes may structurally affect *cis-*regulatory regions by position or composition, leading to either silencing or increasing gene expression, which explains variation of quantitative trait loci (QTL) [4]. For example, Alonge and collaborators [5] found that at least 50% of the SVs found in an assessment of around 100 lines of tomato were associated with gene expression regulatory processes, mostly causing reductions or even silencing of gene products. Another case is when duplications increase the amount of overall transcript-protein production by gene dosage effect. This has proven beneficial for artificial selection in certain plant species where the average size of fruits increased because the plant variant suffered a specific duplication in a cytochrome coding gene [5].

Structural variants also provide fundamental information about evolutionary relationships between organisms and their natural history. Many whole-genome sequencing (WGS) studies have been conducted to assess the prevalence of different SVs and their variation in organisms, populations, or species. In plants, analyzing structural variants allowed elucidation of the dynamics of whole-genome duplication (WGD) events and their evolutionary role [6]. WGDs are followed by a fast diploidization process, mainly because most of the duplicated genes become paralogs [6]. Furthermore, many components of the C4 metabolic pathway were brought by these WGD events and single duplication events. This is an interesting case of convergence throughout the evolution of different plant lineages [7]. These changes are influenced by the synergistic effect of WGDs, transposed duplication, and dispersed gene duplication, evidenced by overlapping peaks in the rates of synonymous substitutions [6]. This shows how SVs can provide substantial amounts of evidence for evolutionary studies.

Given the importance of SVs, a large number of computational methods have been developed to identify and genotype SVs, based on high-throughput sequencing (HTS) data. Most of these SV detection tools are based on short-read sequencing technologies [8, 9]. This presents many limitations, mostly due to the length of structural variants, which usually exceeds the read length, which reduces the precision of both identification and genotyping [10, 11]. Recently, new SV calling tools have adopted long reads as their input data, significantly increasing the accuracy of SV detection in comparison with short read–based callers [11, 12]. This has allowed many researchers to increase their catalog of functionally relevant structural variants, including some that affect the pathophysiology of diseases such as human cancer [13, 14]. However, further improvements could be achieved by novel algorithmic techniques. Some difficulties arise even when long reads are used. Since SV detection relies on accurate read alignment, dissimilar, partial, or inaccurate read alignments obscure the signal to perform a consistent detection and genotyping of SVs. Thus, the results also depend on the accuracy of the aligner software [15]. Additionally, from a software design point of view, our experience indicates that most current tools are difficult to operate because they require a large number of specific libraries and versions, their implementations are not debugged correctly, and exceptions are not handled appropriately. For short read–based callers, these limitations have been described by a recent benchmark study [9].

Benchmarking SV detection is a difficult task. First, there are few independently validated gold-standard datasets for real sequencing data because experimental validation is difficult to perform at a large scale. Consequently, there is no consensus on which of the existing tools produces the closest result to a gold-standard set. Bolognini and collaborators [16] addressed this issue by implementing a simulation software called VISOR, which produces a complete haplotype-resolved sample genome and simulates read alignments from a list of SVs, with either Oxford Nanopore or PacBio error profiles. Trying to optimize the SV calling pipeline, Jiang and collaborators [17] evaluated the accuracy of different SV callers using VISOR simulations on real reported human SVs. For the 20× simulated dataset, they report that the best tools are CuteSV (F1 = 0.8), SVIM (F1 = 0.798), and Sniffles2 (0.769). Additionally, they provide recommendations for SV calling best practices such as sequencing experiments with read lengths of about 20 kb at 20× depth. Regarding real datasets, the most widely recognized and best-curated case is the high-confidence structural variant dataset (Sample HG002 on reference genome GRCh37) from the Genome In A Bottle (GIAB) human sample project crafted for benchmarking [18]. The events reported in this file come from a mixture of sequencing technologies and have been predicted by using a pipeline integrating many different tools.

The HGSVC consortium also generated high-confidence SV calls suitable for benchmarking. In the first version, a haplotype-resolved curated SV callset against the GRCh38 genome was produced for each of 3 samples from different ethnicities, including Han Chinese, Puerto Rican, and Yoruba from Nigeria (HG00514, HG00733, and NA19240), respectively. This provides SV variation profiles for individuals with a wide range of genetic diversities, including admixed individuals [19]. Similar to the GIAB effort, multiple sequencing platforms and variant calling methods were used to produce these datasets, especially the reference-guided assembly of the samples and their parents, which made it possible to determine the haplotype of the SVs. Furthermore, the HGSVC2 version improved these SV calls using *de novo* assembly with the PAV algorithm [19, 20].

Structural variant detection provides the possibility of finding biological insights with many different functional consequences. In this article, we developed a new software solution that improves the detection of germline SVs from long-read alignments using the DBSCAN algorithm to solve the clustering problem and implements a new Bayesian genotyping model. This functionality is integrated into the bioinformatic software suite (Next Generation Sequencing Experience Platform [NGSEP]) to further facilitate the analysis of genomic data.

## Results

### A new clustering algorithm for detection and genotyping of SVs

The process of structural variant detection and genotyping starts from reads aligned to a reference genome and is divided into 3 main stages described as follows.

#### Signature collection

The main input to this algorithm is a set of read alignments in SAM or BAM format, obtained from mapping long reads to a reference genome. Signatures are individual signals of a structural variant that are contained within each read alignment or constructed from discordant partial alignments. They can be divided into intra-alignment and interalignment signatures. Intra-alignment signatures consist of evidence of deletions or insertions that are predicted as part of the read alignment process. Thus, these signatures are collected by reading the description of the alignment (encoded in the CIGAR field of the SAM format) to find signals of insertion or deletion. Conversely, reads with multiple discordant alignment segments, regarding their position or orientation, are selected to identify interalignment signatures.

**Figure 1:**
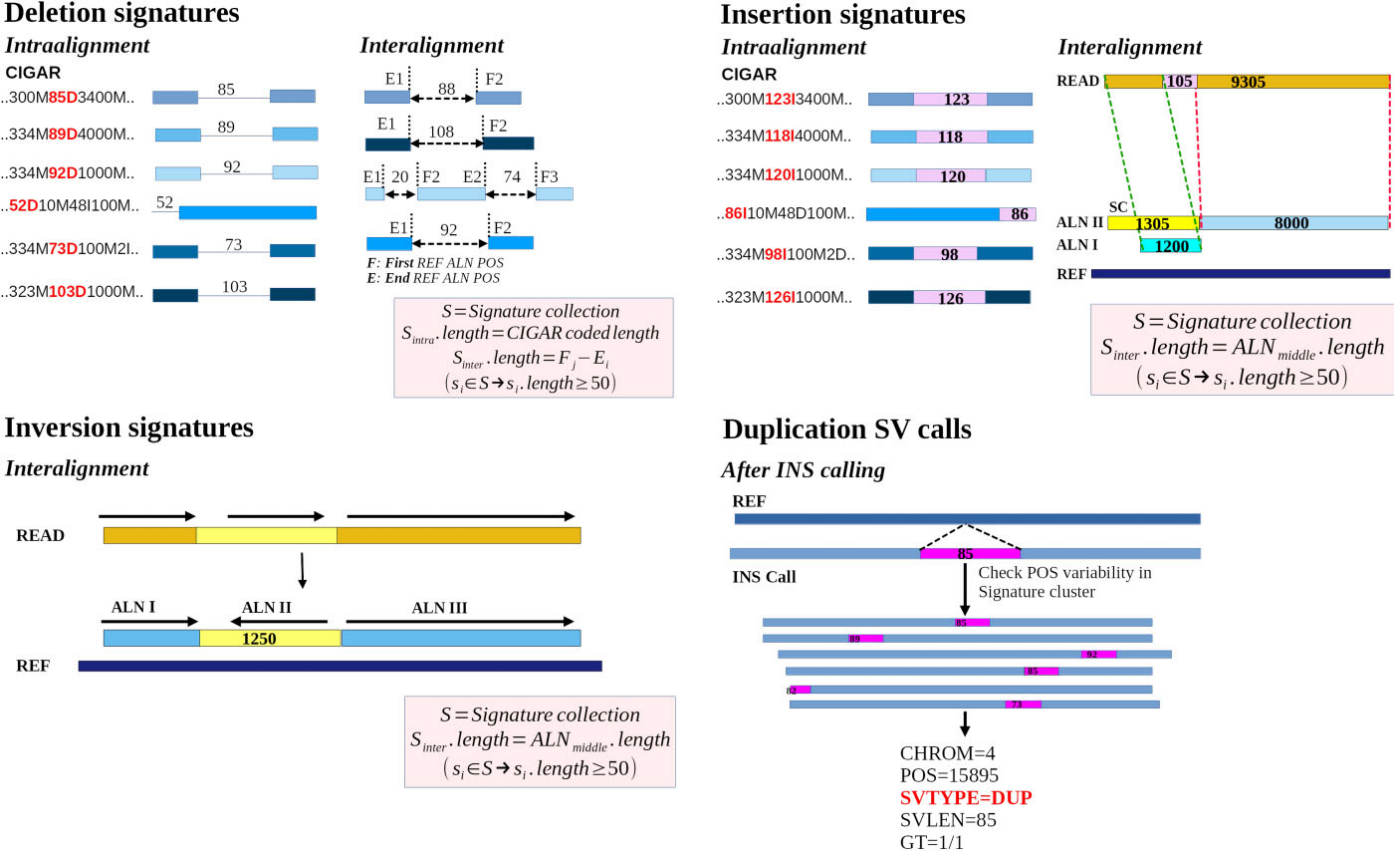
Procedures for intra-alignment and interalignment recollection of evidence (signatures) for indels, inversions, and duplications from reads aligned to a reference genome. Duplication assignment is performed only after insertion SVs have been called from signatures. ALN, read alignment; LSC, longest soft clip; POS, first position in the reference; SC, soft clip.

Figure 1 shows the procedures that we implemented for the recollection of signatures for each SV type. Intra-alignment deletions and insertions are identified by parsing the CIGAR strings and searching for their codes (e.g., D or I, respectively), The CIGAR code includes the length of each event within the alignment. Interalignment deletions are suspected when unmapped regions in the reference genome are flanked by partial alignments. For each read with 2 partial alignments within the same chromosome region, the reference distance between the end of the first partial alignment and the beginning of the second alignment in reference genomic coordinates is considered the length of the deletion signature. Interalignment insertion signatures are identified from reads with 2 adjacent alignments, having a soft clip starting from the presumed insertion point. For each read, the longest soft clip (LSC) is calculated by taking the maximum of soft clips at the end of each alignment. The length of the partial alignment that does not contain the LSC is subtracted from the length of the LSC to estimate the length of the insertion signature. Inversions appear as 3 consecutive partial alignments where the middle alignment has an opposite orientation, compared to the 2 flanking alignments. The length of the inversion is predicted as the length of the middle alignment. Signatures are filtered from the minimum SV length specified by the user (default ≥50 bp) and are added to a collection, which is sorted by chromosome and reference coordinates.

#### Signature clustering

Given a set of SV signatures, we implemented a graph-based clustering in which each cluster becomes a candidate SV event. A graph is built independently for each signature type. The vertices of the graph correspond to the input collection of signatures identified in the previous step. Each signature is represented by a tridimensional vector with 3 numeric values: start coordinate in the reference genome (*B_i_*), end coordinate in the reference genome (*E_i_*), and signature length (*L_i_*). The cost *m_ij_* of the edge between 2 signatures *i* and *j* corresponds to the Euclidean distance of their corresponding vectors:


\begin{eqnarray*} \begin{array}{@{}*{3}{c}@{}} {FP{D}_{ij} = \left| {{B}_j - {B}_i} \right|}& \quad {LP{D}_{ij} = \left| {{E}_j - {E}_i} \right|}& \quad {L{D}_{ij} = \left| {{L}_j - {L}_i} \right|}\\ \,& \quad {{m}_{ij} = \sqrt {FPD_{ij}^2 + LPD_{ij}^2 + LD_{ij}^2} }& \quad \, \end{array}
\end{eqnarray*}

**Figure 2:**
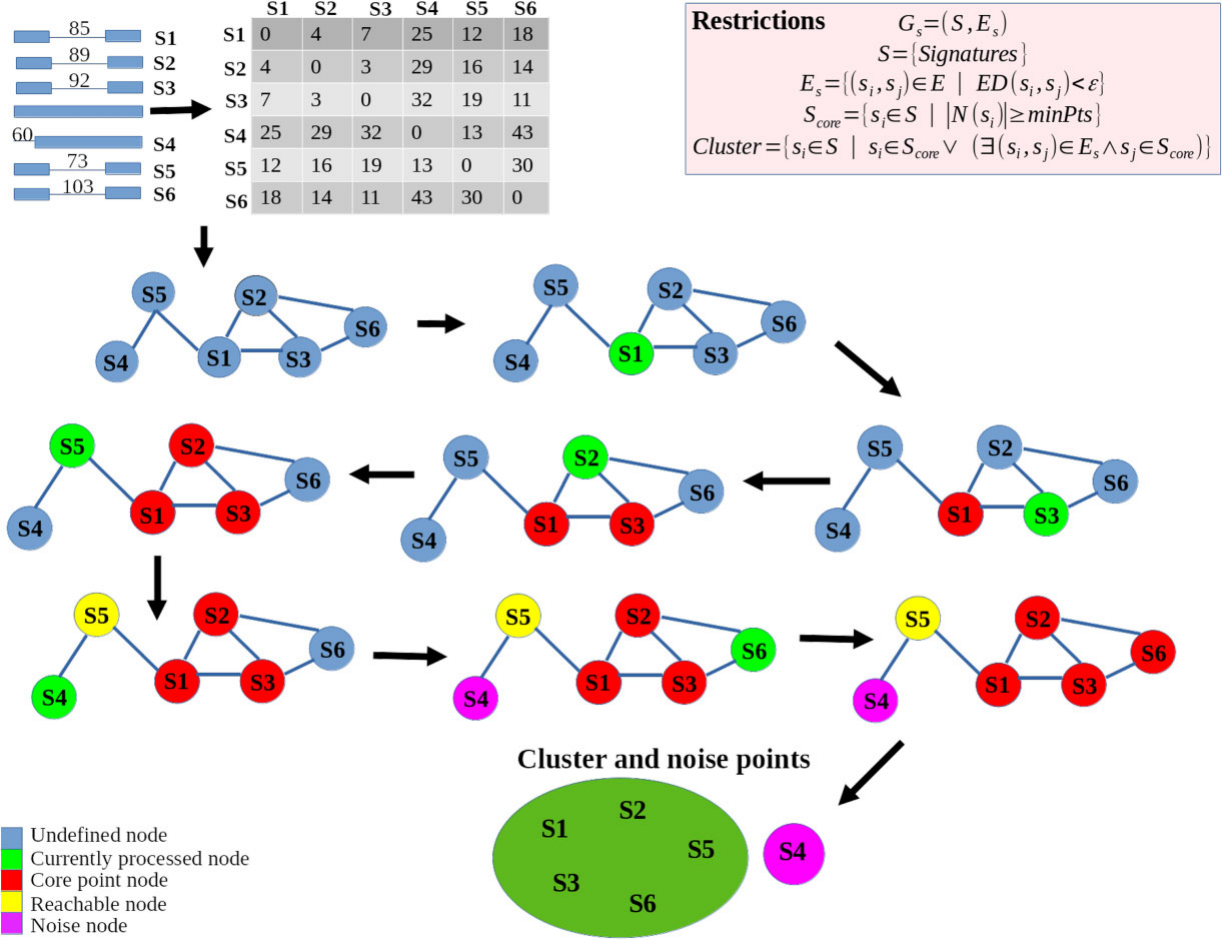
DBSCAN algorithm outlined in the context of variant calling applied to an example deletion event. A distance matrix is built from signatures using the Euclidean distance of 3 numerical values: the difference between the first and last reference position and the length of each signature. BFS is used to build clusters based on core points (points with degrees larger than a given threshold) or points reachable from these core points.

The DBSCAN algorithm is a nonsupervised clustering procedure for *n*-dimensional vectors (points) based on the principle of density-based grouping [21]. The parameters of this algorithm are a threshold *epsilon* (ε), which limits the distance for considering 2 points as neighbors, and a minimum number of neighbors (minPts) that a point should have to be considered a *core point*. The lemma states that considering a cluster that contains certain *core points*, then any point that is density reachable from any of those *core points* (in the graph context, any point that has a path from any *core point*) will be considered part of the cluster. Any point that is not reachable from any *core point* will be considered a noise signal. The procedure to implement this algorithm was as follows. Starting from an initially complete graph with *n* points, the algorithm eliminates the edges where *m_ij_* is bigger than or equal to ε. Then, each point is visited to test if its number of neighbors is at least *minPts*, in which case it is labeled as a core point. Consequently, a new cluster is initialized with the core point and its direct neighbors, and a breadth first search (BFS) is performed by pushing this neighborhood into a queue where each point will also be queried for its neighbors to assess the *core point* property presumption, repeating this process until all of the density reachable points from any core point in the cluster are visited. If there are unvisited points, the procedure continues until all points are visited. Figure 2 shows the main steps and restrictions of this procedure.

#### Cluster to genotyped SV

**Figure 3:**
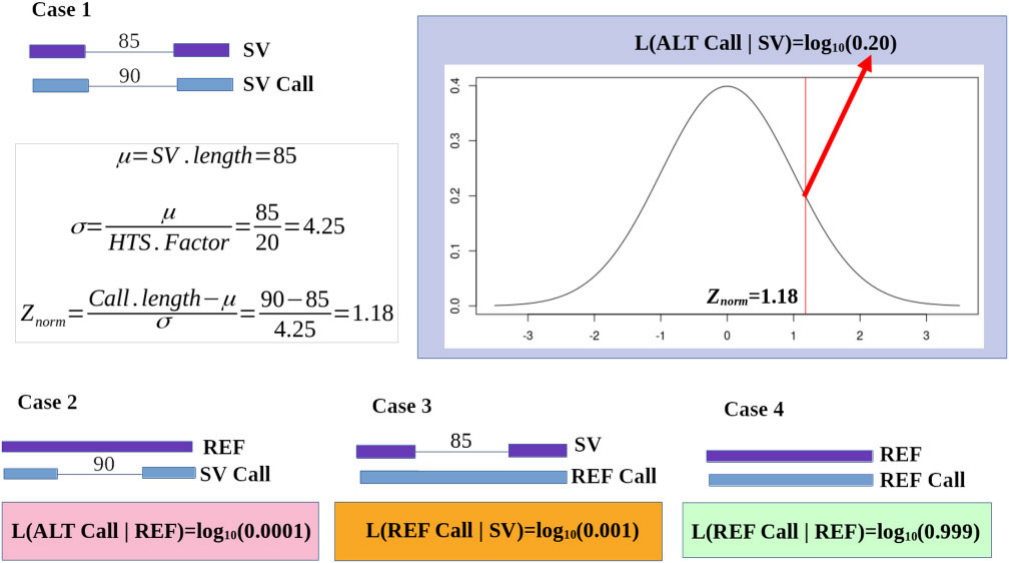
Likelihood estimation for each of 4 possible scenarios for a diploid organism. In each case, the base 10 logarithm of the obtained value is calculated. For case 1, an example of the estimation of the log-likelihood value is shown, from a situation where the SV allele with a length of 85 bp is assumed, and a read alignment contains a call supporting the SV with a length of 90 bp. The HTS factor is a normalization constant based on the sequencing technology and the according error rate.

Each signature cluster identified in the previous step becomes a candidate SV. The last step of the process is the genotyping of these candidates. To identify SV coordinates, the average of the first reference coordinates of the signatures within the cluster is estimated. The last coordinate is calculated likewise. The length is taken as the difference between both the last and first SV coordinates, except for insertions where the average length of the cluster signatures is estimated as the average of the insertion lengths of the signatures. Candidate SVs are stored in a collection sorted by reference coordinates. Then, a Bayesian genotyping process is performed for each candidate SV by reassessing the evidence that read alignments provide. To avoid having to reprocess the alignments file, a collection of compact alignment objects is kept in memory from the first stage, having the minimum possible information needed for this step. For each SV, intersecting read alignments are collected, and those containing clustered signatures are considered supporting evidence for the alternative allele hypothesis. If the spanning read alignment contains no signatures, it is counted as a supporting call for the reference allele. Figure 3 shows the estimation of the likelihood for the 4 possible scenarios, generated from the combination of the hypotheses, the 2 plausible alleles from which the read could be sequenced (SV or REF alleles), with calls from a read alignment that may or may not support these allele hypotheses. The distribution of lengths of the clustered signatures supporting the SV hypothesis is used to estimate the likelihood of a read alignment supporting this SV. In this case, it is assumed that the read was actually sequenced from a chromosome affected by the SV (case 1). If a reference allele is assumed (case 2), a read with an SV signature is proposed to have happened by a misalignment or sequencing error, and a fixed value (0.0001 by default) is used as likelihood. The likelihood of a read supporting the reference allele that is assumed to be sequenced from a haplotype affected by the SV is calculated as the probability of having an indel error that reverts the SV and is also a constant value (0.001 by default) (case 3). Finally, a fixed value (default 0.999) is used for the likelihood of a read supporting the reference allele assuming sequencing from a reference haplotype.

Read likelihoods for each allele hypothesis are transformed into posterior probabilities for each possible genotype following the same procedure implemented in NGSEP to perform single-nucleotide polymorphism (SNP) genotyping [22]. The hypothesis having the largest posterior probability is assigned as the predicted genotype. If the genotype of an SV call is assigned as a homozygous reference (0/0), this call will be considered not well supported, and it will be filtered out of the output. Similar to SNP genotyping, the quality of such SV calls will be the phred score *Q* corresponding to the genotype posterior probability. Finally, duplications are identified after the 3 main steps from genotyped insertion SVs, if the supporting intra-alignment signatures differ significantly in reference coordinates.

### Benchmarking with simulation experiments

We performed 2 simulations of structural variants in the genome of *Arabidopsis thaliana* using the tool VISOR [16]. In total, 1,718 insertions, 2,532 deletions, and 2,065 inversions were generated for benchmark experiments. Read alignment subsets were produced for 20×, 30×, 45×, and 60×. The precision-recall results of our NGSEP algorithm were compared to those of state-of-the-art tools, including SVIM (version 2.0.0) [15], Sniffles2 (version 2.2) [23, 24], CuteSV (version 2.0.3) [25], and Dysgu (version 1.6.1) [8]. After obtaining the metrics for both simulation experiments, precision-recall curves and F-score against depth were plotted for each tool. Additionally, execution times for each depth dataset were evaluated for single-thread runs.

**Figure 4:**
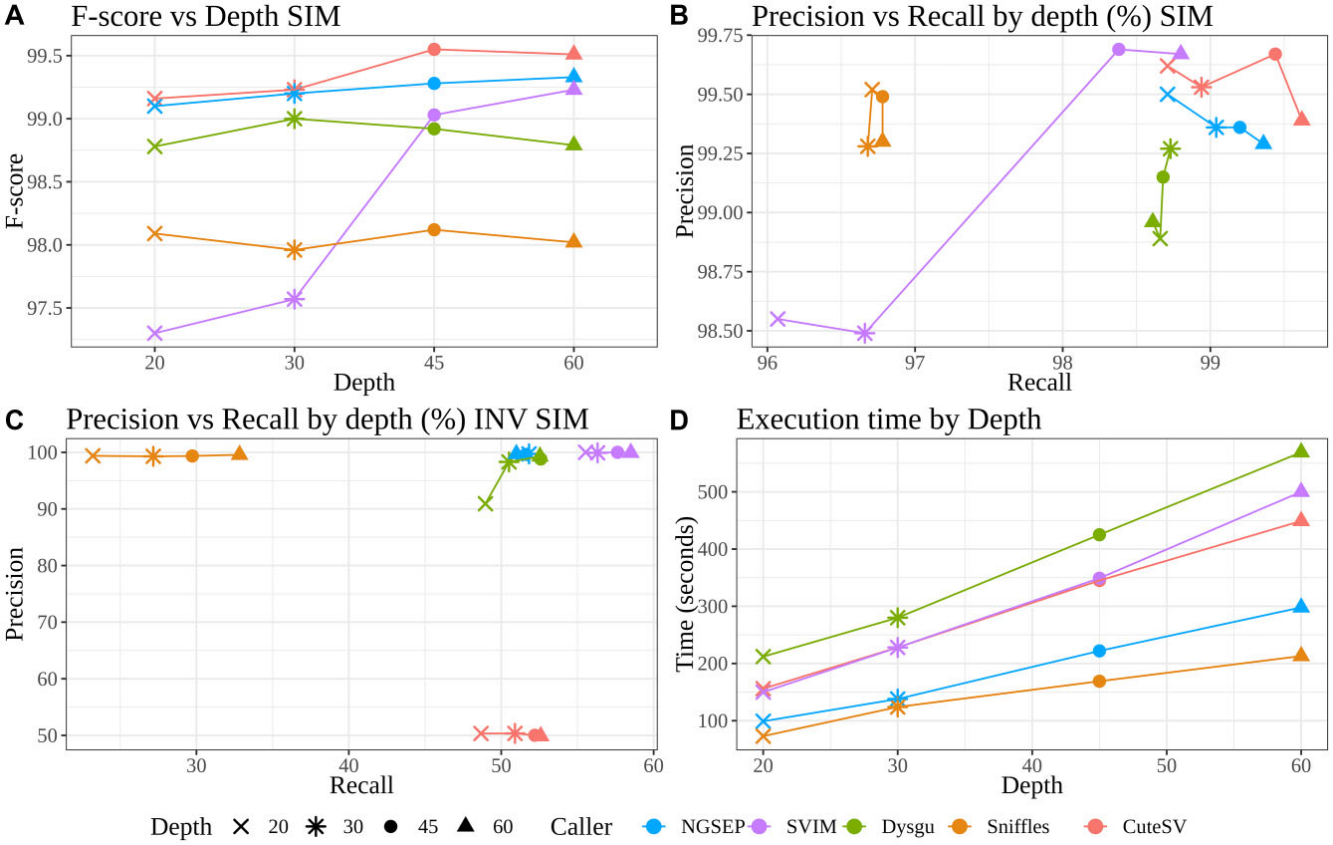
Simulation benchmarking results. The shape of points represents different depths for values of 20×, 30×, 45×, and 60×. (A) F-score as a function of sequencing depth. (B, C) Precision-recall curves of SV detection for alignments at different depths: (B) indels and (C) inversions. The indel 20× F-score values are as follows: NGSEP: 99.1, Sniffles: 98.09, SVIM: 97.3, Dysgu: 98.78, CuteSV: 99.16. For SVIM, a quality score (QS) filter >10 was applied given that this provides the best results for the tool, where 0 filter provides very low precision and >20 filters provide low recall. (D) Single-thread execution time of all callers as a function of the depth of the input alignments.

Figure 4A shows that the NGSEP algorithm presented above achieves an F-score value over 99, outperforming SVIM, Dysgu, and Sniffles2 for all depths (values available in the Supplementary File S1). Only CuteSV achieves similar scores, reaching 99.5 in the 45× dataset. Figure 4B shows that NGSEP and CuteSV keep high performance for varying alignment depths in both precision and recall. In the inversion simulation benchmark, SVIM produced the highest F-score, closely followed by NGSEP and Dysgu (Fig. 4C). The 3 tools showed almost perfect precision and between 50% and 60% recall. Conversely, CuteSV showed a precision of only 50%, and Sniffles2 failed to detect most inversions.

Additionally, we generated a simulated SV gold standard from the Human T2T genome [26] following the same pipeline, as well as increasing the amount of depth samples to 5×, 10×, 20×, 30×, 40×, and 60× including 5,000 insertions and 5,000 deletions, to evaluate the performance of the algorithms with bigger input data sizes and different genome features.

Results from these simulations are similar to those obtained with the *Arabidopsis* genome (Supplementary File S1). Our algorithm also generated good precision and recall values in this simulation. Comparing the results of the different tools, the outcome was similar to that obtained with *Arabidopsi*s, with the exception of the F-scores of Sniffles2, which were the best in this simulation. NGSEP had slightly larger F-scores than CuteSV in the 10× and the 60× datasets (Supplementary Fig. S1).

Single-thread runtimes were recorded for all experiments to compare the tools in terms of computational efficiency. As shown in Fig. 4D, all of them follow a linearly increasing trend. Sniffles2 and NGSEP consistently required lower execution times compared to the other tools. It is worth clarifying that Sniffles2, CuteSV, and Dysgu support multithreading, which significantly reduces runtimes at the cost of processing resources. Dysgu was the worst-performing tool in terms of computational efficiency, requiring about 3 times more execution time than the NGSEP algorithm. For the T2T dataset, Dysgu ran faster than NGSEP, and CuteSV had the highest execution runtime (Supplementary Fig. S1).

### Benchmarking with the GIAB human genome

To assess the performance of our method on real datasets, we performed multiple experiments using reads from the GIAB human individual HG002, for which a gold-standard set of large indel calls is publicly available [27]. Both 56× PacBio HiFi circular consensus sequencing (CCS) and 47× ONT ultra-long (UL) reads sequenced from the HG002 subject were randomly sampled at average depths of 10×, 20×, 30×, and 40× to perform different experiments. Truvari [28] was used to obtain precision and recall metrics of test calls against the gold standard, which was restricted either to the Tier 1 plus Tier 2 (T1+2) regions, or just Tier 1 (T1), and PASS-only SVs. Additionally, a F-score variation called a GTF score was estimated to assess their performance regarding the combination of genotyping accuracy and recall. Further details for this metric are provided in the Methods section.

**Figure 5:**
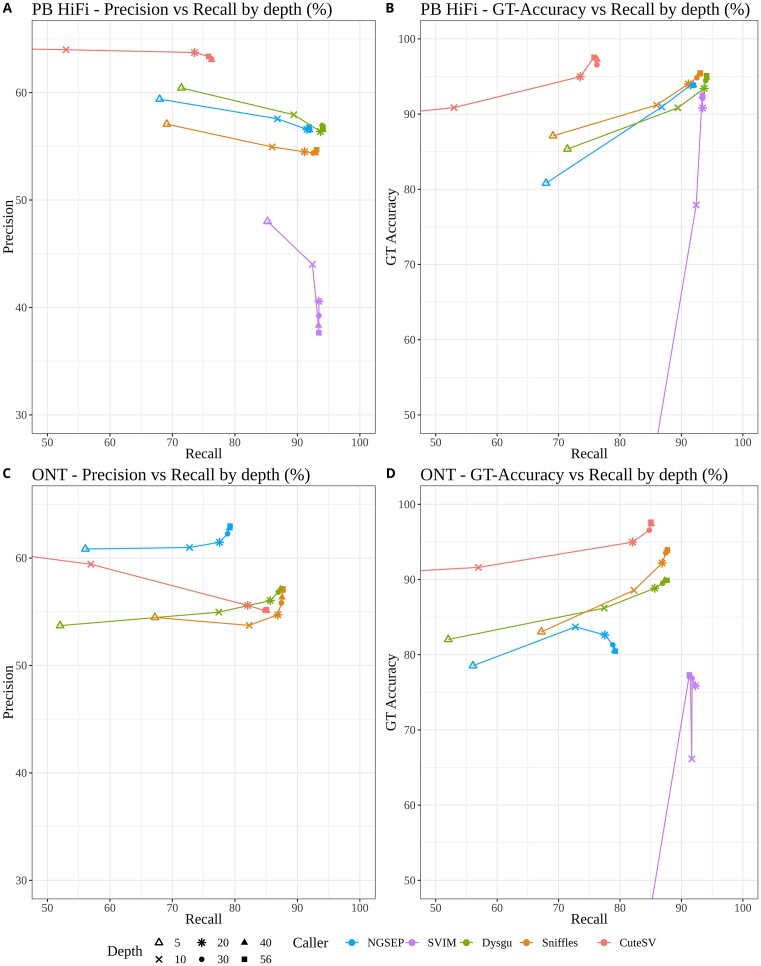
Performance metrics for PacBio HiFi and ONT data of HG002, using the T1+2 SV calls of GIAB as gold standard. (A, C) Precision-recall curves of SV discovery over varying depths for all callers on (A) HiFi data and (C). ONT data. (B, D) Curves comparing genotyping accuracy with recall on (B) HiFi data and (D) ONT data. F-score values for the 20× (HiFi, ONT) depth mappings are: NGSEP: 69.93, 68.58; Sniffles: 68.2, 67.15; SVIM: 56.58, 31.58; Dysgu: 70.4, 67.75; CuteSV: 68.28, 66.28. SVIM and CuteSV had low values in some metrics; thus, some depth points for these tools are not included, but a trajectory line is left to indicate the results trend they followed over the different datasets.

Figure 5A shows the results of the benchmark experiments detecting variants from PacBio HiFi alignments, using as gold standard the T1+2 dataset and varying read depth from 5× to 56×. Dysgu provided the best F-score for low-depth mappings, closely followed by NGSEP (5×, 10×, and 20×; see exact values in the Supplementary File S1). CuteSV had the best precision but the worst recall. Similar to the simulation results, at increasing depths, SVIM increased recall at a high cost on precision. Regarding GTF score, Dysgu produced the highest values. CuteSV produced the highest GT accuracy, but the low recall reduced its GTF score (Fig. 5B). The precision for all tools was low (up to 65%) mainly because Tier 2 included highly repetitive regions in the human genome. If the gold standard is restricted to the T1 dataset, all tools improve precision, reaching values over 90% in almost all cases (Supplementary Fig. S2). Sniffles2 shows the most important increase in this comparison, reaching precision values slightly larger than those of NGSEP for depths above 30×.

Regarding ONT aligned reads and comparing against the T1+2 dataset, NGSEP is the most accurate tool comparing precision and recall. CuteSV, Sniffles2, and Dysgu achieve better recall and genotyping accuracy than NGSEP, at the cost of precision. Restricting the comparison to T1 regions, precision increases for all tools, and Sniffles2 becomes the tool with the highest F-score overall (Supplementary Fig. S2). The behavior of all callers is relatively consistent with the HiFi data, but the values obtained for the different metrics are consistently lower, probably due to the higher error rate of ONT reads.

A breakdown of these results in both deletion and insertion categories shows that the major improvements in performance metrics for NGSEP over the other tools come from the accurate detection of insertions, especially for the ONT data. However, the low GT accuracy of NGSEP for ONT reads is caused by a GT accuracy of insertions below 80% (Supplementary Figs. S3 and S4). Consistent with the global results, the improved precision of NGSEP is not evident if only T1 regions are included in the benchmark experiments (Supplementary Figs. S5 and S6).

We also tried to include in the benchmark experiments the tool PBSV (version 2.9.0) [29]. However, this tool did not work with the original alignments, and hence we had to realign the HiFi reads with the pbmm2 mapper (available with PBSV). PBSV produced low recall values at low depths, improving as depth increased at a cost on precision. The overall accuracy of PBSV was inferior to that of NGSEP, both for the complete gold-standard dataset and for the subset of Tier 1 SVs (Supplementary Fig. S7). Both NGSEP and CuteSV benefited from improved accuracy using these realigned reads. NGSEP became the tool with the best F-score for low read depths (below 20×), whereas CuteSV reported the best metrics above 20×.

Finally, the runtime of each tool behaved similar to the simulations. Sniffles2 was the fastest tool for all subsets, and PBSV was the slowest tool in most cases (Supplementary Fig. S8A). We also analyzed the peak memory consumption for our algorithm. NGSEP took less than 8 Gb of RAM heap space to analyze the datasets up to 30×. For bigger inputs, although more space was used by the Java Virtual Machine, new objects maintained low memory consumption (Supplementary Fig. S8B). All experiments could be performed with up to 16 Gb of RAM.

### Benchmarking with the HGSVC2 samples

Taking advantage of the efforts made by the HGSVC consortium to produce accurate SV callsets [30], we included their 3 most refined samples (HG00514, HG00733, NA19240) into our benchmark experiments. These resulted in a truth set consisting of 74,467 indel SVs. The breakdown per sample and SV type is available in the Supplementary Table S1. To evaluate the quality of SV callers for low-depth and varying genetic diversity inputs, we aligned PacBio HiFi sequencing reads from each of these samples to the GRCh38 genome, using minimap2 [31]. Then, we randomly subsampled the mappings to evaluate the tools at 20× depth.

**Figure 6:**
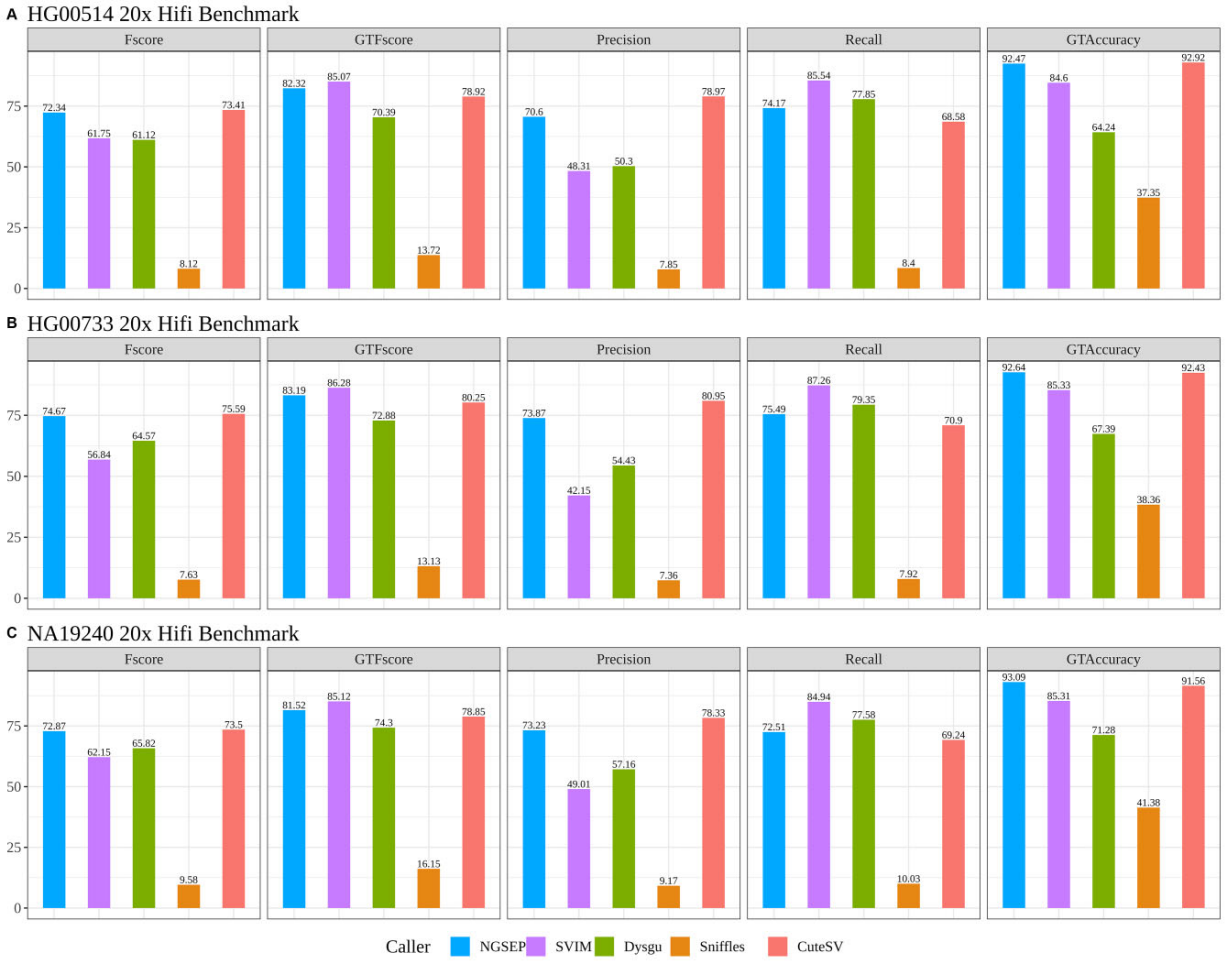
HGSVC2 benchmark experiments on 20× depth HiFi mappings for each sample: (A) HG00514: Han Chinese, (B) HG00733: Puerto Rican, and (C) NA19240: Yoruba from Nigeria. All performance metrics are shown based on the results of the tested variant callers, and their exact percentage values are portrayed over each column.

Figure 6 shows the performance metrics for SV discovery on the 3 samples. NGSEP achieved the second best F-score after CuteSV and the second best GTF score after SVIM (all values are available in Supplementary File S1). Dysgu and SVIM reported very low precision values, although they identified more than 75% of the indels. Conversely, CuteSV had high precision and genotyping accuracy, but it had between 2% and 5% less recall than NGSEP. In this experiment, the calls generated by Sniffles2 had surprisingly low performance metrics, taking into account the performance observed in the simulations and the GIAB data. After manual inspection of the results, we discovered that Sniffles2 was reporting SVs in locations consistent with the gold standards, but the reported SV length was about 2 times the SV length of the gold standard. Relaxing the reciprocal overlap for test-reference allele lengths (see Methods for details), the precision and recall metrics of SVs reported by Sniffles2 improved to values similar to those observed in the previous experiments. However, the GT accuracy was still affected, increasing only up to 45% (Supplementary Fig. S9). Consistent with the experiments with the GIAB dataset, restricting the gold standards to nonrepetitive regions increased the performance of all callers (Supplementary Figs. S10 and S11). In particular, NGSEP achieved the best genotyping accuracy for the admixed Puerto Rican individual (HG00733), suggesting that our genotyping procedure is very accurate even for samples with high heterozygosity (Fig. 6C, Supplementary Figs. S9C, S10C, and S11C).

## Discussion

The availability of long-read sequencing technologies represented a big step forward toward the accurate identification and genotyping of structural variants [13, 14]. Achieving this goal is becoming a requirement for current genomics, given the documented role of SVs as drivers of phenotyping variability and evolution [5–7, 32]. In this work, we present the results of our efforts to develop novel algorithmic techniques, aiming to increase the accuracy of both discovery and genotyping of germline SVs. Transforming the problem of clustering SV signatures into a geometric clustering problem in an Euclidean space allowed us to build a solution based on the well-known DBSCAN clustering algorithm to identify SVs [21]. A similar Euclidean space representation is implemented in the Jasmine algorithm to merge SVs from different samples into a refined call, which improved population-level analyses [33]. Even though both works differ in the clustering algorithm, they demonstrate the advantages of representing SV signals as *n*-dimensional Euclidean points and provide the groundwork for future SV analysis algorithms. Previous experiences implementing Bayesian models for SNV genotyping allowed us to increase the accuracy of SV identification and provided a framework for SV genotyping.

Benchmarking experiments running simulations and analyzing real data with the GIAB and HGSVC2 datasets indicate that our algorithm achieves competitive accuracy compared to current software solutions. NGSEP consistently provided top-tier performance metrics across the experiments, showing a great balance between recall and precision for a variety of samples. The observed differences in the results obtained with Tier 1 and Tier 1+2 regions indicate that our solution provides accurate calls in repetitive regions, which remains one of the main challenges for SV calling efforts. Compared to SVIM, our algorithm provided consistently better accuracy in all experiments. SVIM in particular ranked last in performance for ONT data. This result is consistent with previous experiments [8] and could be explained by the tendency of the hierarchical clustering implemented in SVIM to separate signatures coming from the same variant if there is high variability in alignments due to sequencing error rates. Regarding Sniffles2, this tool was very competitive, achieving in some cases superior discovery and genotyping accuracy, compared to NGSEP, both in the simulations and in the experiments with the GIAB benchmark dataset. However, this behavior was not consistent in our experiments with the HGSVC2 datasets, mainly because in these cases, Sniffles2 produced calls with about 2 times the length of the real calls. We could not identify a rationale for this behavior. Regarding CuteSV, we obtained SV calls with good accuracy running this tool, but only if the minimum read depth was set to 5× and if the testing read depth was superior to 20×. CuteSV implements a 2-step clustering procedure, making initial clusters based on coordinates and then identifying subclusters based on differences in event length. This process is controlled by a set of parameters that need to be tuned for different event types and sequencing technologies. Although we acknowledge that further testing of each tool with different parameters on each specific dataset could yield improved outcomes, we argue that this indicates that our method adapts more naturally to changes in read depths and sequencing technologies, reducing the effort to perform parameter tuning for each experiment. Finally, Dysgu had the best F-score for HiFi reads of the GIAB dataset, but this outcome was not consistent testing other benchmark datasets. Since the initial submission of this article, we have observed an important increase in accuracy for new versions of this tool, suggesting that the underlying algorithm is under active development. Continuous improvements are likely to be developed for all tools, including NGSEP and even including benchmark tools such as Truvari. This means that the current benchmark is only a snapshot of the current status of this field.

Given that even using long reads, it is not easy to identify and cluster signatures for translocations, compared to other SV types, our current solution does not support discovery of translocations. We expect to implement this feature in future versions of NGSEP. We also plan to further improve on genotyping accuracy in future versions of the algorithm.

Researchers performing population genomic studies usually trade read depth by the number of samples sequenced, looking for a balance that maximizes the cost-benefit of the sequencing effort [34, 35]. Thus, it is extremely important for SV detection tools to be able to produce accurate results from a low-depth input. One of the biggest advantages of the NGSEP algorithm, when compared to the other state-of-the-art tools, is that it is robust to reductions of read depth. Even at 20× average read depth, the integration of the Bayesian model provided the best results for genotyping accuracy in the HGSVC2 experiments, also demonstrating reliability for samples with different genetic diversity profiles. Additionally, this probabilistic model improved precision, which was evidenced by the analysis of the 47× ONT GIAB reads, which have a bigger error rate than CCS reads. This suggests that our algorithm is also robust to increased per-base error rates. Beyond tools comparison, our experiments indicate that an average read depth of around 20× is sufficient to achieve high detection and genotyping accuracy.

We believe that this work represents a significant contribution to current research on algorithms to analyze long DNA sequencing reads. We expect that the new functionality developed in NGSEP for SV detection from long reads will be useful for a large number of ongoing and upcoming research in population genomics for different species.

## Methods

### Software development and integration within NGSEP

The algorithm described in this article was implemented in Java 11 as a new option of the single sample variants detector functionality of the NGSEP software tool (RRID: SCR_012827). The reuse of different NGSEP classes significantly decreased the development effort needed to code. Initially, for computing the input file, a ReadAlignment iterator found in the ReadAlignmentFileReader class was used, given that it already collects all of the necessary information for each alignment. A Collection interface class named GenomicRegionSortedCollection allowed GenomicVariant interface implementing objects, such as Signature and CalledGenomicVariant objects, to be stored by sorted sequence (e.g., chromosomes) and by genomic position. This also facilitated computed spanning alignments to specific variants. Additionally, the work made for genotyping SVs consisted mostly of programming the functionality to estimate likelihoods, given that the class CountsHelper allowed calculating the genotype posterior probabilities, as it was implemented before to genotype small indels and SNPs. The class diagram for the functionality inside of the NGSEP class context is shown in the Supplementary Fig. S12.

### Simulation experiments

In order to assess the behavior of our algorithm to identify and genotype SVs, a thorough benchmarking process was established to evaluate performance metrics of recall, precision, and efficiency. After an in-depth literature revision, 4 tools were included in the benchmark based on their performance and impact, including SVIM (version 2.0.0) [15], Sniffles2 (version 2.2) [23], CuteSV (version 2.0.3) [25], and Dysgu (version 1.6.1) [8]. Both simulations and real cases were used to perform benchmark experiments. Output VCF files with SV calls were compared to gold-standard files using the software Truvari (version 4.1.0) [28], which provides recall, precision, F-score, and genotype accuracy of the evaluated SV genotype calls. This tool has been recommended by the GIAB consortium for benchmarking of SV callers [18]. Parameters for each dataset are provided in the Supplementary Table S2. In particular, we reduced the minimum read support parameter of CuteSV to 5× after performing parameter tuning experiments on the GIAB datasets (Supplementary Fig. S13).

SVs were simulated with the software VISOR [16], based on the *Arabidopsis thaliana TAIR10* reference genome [36]. A total of 4,330 structural variants with a minimum length of 50 bp were simulated (2,500 deletions, 1,830 insertions, and 2,065 inversions), and a genome containing these variants was generated. Next, reads with the characteristics of the Oxford Nanopore Sequencing Technology (ONT), including the error profile, were simulated with VISOR from this altered genome. Reads were aligned to the original reference genome using minimap2 [31]. This pipeline was repeated to simulate 4 datasets of varying depths, including 20×, 30×, 45×, and 60×. The resulting alignments were used as the input data for all tools. The Human simulation from the T2T genome [26] was produced following the exact same pipeline.

### GIAB high-confidence dataset

The GIAB consortium has produced a high-confidence curated SV dataset, consisting of indel SVs identified from many biotechnologies and multiple bioinformatic methods on the Ashkenazi son sample (HG002) against the GRCh37 reference genome [18]. All callers, including NGSEP, were used to discover SVs from a PacBio HiFi read alignment dataset of 56× depth and an ONT UL dataset of 47× depth, both sequenced from the same HG002 subject. Minimap2 [31] was used as the mapping tool to the GRCh37 reference genome. These alignments were randomly subsetted to produce 10×, 20×, 30×, and 40× input files in addition to the initial full-depth datasets, to assess the effect of depth variance on the calling algorithms. In order to include PBSV (version 2.9.0) [29], we had to realign reads from the original HiFi HG002 sample using the pbmm2 mapper (available with PBSV). See step-by-step instructions in the Supplementary File S2.

From the GIAB gold standard, we used 2 ground-truth benchmark datasets, one including repetitive regions called Tier 1+2 (T1+2), and another retaining only nonrepetitive regions, called Tier 1 (T1). We filtered these datasets retaining only SVs flagged with a “PASS” in the filter field of the VCF files and having length larger than 50 bp. The final number of SVs for each experiment can be found in Supplementary Table S1.

### HGSVC2 high-confidence samples

The work made by the HGSVC2 consortium provided high-confidence haplotype resolved calls for 3 samples of different ethnicities against the GRCh38 genome [19, 20]. Supplementary Table S1 shows the number of SVs of each type within each gold-standard dataset. PacBio HiFi reads were extracted from publicly available alignments for each of the 3 samples and were realigned with minimap2 [31]. From each one, a 20× depth set of randomly chosen alignments was produced as input for the aforementioned callers. See step-by-step instructions in Supplementary File S2.

For benchmarking using Truvari, we compared the results obtained keeping the default value of reciprocal overlap (70%) with those obtained reducing this parameter to 35% (-pct flag). We adjusted this parameter based on the initial results produced by Sniffles2. Similar to the experiments with the GIAB dataset, we also calculated the metrics using the complete dataset and compared them with those obtained including only SVs in nonrepetitive regions of the reference genome.

### Benchmark metrics

Truvari [28] was used to produce the benchmark metrics, using symbolic alleles only. Performance metric calculation is specified as follows:


\begin{eqnarray*}
&&{\textit{Precision} = \frac{{TP}}{{TP + FP}}} \qquad {\textit{Recall} = \frac{{TP}}{{TP + FN}}}\\ &&\textit{GTAccuracy} = \frac{{HOM_{TP}^{HOM} + HET_{TP}^{HET}}}{{HOM_{TP}^{HOM} + HOM_{TP}^{HET} + HET_{TP}^{HET} + HET_{TP}^{HOM}}}\\ &&{\textit{Fscore} = 2\frac{{\textit{Precision} \times \textit{Recall}}}{{\textit{Precision} + \textit{Recall}}}} \qquad {\textit{GTFscore} = 2\frac{{\textit{GTAccuracy} \times \textit{Recall}}}{{\textit{GTAccuracy} + \textit{Recall}}}}
\end{eqnarray*}

where *GTAccuracy* is a metric obtained by estimating the fraction of the correctly genotyped true-positive SVs over the total amount of true positives. Superscripts indicate their true genotype, which may differ from the caller classification. *GTFscore* is a variation of F-score, to combine correct genotype classification with recall as the harmonic mean between both values.

Truvari also allows the inclusion of SVs in the truth and test call sets if they are located inside the genomic regions annotated in an input bed. This allowed us to produce the separate Tier 1 and Tier 1+2 benchmarks for GIAB and the nonrepetitive regions and all regions for HGSVC2. Finally, this software does not take into account SVs with homozygous reference (0/0) genotype calls.

### Execution environments

*Arabidopsis* simulation software executions, including running all SV callers, were done on an 8-core Ryzen 7 5800H with 16 Gb RAM laptop. Analysis of the human T2T simulation, the GIAB benchmark, and the HGSVC2 benchmark was performed on an Intel Xeon Gold computing node with a capacity of 42 threads and 565 GB RAM. Most of this computing power was required to align reads to the reference genomes. Processes for variants detection were restricted to a single core and 16 Gb of RAM.

## Availability of Supporting Source Code and Requirements

The algorithm presented in this study can be executed through the Single Sample Variants Detector functionality of the open-source software Next Generation Sequencing Experience Platform (NGSEP). Releases of NGSEP are available at SourceForge [37]. Life development is available on GitHub [38]. The following are full details of the availability of supporting source code and requirements:

Project name: Next Generation Sequencing Experience Platform (NGSEP)

Project homepage: http://ngsep.sf.net

Operating system(s): Platform independent

Programming language: Java

Other requirements: Java 11 or higher

License: GNU GPL

RRID: SCR_012827

Biotools ID: NGSEP

## Supplementary Material

giad112_GIGA-D-23-00070_Original_Submission

giad112_GIGA-D-23-00070_Revision_1

giad112_GIGA-D-23-00070_Revision_2

giad112_GIGA-D-23-00070_Revision_3

giad112_Response_to_Reviewer_Comments_Original_Submission

giad112_Response_to_Reviewer_Comments_Revision_1

giad112_Response_to_Reviewer_Comments_Revision_2

giad112_Reviewer_1_Report_Original_Submission Kez Cleal -- 4/17/2023 Reviewed

giad112_Reviewer_1_Report_Revision_1 J Kez Cleal -- 8/14/2023 Reviewed

giad112_Reviewer_1_Report_Revision_2 Kez Cleal -- 10/13/2023 Reviewed

giad112_Reviewer_2_Report_Original_Submission Fritz J Sedlazeck -- 5/2/2023 Reviewed

giad112_Reviewer_3_Report_Original_Submission Ryan Layer -- 5/8/2023 Reviewed

giad112_Reviewer_4_Report_Original_Submission Xuefang Zhao -- 5/9/2023 Reviewed

giad112_Supplemental_Files

## Data Availability

The *A. thaliana* TAIR10 reference genome used for simulations is available in the phytozome v.12 database [39]. The GIAB SV gold-standard VCF file can be downloaded from the GIAB website [27] as well as the bed files containing tier information. The GHC37 human reference genome can be found in the NCBI Assembly database [40] with accession number GCA_000001405.1. PacBio HiFi reads are available at the sequence read archive database of NCBI [41] with BioProject accession number PRJNA586863. Oxford nanopore reads are located at the European Nucleotide Archive [42] under accession PRJEB37264. Assets for the HGSVC2 benchmark are found on the project page [30]. PB HiFi read files for the 3 samples are listed on this website and deposited in the EBI ftp site [43]. Specifically, the GRCH38 reference genome [44] and the vcf, which contains the gold-standard SVs for the 3 samples [45], are available at the 1000 Genomes project repository. An archival copy of the code and supporting data is also available via the *GigaScience* database, GigaDB [46].

## Additional Files

**Supplementary File S1**. Accuracy and efficiency measures for the benchmark experiments presented in this study.

**Supplementary File S2**. Step-by-step instructions to prepare and run the experiments with human benchmark datasets.

**Supplementary File S3**. Supplementary tables and figures.

**Supplementary Table 1**. Number of SVs included in each gold-standard dataset.

**Supplementary Table 2**. Commands executed to test each tool evaluated in this study. For all callers, we filtered the output vcf files with the commands specified in the second to last row. This prevented Truvari from counting duplications as false positives instead of true positive insertions. We also filtered out inversions in the benchmarks with real data because the gold standard sets do not include inversions.

**Supplementary figure 1**. Human T2T Indel Simulation The shape of points represents depths for fixed values of 5x, 10x, 20x, 30x, 40x, and 60x. A. Precision-recall curves of SV detection for alignments at different depths. B. F-score as a function of sequencing depth. The 20x F-score values are as follows: NGSEP: 96.96, Sniffles: 98.34, SVIM: 39.3, Dysgu: 90.22, CuteSV: 97.55. For SVIM, a QS filter > 0 was applied. C. Single thread execution time of all callers as a function of the depth of the input alignments. An apparent linear time complexity is observed for each dataset of increasing sizes (5x: 21Gb, 10x: 42Gb, 20x: 84Gb, 30x: 126Gb, 40x: 167G, 60x: 250Gb), except for Dysgu from 40x to 60x.

**Supplementary figure 2**. Benchmark HG002. Only tier 1. A, C Precision-recall curves of SV discovery over varying depths for all callers restricted to T1 regions on A. HiFi data, and C. ONT data. B, D show curves comparing genotyping accuracy with recall on B. HiFi data and D. ONT data. F-score values for the 20x (HiFi, ONT) depth mappings are: NGSEP: 94.49, 86.49; Sniffles: 93.91, 90.24; SVIM: 88.33, 11.92; Dysgu: 95.47, 90.73; CuteSV: 85.49, 89.18

**Supplementary figure 3**. Benchmark HG002. Only Insertions Tier 1 + 2. A, C Insertion only precision-recall curves of SV discovery over varying depths for all callers restricted to T1+2 regions on A. HiFi data, and C. ONT data. B, D show curves comparing genotyping accuracy with recall on B. HiFi data and D. ONT data.

**Supplementary figure 4**. Benchmark HG002. Only deletions Tier 1 + 2. A, C Deletion only precision-recall curves of SV discovery over varying depths for all callers restricted to T1+2 regions on A. HiFi data, and C. ONT data. B, D show curves comparing genotyping accuracy with recall on B. HiFi data and D. ONT data.

**Supplementary figure 5**. Benchmark HG002. Only Insertions Tier 1 A, C Insertion only precision-recall curves of SV discovery over varying depths for all callers restricted to T1 regions on A. HiFi data, and C. ONT data. B, D show curves comparing genotyping accuracy with recall on B. HiFi data and D. ONT data.

**Supplementary figure 6**. Benchmark HG002. Only deletions Tier 1. A, C Deletion only precision-recall curves of SV discovery over varying depths for all callers restricted to T1 regions on A. HiFi data, and C. ONT data. B, D show curves comparing genotyping accuracy with recall on B. HiFi data and D. ONT data.

**Supplementary figure 7**. Benchmark HiFi HG002. Including pbsv. A, C Precision-recall curves of SV discovery over varying depths of HiFi mappings, for all callers including pbsv restricted to A. T1+2, and C. T1 regions. B, D show curves comparing genotyping accuracy with recall on B. T1+2, and D. T1. F-score values for the 20x (T1+2, T1) depth mappings are: NGSEP: 71.44, 93.89; Sniffles: 69.49, 93.33; SVIM: 58.72, 87.88; Dysgu: 71.55, 93.72; CuteSV: 72.45, 91.94; pbsv: 69.68, 92.96.

**Supplementary figure 8**. Time and peak memory usage from HG002 benchmarking A. Execution time of all callers under the pbmm2 aligned reads at varying input depths. Different from the recorded times of the simulations, all tools perform similarly, increasing at a linear rate. Only pbsv shows a sudden spike from 40 to 56x. B. Java Virtual Machine assessment of peak memory consumption for NGSEP caller executions at different depths of input. Peak memory usage was recorded through executions of the software, and dissected into two types. NAP stands for New allocations peak, which showcases the heap memory assigned to recently created objects. RHP for Remaining heap peak, representing objects that remain in memory, and have not been removed by the garbage collector. As can be seen, for low-depth inputs (5, 10, 20x) total memory consumption is below 8 Gb. From 30x onwards, old objects occupy most of the memory and even reach total memory consumption for the complete mappings. Nonetheless, new objects still consume minimal memory, showcasing that the algorithm is able to handle inputs close to 60x depth for human genomes with a 16Gb memory limit, which is consistent with the executions on simulated human reads.

**Supplementary figure 9**. Benchmark HGSVC2 half pct (0.35) parameter. HGSVC2 Benchmark experiments on 20x depth HiFi mappings for each sample (A. HG00514: Han Chinese, B. HG00733: Puerto Rican, C. NA19240: Yoruba from Nigeria). These results come from altering the reciprocal overlap parameter (-pct 0.35) in Truvari to favor Sniffles2, due to an error in their output. All performance metrics are shown based on the results of the tested variant callers, and their exact percentage values are portrayed over each column.

**Supplementary figure 10**. Benchmark HGSVC2 default parameter non-repetitive regions HGSVC2 Benchmark experiments on 20x depth HiFi mappings for each sample (A. HG00514: Han Chinese, B. HG00733: Puerto Rican, C. NA19240: Yoruba from Nigeria). Tested SVs are restricted to non-repetitive regions. All performance metrics are shown based on the results of the tested variant callers, and their exact percentage values are portrayed over each column.

**Supplementary figure 11**. Benchmark HGSVC2 hal pct (0.35) parameter non-repetitive regions HGSVC2 Benchmark experiments on 20x depth HiFi mappings for each sample (A. HG00514: Han Chinese, B. HG00733: Puerto Rican, C. NA19240: Yoruba from Nigeria). These results come from altering the reciprocal overlap parameter (-pct 0.35) in Truvari to favor Sniffles2, due to an error in their output. Additionally, SVs are restricted to non-repetitive genome regions.

**Supplementary figure 12**. NGSEP SVCaller functionality UML diagram. Class diagram of the functionality as a UML integrated into NGSEP. The generic algorithm classes are designed so that they can be used for other problems and applications. The clustering detection classes adapt them for the SV clustering functionality dictated by an interface, designed to guide the solution to this problem.

**Supplementary figure 13**. Experiments changing min read support for CuteSV. The experiments were performed on (A,B) HiFi and (C,D) ONT reads of the benchmark sample HG002, varying the min read support from 1x to 20x. HiFi reads were realigned using pbmm2. Panels A and C show the results for T1 regions and panels B and D show the results for T1+2 regions.

## Abbreviations

BFS: breadth first search; CCS: circular consensus sequencing; GIAB: Genome In A Bottle; HTS: high-throughput sequencing; LCR: low copy repeat; LSC: longest soft clip; NAHR: nonallelic homologous recombination; NGSEP: Next Generation Sequencing Experience Platform; QTL: quantitative trait loci; SNP: single-nucleotide polymorphism; SV: structural variant; UL: ultra-long; WGD: whole-genome duplication; WGS: whole-genome sequencing.
