# Correction to: A graph clustering algorithm for detection and genotyping of structural variants from long reads

**DOI:** 10.1093/gigascience/giae077

**Published:** 2024-10-17

This is a correction to: Nicolás Gaitán, Jorge Duitama, A graph clustering algorithm for detection and genotyping of structural variants from long reads, *GigaScience*, Volume 13, 2024, https://doi.org/10.1093/gigascience/giad112

In the originally published version of this manuscript, the ethnicity of the sample HG00733 from Puerto Rico and NA19240 were switched by mistake. Emendation has been made, fixing the instances in which the ethnicity of these samples is mentioned.

Each emendation is underlined:

1. **Introduction**, sixth paragraph, last part of the second sentence: “[…] including Han Chinese, Puerto Rican, and Yoruba from Nigeria […]”.

2. Section **Benchmarking with the HGSVC2 samples**, second paragraph, last sentence: “In particular, NGSEP achieved the best genotyping accuracy for the admixed Puerto Rican individual (HG00733), […]”

3. Caption to figure 6: […] (B) HG00733: Puerto Rican, and (C) NA19240: Yoruba from Nigeria. […]”

4. Caption to supplementary figure 9: […] HG00733: Puerto Rican, C. NA19240: Yoruba from Nigeria). […]”

5. Caption to supplementary figure 10: “[,…] HG00733: Puerto Rican, C. NA19240: Yoruba from Nigeria). Tested SVs are restricted to non-repetitive regions. All performance metrics are shown based on the results of the tested variant callers, and their exact percentage values are portrayed over each column.”

6. Caption to supplementary figure 11:”[…]HG00733: Puerto Rican, C. NA19240: Yoruba from Nigeria).[…]”

The corrections have been made in the article.

