# Supplementary material for: A graph clustering algorithm for detection and genotyping of structural variants from long reads
Source: Gigascience. 2024 Jan 11;13:giad112. doi: 10.1093/gigascience/giad112 (PMC10783151; doi:10.1093/gigascience/giad112)
Supplement: giad112_GIGA-D-23-00070_Original_Submission [file giad112_giga-d-23-00070_original_submission.pdf]

GigaScience

A graph clustering algorithm for detection and genotyping of structural variants from long reads

--Manuscript Draft--

**Manuscript Number:**	GIGA-D-23-00070
**Full Title:**	A graph clustering algorithm for detection and genotyping of structural variants from long reads
**Article Type:**	Technical Note
**Funding Information:**	Ministerio de Ciencia, tecnología e innovación de Colombia (80740-441-2020) — Dr. Jorge Duitama
**Abstract:**	Structural variants (SV) are genomic polymorphisms defined by their length (>50 bp). The usual types of SVs are deletions, insertions, translocations, inversions, and copy number variants. SV detection and genotyping is fundamental given the role of SVs in phenomena such as phenotypic variation and evolutionary events. Thus, methods to identify SVs using long-read sequencing data have been recently developed. We present an accurate and efficient algorithm to predict SVs from long-read sequencing data. The algorithm starts collecting evidence (Signatures) of SVs from read alignments. Then, signatures are clustered based on a Euclidean graph with coordinates calculated from lengths and genomic positions. Clustering is performed by the DBSCAN algorithm, which provides the advantage of delimiting clusters with high resolution. Clusters are transformed into SVs and a Bayesian model allows to precisely genotype SVs based on their supporting evidence. This algorithm is integrated into the single sample variants detector of the Next Generation Sequencing Experience Platform (NGSEP), which facilitates the integration with other functionalities for genomics analysis. Benchmarking experiments show that our approach outperformed state-of-the-art tools on SV calling and genotyping, especially at 20x depth on a simulated dataset with an F-score of 99.1%, and on the HG002 GIAB PacBio HiFi sample with an F-score of 94.5% and 96% GT-Accuracy. This shows that our algorithm reliably detects and genotypes most SV events. We believe this work significantly contributes to the development of bioinformatic strategies to maximize the use of long-read sequencing technologies.
**Corresponding Author:**	Jorge Duitama<br><br>COLOMBIA
**Corresponding Author Secondary Information:**	
**Corresponding Author's Institution:**	
**Corresponding Author's Secondary Institution:**	
**First Author:**	Nicolás Gaitán
**First Author Secondary Information:**	
**Order of Authors:**	Nicolás Gaitán<br>Jorge Duitama
**Order of Authors Secondary Information:**	
**Additional Information:**	
**Question**	**Response**
Are you submitting this manuscript to a special series or article collection?	No
**Experimental design and statistics**	Yes

Powered by Editorial Manager® and ProduXion Manager® from Aries Systems Corporation

Full details of the experimental design and statistical methods used should be given in the Methods section, as detailed in our Minimum Standards Reporting Checklist. Information essential to interpreting the data presented should be made available in the figure legends.<br><br>Have you included all the information requested in your manuscript?	
**Resources**<br><br>A description of all resources used, including antibodies, cell lines, animals and software tools, with enough information to allow them to be uniquely identified, should be included in the Methods section. Authors are strongly encouraged to cite Research Resource Identifiers (RRIDs) for antibodies, model organisms and tools, where possible.<br><br>Have you included the information requested as detailed in our Minimum Standards Reporting Checklist?	Yes
**Availability of data and materials**<br><br>All datasets and code on which the conclusions of the paper rely must be either included in your submission or deposited in publicly available repositories (where available and ethically appropriate), referencing such data using a unique identifier in the references and in the "Availability of Data and Materials" section of your manuscript.<br><br>Have you have met the above requirement as detailed in our Minimum Standards Reporting Checklist?	Yes

Powered by Editorial Manager® and ProduXion Manager® from Aries Systems Corporation

Manuscript

Click here to access/download;Manuscript;NGSEPSVsLongReads_GigaSci

A graph clustering algorithm for detection and genotyping of structural variants from long reads

Nicolás Gaitán[1], Jorge Duitama[1,*].

[1]Systems and Computing Engineering Department, Universidad de Los Andes, Bogotá, Colombia.

* Corresponding author. E-mail: ja.duitama@uniandes.edu.co

ABSTRACT

Structural variants (SV) are genomic polymorphisms defined by their length (>50 bp). The usual types of SVs are deletions, insertions, translocations, inversions, and copy number variants. SV detection and genotyping is fundamental given the role of SVs in phenomena such as phenotypic variation and evolutionary events. Thus, methods to identify SVs using long-read sequencing data have been recently developed. We present an accurate and efficient algorithm to predict SVs from long-read sequencing data. The algorithm starts collecting evidence (Signatures) of SVs from read alignments. Then, signatures are clustered based on a Euclidean graph with coordinates calculated from lengths and genomic positions. Clustering is performed by the DBSCAN algorithm, which provides the advantage of delimiting clusters with high resolution. Clusters are transformed into SVs and a Bayesian model allows to precisely genotype SVs based on their supporting evidence. This algorithm is integrated into the single sample variants detector of the Next Generation Sequencing Experience Platform (NGSEP), which facilitates the integration with other functionalities for genomics analysis. Benchmarking experiments show that our approach outperformed state-of-the-art tools on SV calling and genotyping, especially at 20x depth on a simulated dataset with an F-score of 99.1%, and on the HG002 GIAB PacBio HiFi sample with an F-score of 94.5% and 96% GT-

Accuracy. This shows that our algorithm reliably detects and genotypes most SV events. We believe this work significantly contributes to the development of bioinformatic strategies to maximize the use of long-read sequencing technologies.

INTRODUCTION

Structural variants (SV) are a type of genetic polymorphism, in both coding and non-coding sequences, which are usually defined by their length (>50 bp). The main types of SVs are deletions, insertions, translocations, inversions, and copy number variants (Alkan et al., 2011). The main genomic processes that cause the formation of structural variants are DNA recombination, replication, and repair-associated processes (Carvalho et al., 2016). For example, one common mechanism is Non-Allelic Homologous Recombination (NAHR) which is a genetic repair mechanism in which misalignment of previously duplicated regions called low copy repeats (LCR) occurs during meiosis. This subsequently causes a genomic rearrangement event on another locus that does not belong to the LCR gene, thus creating further deletions or duplications (Parks et al., 2015).

The interest in SVs comes mainly from the functional consequences of their genetic diversity. It has been proven that many SVs are involved in different gene expression patterns and influence different characteristics. SVs that are located adjacent to genes may structurally affect *cis*-regulatory regions by position or composition, leading to either silencing or increasing gene expression, which explains variation of Quantitative Trait Loci (QTL)(Chiang et al., 2017). For example, Alonge *et al.* (2020) found that at least 50% of the SVs found in an assessment of around 100 lines of tomato were associated with gene expression regulatory processes, mostly causing reductions or even silencing of gene products. Another case is when duplications increase the amount of overall transcript-protein production by gene dosage effect. This has proven beneficial for artificial selection in certain plant species where the average size

of fruits increased because the plant variant suffered a specific duplication in a cytochrome coding gene (Alonge et al., 2020).

Structural variants also provide fundamental information about evolutionary relationships between organisms and their natural history. Many Whole-Genome Sequencing (WGS) studies have been conducted to assess the prevalence of different SVs and their variation in organisms, populations, or species. In plants, analyzing structural variants allowed elucidation of the dynamics of whole-genome duplication (WGD) events and their evolutionary role (Qiao et al., 2019). WGDs are followed by a fast diploidization process, mainly because most of the duplicated genes become paralogs (Qiao et al., 2019). Furthermore, many components of the C4 metabolic pathway were brought by these WGD events and single duplication events. This is an interesting case of convergence throughout the evolution of different plant lineages (Wang et al., 2009). These changes are influenced by the synergistic effect of WGDs, transposed duplication, and dispersed gene duplication, evidenced by overlapping peaks in the rates of synonymous substitutions (Qiao et al., 2019). This shows how SVs can provide substantial amounts of evidence for evolutionary studies.

Given the importance of SVs, a large number of computational methods have been developed to identify and genotype SVs, based on high throughput sequencing (HTS) data. Most of these SV detection tools are based on short-read sequencing technologies (Cleal et.al., 2022; Sarwal et.al., 2022). This presents many limitations, mostly due to the length of structural variants, which usually exceeds the read length, which reduces the precision of both identification and genotyping (Luan et.al., 2020, Mahmoud et.al., 2019). Recently, new SV calling tools have adopted long reads as their input data, significantly increasing the accuracy of SV detection in comparison with short read-based callers (Mahmoud et.al., 2019; Schwarz et.al., 2021). This has allowed many researchers to increase their catalog of functionally relevant structural variants, including some that affect the pathophysiology of diseases such as human cancer

(Fujimoto et.al., 2021; Thibodeau et.al., 2020). However, further improvements could be achieved by novel algorithmic techniques. Some difficulties arise even when long reads are used. Since SV detection relies on accurate read alignment, dissimilar, partial, or inaccurate read alignments obscure the signal to perform a consistent detection and genotyping of SVs. Thus, the results also depend on the accuracy of the aligner software (Heller et.al., 2019). Additionally, from a software design point of view, our experience indicates that most current tools are difficult to operate because they require a large number of specific libraries and versions, their implementations are not debugged correctly and exceptions are not handled appropriately. For short read-based callers, these limitations have been described by a recent benchmark study by Sarwal et.al (2022).

Benchmarking SV detection has been a difficult task to perform. There are few independently validated gold standard datasets for real sequencing data because experimental validation is difficult to perform at a large scale. Consequently, there is no consensus on which of the existing tools produces the closest result to a gold standard set. Bolognini et.al (2020) addressed this issue by implementing a simulation software called VISOR, which produces a complete haplotype-resolved sample genome and simulates read alignments from a list of SVs, with either Oxford Nanopore or PacBio error profiles. Trying to optimize the SV calling pipeline, Jiang et.al (2021) evaluated the accuracy of different SV callers using VISOR simulations on real reported human SVs. For the 20x simulated dataset, they report that the best tools are CuteSV (F1=0.8), SVIM (F1=0.798), and Sniffles (0.769). Additionally, they provide recommendations for SV calling best practices such as sequencing experiments with read lengths of about 20 kb at 20x depth. Regarding real datasets, the most widely recognized and best-curated case is the high-confidence structural variant dataset (Sample HG002 on reference genome GRCh37) from the Genome In A Bottle human sample project (GIAB) crafted for benchmarking (Zook et.al.,2020). The events reported in this file come from a mixture of

sequencing technologies and have been predicted by using a pipeline integrating many different tools.

Structural variant detection provides the possibility of finding biological insights with many different functional consequences. In this manuscript, we developed a new software solution that improves the detection of SVs from long read alignments using the DBSCAN algorithm to solve the clustering problem, and implements a new bayesian genotyping model. This functionality is integrated into the bioinformatic software suite (NGSEP) to further facilitate the analysis of genomic data.

RESULTS

A new clustering algorithm for detection and genotyping of Structural Variants

The process of structural variant detection and genotyping starts from reads aligned to a reference genome and is divided into three main stages described as follows.

1. Signature Collection

The main input to this algorithm is a set of read alignments in SAM or BAM format, obtained from mapping long reads to a reference genome. Signatures are individual signals of a structural variant that are contained within each read alignment or constructed from discordant partial alignments. They can be divided into intra-alignment and inter-alignment signatures. Intra-alignment signatures consist of evidence of deletions or insertions that are predicted as part of the read alignment process. Thus, these signatures are collected by reading the description of the alignment (encoded in the CIGAR field of the SAM format) to find signals of insertion or deletion. Conversely, reads with multiple discordant alignment segments, regarding their position or orientation, are selected to identify inter-alignment signatures.

Figure 1 shows the procedures that we implemented for the recollection of signatures for each SV type. Intra-alignment deletions and insertions are identified by parsing the CIGAR strings, and searching for their codes (e.g. D or I, respectively), The CIGAR code includes the length

of each event within the alignment. Inter-alignment deletions are suspected when unmapped regions in the reference genome are flanked by partial alignments. For each read with two partial alignments within the same chromosome region, the reference distance between the end of the first partial alignment and the beginning of the second alignment in reference genomic coordinates is considered the length of the deletion signature. Inter-alignment insertion signatures are identified from reads with two adjacent alignments, having a soft clip starting from the presumed insertion point. For each read, the Longest Soft Clip (LSC) is calculated by taking the maximum of soft clips at the end of each alignment. The length of the partial alignment that does not contain the LSC is subtracted from the length of the LSC to estimate the length of the insertion signature. Inversions appear as three consecutive partial alignments where the middle alignment has an opposite orientation, compared to the two flanking alignments. The length of the inversion is predicted as the length of the middle alignment. Signatures are filtered from the minimum SV length specified by the user (default ≥50 bp) and are added to a collection, which is sorted by chromosome and reference coordinates. The process to identify duplications happens later on in the algorithm because they are classified from already called Insertion SVs if the intra-alignment signatures that allowed its discovery differ significantly in genomic positions.

2. *Signature Clustering*

Given a set of SV signatures, we implemented a graph-based clustering in which each cluster becomes a candidate SV event. A graph is built independently for each signature type. The vertices of the graph correspond to the input collection of signatures identified in the previous step. Each signature is represented by a tridimensional vector with three numeric values: Start coordinate in the reference genome (B_i) end coordinate in the reference genome (E_i), and signature length (L_i). The cost m_{ij} of the edge between two signatures i and j corresponds to the Euclidean distance of their corresponding vectors:

$$FPD_{ij} = |B_j - B_i| \qquad LPD_{ij} = |E_j - E_i| \qquad LD_{ij} = |L_j - L_i|$$

$$m_{ij} = \sqrt{FPD_{ij}^2 + LPD_{ij}^2 + LD_{ij}^2}$$

The DBSCAN algorithm is a non-supervised clustering procedure for n-dimensional vectors (points) based on the principle of density-based grouping (Schubert et.al., 2017). The parameters of this algorithm are a threshold *epsilon* (ε) which limits the distance for considering two points as neighbors, and a minimum number of neighbors (minPts) that a point should have to be considered a *core point*. The lemma states that considering a cluster that contains certain *core points,* then any point which is density reachable from any of those *core points* (in the graph context, any point that has a path from any *core point*) will be considered as part of the cluster. Any point that is not reachable from any *core point* will be considered a noise signal. The procedure to implement this algorithm was as follows. Starting from an initially complete graph with *n* points, the algorithm eliminates the edges where m_{ij} is bigger than or equal to ε. Then, each point is visited to test if its number of neighbors is at least *minPts,* in which case it is labeled as a core point. Consequently, a new cluster is initialized with the core point and its direct neighbors, and a Breadth First Search (BFS) is performed by pushing this neighborhood into a queue where each point will also be queried for its neighbors to assess the *core point* property presumption, repeating this process until all of the density reachable points from any core point in the cluster are visited. If there are unvisited points, the procedure continues until all points are visited. Figure 2 shows the main steps and restrictions of this procedure.

3. *Cluster to Genotyped SV*

Each signature cluster identified in the previous step becomes a candidate SV. The last step of the process is the genotyping of these candidates. To identify SV coordinates, the average of the first reference coordinates of the signatures within the cluster is estimated. The last coordinate is calculated likewise. The length is taken as the difference between both the last

and first SV coordinates, except for insertions where the average length of the cluster signatures is estimated as the average of the insertion lengths of the signatures. Candidate SVs are stored in a collection sorted by reference coordinates. Then, a Bayesian genotyping process is performed for each candidate SV by reassessing the evidence that read alignments provide. To avoid having to reprocess the alignments file, a collection of compact alignment objects is kept in memory from the first stage, having the minimum possible information needed for this step. For each SV, intersecting read alignments are collected, and those containing clustered signatures are considered supporting evidence for the alternative allele hypothesis. If the spanning read alignment contains no signatures, it is counted as a supporting call for the reference allele. Figure 3 shows the estimation of the likelihood for the four possible scenarios, generated from the combination of the hypotheses, the two plausible alleles from which the read could be sequenced (SV or REF alleles), with calls from a read alignment that may or may not support these allele hypotheses. The distribution of lengths of the clustered signatures supporting the SV hypothesis is used to estimate the likelihood of a read alignment supporting this SV. In this case, it is assumed that the read was actually sequenced from a chromosome affected by the SV (case 1). If a reference allele is assumed (case 2), a read with an SV signature is proposed to have happened by a misalignment or sequencing error and a fixed value (0.0001 by default) is used as likelihood. The likelihood of a read supporting the reference allele that is assumed to be sequenced from a haplotype affected by the SV is calculated as the probability of having an indel error that reverts the SV and is also a constant value (0.001 by default) (case 3). Finally, a fixed value (default 0.999) is used for the likelihood of a read supporting the reference allele assuming sequencing from a reference haplotype.

Read likelihoods for each allele hypothesis are transformed into posterior probabilities for each possible genotype following the same procedure implemented in NGSEP to perform SNP genotyping (Gil et al., 2021). The hypothesis having the largest posterior probability is

assigned as the predicted genotype. Similar to SNP genotyping, the quality of such SV calls will be the phred score Q corresponding to the genotype posterior probability.

Benchmarking with simulation experiments

We performed two simulations of structural variants in the genome of *Arabidopsis thaliana* using the tool VISOR (Bolognini et.al., 2020). 1718 insertions and 2532 deletions, and 2065 inversions were generated for benchmark experiments. Read alignment subsets were produced for 20x, 30x, 45x, and 60x. The precision-recall results of our NGSEP algorithm were compared to those of state-of-the-art tools, including SVIM (Heller et.al., 2019), Sniffles (Sedlazeck et.al., 2018), CuteSV (Jiang et.al., 2020) and Dysgu (Cleal et.al., 2022). After obtaining the metrics for both simulation experiments, precision-recall curves and F-score against depth were plotted for each tool. Additionally, execution times for each depth dataset were evaluated for single-thread runs (Supplementary file 1).

Figure 4A shows that the NGSEP algorithm presented above outperforms all of the tools for depths of 20x, 30x, and 45x, according to F-score values. Only in the 60x test, CuteSV achieves an equal value. Dysgu suffers from a significant drop in precision, which decreases the F-score for bigger depths. Figure 4B shows that only NGSEP keeps high performance for varying alignment depths in both precision and recall. Although Dysgu outperforms all of the tools in terms of recall for each coverage, it reduces precision below 95% as depth increases. In the inversion simulation benchmark, SVIM produced the highest F-score, closely followed by the NGSEP algorithm (Figure 4C) while most of the other algorithms fail to accurately detect most inversions.

Single-thread runtimes were recorded for all experiments to compare the tools in terms of computational efficiency. As shown in Figure 4D, all of them follow a linearly increasing trend. Sniffles and NGSEP consistently required lower execution times compared to the other tools. It is worth clarifying that Sniffles, CuteSV, and Dysgu support multithreading which

significantly reduces runtimes at the cost of processing resources. Dysgu was the worst-performing tool in terms of computational efficiency, requiring about three times more execution time than the NGSEP algorithm.

Benchmarking with the Genome In a Bottle human genome

To assess the performance of our method on real datasets, we performed multiple experiments using reads from the Genome In a Bottle (GIAB) human individual HG002, for which a gold standard set of large indel calls is publicly available. Both 56x PacBio HiFi CCS (Circular Consensus Sequencing), and 47x ONT UL (Ultra Long Reads) reads sequenced from the HG002 subject were randomly sampled at average read depths of 10x, 20x, 30x, and 40x to perform experiments varying sequencing depth. Truvari (English et.al., 2022) was used to obtain precision and recall metrics of test calls against the gold standard, which was restricted to the Tier 1 regions and PASS-only SVs. Additionally, a F-score variation called GTFscore was also estimated to assess their performance regarding the combination of genotyping accuracy and recall. Further details for this metric are provided in the methods section.

For the PacBio HiFi varying depth experiments, Figure 5A shows that the best-performing tool in terms of F-score is NGSEP for 10x and 20x depth (See exact values in the Supplementary file 1). The precision of the NGSEP algorithm is also superior to that of the other tools, except Sniffles and CuteSV when they are executed on subsets with more than 30x depth. Regarding GTF-Score, NGSEP has also the highest value at 10x and 20x depth (Figure 5B). The NGSEP algorithm is robust to changes in the average read depth. Conversely, at increasing read depths Dysgu and especially SVIM improve recall at a high cost on precision. For ONT data, NGSEP keeps precision, recall, and genotyping accuracy over 80%, even at 10x read depth. However, Sniffles achieves the best results overall. The behavior of all tools is relatively consistent with that observed on the HiFi data, but the absolute values are consistently lower, probably due to the higher error rate of ONT reads.

DISCUSSION

The availability of long-read sequencing technologies represented a big step forward toward the accurate identification and genotyping of structural variants (Fujimoto et.al., 2021; Thibodeau et.al., 2020). Achieving this goal is becoming a requirement for current genomics, given the documented role of SVs as drivers of phenotyping variability and evolution (Alonge et al., 2020; Gorkovskiy et.al., 2021; Qiao et al., 2019; Wang et al., 2009). In this work we present the results of our efforts to develop novel algorithmic techniques, aiming to increase the accuracy of both discovery and genotyping of SVs. Transforming the problem of clustering SV signatures into a geometric clustering problem in an Euclidean space, allowed us to build a solution based on the well-known DBSCAN clustering algorithm (Schubert et.al., 2017) to identify SVs. Previous experiences implementing Bayesian models for SNV genotyping, allowed us to increase the accuracy of SV identification and provided a framework for SV genotyping. Benchmarking experiments running simulations and analyzing the GIAB dataset indicate that our algorithm achieves competitive accuracy compared to current software solutions.

Researchers performing population genomic studies usually trade read depth by the number of samples sequenced, looking for a balance that maximizes the cost-benefit of the sequencing effort (Cericola et.al., 2018; Fumagalli et.al., 2013). Thus, it is extremely important for SV detection tools to be able to produce accurate results from a low-depth input. One of the biggest advantages of the NGSEP algorithm, when compared to the other state-of-the-art tools, is that it is robust to reductions of read depth. Even at 20x average read depth, the integration of the Bayesian model provided the best results for genotyping accuracy. This outcome was also observed in the analysis of the 47x ONT reads, which have a bigger error rate than CCS reads. This suggests that our algorithm is also robust to increased per-base error rates. Beyond tools

comparison, our experiments indicate that an average read depth of around 20x is sufficient to achieve high detection and genotyping accuracy.

We believe that this work represents a significant contribution to current research on algorithms to analyze long DNA sequencing reads. We expect that the new functionality developed in NGSEP for SV detection from long reads will be useful for a large number of ongoing and upcoming research in population genomics for different species.

METHODS

Software development and integration within NGSEP

The algorithm described in this manuscript was implemented in Java 11 as a new option of the single sample variants detector functionality of the NGSEP software tool. The reuse of different NGSEP classes significantly decreased the development effort needed to code. Initially, for computing the input file a ReadAlignment iterator found in the ReadAlignmentFileReader class was used, given that it already collects all of the necessary information for each alignment. A Collection interface class named GenomicRegionSortedCollection allowed GenomicVariant interface implementing objects, such as Signature and CalledGenomicVariant objects, to be stored by sorted sequence, e.g chromosomes, and by genomic position. This also facilitated computed spanning alignments to specific variants. Additionally, the work made for genotyping SVs consisted mostly of programming the functionality to estimate likelihoods, given that the class CountsHelper allowed calculating the genotype posterior probabilities, as it was implemented before to genotype small variants and SNPs. The class diagram for the functionality inside of the NGSEP class context is shown in Figure 6.

Simulation experiments

In order to assess the behavior of our algorithm to identify and genotype SVs, a thorough benchmarking process was established to evaluate performance metrics of recall, precision, and efficiency. After an in-depth literature revision, four tools were included in the benchmark

based on their performance and impact, including SVIM (version 2.0.0) (Heller et.al., 2019), Sniffles 2 (version 2.0.6) (Sedlazeck et.al., 2018), CuteSV (version 1.0.13) (Jiang et.al., 2020) and Dysgu (version 1.3.11) (Cleal et.al., 2022). Both simulations and real cases were used to perform benchmark experiments. Output VCF files with SV calls were compared to Gold Standard files using the software Truvari (English et.al., 2022), which provides recall, precision, f-score, and genotype accuracy of the evaluated SV genotype calls. This tool has been recommended by the GIAB consortium for benchmarking of SV callers (Zook et.al., 2020). Parameters for each dataset are provided in the supplementary file 2.

SVs were simulated with the software VISOR (Bolognini et.al., 2020), based on the *Arabidopsis thaliana TAIR10* reference genome (Lamesch et.al., 2012). A total of 4,330 indel structural variants with a minimum length of 50 bp were simulated (2500 deletions, 1830 insertions), and a genome containing these variants was generated. Next, reads with the characteristics of the Oxford Nanopore Sequencing Technology (ONT), including the error profile, were simulated with VISOR from this altered genome. Reads were aligned to the original reference genome using minimap2 (Li et.al., 2018). This pipeline was repeated to simulate four datasets of varying depths, including 20x, 30x, 45x, and 60x. The resulting alignments were used as the input data for all tools.

GIAB high-confidence dataset

The Genome in a Bottle (GIAB) consortium has produced a high-confidence curated SV dataset, consisting of 9284 indels SVs called from many different sequencing technologies and discovered by different bioinformatic methods on the Ashkenazi son sample (HG002) compared to the GRCh37 reference genome (Zook et.al., 2020). To perform benchmarking based on this asset, indel variants of more than 50 bp in length, which fulfill the PASS filter (the most reliable calls) and that are located within Tier 1 regions (High confidence genomic regions) were used for the reference Gold Standard. All callers, including NGSEP, were used

to discover SVs from a PacBio CCS HiFi read alignment dataset of 56x depth and an ONT UL dataset of 47x depth, both sequenced from the same HG002 subject. Minimap2 (Li et.al., 2018) was used as the mapping tool to the GRCh37 reference genome. These alignments were randomly subsetted to produce 10x, 20x, 30x, and 40x input files in addition to the initial full-depth datasets, to assess the effect of depth variance on the calling algorithms. Gold standard SVs were restricted to Tier 1 genomic regions and selected by PASS-only value in the FILTER field, leaving 9284 in total, comprising 4050 deletions and 5234 insertions. Truvari (English et.al., 2022) was used to produce the benchmark metrics, using symbolic alleles only, and the same procedure was applied for the simulated data benchmark. Performance metric calculation is specified as follows:

$$Precision = \frac{TP}{TP+FP} \qquad Recall = \frac{TP}{TP+FN}$$

$$GTAccuracy = \frac{HOM_{TP}^{HOM} + HET_{TP}^{HET}}{HOM_{TP}^{HOM} + HOM_{TP}^{HET} + HET_{TP}^{HET} + HET_{TP}^{HOM}}$$

$$Fscore = 2\frac{Precision \times Recall}{Precision + Recall} \qquad GTFscore = 2\frac{GTAccuracy \times Recall}{GTAccuracy + Recall}$$

Where GT-Accuracy is a metric obtained by estimating the fraction of the correctly genotyped true positive SVs over the total amount of true positives. Thus, superscripts indicate their true genotype, which may differ from the caller classification. GTFscore is a variation from Fscore, to combine correct genotype classification with recall as the harmonic mean between both values.

ACKNOWLEDGEMENTS AND FUNDING

This work has been supported by the "Patrimonio autónomo del Fondo Nacional de Financiamiento para la ciencia, la tecnología y la innovación Francisco José de Caldas" with the contract number 80740-441-2020, awarded by the Colombian Ministry of Science to JD.

We also acknowledge the high-performance computing unit of Universidad de Los Andes for their technical support to conduct the benchmark experiments presented in this manuscript.

DATA AVAILABILITY

The *A.thaliana* TAIR10 reference genome used for simulations is available in the phytozome v.12 database (https://phytozome-next.jgi.doe.gov). The GIAB TIER1 SV gold standard VCF file can be downloaded from the GIAB website (https://www.nist.gov/programs-projects/genome-bottle). The GHC37 human reference genome can be found in the NCBI Assembly database (accession number GCA_000001405.1). PacBio HiFi reads are available at SRA BioProject accession number PRJNA586863. Oxford nanopore reads are located at the European Nucleotide Archive (ENA) under accession PRJEB37264.

SOFTWARE AVAILABILITY

The algorithm presented in this study can be executed through the Single sample Variants Detector functionality of the open-source software Next Generation Sequencing Experience Platform (NGSEP). Releases of NGSEP are available at SourceForge (http://ngsep.sf.net). Life development is available on Git Hub (https://github.com/NGSEP). These are full details of the availability of supporting source code and requirements:

Project name: Next Generation Sequencing Experience Platform (NGSEP)

Project home page: http://ngsep.sf.net

Operating system(s): Platform independent

Programming language: Java

Other requirements: Java 11 or higher

License: GNU GPL

RRID: SCR_012827

Biotools ID: NGSEP

COMPETING INTEREST STATEMENT

The authors declare that there are no competing interests related to the publication of this manuscript.

REFERENCES

Alkan, C., Coe, B. P., & Eichler, E. E. (2011). Genome structural variation discovery and genotyping. *Nature Reviews Genetics*, *12*(5), 363-376.

Alonge, M., Wang, X., Benoit, M., Soyk, S., Pereira, L., Zhang, L., ... & Lippman, Z. B. (2020). Major impacts of widespread structural variation on gene expression and crop improvement in tomato. *Cell*, *182*(1), 145-161. http://doi.org/10.1016/j.cell.2020.05.021

Bolognini, D., Sanders, A., Korbel, J. O., Magi, A., Benes, V., & Rausch, T. (2020). VISOR: a versatile haplotype-aware structural variant simulator for short-and long-read sequencing. *Bioinformatics*, *36*(4), 1267-1269. https://doi.org/10.1093/bioinformatics/btz719

Carvalho, C. M., & Lupski, J. R. (2016). Mechanisms underlying structural variant formation in genomic disorders. *Nature Reviews Genetics*, *17*(4), 224-238. https://doi.org/10.1038/nrg.2015.25

Cericola, F., Lenk, I., Fè, D., Byrne, S., Jensen, C. S., Pedersen, M. G., ... & Janss, L. (2018). Optimized use of low-depth genotyping-by-sequencing for genomic prediction among multi-parental family pools and single plants in perennial ryegrass (Lolium perenne L.). *Frontiers in plant science*, *9*, 369. https://doi.org/10.3389/fpls.2018.00369

Chiang, C., Scott, A. J., Davis, J. R., Tsang, E. K., Li, X., Kim, Y., ... & Hall, I. M. (2017). The impact of structural variation on human gene expression. *Nature genetics*, *49*(5), 692-699. https://doi.org/10.1038/ng.3834

Cleal, K., & Baird, D. (2022). Dysgu: efficient structural variant calling using short or long reads. *Nucleic Acids Research* 50(9): e53. https://doi.org/10.1093/nar/gkac039

English, A. C., Menon, V. K., Gibbs, R., Metcalf, G. A., & Sedlazeck, F. J. (2022). Truvari: Refined structural variant comparison preserves allelic diversity. *bioRxiv*. https://doi.org/10.1101/2022.02.21.481353

Fujimoto, A., Wong, J. H., Yoshii, Y., Akiyama, S., Tanaka, A., Yagi, H., ... & Shimada, M. (2021). Whole-genome sequencing with long reads reveals complex structure and origin of structural variation in human genetic variations and somatic mutations in cancer. *Genome medicine*, *13*(1), 1-15. https://doi.org/10.1186/s13073-021-00883-1

Fumagalli, M. (2013). Assessing the effect of sequencing depth and sample size in population genetics inferences. *PloS one*, *8*(11), e79667. https://doi.org/10.1371/journal.pone.0079667

Gil J, Andrade-Martínez JS and Duitama J (2021) Accurate, Efficient and User-Friendly Mutation Calling and Sample Identification for TILLING Experiments. Front. Genet. 12:624513. doi: 10.3389/fgene.2021.624513

Gorkovskiy, A., & Verstrepen, K. J. (2021). The Role of Structural Variation in Adaptation and Evolution of Yeast and Other Fungi. *Genes*, *12*(5), 699. http://doi.org/10.3390/genes12050699

Heller, D., & Vingron, M. (2019). SVIM: structural variant identification using mapped long reads. *Bioinformatics*, *35*(17), 2907-2915. https://doi.org/10.1093/bioinformatics/btz041

Jiang, T., Liu, S., Cao, S., Liu, Y., Cui, Z., Wang, Y., & Guo, H. (2021). Long-read sequencing settings for efficient structural variation detection based on comprehensive evaluation. *BMC bioinformatics*, *22*(1), 1-17. https://doi.org/10.1186/s12859-021-04422-y

Jiang, T., Liu, Y., Jiang, Y., Li, J., Gao, Y., Cui, Z., ... & Wang, Y. (2020). Long-read-based human genomic structural variation detection with cuteSV. *Genome biology*, *21*(1), 1-24. https://doi.org/10.1186/s13059-020-02107-y

Lamesch, P., Berardini, T. Z., Li, D., Swarbreck, D., Wilks, C., Sasidharan, R., ... & Huala, E. (2012). The Arabidopsis Information Resource (TAIR): improved gene annotation and new tools. *Nucleic acids research*, *40*(D1), D1202-D1210. http://doi.org/10.1093/nar/gkr1090

Li, H. (2018). Minimap2: pairwise alignment for nucleotide sequences. *Bioinformatics*, *34*(18), 3094-3100. https://doi.org/10.1093/bioinformatics/bty191

Luan, M. W., Zhang, X. M., Zhu, Z. B., Chen, Y., & Xie, S. Q. (2020). Evaluating structural variation detection tools for long-read sequencing datasets in saccharomyces cerevisiae. *Frontiers in genetics*, *11*, 159. http://doi.org/10.3389/fgene.2020.00159

Mahmoud, M., Gobet, N., Cruz-Dávalos, D. I., Mounier, N., Dessimoz, C., & Sedlazeck, F. J. (2019). Structural variant calling: the long and the short of it. *Genome biology*, *20*(1), 1-14. https://doi.org/10.1186/s13059-019-1828-7

Parks, M. M., Lawrence, C. E., & Raphael, B. J. (2015). Detecting non-allelic homologous recombination from high-throughput sequencing data. *Genome biology*, *16*(1), 1-19. https://doi.org/10.1186/s13059-015-0633-1

Qiao, X., Li, Q., Yin, H., Qi, K., Li, L., Wang, R., ... & Paterson, A. H. (2019). Gene duplication and evolution in recurring polyploidization–diploidization cycles in plants. *Genome biology*, *20*(1), 1-23. https://doi.org/10.1186/s13059-019-1650-2

Schubert, E., Sander, J., Ester, M., Kriegel, H. P., & Xu, X. (2017). DBSCAN revisited, revisited: why and how you should (still) use DBSCAN. *ACM Transactions on Database Systems (TODS)*, *42*(3), 1-21. https://doi.org/10.1145/3068335

Sarwal, V., Niehus, S., Ayyala, R., Kim, M., Sarkar, A., Chang, S., ... & Mangul, S. (2022). A comprehensive benchmarking of WGS-based deletion structural variant callers. *Briefings in Bioinformatics*, *23*(4), bbac221. http://doi.org/10.1093/bib/bbac221

Schwarz, J. M., Lüpken, R., Seelow, D., & Kehr, B. (2021). Novel sequencing technologies and bioinformatic tools for deciphering the non-coding genome. *Medizinische Genetik*, *33*(2), 133-145. https://doi.org/10.1515/medgen-2021-2072

Sedlazeck, F. J., Rescheneder, P., Smolka, M., Fang, H., Nattestad, M., Von Haeseler, A., & Schatz, M. C. (2018). Accurate detection of complex structural variations using single-molecule sequencing. *Nature methods*, *15*(6), 461-468. https://doi.org/10.1038/s41592-018-0001-7

Thibodeau, M. L., O'Neill, K., Dixon, K., Reisle, C., Mungall, K. L., Krzywinski, M., ... & Jones, S. J. (2020). Improved structural variant interpretation for hereditary cancer susceptibility using long-read sequencing. *Genetics in Medicine*, *22*(11), 1892-1897. https://doi.org/10.1038/s41436-020-0880-8

Wang, X., Gowik, U., Tang, H., Bowers, J. E., Westhoff, P., & Paterson, A. H. (2009). Comparative genomic analysis of C4 photosynthetic pathway evolution in grasses. *Genome biology*, *10*(6), 1-18. https://doi.org/10.1186/gb-2009-10-6-r68

Zook, J. M., Hansen, N. F., Olson, N. D., Chapman, L., Mullikin, J. C., Xiao, C., ... & Salit, M. (2020). A robust benchmark for detection of germline large deletions and insertions. *Nature biotechnology*, *38*(11), 1347-1355. https://doi.org/10.1038/s41587-020-0538-8

FIGURE LEGENDS

Figure 1. Procedures for intra-alignment and inter-alignment recollection of evidence (signatures) for indels, inversions, and duplications from reads aligned to a reference genome. Duplication assignment is performed only after insertion SVs have been called from signatures (SC=Soft Clip, LSC=Longest Soft Clip, ALN=Read alignment, POS=First position in the reference).

Figure 2. DBSCAN algorithm outlined in the context of variant calling applied to an example deletion event. A distance matrix is built from signatures using the euclidean distance of three numerical values: The difference between the first and last reference position and the length of each signature. BFS is used to build clusters based on core points (points with degrees larger than a given threshold) or points reachable from these core points.

Figure 3. Likelihood estimation for each of four possible scenarios for a diploid organism. In each case, the base 10 logarithm of the obtained value is calculated. For case 1, an example of the estimation of the log-likelihood value is shown, from a situation where the SV allele with a length of 85 bp is assumed, and a read alignment contains a call supporting the SV with a length of 90 bp. The HTS factor is a normalization constant based on the sequencing technology and the according error rate.

Figure 4. Simulation benchmarking results. The size of points increases according to depth for fixed values of 20x,30x,45x, and 60x. A. F-score as a function of sequencing depth. B and C precision-recall curves of SV detection for alignments at different depths B. indels and C inversions. The F-score values for the 20x depth dataset are highlighted with a star. For SVIM, a QS filter > 10 was applied given that this provides the best results for the tool, where 0 filter provides very low precision and >20 filters provide low recall. D. Single thread execution time of all callers as a function of the depth of the input alignments.

Figure 5. Performance metrics for PacBio HiFi and ONT data of HG002, using the Tier 1 SV calls of GIAB as gold standard. A, C show precision-recall curves of SV discovery over varying depths for all callers on A) HiFi data, and C) ONT data. B, D show curves comparing genotyping accuracy with recall on B) HiFi data and D) ONT data. The F-score for the 20x

depth subset is highlighted with a star symbol. SVIM and CuteSV had low values in some metrics, thus, some depth points for these tools are not included, but a trajectory line is left to indicate the results trend they followed over the different datasets.

Figure 6. Class diagram of the functionality as a UML integrated into NGSEP. The generic algorithm classes are designed so that they can be used for other problems and applications. The clustering detection classes adapt them for the SV clustering functionality dictated by an interface designed to guide the solution to this problem.

SUPPLEMENTARY FILES

Supplementary file 1. Accuracy and efficiency measures for the benchmark experiments presented in this study.

Supplementary file 2. Parameters used to run the tools included in the benchmark experiments

Figure 1
Click here to access/download;Figure;Figure1_signature_recollection.pdf

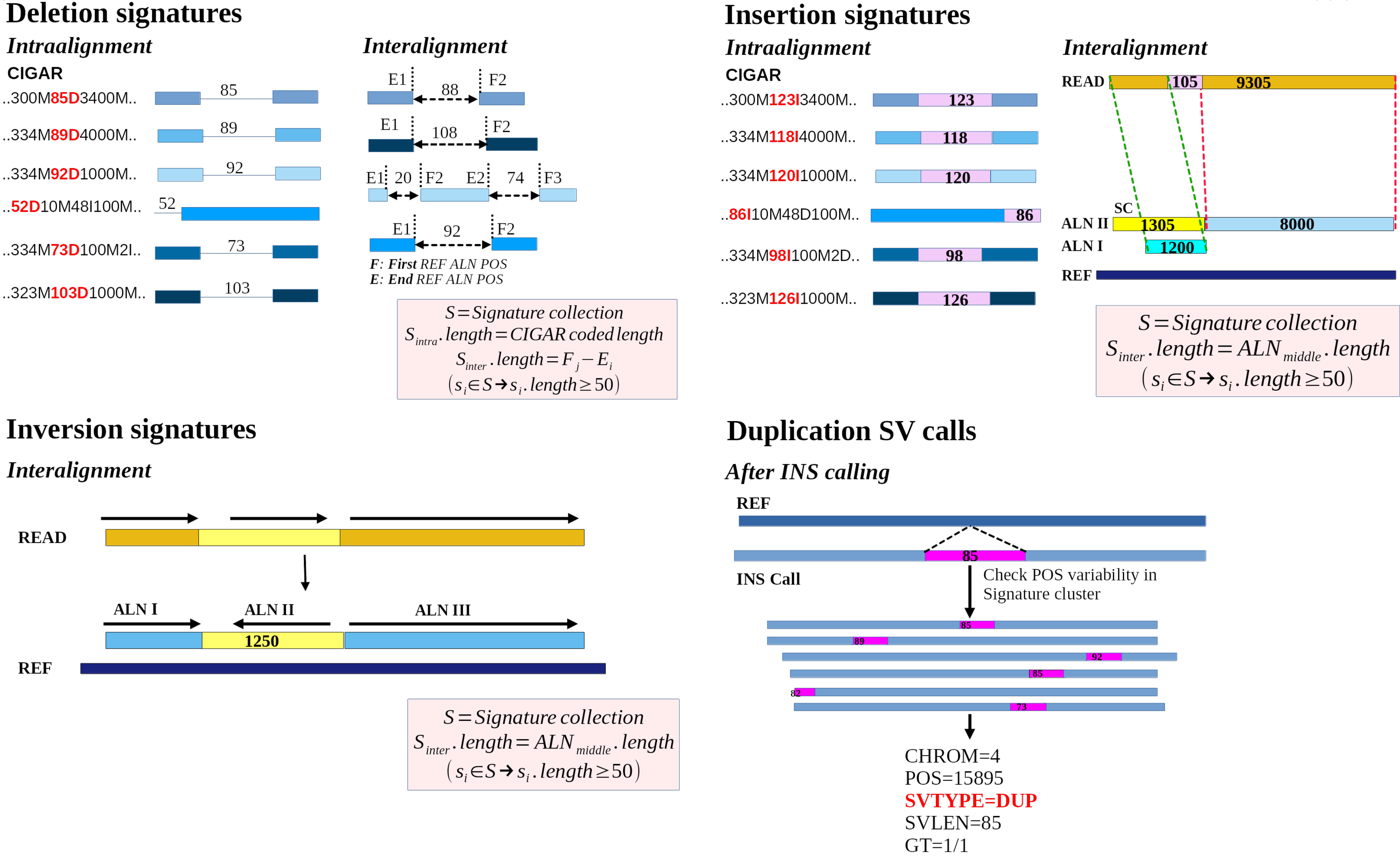

Click here to access/download;Figure;Figure2_DBSCAN_algorithm.pdf

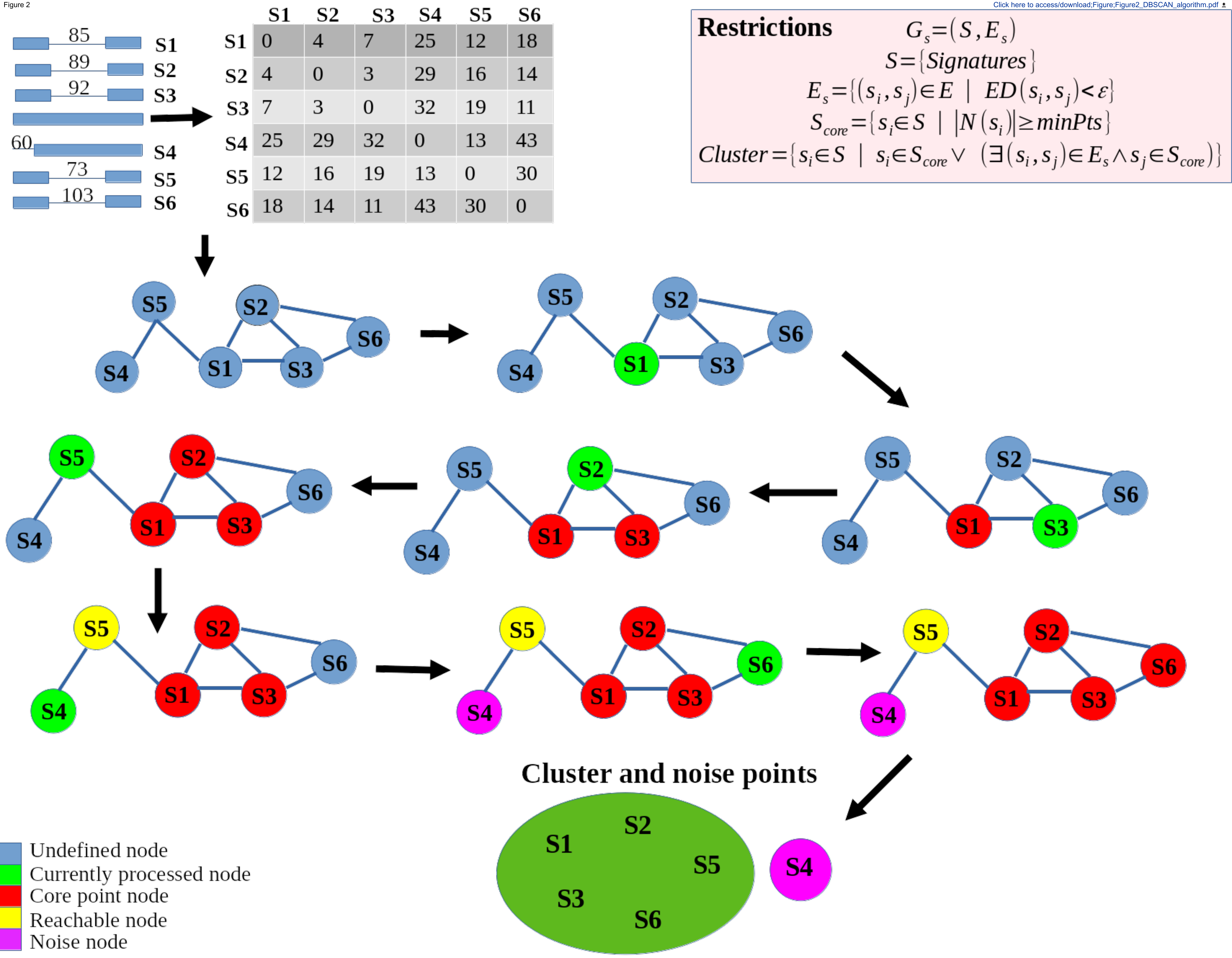

	S1	S2	S3	S4	S5	S6
S1	0	4	7	25	12	18
S2	4	0	3	29	16	14
S3	7	3	0	32	19	11
S4	25	29	32	0	13	43
S5	12	16	19	13	0	30
S6	18	14	11	43	30	0

Restrictions

$$G_s = (S, E_s)$$
$$S = \{Signatures\}$$
$$E_s = \{(s_i, s_j) \in E \mid ED(s_i, s_j) < \varepsilon\}$$
$$S_{core} = \{s_i \in S \mid |N(s_i)| \geq minPts\}$$
$$Cluster = \{s_i \in S \mid s_i \in S_{core} \vee (\exists (s_i, s_j) \in E_s \wedge s_j \in S_{core})\}$$

Figure 3

Click here to access/download;Figure;Figure3_likelihood_estimation.pdf

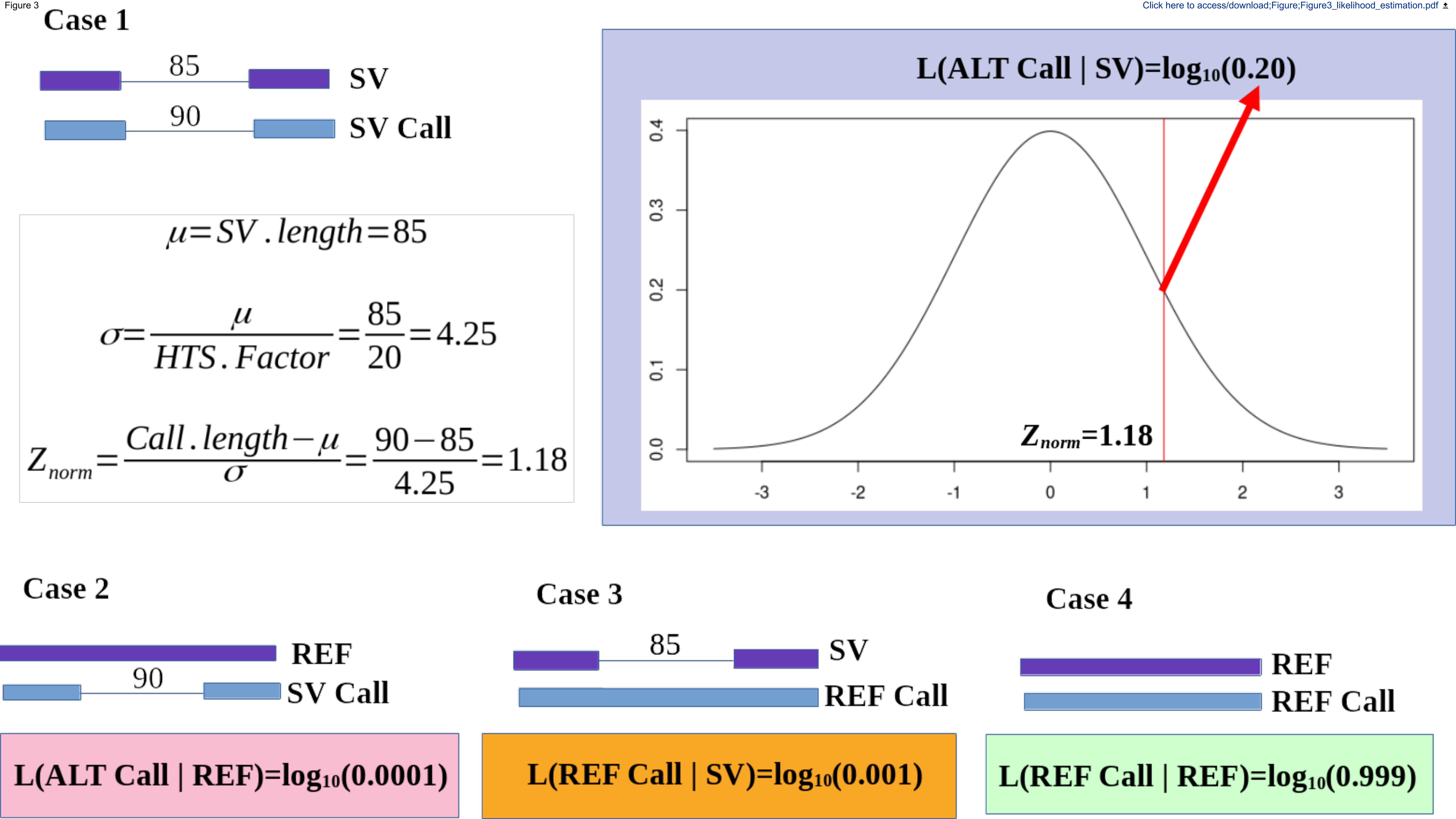

Figure 4

Click here to access/download;Figure;Figure4_SIM_benchmarking.pdf

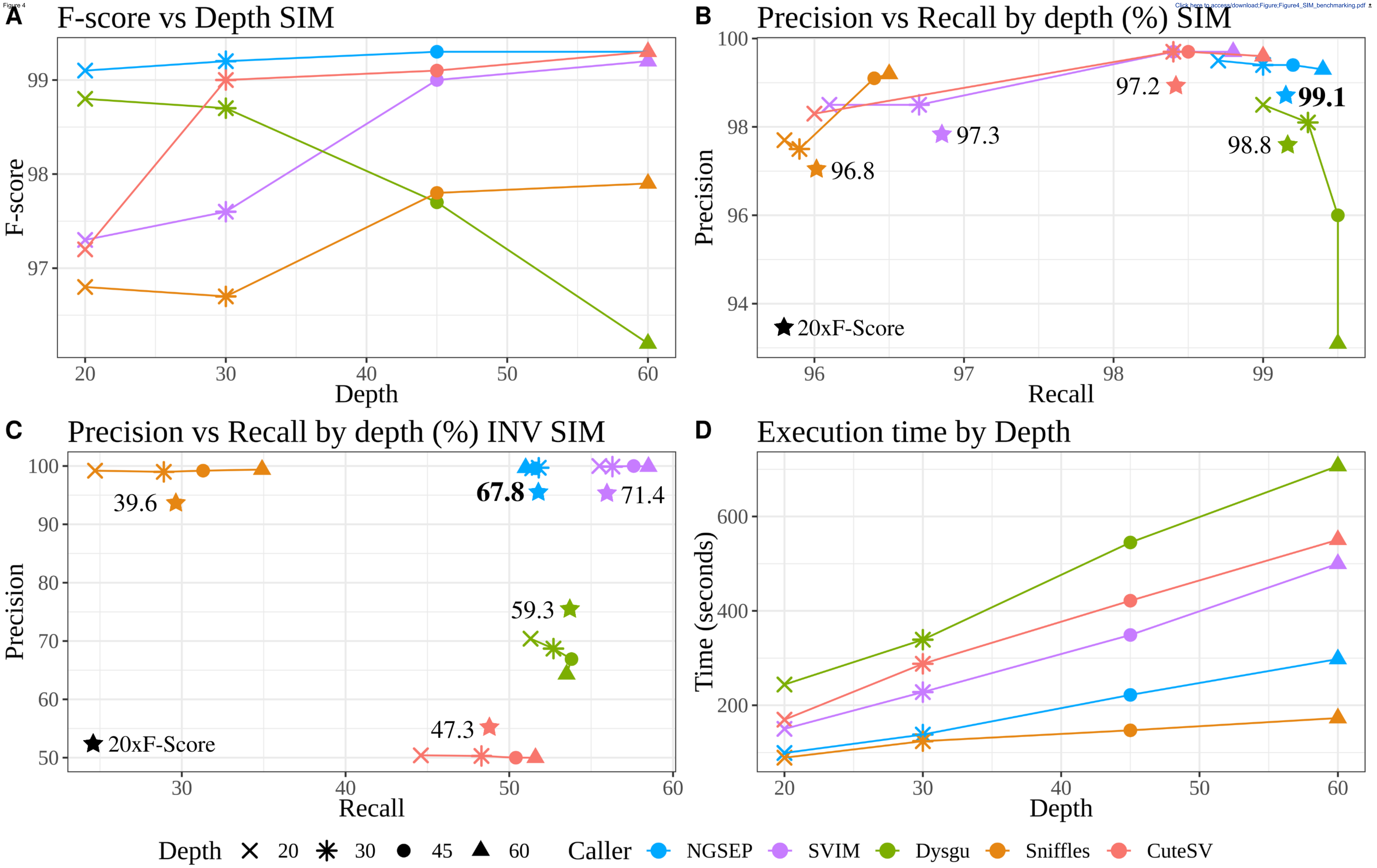

Figure 5
Click here to access/download;Figure;Figure5_GIAB_benchmarking.pdf

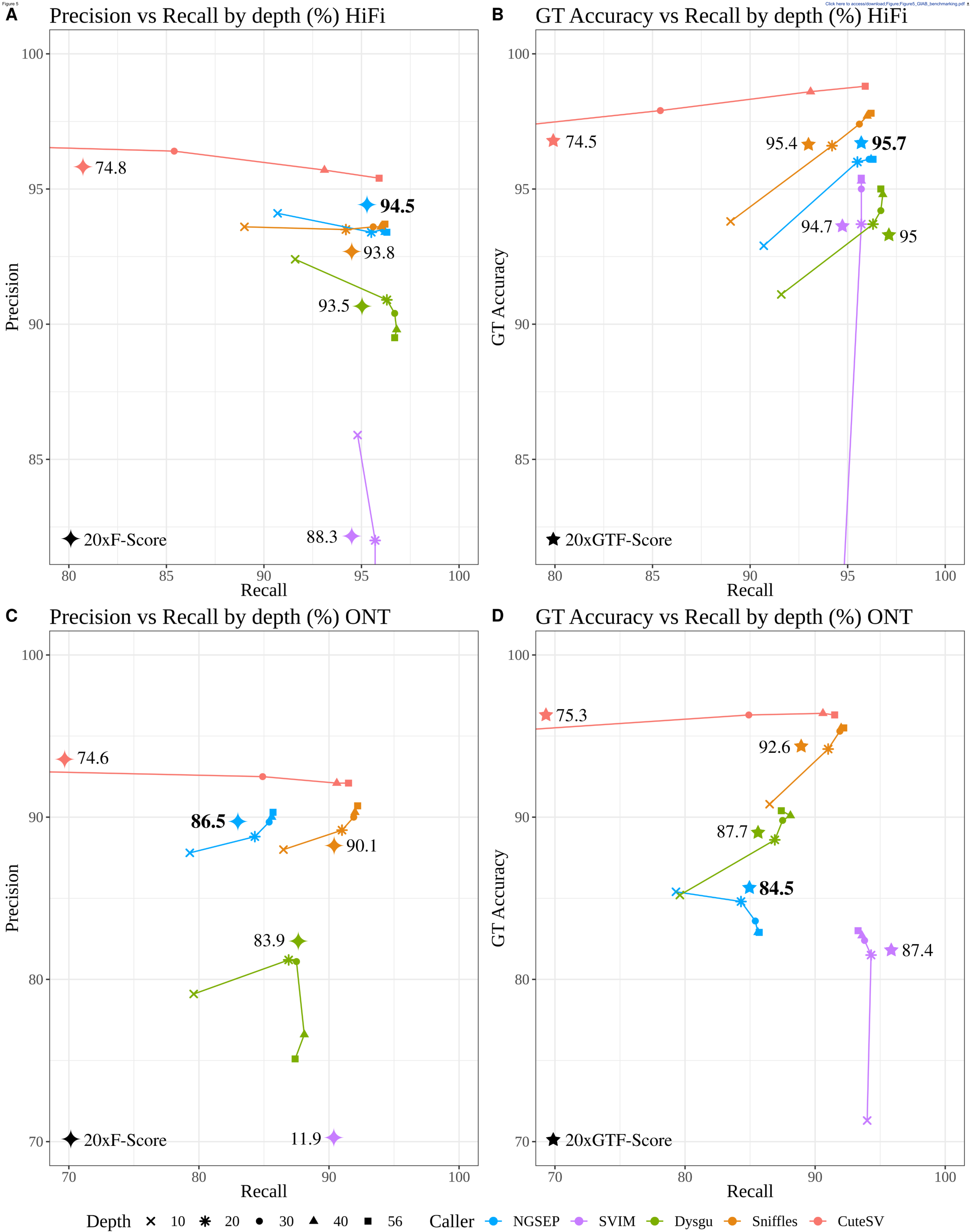

Figure 6
Click here to access/download;Figure;Figure6_NGSEP SV CALLER UML PAPER.pdf

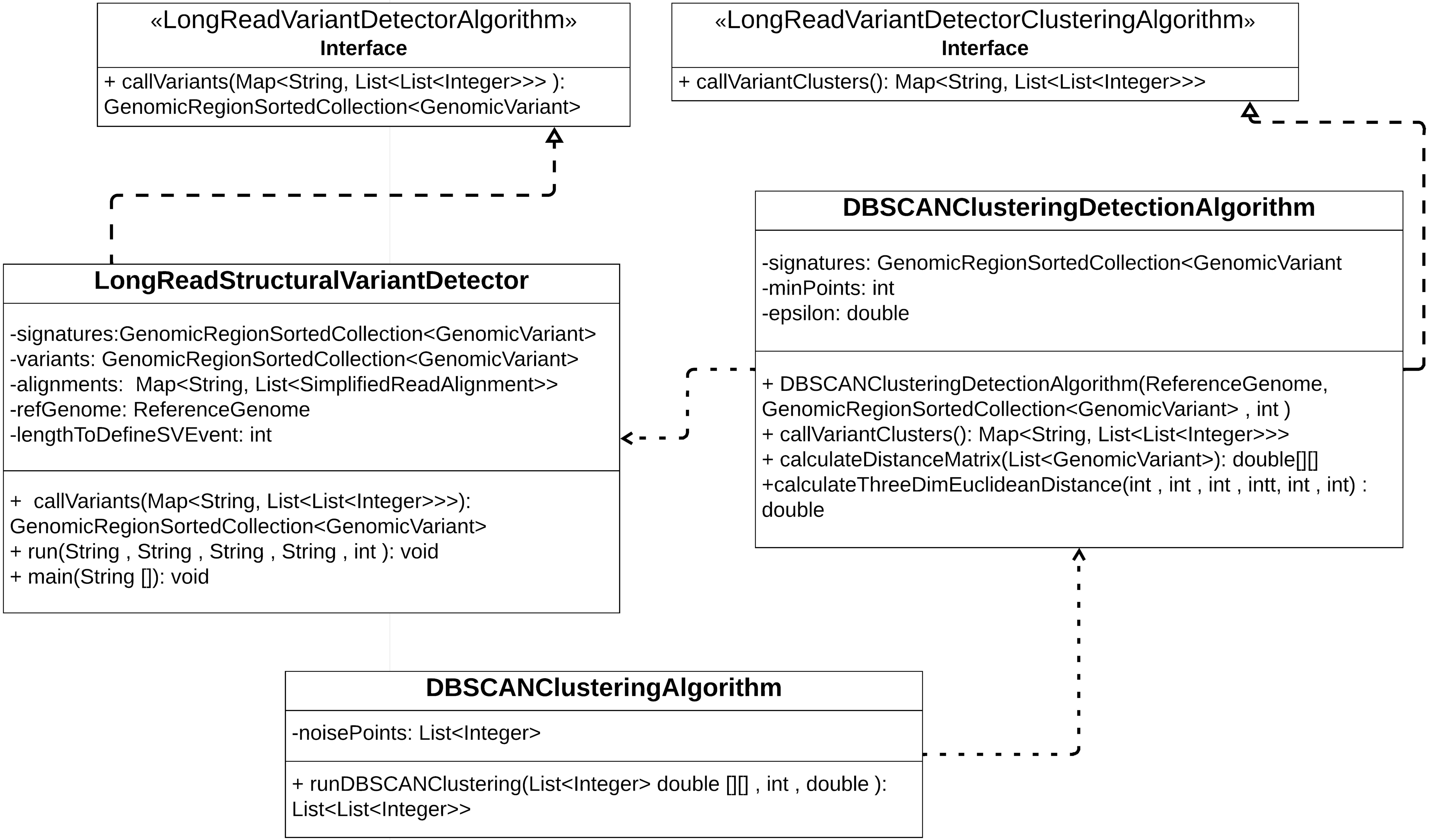

Supplementary file 1

Click here to access/download
Supplementary Material
SupplementaryFile1 .xlsx

Click here to access/download
Supplementary Material
SupplementaryFile2.docx

Personal Cover
Click here to access/download;Personal Cover;NGSEPSVsLongReads_GigaScience_coverLetter_signed

Universidad de los Andes
Colombia
Departamento de Ingeniería de Sistemas y Computación

ABET Engineering Accreditation Commission

Dr. Laurie Goodman
Publishing director
GigaScience

March 20th, 2023

RE: A graph clustering algorithm for detection and genotyping of structural variants from long reads

Dear publishing director

We wish to put in your consideration the manuscript 'A graph clustering algorithm for detection and genotyping of structural variants from long reads' by Gaitan and Duitama for publication in GigaScience.

This manuscript describes a new algorithm for analysis of long DNA sequencing reads aligned to a reference genome to identify and genotype structural variants (SVs), namely, long insertions, deletions and inversions. We integrated the geometric graph based algorithm DBScan to handle the central step of clustering of SV signatures. Benchmarking experiments with state-of-the-art software indicate that our algorithm has superior accuracy among SV types and that it is robust to changes in average read depth and error profiles. This algorithm is implemented within the open source software product Next Generation sequencing Experience Platform (NGSEP).

We believe that our results will be very interesting for a wide range of researchers and practitioners working in human genetics, as well as other species. NGSEP is a well established open source software solution with more than eight years of development and has been extensively used by different research groups working on population genomics of a wide range of species (See https://sourceforge.net/p/ngsep/wiki/Home/ for details). We believe that sequencing of long reads will be a common practice within the next years. In particular, it is likely to replace sequencing of short reads for applications such as population genomics and patient genomic profiling. The reason for this is that long reads provide the information needed to discover and genotype most structural variation that can not be confidently identified using short reads. Hence, accurate algorithms to identify and genotype SVs from long reads will become critical building blocks in upcoming bioinformatic analysis pipelines to support projects requiring high thorughput DNA sequencing.

For these reasons, we believe that this manuscript will have a strong impact in a wide range of research fields and will make a strong contribution to GigaScience.

The manuscript has been seen and approved by all authors.

A previous version of this work was presented this year as a talk in the Colombian Congress of Bioinformatics and Computational Biology (CCBCOL VI), and, as a poster, at the European Conference of Computational Biology (ECCB). A preprint of a previous version of the manuscript is available on bioRxiv (http://doi.org/10.1101/2022.11.04.515241).

We suggest the following referees:

Jay Shendure
University of Washington
shendure@uw.edu

Karen Miga
University of California, Santa cruz
khmiga@ucsc.edu

Mark Blaxter
Sanger Institute
mb35@sanger.ac.uk

Can Alkan
Bilkent University, Ankara, Turkey
calkan@cs.bilkent.edu.tr

We hope that you can take this manuscript in consideration for publication and are looking forward to your assessment.

Yours sincerely

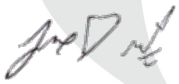

Jorge Duitama Ph.D.
Associate professor
Systems and computing engineering department
Universidad de los Andes
Bogotá, Colombia
Tel: (+57) (1) 3394949 Ext 1686.
E-mail: ja.duitama@uniandes.edu.co

Universidad de los Andes | Vigilada Mineducación | Reconocimiento como Universidad: Decreto 1297 del 30 de mayo de 1964
Reconocimiento Personería Jurídica: Resolución 28 del 23 de febrero de 1949 Minjusticia.
