# Supplementary material for: A graph clustering algorithm for detection and genotyping of structural variants from long reads
Source: Gigascience. 2024 Jan 11;13:giad112. doi: 10.1093/gigascience/giad112 (PMC10783151; doi:10.1093/gigascience/giad112)
Supplement: giad112_GIGA-D-23-00070_Revision_1 [file giad112_giga-d-23-00070_revision_1.pdf]

GigaScience

A graph clustering algorithm for detection and genotyping of structural variants from long reads

--Manuscript Draft--

**Manuscript Number:**	GIGA-D-23-00070R1
**Full Title:**	A graph clustering algorithm for detection and genotyping of structural variants from long reads
**Article Type:**	Technical Note
**Funding Information:**	Ministerio de Ciencia, tecnología e innovación de Colombia (80740-441-2020) — Dr. Jorge Duitama
**Abstract:**	Structural variants (SV) are genomic polymorphisms defined by their length (>50 bp). The usual types of SVs are deletions, insertions, translocations, inversions, and copy number variants. SV detection and genotyping is fundamental given the role of SVs in phenomena such as phenotypic variation and evolutionary events. Thus, methods to identify SVs using long-read sequencing data have been recently developed. We present an accurate and efficient algorithm to predict germline SVs from long-read sequencing data. The algorithm starts collecting evidence (Signatures) of SVs from read alignments. Then, signatures are clustered based on a Euclidean graph with coordinates calculated from lengths and genomic positions. Clustering is performed by the DBSCAN algorithm, which provides the advantage of delimiting clusters with high resolution. Clusters are transformed into SVs and a Bayesian model allows to precisely genotype SVs based on their supporting evidence. This algorithm is integrated into the single sample variants detector of the Next Generation Sequencing Experience Platform (NGSEP), which facilitates the integration with other functionalities for genomics analysis. We performed multiple benchmark experiments including simulation, and real data, representing different genome profiles, sequencing technologies (PacBio HiFi, ONT), and read-depths. The results show that our approach outperformed state-of-the-art tools on germline SV calling and genotyping especially at low depths, and in error-prone repetitive regions. We believe this work significantly contributes to the development of bioinformatic strategies to maximize the use of long-read sequencing technologies.
**Corresponding Author:**	Jorge Duitama<br><br>COLOMBIA
**Corresponding Author Secondary Information:**	
**Corresponding Author's Institution:**	
**Corresponding Author's Secondary Institution:**	
**First Author:**	Nicolás Gaitán
**First Author Secondary Information:**	
**Order of Authors:**	Nicolás Gaitán<br>Jorge Duitama
**Order of Authors Secondary Information:**	
**Response to Reviewers:**	Please see the complete answer with supporting figures in the personal cover letter file. Here we transcribe the answers to the comments of the reviewers.<br><br>Reviewer #1: The submitted study presents an innovative new software for calling de novo structural variants using long-read data. The novelty of the work lies in the application of the DBSCAN clustering algorithm to the problem of identifying SVs from a collection of SV signatures. The paper is well written an easy to follow, and the authors present some good performance metrics for their new software. However, I will

Powered by Editorial Manager® and ProduXion Manager® from Aries Systems Corporation

highlight some short comings with the manuscript in its current form:

R. We thank the reviewer for the evaluation of our work. We are glad to hear that the main innovation of our work was properly communicated. We performed new benchmark experiments and modified significant parts of the manuscript to properly address the comments of the reviewer. Please find below our answers to each specific comment.

Major points:

The results are currently not reproducible due to missing information, and perhaps missing scripts. In the supplementary, the command used to run NGSEP is not given. I was able to repeat a run on PacBio HiFi data (HG002 sample, 8X coverage) using the following command, and will use the results of this run to raise some further points:

'/usr/bin/time java -jar NGSEPcore_4.3.1.jar SingleSampleVariantsDetector -runOnlySVs -i HG002.pacbio.cram -r ucsc.hg19.fasta -o HG002.ngsep -runLongReadSVs'

The output of this command was a gff file with 17277 SVs which is in line with the Supplementary Data file NGSEP_SVCaller_output_PBHifi_10x.vcf which contained around 20800 SVs using 10X coverage. However, the program only produced a gff file rather than a vcf file, so I was not able to analyze results further. Please can the authors state how to use the software to produce a vcf output and include any further scripts necessary for this.

R. We apologize for this issue with the output format. We also thank the reviewer for taking the time to test the software. On the positive side, the command line tried by the reviewer is accurate, according to the current manual. We included the SVs in the VCF file if SNVs were also called, but unfortunately we were not generating a VCF if SNVs were not called. We fixed this usability issue and, moreover, version 4.3.2 now produces a separate VCF for structural variants identified from long reads. We added to the Supplementary material the command line to run NGSEP and the procedure to replicate the benchmarking using Truvari

The runtime and memory usage of the above command was higher than expected compared to the presented benchmark on the simulated reads (gnu-time command was used to test). The test command appeared to use several threads during execution and finished in 24mins 25s and used 16.7 Gb of memory. The input cram file was only 8.9 Gb in size, so the memory usage may cause issues on some systems. The manuscript would be improved by running the benchmark on the real datasets (PacBio and ONT), and reporting memory usage alongside time.

R. We performed further testing experiments and we believe that both the usage of more than one core and the reports of high memory peaks are more related to the behavior of the Java Virtual machine, which is able to use resources that it sees idle. We have been able to run the functionality on 60x human data using up to 16 Gb of RAM in a laptop having only 4 processors by limiting the available memory using the java option -Xmx. We included in the manuscript the use of memory and time for the GIAB benchmark (see supplementary figure 8). Additionaly, we included the java option in the description of the command line.

The manuscript would be improved by presenting data on the Tier2 HG002 GIAB benchmark dateset, not just Tier 1 regions. Although Tier1 regions are the highest accuracy regions, these also present an 'easy case' for long read callers, as demonstrated by the results - there are relatively small differences among callers in these regions. A key use-case of long-reads is to analyse more difficult genomic regions; therefore, it would be of interest to readers to assess Tier1+2 regions. A quick comparison of NGSEP, dysgu, svim and sniffles indicated large differences in the total number of deletion SVs >=50 bp called and most of these differences probably arise outside of Tier1 regions.

R. We actually had comparisons including tier1+tier2 in an earlier version of the work but we used only tier1 based on a suggestion of a previous reviewer. We finally

Powered by Editorial Manager® and ProduXion Manager® from Aries Systems Corporation

decided to report both results. The new results are shown in Figure 5 and the new supplementary figures 2 to 6.

The total numbers of SVs called per genome should be presented and discussed. The numbers of called SV that intersect the Tier1+2 and regions should be shown.

R. Following the suggestion, we now refer in the methods section to the supplementary table 1, which contains a thorough description of the number of SV types included in all the datasets used for benchmarking.

Minor points:

The software versions of tools are not given.

R. We improved the results to show the exact version of each tool included in the benchmark experiments, given that it was only specified in the methods section.

Please could the authors also include details of the computer system used for benchmarking.

R. We added a methods section to describe the execution environment used for each experiment.

The software does not report translocations/BND, in contrast to the comparison tools, please could a sentence be included to indicate this.

R. We clarified in the discussion that at this stage the software does not call translocations

The use of a star in the plots to indicate an F1 score is confusing to the reader as it looks like the star is an extra data point rather than an annotation. The stars would be easier to read if they were moved to a legend.

R. We followed the suggestion and moved the F1 scores to the legend

CuteSV appeared to show unexpectedly poor performance at low coverage values, could the authors comment on why this was the case in their experiments.

R. We took a close look at the manuscript to understand the method implemented in CuteSV. The clustering algorithm is relatively simple. They first create clusters based on distance between genomic coordinates, and then they create subclusters based on length differences. The process to define clusters and subclusters is based on simple decision rules tuned by fixed parameters for each event type (which can be changed by user options), such as the distance between signatures, the absolute number of reads needed to support a variant, and the percentage of the average event length within a cluster after which a new subcluster is formed. Given that a large space of parameter options are needed to be tuned for each experiment, we only ran the experiments with default values. In particular, the default value of the “--min_support” parameter (10) is likely to explain the outcome that we obtained. After this analysis, we believe that our method adapts more naturally to different event types and sequencing technologies because we did not have to tune parameters for each particular experiment. We improved the discussion adding a brief summary of this rationale.

SVIM showed very poor precision on nanopore data, could the authors comment on this finding - previous studies suggest SVIM performance suffers on ONT data due to a high number of duplicated true-positive SVs (Cleal et al., 2021).

R. In our experience with SVIM, we observed that they changed their clustering algorithm from a Maximal Clique finder to a hierarchical clustering process. This algorithm produces flat clusters that may fail to resolve close calls. It seems this leads to duplicated SV calls from individual or few nearby signatures, as evidenced by the ratio of duplicate true-positive calls to the number of true-positive calls in Cleal et.al (2022). Additionally, discordant signals may be grouped, and called as a single SV, as

Powered by Editorial Manager® and ProduXion Manager® from Aries Systems Corporation

the example attached below where only SVIM calls the insertion in the picture from the 10x GIAB benchmark. This is consistent with our results. Thus, we decided to filter their output for comparison as explained in the supplementary table 2 to improve their precision. Further filtering by quality score leads to significant decreases in recall.

The manuscript would be improved by including a lower coverage test on real data, e.g. 5X coverage, as many researchers are interested in utilizing lower coverages.

R. We included the results at 5x in the new simulation of a human individual and in the results with HG002.

Reviewer #2: The authors of the manuscript "A graph clustering algorithm for detection and genotyping of structural variants from long reads" introduce a new method for long read based SV analysis integrated in their NGSEP framework. The main advancement of what they are presenting is the clustering and genotyping methodology, which they show it improves SV detection based on simulated and GIAB benchmark data. The manuscript is overall easy to understand and to follow the points made by the authors. Nevertheless, I feel like I am missing many details on why the algorithm should perform better than existing methodologies that also uses similar concepts on genotyping. In the following I list my questions and concerns in no particular order:

R. We thank the reviewer for the evaluation of our work. We are glad to hear that the text was easy to follow. We performed changes to the methods, results and discussion to address the comments of the reviewer. Please find below our specific answer to each comment.

1. There are overall not many details given that helps me to understand why your algorithm should perform better than the others. This is a bit tricky for me, because I like to understand this to better understand the results that you are showing. For example, you give great detail about why you cluster but then you are not giving much on concrete examples where this clustering matters.

R. We improved the discussion to provide a rationale on why the algorithm could work better than other solutions. Specific reasons for the observed differences against SVIM and CuteSV are described in the answers to the minor comments of reviewer 1. Below we provide examples in which our clustering algorithm provides improved analysis of candidate SV signatures.

2. Their approach of clustering might be novel, but I fail to understand the differences this makes from traditional clustering approaches. Like it is often hard to cluster signals accurately as SV often occur in repeat regions and thus over or under merging can happen. It would be great if you could show some examples of this. It would be interesting to see (since it is one of the main novelties) how it behaves under different circumstances. Like some methods take the SV size into account when clustering the reads to the individual breakpoint. Is that also the case here?

R. The event length is included as one of the dimensions for DBSCAN. Signature false merging is in fact one of the main challenges, especially in repeat regions. We include in the cover letter three examples from the 10x GIAB benchmark in which our algorithm performed a better signal clustering, compared to other approaches.

3. I am also failing to fully understand how an improved clustering can improve the precision of the method, but maybe this comes from genotyping? It would be insightful if the authors give more details on how they filter their SV candidates.

R. You are right on your assessment that both phases are important for our algorithm to achieve high precision values. In the clustering phase, the DBSCAN algorithm allows for a better differentiation of clusters compared to other algorithms because it is capable of producing different cluster geometries. Even apparently similar points may border two different clusters. In the SV calling context for example, we can precisely call SVs that are adjacent in the genome but vary in length, avoiding false-merging events and a false positive call. In the genotyping phase, due to the power of our Bayesian model, if the posterior hypothesis results in a reference homozygote, we can filter that SV call from the output. We improved the results to describe this process

Powered by Editorial Manager® and ProduXion Manager® from Aries Systems Corporation

more clearly.

4. It was interesting to me that the authors identify deletions, insertions and inversions, but didn't call duplications or translocations. Is there any reason for this?

R. We improved the results section to better explain that duplications are called after analyzing the variability of starting positions for insertion calls. We decided to not include translocations at this stage because signatures related to these events are more difficult to identify and to cluster, compared to other SV types. Also, we could not find gold standard datasets from real data to assess the reliability of translocation calls.

5. For the simulated data set, how did you evaluate the data set?

R. We improved the methods to explain better the simulation procedures, the number of simulated events and the evaluation procedure. Because the data is simulated, we have a perfect gold standard of structural variants that should be called, and hence we can calculate precision and recall.

6. For the GIAB I saw that you are using custom parameters for Truvari? I see the -p 0.0, which I think refers to the sequence identify comparison. What I am more wondering is the -r 1000. Could you justify this?

R. The -r 1000 parameter allows Truvari to compare SVs that are located at maximum 1000bp between each other, in terms of reference coordinates. This is specially important for bigger SVs, given that breakpoints are not consistently detected between callers. Using the default -r 500 could lead to underestimating the recall of some tools that may miss on the coordinates of the variant but are detecting it nonetheless.

7. I would have liked to test this SV caller myself but it seems that this is not so easily possible since I have to install the entire software analysis suit.. or is there a way around this?

R. To run NGSEP from the command line you just need to have the general purpose java virtual machine (v11 or superior) installed in your computer. You can either install the versions from Oracle or the openJDK in the case of linux. After that, you just need the jar file of NGSEP (currently version 4.3.2), which you can download from our website (https://sourceforge.net/projects/ngsep/files/Library/). Once downloaded, if you go to the download folder in the command line and type:

java -jar NGSEPcore_4.3.2.jar

You will see the different functionalities offered by the software. In particular, the functionality described in this manuscript can be executed typing:

java -jar NGSEPcore_4.3.2.jar SingleSampleVariantsDetector

Finally, the command we used for our experiments has been included in the Supplementary table 2.

8. I am missing also some details on the simulation. How many SV were simulated per data set, whats the sizes of SV simulated etc. I see some detail in the methods, but it only says indel were simulated and not inversion?

R. We improved the methods to better explain the simulation procedures, the number of simulated events and the evaluation procedure. We included the number of inversions in the text. You can find the exact breakdown by type of number of SVs for each gold standard dataset in the Supplementary Table 1.

Reviewer #3: The paper is well written and the results are good.
The methods described here remind me of those from Jasmine and Iris (Kirsche 2023), and I would like to authors to help me understand the differences between the two clustering approaches.

R. Thanks for your assessment of the manuscript. We went over the work of Kirsche et

al., 2023 and we found that Jasmine solves a relatively different problem, namely the merging of individual SV calls. We are glad to see that they follow an alternative similar to that implemented in NGSEP. Going over the details of the algorithm, the Jasmine algorithm represents SVs as points in a two dimensional Euclidean space consisting of the first reference coordinate and length of the variants. Then, they produce a graph with the SVs as vertices by adding distance weighted-edges between them, complying with different restrictions. In our approach, we also include the end coordinate of each event and hence our graph represents signatures into a 3D Euclidean space, restricting the complete graph based on a distance threshold. The major divergence comes when Jasmine produces a Minimal Spanning Forest, to merge SVs into a single call if they are found in the same tree. In contrast, we use the DBSCAN algorithm to cluster the signatures into an individual SV call, processing the graph through a Breadth First Search. We included the paper in the references and made an appropriate citation in the discussion.

Also, it would be helpful to break up the results by SV type to see if the improvements seen are general or specific.

R. We improved the results to show clearly the behavior of the method separately for insertions and deletions in the human GIAB benchmark experiments (Supplementary figures 3-6). We only could assess inversions in the simulations with the Arabidopsis genome because the human gold standard datasets do not include inversions. The results for inversions are separated from those of insertions and deletions in figure 4.

Reviewer #4: In this manuscript named "A graph clustering algorithm for detection and genotyping of structural variants from long reads", Gaitán et al represented an algorithm that detect genomic structural variants (SV) from long reads. Albeit the existence of multiple long-read based SV discovery methods, I can see the value of this method to the field of genomic SV and long-read sequencing. However, the benchmarking data and methods described in this manuscript are outdated, and significant revision should be applied before this manuscript can qualify for publication. Below are my specific comments:

R. We thank the reviewer for the assessment of our work. We are glad to hear that the work is perceived as valuable by the reviewer. We performed further benchmark experiments and improved the results and discussion to address each comment of the reviewer. Please find below our specific answer to each comment.

1. Benchmarking methods: the authors included SVIM, Sniffles, CuteSV and Dysgu as benchmarking methods. However, there are newer and better algorithms available, including Sniffles2 (which has significant improvements over Sniffles) and PBSV. In addition, assembly based long-read SV methods, such as PAV, could also be considered for benchmarking.

R. We mentioned the exact version numbers for the different tools in the results section. Although we cited the manuscript of Sniffles1, the results shown in the initial review were generated with the 2.0.6 version of Sniffles. In any case, we double checked that the benchmark was performed using the latest version of each tool. Regarding PBSV, we did not include it in the initial benchmark because we could not find a publication related to this tool. We tried to include this tool directly, but unfortunately it could not run with the minimap alignment files. Thus, we had to realign the reads of the original alignment files with the pbmm2 minimap2 wrapper. Since this would represent a different dataset for benchmarking, we ran all tools on these alignments. Please see the supplementary figure 7 for details. Overall, PBSV ranked below CuteSV and NGSEP,on precision, recall and GT accuracy.
Regarding comparisons with a de-novo assembly and contig mapping approach, we believe that results of de-novo assembly comparisons are generally better than those of read alignment based SV callers. However, they are more expensive given that larger depths are required to achieve a high quality de-novo genome assembly, in comparison to low-depth long-read sampling. Additionally, the two gold-standard datasets we used for real data benchmarking include calls from assembly based methods (especially HGSVC). In the second version, this consortium improved their callset by using the PAV algorithm. Hence, we believe that benchmarking against de-novo assembly methods will not make a fair comparison in these cases.

Powered by Editorial Manager® and ProduXion Manager® from Aries Systems Corporation

	2. Simulation data benchmarking: it's useful to learn the performance of simulation data in the genome of Arabidopsis thaliana, but most important to simulate the human genome for benchmarking.<br><br>R. We did not do this before because we had the HG002 data. We performed the suggested simulation based on the T2T genome, and described the results (Supplementary figure 1).<br><br>3. Real data benchmarking: Gaitán et al compared the performance of NGSEP using HG002 base on the reference of GRCh37.Hhowever, in most of the current studies, both short-read and long-read data, are aligned against GRCh38 or T2T, and these data are of significantly more interest to the filed. Please either re-align the GIAB data against the newer reference genomes for the benchmarking or use other data such as those generated by the human genome structural variation consortium (HGSVC, Check Chaisson et al. 2019. and Ebert et al. 2021)<br><br>R. Although we agree that the GRCh38 genome, or even the T2T genome would be more interesting to perform these benchmark experiments, the public gold standard of GIAB to perform independent benchmark experiments is currently available only for GRCh37. Nevertheless, following this comment we found the dataset of the HGSVC consortium and we performed further benchmark experiments using this dataset, achieving very good performance. Please see the results in figure 6 and supplementary figures 9-11. We thank the reviewer for leading us to this asset, and allowing us to further improve our benchmark experiments.
**Additional Information:**	
**Question**	**Response**
Are you submitting this manuscript to a special series or article collection?	No
**Experimental design and statistics**<br><br>Full details of the experimental design and statistical methods used should be given in the Methods section, as detailed in our Minimum Standards Reporting Checklist. Information essential to interpreting the data presented should be made available in the figure legends.<br><br>Have you included all the information requested in your manuscript?	Yes
**Resources**<br><br>A description of all resources used, including antibodies, cell lines, animals and software tools, with enough information to allow them to be uniquely identified, should be included in the Methods section. Authors are strongly encouraged to cite Research Resource Identifiers (RRIDs) for antibodies, model	Yes

organisms and tools, where possible.<br><br>Have you included the information requested as detailed in our Minimum Standards Reporting Checklist?	
**Availability of data and materials**<br><br>All datasets and code on which the conclusions of the paper rely must be either included in your submission or deposited in publicly available repositories (where available and ethically appropriate), referencing such data using a unique identifier in the references and in the “Availability of Data and Materials” section of your manuscript.<br><br>Have you have met the above requirement as detailed in our Minimum Standards Reporting Checklist?	Yes

Powered by Editorial Manager® and ProduXion Manager® from Aries Systems Corporation

Manuscript

Click here to access/download;Manuscript;NGSEPSVsLongReads_GigaSci

A graph clustering algorithm for detection and genotyping of structural variants from long reads

Nicolás Gaitán[1], Jorge Duitama[1,*].

[1]Systems and Computing Engineering Department, Universidad de Los Andes, Bogotá, Colombia.

* Corresponding author. E-mail: ja.duitama@uniandes.edu.co

ABSTRACT

Structural variants (SV) are genomic polymorphisms defined by their length (>50 bp). The usual types of SVs are deletions, insertions, translocations, inversions, and copy number variants. SV detection and genotyping is fundamental given the role of SVs in phenomena such as phenotypic variation and evolutionary events. Thus, methods to identify SVs using long-read sequencing data have been recently developed. We present an accurate and efficient algorithm to predict germline SVs from long-read sequencing data. The algorithm starts collecting evidence (Signatures) of SVs from read alignments. Then, signatures are clustered based on a Euclidean graph with coordinates calculated from lengths and genomic positions. Clustering is performed by the DBSCAN algorithm, which provides the advantage of delimiting clusters with high resolution. Clusters are transformed into SVs and a Bayesian model allows to precisely genotype SVs based on their supporting evidence. This algorithm is integrated into the single sample variants detector of the Next Generation Sequencing Experience Platform (NGSEP), which facilitates the integration with other functionalities for genomics analysis. We performed multiple benchmark experiments including simulation, and real data, representing different genome profiles, sequencing technologies (PacBio HiFi, ONT), and read-depths. The results show that our approach outperformed state-of-the-art tools

on germline SV calling and genotyping especially at low depths, and in error-prone repetitive regions. We believe this work significantly contributes to the development of bioinformatic strategies to maximize the use of long-read sequencing technologies.

INTRODUCTION

Structural variants (SV) are a type of genetic polymorphism, in both coding and non-coding sequences, which are usually defined by their length (>50 bp). The main types of SVs are deletions, insertions, translocations, inversions, and copy number variants (Alkan et al., 2011). The main genomic processes that cause the formation of structural variants are DNA recombination, replication, and repair-associated processes (Carvalho et al., 2016). For example, one common mechanism is Non-Allelic Homologous Recombination (NAHR) which is a genetic repair mechanism in which misalignment of previously duplicated regions called low copy repeats (LCR) occurs during meiosis. This subsequently causes a genomic rearrangement event on another locus that does not belong to the LCR gene, thus creating further deletions or duplications (Parks et al., 2015).

The interest in SVs comes mainly from the functional consequences of their genetic diversity. It has been proven that many SVs are involved in different gene expression patterns and influence different characteristics. SVs that are located adjacent to genes may structurally affect *cis*-regulatory regions by position or composition, leading to either silencing or increasing gene expression, which explains variation of Quantitative Trait Loci (QTL)(Chiang et al., 2017). For example, Alonge *et al.* (2020) found that at least 50% of the SVs found in an assessment of around 100 lines of tomato were associated with gene expression regulatory processes, mostly causing reductions or even silencing of gene products. Another case is when duplications increase the amount of overall transcript-protein production by gene dosage effect. This has proven beneficial for artificial selection in certain plant species where the average size

of fruits increased because the plant variant suffered a specific duplication in a cytochrome coding gene (Alonge et al., 2020).

Structural variants also provide fundamental information about evolutionary relationships between organisms and their natural history. Many Whole-Genome Sequencing (WGS) studies have been conducted to assess the prevalence of different SVs and their variation in organisms, populations, or species. In plants, analyzing structural variants allowed elucidation of the dynamics of whole-genome duplication (WGD) events and their evolutionary role (Qiao et al., 2019). WGDs are followed by a fast diploidization process, mainly because most of the duplicated genes become paralogs (Qiao et al., 2019). Furthermore, many components of the C4 metabolic pathway were brought by these WGD events and single duplication events. This is an interesting case of convergence throughout the evolution of different plant lineages (Wang et al., 2009). These changes are influenced by the synergistic effect of WGDs, transposed duplication, and dispersed gene duplication, evidenced by overlapping peaks in the rates of synonymous substitutions (Qiao et al., 2019). This shows how SVs can provide substantial amounts of evidence for evolutionary studies.

Given the importance of SVs, a large number of computational methods have been developed to identify and genotype SVs, based on high throughput sequencing (HTS) data. Most of these SV detection tools are based on short-read sequencing technologies (Cleal et.al., 2022; Sarwal et.al., 2022). This presents many limitations, mostly due to the length of structural variants, which usually exceeds the read length, which reduces the precision of both identification and genotyping (Luan et.al., 2020, Mahmoud et.al., 2019). Recently, new SV calling tools have adopted long reads as their input data, significantly increasing the accuracy of SV detection in comparison with short read-based callers (Mahmoud et.al., 2019; Schwarz et.al., 2021). This has allowed many researchers to increase their catalog of functionally relevant structural variants, including some that affect the pathophysiology of diseases such as human cancer

(Fujimoto et.al., 2021; Thibodeau et.al., 2020). However, further improvements could be achieved by novel algorithmic techniques. Some difficulties arise even when long reads are used. Since SV detection relies on accurate read alignment, dissimilar, partial, or inaccurate read alignments obscure the signal to perform a consistent detection and genotyping of SVs. Thus, the results also depend on the accuracy of the aligner software (Heller et.al., 2019). Additionally, from a software design point of view, our experience indicates that most current tools are difficult to operate because they require a large number of specific libraries and versions, their implementations are not debugged correctly and exceptions are not handled appropriately. For short read-based callers, these limitations have been described by a recent benchmark study by Sarwal et.al (2022).

Benchmarking SV detection is a difficult task. First, there are few independently validated gold standard datasets for real sequencing data because experimental validation is difficult to perform at a large scale. Consequently, there is no consensus on which of the existing tools produces the closest result to a gold standard set. Bolognini et.al (2020) addressed this issue by implementing a simulation software called VISOR, which produces a complete haplotype-resolved sample genome and simulates read alignments from a list of SVs, with either Oxford Nanopore or PacBio error profiles. Trying to optimize the SV calling pipeline, Jiang et.al (2021) evaluated the accuracy of different SV callers using VISOR simulations on real reported human SVs. For the 20x simulated dataset, they report that the best tools are CuteSV (F1=0.8), SVIM (F1=0.798), and Sniffles2 (0.769). Additionally, they provide recommendations for SV calling best practices such as sequencing experiments with read lengths of about 20 kb at 20x depth. Regarding real datasets, the most widely recognized and best-curated case is the high-confidence structural variant dataset (Sample HG002 on reference genome GRCh37) from the Genome In A Bottle human sample project (GIAB) crafted for benchmarking (Zook et.al.,

2020). The events reported in this file come from a mixture of sequencing technologies and have been predicted by using a pipeline integrating many different tools.

The HGSVC consortium also generated high confidence SV calls suitable for benchmarking. In the first version, a haplotype-resolved curated SV callset against the GRCh38 genome was produced for each of three samples from different ethnicities, including Han Chinese, Yoruban Nigerian, and Puerto Rican (HG00514, HG00733, NA19240) respectively. This provides SV variation profiles for individuals with a wide range of genetic diversities, including admixed individuals (Chaisson et.al., 2019). Similar to the GIAB effort, multiple sequencing platforms, and variant calling methods were used to produce these datasets, specially the reference guided assembly of the samples and their parents, which made it possible to determine the haplotype of the SVs. Furthermore, the HGSVC2 version improved these SV calls using *de-novo* assembly with the PAV algorithm (Chaisson et.al., 2019; Ebert et.al., 2021).

Structural variant detection provides the possibility of finding biological insights with many different functional consequences. In this manuscript, we developed a new software solution that improves the detection of germline SVs from long-read alignments using the DBSCAN algorithm to solve the clustering problem, and implements a new bayesian genotyping model. This functionality is integrated into the bioinformatic software suite (NGSEP) to further facilitate the analysis of genomic data.

RESULTS

A new clustering algorithm for detection and genotyping of Structural Variants

The process of structural variant detection and genotyping starts from reads aligned to a reference genome and is divided into three main stages described as follows.

1. Signature Collection

The main input to this algorithm is a set of read alignments in SAM or BAM format, obtained from mapping long reads to a reference genome. Signatures are individual signals of a structural variant that are contained within each read alignment or constructed from discordant partial alignments. They can be divided into intra-alignment and inter-alignment signatures. Intra-alignment signatures consist of evidence of deletions or insertions that are predicted as part of the read alignment process. Thus, these signatures are collected by reading the description of the alignment (encoded in the CIGAR field of the SAM format) to find signals of insertion or deletion. Conversely, reads with multiple discordant alignment segments, regarding their position or orientation, are selected to identify inter-alignment signatures.

Figure 1 shows the procedures that we implemented for the recollection of signatures for each SV type. Intra-alignment deletions and insertions are identified by parsing the CIGAR strings, and searching for their codes (e.g. D or I, respectively), The CIGAR code includes the length of each event within the alignment. Inter-alignment deletions are suspected when unmapped regions in the reference genome are flanked by partial alignments. For each read with two partial alignments within the same chromosome region, the reference distance between the end of the first partial alignment and the beginning of the second alignment in reference genomic coordinates is considered the length of the deletion signature. Inter-alignment insertion signatures are identified from reads with two adjacent alignments, having a soft clip starting from the presumed insertion point. For each read, the Longest Soft Clip (LSC) is calculated by taking the maximum of soft clips at the end of each alignment. The length of the partial alignment that does not contain the LSC is subtracted from the length of the LSC to estimate the length of the insertion signature. Inversions appear as three consecutive partial alignments where the middle alignment has an opposite orientation, compared to the two flanking alignments. The length of the inversion is predicted as the length of the middle alignment.

Signatures are filtered from the minimum SV length specified by the user (default ≥50 bp) and are added to a collection, which is sorted by chromosome and reference coordinates.

2. *Signature Clustering*

Given a set of SV signatures, we implemented a graph-based clustering in which each cluster becomes a candidate SV event. A graph is built independently for each signature type. The vertices of the graph correspond to the input collection of signatures identified in the previous step. Each signature is represented by a tridimensional vector with three numeric values: Start coordinate in the reference genome (B_i) end coordinate in the reference genome (E_i), and signature length (L_i). The cost m_{ij} of the edge between two signatures i and j corresponds to the Euclidean distance of their corresponding vectors:

$$FPD_{ij} = |B_j - B_i| \qquad LPD_{ij} = |E_j - E_i| \qquad LD_{ij} = |L_j - L_i|$$

$$m_{ij} = \sqrt{FPD_{ij}^2 + LPD_{ij}^2 + LD_{ij}^2}$$

The DBSCAN algorithm is a non-supervised clustering procedure for n-dimensional vectors (points) based on the principle of density-based grouping (Schubert et.al., 2017). The parameters of this algorithm are a threshold *epsilon* (ε) which limits the distance for considering two points as neighbors, and a minimum number of neighbors (minPts) that a point should have to be considered a *core point*. The lemma states that considering a cluster that contains certain *core points*, then any point which is density reachable from any of those *core points* (in the graph context, any point that has a path from any *core point*) will be considered as part of the cluster. Any point that is not reachable from any *core point* will be considered a noise signal. The procedure to implement this algorithm was as follows. Starting from an initially complete graph with *n* points, the algorithm eliminates the edges where m_{ij} is bigger than or equal to ε. Then, each point is visited to test if its number of neighbors is at least *minPts*, in which case it is labeled as a core point. Consequently, a new cluster is initialized with the core point and its

direct neighbors, and a Breadth First Search (BFS) is performed by pushing this neighborhood into a queue where each point will also be queried for its neighbors to assess the *core point* property presumption, repeating this process until all of the density reachable points from any core point in the cluster are visited. If there are unvisited points, the procedure continues until all points are visited. Figure 2 shows the main steps and restrictions of this procedure.

3. *Cluster to Genotyped SV*

Each signature cluster identified in the previous step becomes a candidate SV. The last step of the process is the genotyping of these candidates. To identify SV coordinates, the average of the first reference coordinates of the signatures within the cluster is estimated. The last coordinate is calculated likewise. The length is taken as the difference between both the last and first SV coordinates, except for insertions where the average length of the cluster signatures is estimated as the average of the insertion lengths of the signatures. Candidate SVs are stored in a collection sorted by reference coordinates. Then, a Bayesian genotyping process is performed for each candidate SV by reassessing the evidence that read alignments provide. To avoid having to reprocess the alignments file, a collection of compact alignment objects is kept in memory from the first stage, having the minimum possible information needed for this step. For each SV, intersecting read alignments are collected, and those containing clustered signatures are considered supporting evidence for the alternative allele hypothesis. If the spanning read alignment contains no signatures, it is counted as a supporting call for the reference allele. Figure 3 shows the estimation of the likelihood for the four possible scenarios, generated from the combination of the hypotheses, the two plausible alleles from which the read could be sequenced (SV or REF alleles), with calls from a read alignment that may or may not support these allele hypotheses. The distribution of lengths of the clustered signatures supporting the SV hypothesis is used to estimate the likelihood of a read alignment supporting this SV. In this case, it is assumed that the read was actually sequenced from a chromosome

affected by the SV (case 1). If a reference allele is assumed (case 2), a read with an SV signature is proposed to have happened by a misalignment or sequencing error and a fixed value (0.0001 by default) is used as likelihood. The likelihood of a read supporting the reference allele that is assumed to be sequenced from a haplotype affected by the SV is calculated as the probability of having an indel error that reverts the SV and is also a constant value (0.001 by default) (case 3). Finally, a fixed value (default 0.999) is used for the likelihood of a read supporting the reference allele assuming sequencing from a reference haplotype.

Read likelihoods for each allele hypothesis are transformed into posterior probabilities for each possible genotype following the same procedure implemented in NGSEP to perform SNP genotyping (Gil et al., 2021). The hypothesis having the largest posterior probability is assigned as the predicted genotype. If the genotype of an SV call is assigned as homozygous reference (0/0), this call will be considered as not well supported, and it will be filtered out of the output. Similar to SNP genotyping, the quality of such SV calls will be the phred score *Q* corresponding to the genotype posterior probability. Finally, duplications are identified after the three main steps from genotyped insertion SVs, if the supporting intra-alignment signatures differ significantly in reference coordinates.

Benchmarking with simulation experiments

We performed two simulations of structural variants in the genome of *Arabidopsis thaliana* using the tool VISOR (Bolognini et.al., 2020). 1718 insertions, 2532 deletions, and 2065 inversions were generated for benchmark experiments. Read alignment subsets were produced for 20x, 30x, 45x, and 60x. The precision-recall results of our NGSEP algorithm were compared to those of state-of-the-art tools, including SVIM (version 2.0.0) (Heller et.al., 2019), Sniffles2 (version 2.0.6) (Sedlazeck et.al., 2018; Smolka et.al., 2022), CuteSV (version 1.0.13) (Jiang et.al., 2020) and Dysgu (version 1.3.11) (Cleal et.al., 2022). After obtaining the metrics

for both simulation experiments, precision-recall curves and F-score against depth were plotted for each tool. Additionally, execution times for each depth dataset were evaluated for single-thread runs.

Figure 4A shows that the NGSEP algorithm presented above outperforms all of the tools for depths of 20x, 30x, and 45x, according to F-score values (Values available at the Supplementary File 1). Only in the 60x test, CuteSV achieves an equal result. Dysgu suffers from a significant drop in precision, which decreases the F-score for bigger depths. Figure 4B shows that only NGSEP keeps high performance for varying alignment depths in both precision and recall. Although Dysgu outperforms all of the tools in terms of recall for each coverage, it reduces precision below 95% as depth increases. In the inversion simulation benchmark, SVIM produced the highest F-score, closely followed by the NGSEP algorithm (Figure 4C) while most of the other algorithms fail to accurately detect most inversions.

Additionally, we generated a simulated SV gold-standard from the Human T2T genome (Nurk et.al., 2022) following the same pipeline, and increasing the amount of depth samples to 5x, 10x, 20x, 30x, 40x, and 60x including 5,000 insertions and 5,000 deletions, to evaluate the performance of the algorithms with bigger input data sizes and different genome features.

Results from these simulations are similar to those obtained with the Arabidopsis genome (Supplementary File 1). Interestingly, our algorithm generated better recall values in this dataset, and Sniffles2 showed improved precision compared to the other algorithms (Supplementary Figure 1).

Single-thread runtimes were recorded for all experiments to compare the tools in terms of computational efficiency. As shown in Figure 4D, all of them follow a linearly increasing trend. Sniffles2 and NGSEP consistently required lower execution times compared to the other tools. It is worth clarifying that Sniffles2, CuteSV, and Dysgu support multithreading which significantly reduces runtimes at the cost of processing resources. Dysgu was the worst-

performing tool in terms of computational efficiency, requiring about three times more execution time than the NGSEP algorithm. Additionally, Dysgu was the only algorithm that appeared to increase time usage non-linearly from the 40x to the 60x human dataset (Supplementary figure 1).

Benchmarking with the Genome In a Bottle human genome

To assess the performance of our method on real datasets, we performed multiple experiments using reads from the Genome In a Bottle (GIAB) human individual HG002, for which a gold standard set of large indel calls is publicly available. Both 56x PacBio HiFi CCS (Circular Consensus Sequencing), and 47x ONT UL (Ultra Long Reads) reads sequenced from the HG002 subject were randomly sampled at average depths of 10x, 20x, 30x, and 40x to perform different experiments. Truvari (English et.al., 2022) was used to obtain precision and recall metrics of test calls against the gold standard, which was restricted either to the Tier 1 plus Tier 2 (T1+2) regions, or just Tier 1 (T1), and PASS-only SVs. Additionally, a F-score variation called GTF-score was estimated to assess their performance regarding the combination of genotyping accuracy and recall. Further details for this metric are provided in the methods section.

Figure 5A shows the results of the benchmark experiments detecting variants from PacBio HiFi alignments, using as gold-standard the T1+2 dataset, and varying read depth from 5x to 56x. Our algorithm provides the best F-Score for low-depth mappings (5,10 and 20x, see exact values in the Supplementary file 1). Only CuteSV has better precision compared to NGSEP, but this tool has the worst recall below 30x. Similar to the simulation results, at increasing depths Dysgu and SVIM increase recall at a high cost on precision. Regarding GTF-Score, NGSEP produces the highest value for 10x and 20x depth, reaching values of 94% (Figure 5B). At higher read depths, Sniffles2 and CuteSV produce GT accuracy values 2% and 4% higher

than those of NGSEP, respectively. The precision for all tools is low (up to 65%) mainly because the Tier 2 includes highly repetitive regions in the human genome. If the gold standard is restricted to the T1 dataset, all tools improve precision, reaching values over 90% in almost all cases (Supplementary figure 2). Sniffles2 shows the most important increase in this comparison, reaching precision values slightly larger than those of NGSEP for depths above 30x.

Regarding ONT aligned reads and comparing against the T1+2 dataset, NGSEP is the most accurate tool comparing precision and recall. CuteSV and Sniffles2 achieve better recall and genotyping accuracy than NGSEP, at the cost of precision. Restricting the comparison to T1 regions, precision increases for all tools and Sniffles2 becomes the tool with the highest F-Score overall (Supplementary figure 2). The behavior of all callers is relatively consistent with the HiFi data, but the values obtained for the different metrics are consistently lower, probably due to the higher error rate of ONT reads.

A breakdown of these results in both deletion and insertion categories, shows that the major improvements in performance metrics for NGSEP over the other tools comes from the accurate detection of insertions, especially for the ONT data. However, the low GT accuracy of NGSEP for ONT reads is caused by a GT accuracy of insertions below 80% (Supplementary figures 3 and 4). Consistent with the global results, the improved precision of NGSEP is not evident if only T1 regions are included in the benchmark experiments (Supplementary figures 5 and 6).

We also tried to include in the benchmark experiments the tool PBSV (version 2.9.0)(https://github.com/PacificBiosciences/pbsv). However, this tool did not work with the original alignments, and hence we had to realign the HiFi reads with the pbmm2 mapper (available with PBSV). PBSV produced low recall values at low depths, improving as depth increases at a cost on precision. The overall accuracy of PBSV was inferior to that of NGSEP

and CuteSV, both for the complete gold standard dataset and for the subset of Tier 1 SVs (Supplementary Figure 7).

Finally, the runtime of each tool behaves similar to the simulations. Sniffles2 was the fastest tool for all subsets, and PBSV was the slowest tool in most cases (Supplementary Figure 8A). We also analyzed the peak memory consumption for our algorithm. NGSEP takes less than 8Gb of RAM heap space to analyze the datasets up to 30x. For bigger inputs, although more space is used by the Java Virtual Machine, new objects maintain low memory consumption (Supplementary Figure 8B). All experiments could be performed with up to 16 Gb of RAM.

Benchmarking with the HGSVC2 samples

Taking advantage of the efforts made by the HGSVC consortium to produce accurate SV callsets, we included their three most refined samples (HG00514, HG00733, NA19240) into our benchmark experiments. These resulted in a truth set consisting of 74,467 indel SVs. The breakdown per sample and SV type is available in the Supplementary table 1. To evaluate the quality of SV callers for low-depth and varying genetic diversity inputs, we aligned PacBio HiFi sequencing reads from each of these samples to the GRCh38 genome, using minimap2 (Li et.al., 2018). Then, we randomly subsampled the mappings to evaluate the tools at 20x depth.

Figure 6 shows the performance metrics for SV discovery on the three samples. NGSEP achieved the best performance on both F-score and genotyping accuracy (All values are available at the Supplementary file 1). Consistent with the experiments with the GIAB datasets, Dysgu and SVIM reported very low precision values, although they identified more than 80% of the indels. Conversely, CuteSV has high precision and genotyping accuracy, but it could only achieve up to 51% of recall. In this experiment, the calls generated by Sniffles2 had surprisingly low performance metrics, taking into account the performance observed in the

simulations and the GIAB data. After manual inspection of the results, we discovered that Sniffles was reporting SVs in locations consistent with the gold-standards, but the reported SV length was about two times the SV length of the gold standard. Relaxing the reciprocal overlap for test-reference allele lengths (See methods for details), the precision and recall metrics of SVs reported by Sniffles2 improve to values similar to those observed in the previous experiments. However, the GT-accuracy is still affected, increasing only up to 45% (Supplementary Figure 9). Consistent with the experiments with the GIAB dataset, restricting the gold-standards to non-repetitive regions increases the performance of all callers. Both the Fscores and the GTFscores of NGSEP are consistently larger than 90% and the best among tools for all individuals (Supplementary Figures 10 and 11). In particular, our algorithm achieves the best genotyping accuracy for the admixed Puerto Rican individual (NA19240), suggesting that our genotyping procedure is very accurate even for samples with high heterozygosity (Figure 6C, Supplementary Figures 9C,10C and 11C).

DISCUSSION

The availability of long-read sequencing technologies represented a big step forward toward the accurate identification and genotyping of structural variants (Fujimoto et.al., 2021; Thibodeau et.al., 2020). Achieving this goal is becoming a requirement for current genomics, given the documented role of SVs as drivers of phenotyping variability and evolution (Alonge et al., 2020; Gorkovskiy et.al., 2021; Qiao et al., 2019; Wang et al., 2009). In this work we present the results of our efforts to develop novel algorithmic techniques, aiming to increase the accuracy of both discovery and genotyping of germline SVs. Transforming the problem of clustering SV signatures into a geometric clustering problem in an Euclidean space, allowed us to build a solution based on the well-known DBSCAN clustering algorithm (Schubert et.al., 2017) to identify SVs. A similar Euclidean space representation is implemented in the Jasmine

algorithm to merge SVs from different samples into a refined call, which improved population level analyses (Kirsche et.al., 2023). Even though both works differ in the clustering algorithm, they demonstrate the advantages of representing SV signals as n-dimensional euclidean points, and provide the groundwork for future SV analysis algorithms. Previous experiences implementing Bayesian models for SNV genotyping, allowed us to increase the accuracy of SV identification and provided a framework for SV genotyping.

Benchmarking experiments running simulations, and analyzing real data with the GIAB, and HGSVC2 datasets indicate that our algorithm achieves competitive accuracy compared to current software solutions. The observed differences in the results obtained with Tier 1 and Tier 1+2 regions indicate that our solution provides accurate calls in repetitive regions, which remains as one of the main challenges for SV calling efforts. Compared to Dysgu and SVIM, our algorithm provided consistently better accuracy in all experiments. SVIM in particular ranked last in performance for ONT data. This result is consistent with previous experiments (Cleal et al., 2022) and could be explained by the tendency of the hierarchical clustering implemented in SVIM to separate signatures coming from the same variant if there is high variability in alignments due to sequencing error rates. Regarding Sniffles2, this tool was very competitive, achieving in some cases superior discovery and genotyping accuracy, compared to NGSEP, both in the simulations and in the experiments with the GIAB benchmark dataset. However, this behavior was not consistent in our experiments with the HGSVC2 datasets, mainly because in these cases Sniffles2 produced calls with about two times the length of the real calls. We could not identify a rationale for this behavior. Finally, we obtained SV calls with good accuracy running CuteSV, but only if the read depth was superior to 30x. CuteSV implements a two step clustering procedure, making initial clusters based on coordinates and then identifying subclusters based on differences in event length. This process is controlled by a set of parameters which need to be tuned for different event types and sequencing

technologies. Although we acknowledge that further testing with different parameters could yield improved outcomes running CuteSV, we argue that this indicates that our method adapts more naturally to changes in read depths and sequencing technologies, reducing the effort to perform parameter tuning for each experiment.

Given that even using long reads it is not easy to identify and cluster signatures for translocations, compared to other SV types, our current solution does not support discovery of translocations. We expect to implement this feature in future versions of NGSEP. We also plan to further improve on genotyping accuracy in future versions of the algorithm.

Researchers performing population genomic studies usually trade read depth by the number of samples sequenced, looking for a balance that maximizes the cost-benefit of the sequencing effort (Cericola et.al., 2018; Fumagalli et.al., 2013). Thus, it is extremely important for SV detection tools to be able to produce accurate results from a low-depth input. One of the biggest advantages of the NGSEP algorithm, when compared to the other state-of-the-art tools, is that it is robust to reductions of read depth. Even at 20x average read depth, the integration of the Bayesian model provided the best results for genotyping accuracy in the HGSVC2 experiments, also demonstrating reliability for samples with different genetic diversity profiles. Additionally, this probabilistic model improved precision, which was evidenced by the analysis of the 47x ONT GIAB reads, which have a bigger error rate than CCS reads. This suggests that our algorithm is also robust to increased per-base error rates. Beyond tools comparison, our experiments indicate that an average read depth of around 20x is sufficient to achieve high detection and genotyping accuracy.

We believe that this work represents a significant contribution to current research on algorithms to analyze long DNA sequencing reads. We expect that the new functionality developed in NGSEP for SV detection from long reads will be useful for a large number of ongoing and upcoming research in population genomics for different species.

METHODS

Software development and integration within NGSEP

The algorithm described in this manuscript was implemented in Java 11 as a new option of the single sample variants detector functionality of the NGSEP software tool. The reuse of different NGSEP classes significantly decreased the development effort needed to code. Initially, for computing the input file a ReadAlignment iterator found in the ReadAlignmentFileReader class was used, given that it already collects all of the necessary information for each alignment. A Collection interface class named GenomicRegionSortedCollection allowed GenomicVariant interface implementing objects, such as Signature and CalledGenomicVariant objects, to be stored by sorted sequence, e.g chromosomes, and by genomic position. This also facilitated computed spanning alignments to specific variants. Additionally, the work made for genotyping SVs consisted mostly of programming the functionality to estimate likelihoods, given that the class CountsHelper allowed calculating the genotype posterior probabilities, as it was implemented before to genotype small indels and SNPs. The class diagram for the functionality inside of the NGSEP class context is shown in the supplementary figure 12.

Simulation experiments

In order to assess the behavior of our algorithm to identify and genotype SVs, a thorough benchmarking process was established to evaluate performance metrics of recall, precision, and efficiency. After an in-depth literature revision, four tools were included in the benchmark based on their performance and impact, including SVIM (version 2.0.0) (Heller et.al., 2019), Sniffles2 (version 2.0.6) (Sedlazeck et.al., 2018), CuteSV (version 1.0.13) (Jiang et.al., 2020) and Dysgu (version 1.3.11) (Cleal et.al., 2022). Both simulations and real cases were used to perform benchmark experiments. Output VCF files with SV calls were compared to Gold

Standard files using the software Truvari (English et.al., 2022), which provides recall, precision, f-score, and genotype accuracy of the evaluated SV genotype calls. This tool has been recommended by the GIAB consortium for benchmarking of SV callers (Zook et.al., 2020). Parameters for each dataset are provided in the supplementary table 2.

SVs were simulated with the software VISOR (Bolognini et.al., 2020), based on the *Arabidopsis thaliana TAIR10* reference genome (Lamesch et.al., 2012). A total of 4330 structural variants with a minimum length of 50 bp were simulated (2500 deletions, 1830 insertions, and 2065 inversions), and a genome containing these variants was generated. Next, reads with the characteristics of the Oxford Nanopore Sequencing Technology (ONT), including the error profile, were simulated with VISOR from this altered genome. Reads were aligned to the original reference genome using minimap2 (Li et.al., 2018). This pipeline was repeated to simulate four datasets of varying depths, including 20x, 30x, 45x, and 60x. The resulting alignments were used as the input data for all tools. The Human simulation from the T2T genome (Nurk et.al., 2022) was produced following the exact same pipeline.

GIAB high-confidence dataset

The Genome in a Bottle (GIAB) consortium has produced a high-confidence curated SV dataset, consisting of indel SVs identified from many biotechnologies, and multiple bioinformatic methods on the Ashkenazi son sample (HG002) against the GRCh37 reference genome (Zook et.al., 2020). All callers, including NGSEP, were used to discover SVs from a PacBio HiFi read alignment dataset of 56x depth and an ONT UL dataset of 47x depth, both sequenced from the same HG002 subject. Minimap2 (Li et.al., 2018) was used as the mapping tool to the GRCh37 reference genome. These alignments were randomly subsetted to produce 10x, 20x, 30x, and 40x input files in addition to the initial full-depth datasets, to assess the effect of depth variance on the calling algorithms. In order to include PBSV (version 2.9.0)

(https://github.com/PacificBiosciences/pbsv) we had to realign reads from the original HiFi HG002 sample using the pbmm2 mapper (available with PBSV).

From the GIAB gold standard, we used two ground-truth benchmark datasets, one including repetitive regions called Tier 1+2 (T1+2), and another retaining only non-repetitive regions, called Tier 1 (T1). We filtered these datasets retaining only SVs with length larger than 50 bp, and flagged with a "PASS" in the filter field of the VCF files. The final number of SVs for each experiment can be found in the Supplementary table 1.

HGSVC2 high-confidence samples

The work made by the HGSVC2 consortium provided high-confidence haplotype resolved calls for three samples of different ethnicities against the GRCh38 genome (Chaisson et.al., 2019; Ebert et.al., 2021). The supplementary table 1 shows the number of SVs of each type within each gold-standard dataset. PacBio HiFi reads were extracted from publicly available alignments for each of the three samples and were realigned with minimap2 (Li et.al., 2018). From each one, a 20x depth set of randomly chosen alignments was produced as input for the aforementioned callers.

For benchmarking using Truvari, we compared the results obtained keeping the default value of reciprocal overlap (70%) with those obtained reducing this parameter to 35% (-pct flag). We adjusted this parameter based on the initial results produced by Sniffles2. Similar to the experiments with the GIAB dataset, we also calculated the metrics using the complete dataset, and compared them with those obtained including only SVs in non repetitive regions of the reference genome.

Benchmark metrics

Truvari (English et.al., 2022) was used to produce the benchmark metrics, using symbolic alleles only. Performance metric calculation is specified as follows:

$$Precision = \frac{TP}{TP+FP} \qquad Recall = \frac{TP}{TP+FN}$$

$$GTAccuracy = \frac{HOM_{TP}^{HOM} + HET_{TP}^{HET}}{HOM_{TP}^{HOM} + HOM_{TP}^{HET} + HET_{TP}^{HET} + HET_{TP}^{HOM}}$$

$$Fscore = 2\frac{Precision \times Recall}{Precision + Recall} \qquad GTFscore = 2\frac{GTAccuracy \times Recall}{GTAccuracy + Recall}$$

Where GT-Accuracy is a metric obtained by estimating the fraction of the correctly genotyped true positive SVs over the total amount of true positives. Superscripts indicate their true genotype, which may differ from the caller classification. GTF-score is a variation of Fscore, to combine correct genotype classification with recall as the harmonic mean between both values.

Truvari also allows the inclusion of SVs in the truth and test call sets if they are located inside the genomic regions annotated in an input bed. This allowed us to produce the separate Tier1 and Tier1+2 benchmarks for GIAB and the non-repetitive-regions and all-regions for HGSVC2. Finally, this software does not take into account SVs with homozygous reference (0/0) genotype calls.

Execution environments

Arabidopsis simulation software executions including running all SV callers were done on an 8-core Ryzen 7 5800H with 16Gb RAM Laptop. Analysis of the human T2T simulation, the GIAB benchmark and the HGSVC2 benchmark, was performed on an Intel Xeon Gold computing node with a capacity of 42 threads and 565 GB RAM. Most of this computing power was required to align reads to the reference genomes. Processes for variants detection were restricted to a single core and 16Gb of RAM.

ACKNOWLEDGEMENTS AND FUNDING

This work has been supported by the "Patrimonio autónomo del Fondo Nacional de Financiamiento para la ciencia, la tecnología y la innovación Francisco José de Caldas" with the contract number 80740-441-2020, awarded by the Colombian Ministry of Science to JD. We also acknowledge the high-performance computing unit of Universidad de Los Andes for their technical support to conduct the benchmark experiments presented in this manuscript.

DATA AVAILABILITY

The *A. thaliana* TAIR10 reference genome used for simulations is available in the phytozome v.12 database (https://phytozome-next.jgi.doe.gov). The GIAB SV gold standard VCF file can be downloaded from the GIAB website (https://www.nist.gov/programs-projects/genome-bottle) as well as the bed files containing tier information. The GHC37 human reference genome can be found in the NCBI Assembly database (accession number GCA_000001405.1). PacBio HiFi reads are available at SRA BioProject accession number PRJNA586863. Oxford nanopore reads are located at the European Nucleotide Archive (ENA) under accession PRJEB37264.

Assets for the HGSVC2 benchmark are found in the project page at https://www.internationalgenome.org/data-portal/data-collection/hgsvc2. PB HiFi read files for the three samples are listed in this website, and deposited in the EBI ftp site at ftp://ftp.sra.ebi.ac.uk. Specifically, the GRCH38 reference genome can be found at http://ftp.1000genomes.ebi.ac.uk/vol1/ftp/data_collections/HGSVC2/technical/reference/20200513_hg38_NoALT/hg38.no_alt.fa.gz, and the vcf which contains the gold standard SVs for the three samples is available at

http://ftp.1000genomes.ebi.ac.uk/vol1/ftp/data_collections/HGSVC2/release/v2.0/integrated_callset/variants_freeze4_sv_insdel_alt.vcf.gz.

SOFTWARE AVAILABILITY

The algorithm presented in this study can be executed through the Single sample Variants Detector functionality of the open-source software Next Generation Sequencing Experience Platform (NGSEP). Releases of NGSEP are available at SourceForge (http://ngsep.sf.net). Life development is available on Git Hub (https://github.com/NGSEP). These are full details of the availability of supporting source code and requirements:

Project name: Next Generation Sequencing Experience Platform (NGSEP)

Project home page: http://ngsep.sf.net

Operating system(s): Platform independent

Programming language: Java

Other requirements: Java 11 or higher

License: GNU GPL

RRID: SCR_012827

Biotools ID: NGSEP

COMPETING INTEREST STATEMENT

The authors declare that there are no competing interests related to the publication of this manuscript.

REFERENCES

Alkan, C., Coe, B. P., & Eichler, E. E. (2011). Genome structural variation discovery and genotyping. *Nature Reviews Genetics*, *12*(5), 363-376.

Alonge, M., Wang, X., Benoit, M., Soyk, S., Pereira, L., Zhang, L., ... & Lippman, Z. B. (2020). Major impacts of widespread structural variation on gene expression and crop improvement in tomato. *Cell*, *182*(1), 145-161. http://doi.org/10.1016/j.cell.2020.05.021

Bolognini, D., Sanders, A., Korbel, J. O., Magi, A., Benes, V., & Rausch, T. (2020). VISOR: a versatile haplotype-aware structural variant simulator for short-and long-read sequencing. *Bioinformatics*, *36*(4), 1267-1269. https://doi.org/10.1093/bioinformatics/btz719

Carvalho, C. M., & Lupski, J. R. (2016). Mechanisms underlying structural variant formation in genomic disorders. *Nature Reviews Genetics*, *17*(4), 224-238. https://doi.org/10.1038/nrg.2015.25

Cericola, F., Lenk, I., Fè, D., Byrne, S., Jensen, C. S., Pedersen, M. G., ... & Janss, L. (2018). Optimized use of low-depth genotyping-by-sequencing for genomic prediction among multi-parental family pools and single plants in perennial ryegrass (Lolium perenne L.). *Frontiers in plant science*, *9*, 369. https://doi.org/10.3389/fpls.2018.00369

Chaisson, M. J., Sanders, A. D., Zhao, X., Malhotra, A., Porubsky, D., Rausch, T., ... & Lee, C. (2019). Multi-platform discovery of haplotype-resolved structural variation in human genomes. *Nature communications*, *10*(1), 1784. https://doi.org/10.1038/s41467-018-08148-z

Chiang, C., Scott, A. J., Davis, J. R., Tsang, E. K., Li, X., Kim, Y., ... & Hall, I. M. (2017). The impact of structural variation on human gene expression. *Nature genetics*, *49*(5), 692-699. https://doi.org/10.1038/ng.3834

Cleal, K., & Baird, D. (2022). Dysgu: efficient structural variant calling using short or long reads. *Nucleic Acids Research* 50(9): e53. https://doi.org/10.1093/nar/gkac039

Ebert, P., Audano, P. A., Zhu, Q., Rodriguez-Martin, B., Porubsky, D., Bonder, M. J., ... & Eichler, E. E. (2021). Haplotype-resolved diverse human genomes and integrated analysis of structural variation. *Science*, *372*(6537), eabf7117. https://doi.org/10.1126/science.abf7117

English, A. C., Menon, V. K., Gibbs, R., Metcalf, G. A., & Sedlazeck, F. J. (2022). Truvari: Refined structural variant comparison preserves allelic diversity. *bioRxiv*. https://doi.org/10.1101/2022.02.21.481353

Fujimoto, A., Wong, J. H., Yoshii, Y., Akiyama, S., Tanaka, A., Yagi, H., ... & Shimada, M. (2021). Whole-genome sequencing with long reads reveals complex structure and origin of structural variation in human genetic variations and somatic mutations in cancer. *Genome medicine*, *13*(1), 1-15. https://doi.org/10.1186/s13073-021-00883-1

Fumagalli, M. (2013). Assessing the effect of sequencing depth and sample size in population genetics inferences. *PloS one*, *8*(11), e79667. https://doi.org/10.1371/journal.pone.0079667

Gil J, Andrade-Martínez JS and Duitama J (2021) Accurate, Efficient and User-Friendly Mutation Calling and Sample Identification for TILLING Experiments. Front. Genet. 12:624513. doi: 10.3389/fgene.2021.624513

Gorkovskiy, A., & Verstrepen, K. J. (2021). The Role of Structural Variation in Adaptation and Evolution of Yeast and Other Fungi. *Genes*, *12*(5), 699. http://doi.org/10.3390/genes12050699

Heller, D., & Vingron, M. (2019). SVIM: structural variant identification using mapped long reads. *Bioinformatics*, *35*(17), 2907-2915. https://doi.org/10.1093/bioinformatics/btz041

Kirsche, M., Prabhu, G., Sherman, R., Ni, B., Battle, A., Aganezov, S., & Schatz, M. C. (2023). Jasmine and Iris: population-scale structural variant comparison and analysis. *Nature Methods*, *20*(3), 408-417. https://doi.org/10.1038/s41592-022-01753-3

Jiang, T., Liu, S., Cao, S., Liu, Y., Cui, Z., Wang, Y., & Guo, H. (2021). Long-read sequencing settings for efficient structural variation detection based on comprehensive evaluation. *BMC bioinformatics*, *22*(1), 1-17. https://doi.org/10.1186/s12859-021-04422-y

Jiang, T., Liu, Y., Jiang, Y., Li, J., Gao, Y., Cui, Z., ... & Wang, Y. (2020). Long-read-based human genomic structural variation detection with cuteSV. *Genome biology*, *21*(1), 1-24. https://doi.org/10.1186/s13059-020-02107-y

Lamesch, P., Berardini, T. Z., Li, D., Swarbreck, D., Wilks, C., Sasidharan, R., ... & Huala, E. (2012). The Arabidopsis Information Resource (TAIR): improved gene annotation and new tools. *Nucleic acids research*, *40*(D1), D1202-D1210. http://doi.org/10.1093/nar/gkr1090

Li, H. (2018). Minimap2: pairwise alignment for nucleotide sequences. *Bioinformatics*, *34*(18), 3094-3100. https://doi.org/10.1093/bioinformatics/bty191

Luan, M. W., Zhang, X. M., Zhu, Z. B., Chen, Y., & Xie, S. Q. (2020). Evaluating structural variation detection tools for long-read sequencing datasets in saccharomyces cerevisiae. *Frontiers in genetics*, *11*, 159. http://doi.org/10.3389/fgene.2020.00159

Mahmoud, M., Gobet, N., Cruz-Dávalos, D. I., Mounier, N., Dessimoz, C., & Sedlazeck, F. J. (2019). Structural variant calling: the long and the short of it. *Genome biology*, *20*(1), 1-14. https://doi.org/10.1186/s13059-019-1828-7

Nurk, S., Koren, S., Rhie, A., Rautiainen, M., Bzikadze, A. V., Mikheenko, A., ... & Phillippy, A. M. (2022). The complete sequence of a human genome. *Science*, *376*(6588), 44-53. https://doi.org/10.1126/science.abj6987

Parks, M. M., Lawrence, C. E., & Raphael, B. J. (2015). Detecting non-allelic homologous recombination from high-throughput sequencing data. *Genome biology*, *16*(1), 1-19. https://doi.org/10.1186/s13059-015-0633-1

Qiao, X., Li, Q., Yin, H., Qi, K., Li, L., Wang, R., ... & Paterson, A. H. (2019). Gene duplication and evolution in recurring polyploidization–diploidization cycles in plants. *Genome biology*, *20*(1), 1-23. https://doi.org/10.1186/s13059-019-1650-2

Schubert, E., Sander, J., Ester, M., Kriegel, H. P., & Xu, X. (2017). DBSCAN revisited, revisited: why and how you should (still) use DBSCAN. *ACM Transactions on Database Systems (TODS)*, *42*(3), 1-21. https://doi.org/10.1145/3068335

Sarwal, V., Niehus, S., Ayyala, R., Kim, M., Sarkar, A., Chang, S., ... & Mangul, S. (2022). A comprehensive benchmarking of WGS-based deletion structural variant callers. *Briefings in Bioinformatics*, *23*(4), bbac221. http://doi.org/10.1093/bib/bbac221

Schwarz, J. M., Lüpken, R., Seelow, D., & Kehr, B. (2021). Novel sequencing technologies and bioinformatic tools for deciphering the non-coding genome. *Medizinische Genetik*, *33*(2), 133-145. https://doi.org/10.1515/medgen-2021-2072

Sedlazeck, F. J., Rescheneder, P., Smolka, M., Fang, H., Nattestad, M., Von Haeseler, A., & Schatz, M. C. (2018). Accurate detection of complex structural variations using single-molecule sequencing. *Nature methods*, *15*(6), 461-468. https://doi.org/10.1038/s41592-018-0001-7

Smolka, M., Paulin, L. F., Grochowski, C. M., Mahmoud, M., Behera, S., Gandhi, M., ... & Sedlazeck, F. J. (2022). Comprehensive structural variant detection: from mosaic to population-level. BioRxiv, 2022-04.

Thibodeau, M. L., O'Neill, K., Dixon, K., Reisle, C., Mungall, K. L., Krzywinski, M., ... & Jones, S. J. (2020). Improved structural variant interpretation for hereditary cancer susceptibility using long-read sequencing. *Genetics in Medicine*, *22*(11), 1892-1897. https://doi.org/10.1038/s41436-020-0880-8

Wang, X., Gowik, U., Tang, H., Bowers, J. E., Westhoff, P., & Paterson, A. H. (2009). Comparative genomic analysis of C4 photosynthetic pathway evolution in grasses. *Genome biology*, *10*(6), 1-18. https://doi.org/10.1186/gb-2009-10-6-r68

Zook, J. M., Hansen, N. F., Olson, N. D., Chapman, L., Mullikin, J. C., Xiao, C., ... & Salit, M. (2020). A robust benchmark for detection of germline large deletions and insertions. *Nature biotechnology*, *38*(11), 1347-1355. https://doi.org/10.1038/s41587-020-0538-8

FIGURE LEGENDS

Figure 1. Procedures for intra-alignment and inter-alignment recollection of evidence (signatures) for indels, inversions, and duplications from reads aligned to a reference genome. Duplication assignment is performed only after insertion SVs have been called from signatures (SC=Soft Clip, LSC=Longest Soft Clip, ALN=Read alignment, POS=First position in the reference).

Figure 2. DBSCAN algorithm outlined in the context of variant calling applied to an example deletion event. A distance matrix is built from signatures using the euclidean distance of three numerical values: The difference between the first and last reference position and the length of each signature. BFS is used to build clusters based on core points (points with degrees larger than a given threshold) or points reachable from these core points.

Figure 3. Likelihood estimation for each of four possible scenarios for a diploid organism. In each case, the base 10 logarithm of the obtained value is calculated. For case 1, an example of the estimation of the log-likelihood value is shown, from a situation where the SV allele with a length of 85 bp is assumed, and a read alignment contains a call supporting the SV with a length of 90 bp. The HTS factor is a normalization constant based on the sequencing technology and the according error rate.

Figure 4. Simulation benchmarking results. The shape of points represents different depths for values of 20x,30x,45x, and 60x. A. F-score as a function of sequencing depth. B and C precision-recall curves of SV detection for alignments at different depths B. indels and C. inversions. The indel 20x F-score values are as follows: NGSEP: 99.1, Sniffles: 96.8, SVIM: 97.3, Dysgu: 98.8, CuteSV: 97.2. For SVIM, a QS filter > 10 was applied given that this provides the best results for the tool, where 0 filter provides very low precision and >20 filters

provide low recall. D. Single thread execution time of all callers as a function of the depth of the input alignments.

Figure 5. Performance metrics for PacBio HiFi and ONT data of HG002, using the T1+2 SV calls of GIAB as gold standard. A, C show precision-recall curves of SV discovery over varying depths for all callers on A. HiFi data, and C. ONT data. B, D show curves comparing genotyping accuracy with recall on B. HiFi data and D. ONT data. F-score values for the 20x (HiFi, ONT) depth mappings are: NGSEP: 69.88, 68.56; Sniffles: 68.14, 67.08; SVIM: 56.58, 31.58; Dysgu: 66.73, 64.34; CuteSV: 59.47, 58.08. SVIM and CuteSV had low values in some metrics, thus, some depth points for these tools are not included, but a trajectory line is left to indicate the results trend they followed over the different datasets.

Figure 6. HGSVC2 Benchmark experiments on 20x depth HiFi mappings for each sample (A. HG00514: Han Chinese, B. HG00733: Yoruba from Nigeria, C. NA19240: Puerto Rican). All performance metrics are shown based on the results of the tested variant callers, and their exact percentage values are portrayed over each column.

SUPPLEMENTARY FILES

Supplementary file 1. Accuracy and efficiency measures for the benchmark experiments presented in this study.

Supplementary file 2. Supplementary tables and figures

Figure 1

Click here to access/download;Figure;Figure1_signature_recollection.pdf

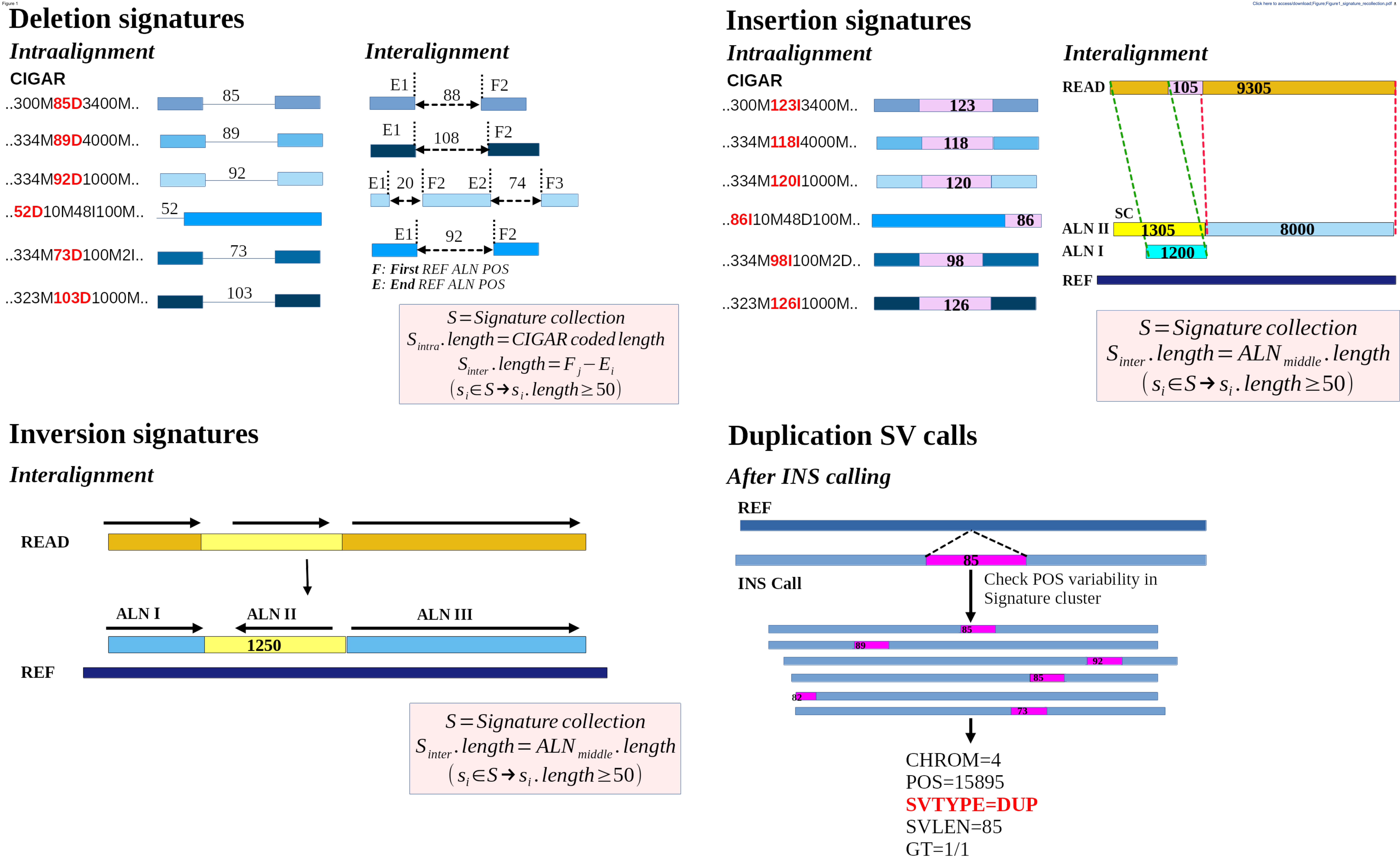

Figure 2
Click here to access/download;Figure;Figure2_DBSCAN_algorithm.pdf

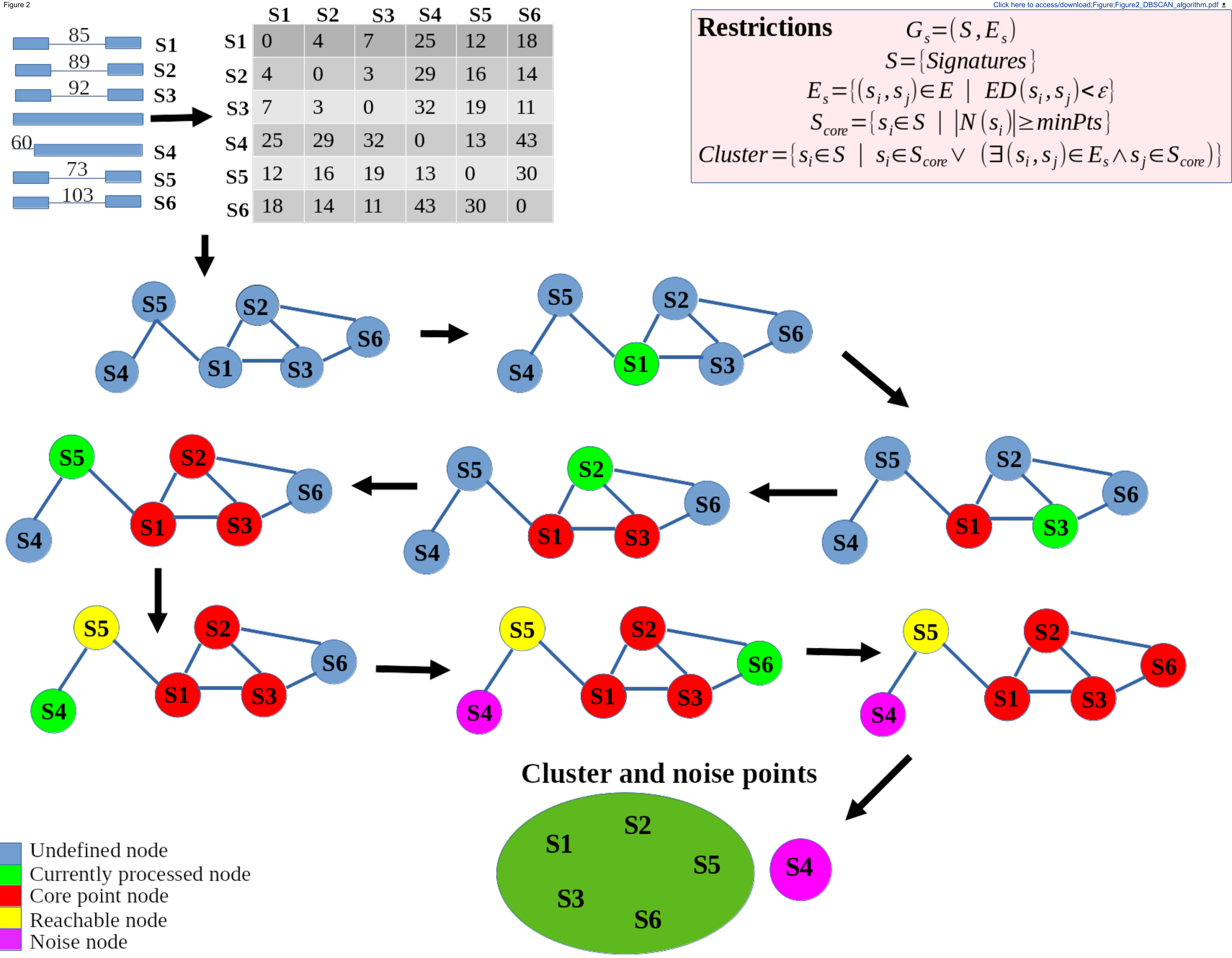

Figure 3

Click here to access/download;Figure;Figure3_likelihood_estimation.pdf

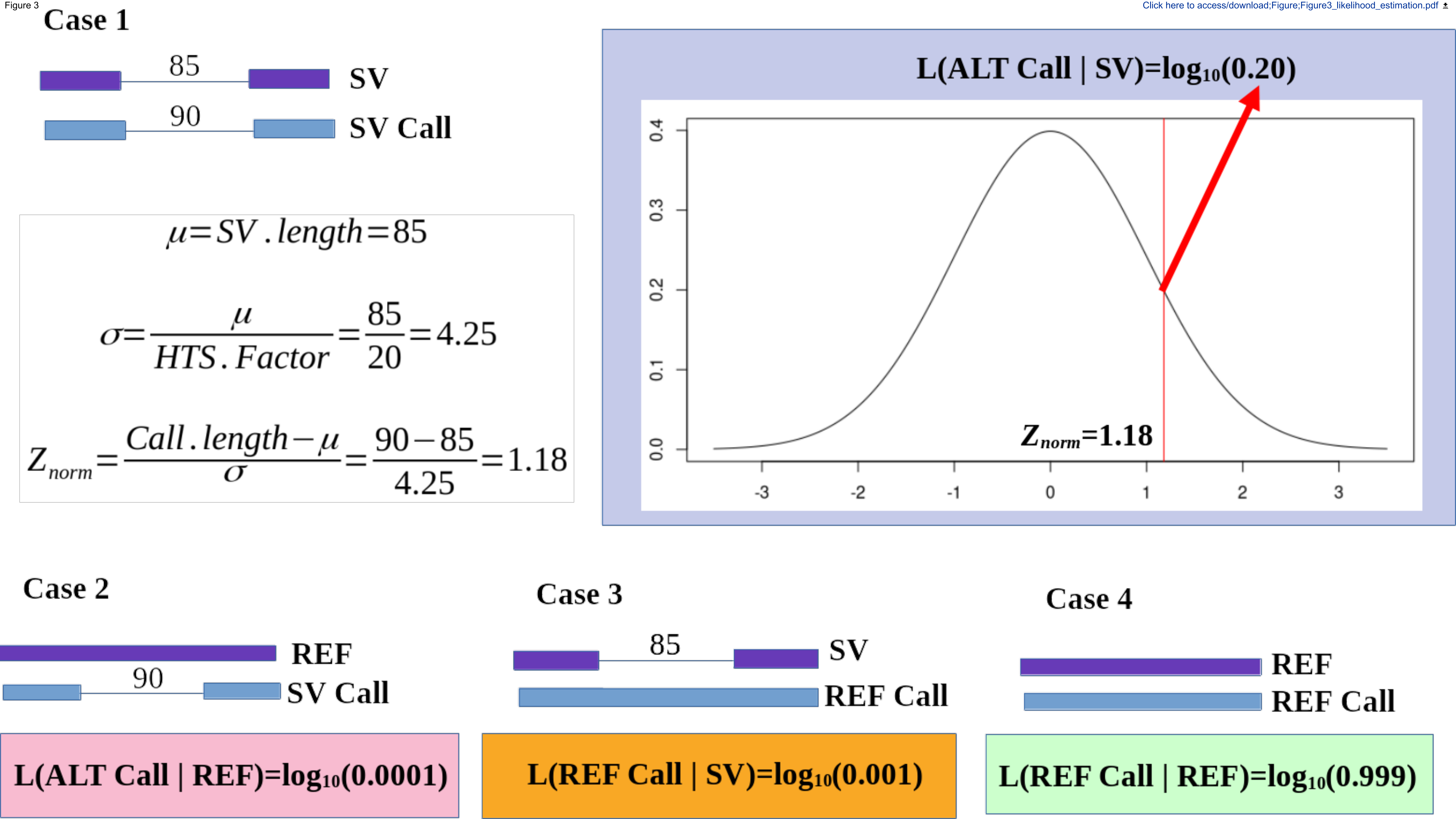

Figure 4

Click here to access/download;Figure;Figure4_SIMFigure.png

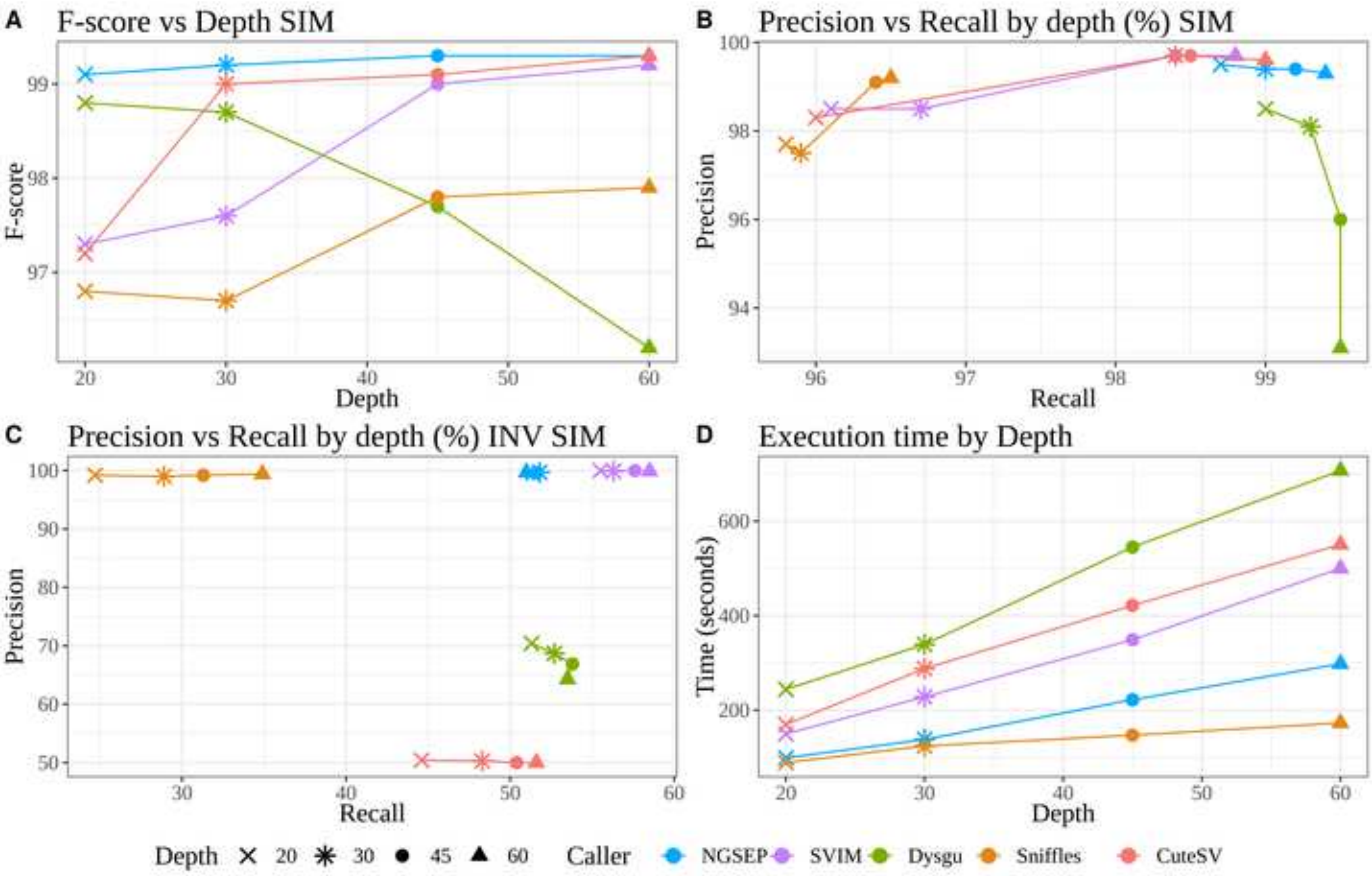

Figure 5

Click here to access/download;Figure;Figure5_HG002Tier1PlusTier2.png

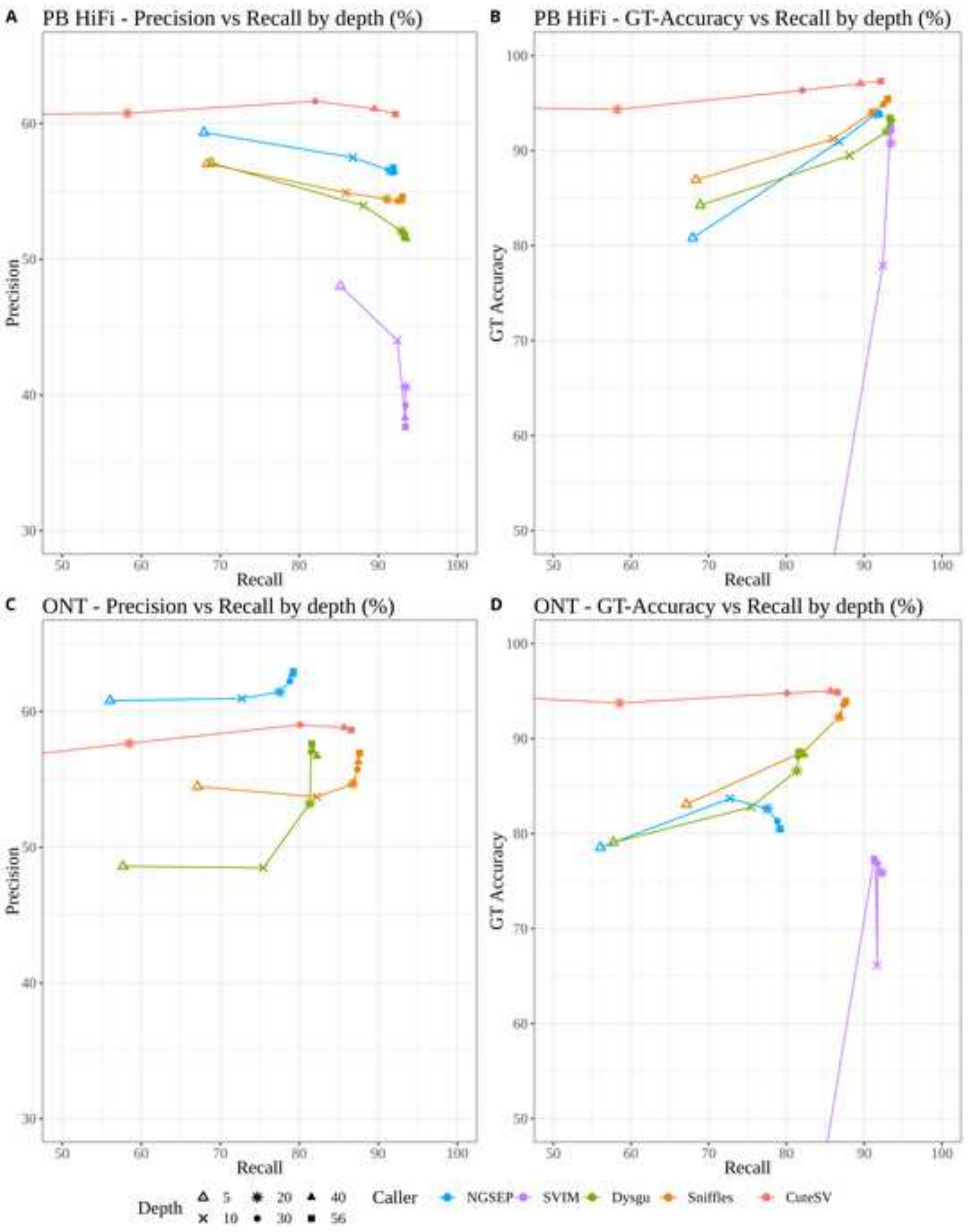

Figure 6

Click here to access/download;Figure;Figure6_HGSVC2.png

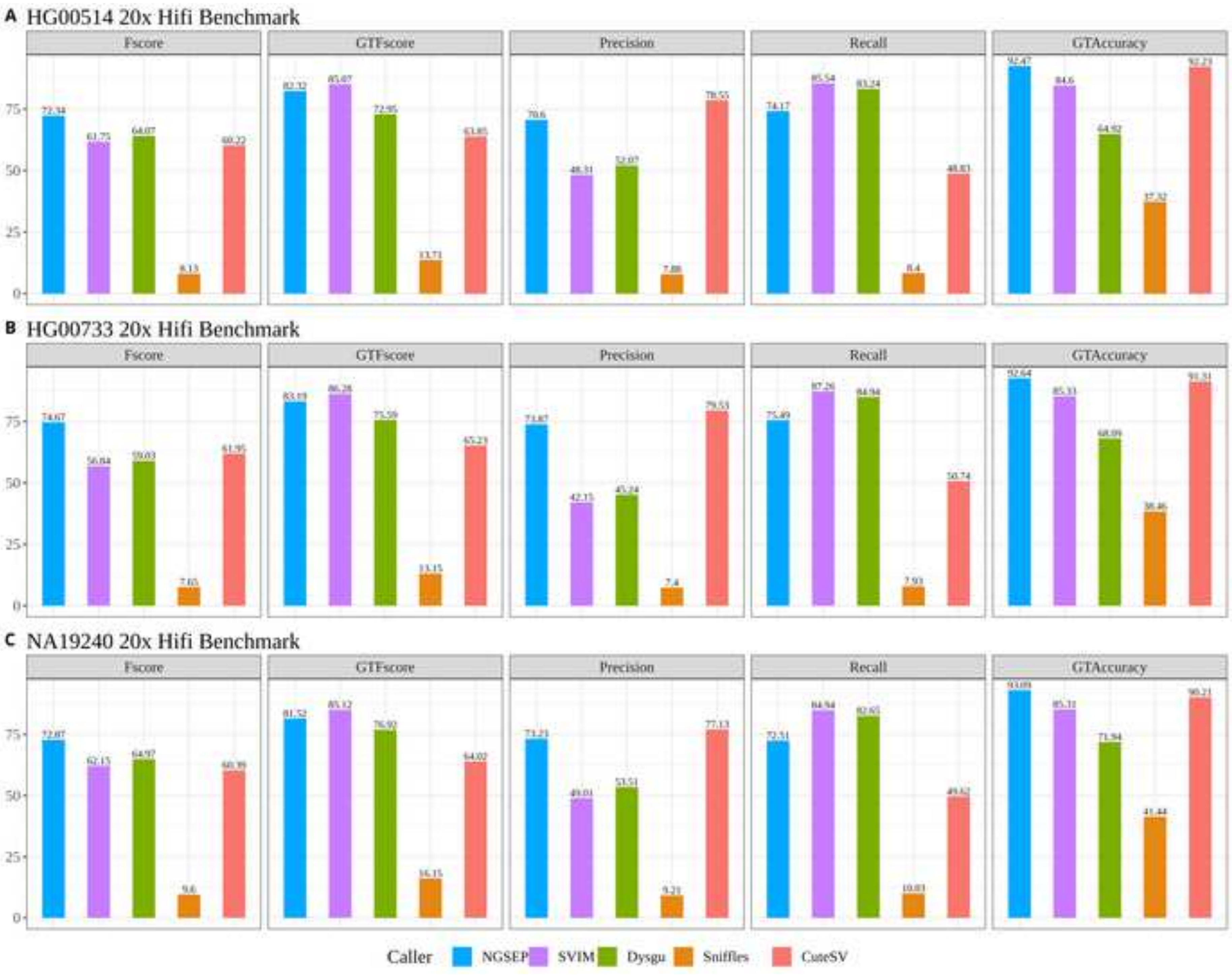

Supplementary File with results of benchmark experiments

Click here to access/download
Supplementary Material
SupplementaryFile1.xlsx

Supplementary tables and figures

Click here to access/download
Supplementary Material
SupplementaryInformation_20230802.pdf

2-Aug-2023

Dear editor Dr. Hans Zauner

Many thanks for your consideration of our manuscript 'A graph clustering algorithm for detection and genotyping of structural variants from long reads' and for giving us the opportunity to submit a revised version of the manuscript. We carefully read the concerns and comments of the referees. We performed the additional benchmark experiments and made substantial changes in the manuscript to address each comment. Please find our answers below for each specific comment. To facilitate the revision process, we marked in red the changes performed from the previous version of the manuscript.

This revised version was seen and approved by all co-authors of this manuscript. We appreciate your consideration of this version and we look forward to your assessment.

Sincerely

Jorge Duitama Ph.D
Associate professor
Systems and Computing Engineering Department
Universidad de los Andes
Bogotá, Colombia
Tel: (+57) (1) 3394949 Ext 1686.
E-mail: ja.duitama@uniandes.edu.co

GIGA-D-23-00070
A graph clustering algorithm for detection and genotyping of structural variants from long reads
Nicolás Gaitán; Jorge Duitama
GigaScience

Dear Dr. Duitama,

Your manuscript "A graph clustering algorithm for detection and genotyping of structural variants from long reads" (GIGA-D-23-00070) has been assessed by four reviewers. Although it is of interest, we are unable to consider it for publication in its current form. The reviewers have raised a number of points which we believe would improve the manuscript and may allow a revised version to be published in GigaScience.

Their reports, together with any other comments, are below.

Major concerns raised by the reviewers include the need for more rigorous validation and benchmarking, using up-to-date data and competitor tools in the process. Another important

point is that reviewers lacked sufficient information to reproduce your work, and other technical clarifications; and that the analysis was restricted to Tier1 of the GIAB data (reviewer #1).

If you are able to fully address the reviewers' points, we would encourage you to submit a revised manuscript to GigaScience.

In addition, please register any new software application in the bio.tools and SciCrunch.org databases to receive RRID (Research Resource Identification Initiative ID) and biotoolsID identifiers, and include these in your manuscript. Computational workflows should be registered in workflowhub.eu and the DOIs cited in the relevant places in the manuscript. These will facilitate tracking, reproducibility and re-use of your tool

Once you have made the necessary corrections, please submit online at:

If you have forgotten your username or password please use the "Send Login Details" link to get your login information. For security reasons, your password will be reset.

Please include a point-by-point within the 'Response to Reviewers' box in the submission system. Please ensure you describe additional experiments that were carried out and include a detailed rebuttal of any criticisms or requested revisions that you disagreed with. Please also ensure that your revised manuscript conforms to the journal style, which can be found in the Instructions for Authors on the journal homepage. If the data and code has been modified in the revision process please be sure to update the public versions of this too.

The due date for submitting the revised version of your article is 09 Aug 2023.

I look forward to receiving your revised manuscript soon.

Best wishes,

Hans

Hans Zauner
GigaScience

Reviewer reports:

Reviewer #1: The submitted study presents an innovative new software for calling de novo structural variants using long-read data. The novelty of the work lies in the application of the DBSCAN clustering algorithm to the problem of identifying SVs from a collection of SV signatures. The paper is well written an easy to follow, and the authors present some good performance metrics for their new software. However, I will highlight some short comings with the manuscript in its current form:

R. We thank the reviewer for the evaluation of our work. We are glad to hear that the main innovation of our work was properly communicated. We performed new benchmark experiments and modified significant parts of the manuscript to properly address the comments of the reviewer. Please find below our answers to each specific comment.

Major points:

The results are currently not reproducible due to missing information, and perhaps missing scripts. In the supplementary, the command used to run NGSEP is not given. I was able to repeat a run on PacBio HiFi data (HG002 sample, 8X coverage) using the following command, and will use the results of this run to raise some further points:

'/usr/bin/time java -jar NGSEPcore_4.3.1.jar SingleSampleVariantsDetector -runOnlySVs -i HG002.pacbio.cram -r ucsc.hg19.fasta -o HG002.ngsep -runLongReadSVs'

The output of this command was a gff file with 17277 SVs which is in line with the Supplementary Data file NGSEP_SVCaller_output_PBHifi_10x.vcf which contained around 20800 SVs using 10X coverage. However, the program only produced a gff file rather than a vcf file, so I was not able to analyze results further. Please can the authors state how to use the software to produce a vcf output and include any further scripts necessary for this.

R. We apologize for this issue with the output format. We also thank the reviewer for taking the time to test the software. On the positive side, the command line tried by the reviewer is accurate, according to the current manual. We included the SVs in the VCF file if SNVs were also called, but unfortunately we were not generating a VCF if SNVs were not called. We fixed this usability issue and, moreover, version 4.3.2 now produces a separate VCF for structural variants identified from long reads. We added to the Supplementary material the command line to run NGSEP and the procedure to replicate the benchmarking using Truvari

The runtime and memory usage of the above command was higher than expected compared to the presented benchmark on the simulated reads (gnu-time command was used to test). The test command appeared to use several threads during execution and finished in 24mins 25s and used 16.7 Gb of memory. The input cram file was only 8.9 Gb in size, so the memory usage may cause issues on some systems. The manuscript would be improved by running the benchmark on the real datasets (PacBio and ONT), and reporting memory usage alongside time.

R. We performed further testing experiments and we believe that both the usage of more than one core and the reports of high memory peaks are more related to the behavior of the Java Virtual machine, which is able to use resources that it sees idle. We have been able to run the functionality on 60x human data using up to 16 Gb of RAM in a laptop having only 4 processors by limiting the available memory using the java option -Xmx. We included in the manuscript the use of memory and time for the GIAB benchmark (see supplementary figure 8). Additionaly, we included the java option in the description of the command line.

The manuscript would be improved by presenting data on the Tier2 HG002 GIAB benchmark dateset, not just Tier 1 regions. Although Tier1 regions are the highest accuracy regions, these also present an 'easy case' for long read callers, as demonstrated by the results - there are relatively small differences among callers in these regions. A key use-case of long-reads is to analyse more difficult genomic regions; therefore, it would be of interest to readers to assess Tier1+2 regions. A quick comparison of NGSEP, dysgu, svim and sniffles indicated large differences in the total number of deletion SVs >=50 bp called and most of these differences probably arise outside of Tier1 regions.

R. We actually had comparisons including tier1+tier2 in an earlier version of the work but we used only tier1 based on a suggestion of a previous reviewer. We finally decided to report both results. The new results are shown in Figure 5 and the new supplementary figures 2 to 6.

The total numbers of SVs called per genome should be presented and discussed. The numbers of called SV that intersect the Tier1+2 and regions should be shown.

R. Following the suggestion, we now refer in the methods section to the supplementary table 1, which contains a thorough description of the number of SV types included in all the datasets used for benchmarking.

Minor points:

The software versions of tools are not given.

R. We improved the results to show the exact version of each tool included in the benchmark experiments, given that it was only specified in the methods section.

Please could the authors also include details of the computer system used for benchmarking.

R. We added a methods section to describe the execution environment used for each experiment.

The software does not report translocations/BND, in contrast to the comparison tools, please could a sentence be included to indicate this.

R. We clarified in the discussion that at this stage the software does not call translocations

The use of a star in the plots to indicate an F1 score is confusing to the reader as it looks like the star is an extra data point rather than an annotation. The stars would be easier to read if they were moved to a legend.

R. We followed the suggestion and moved the F1 scores to the legend

CuteSV appeared to show unexpectedly poor performance at low coverage values, could the authors comment on why this was the case in their experiments.

R. We took a close look at the manuscript to understand the method implemented in CuteSV. The clustering algorithm is relatively simple. They first create clusters based on distance between genomic coordinates, and then they create subclusters based on length differences. The process to define clusters and subclusters is based on simple decision rules tuned by fixed parameters for each event type (which can be changed by user options), such as the distance between signatures, the absolute number of reads needed to support a variant, and the percentage of the average event length within a cluster after which a new subcluster is formed. Given that a large space of parameter options are needed to be tuned for each experiment, we only ran the experiments with default values. In particular, the default value of the “--min_support” parameter (10) is likely to explain the outcome that we obtained. After this analysis, we believe that our method adapts more naturally to different event types and sequencing technologies because we did not have to tune parameters for each particular experiment. We improved the discussion adding a brief summary of this rationale.

SVIM showed very poor precision on nanopore data, could the authors comment on this finding - previous studies suggest SVIM performance suffers on ONT data due to a high number of duplicated true-positive SVs (Cleal et al., 2021).

R. In our experience with SVIM, we observed that they changed their clustering algorithm from a Maximal Clique finder to a hierarchical clustering process. This algorithm produces flat clusters that may fail to resolve close calls. It seems this leads to duplicated SV calls from individual or few nearby signatures, as evidenced by the ratio of duplicate true-positive calls to the number of true-positive calls in Cleal et.al (2022). Additionally, discordant signals may be grouped, and called as a single SV, as the example attached below where only SVIM calls the insertion in the picture from the 10x GIAB benchmark. This is consistent with our results. Thus, we decided to filter their output for comparison as explained in the supplementary table 2 to improve their precision. Further filtering by quality score leads to significant decreases in recall.

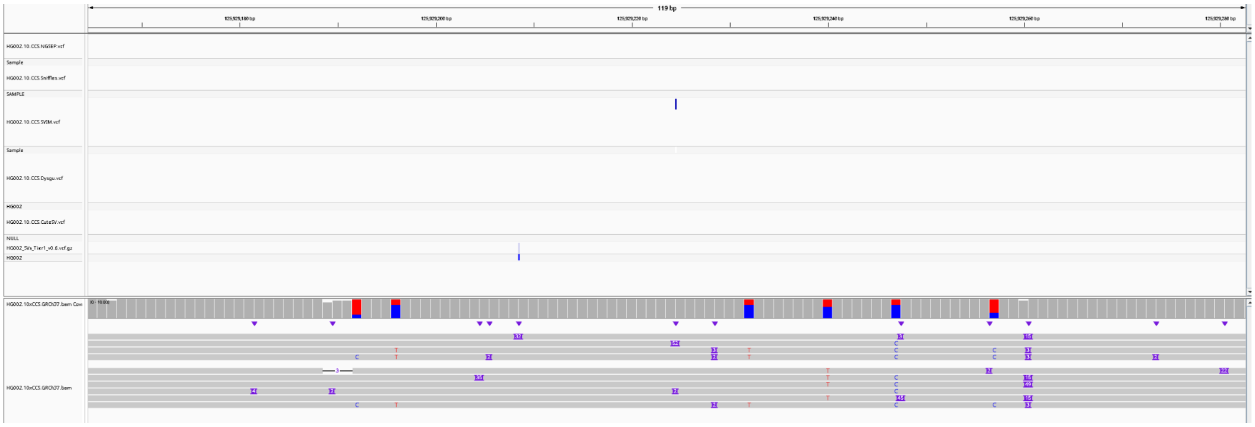

Example 1: SVIM groups all these intra-alignment signatures which differ greatly in position, and length. Regardless of their difference, their algorithm produces a single SV call.

The manuscript would be improved by including a lower coverage test on real data, e.g. 5X coverage, as many researchers are interested in utilizing lower coverages.

R. We included the results at 5x in the new simulation of a human individual and in the results with HG002.

Reviewer #2: The authors of the manuscript "A graph clustering algorithm for detection and genotyping of structural variants from long reads" introduce a new method for long read based SV analysis integrated in their NGSEP framework. The main advancement of what they are presenting is the clustering and genotyping methodology, which they show it improves SV detection based on simulated and GIAB benchmark data. The manuscript is overall easy to understand and to follow the points made by the authors. Nevertheless, I feel like I am missing many details on why the algorithm should perform better than existing methodologies that also uses similar concepts on genotyping. In the following I list my questions and concerns in no particular order:

R. We thank the reviewer for the evaluation of our work. We are glad to hear that the text was easy to follow. We performed changes to the methods, results and discussion to address the comments of the reviewer. Please find below our specific answer to each comment.

1. There are overall not many details given that helps me to understand why your algorithm should perform better than the others. This is a bit tricky for me, because I like to understand this to better understand the results that you are showing. For example, you give great detail about why you cluster but then you are not giving much on concrete examples where this clustering matters.

R. We improved the discussion to provide a rationale on why the algorithm could work better than other solutions. Specific reasons for the observed differences against SVIM and CuteSV are described in the answers to the minor comments of reviewer 1. Below we provide examples in which our clustering algorithm provides improved analysis of candidate SV signatures.

2. Their approach of clustering might be novel, but I fail to understand the differences this makes from traditional clustering approaches. Like it is often hard to cluster signals accurately as SV often occur in repeat regions and thus over or under merging can happen. It would be great if you could show some examples of this. It would be interesting to see (since it is one of the main novelties) how it behaves under different circumstances. Like some methods take the SV size into account when clustering the reads to the individual breakpoint. Is that also the case here?

R. The event length is included as one of the dimensions for DBSCAN. Signature false merging is in fact one of the main challenges, especially in repeat regions. We include here three examples from the 10x GIAB benchmark in which our algorithm performed a better signal clustering, compared to other approaches.

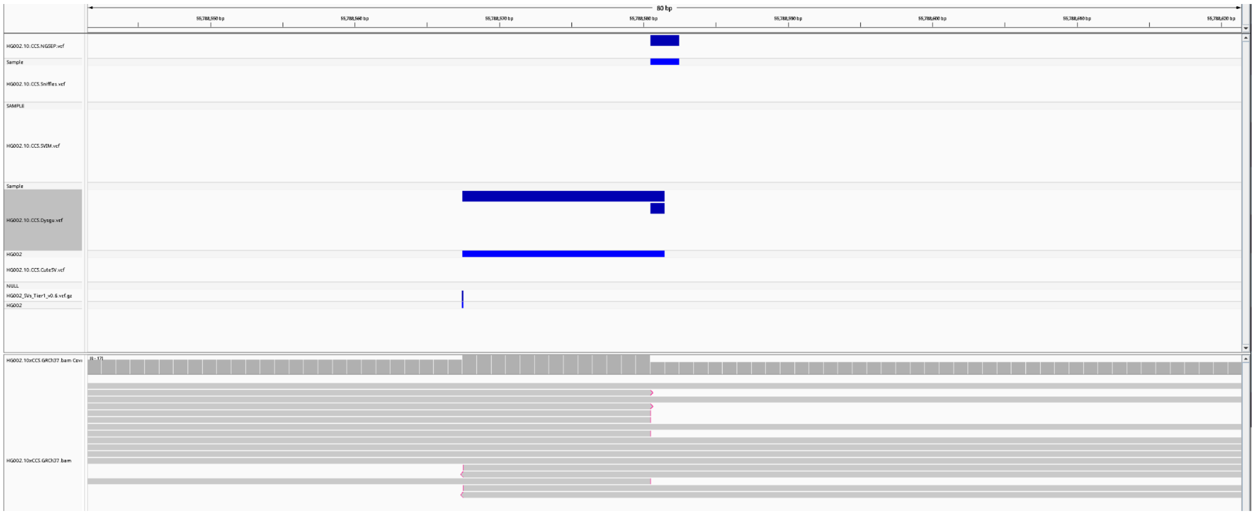

Example 1: Only NGSEP and Dysgu detect this true positive call. This comes from inter-alignment signatures which are very difficult to cluster because they usually differ in position, and length, causing some algorithms to avoid merging these signals. Our algorithm demonstrates here that it can correctly group complicated signals as a single cluster.

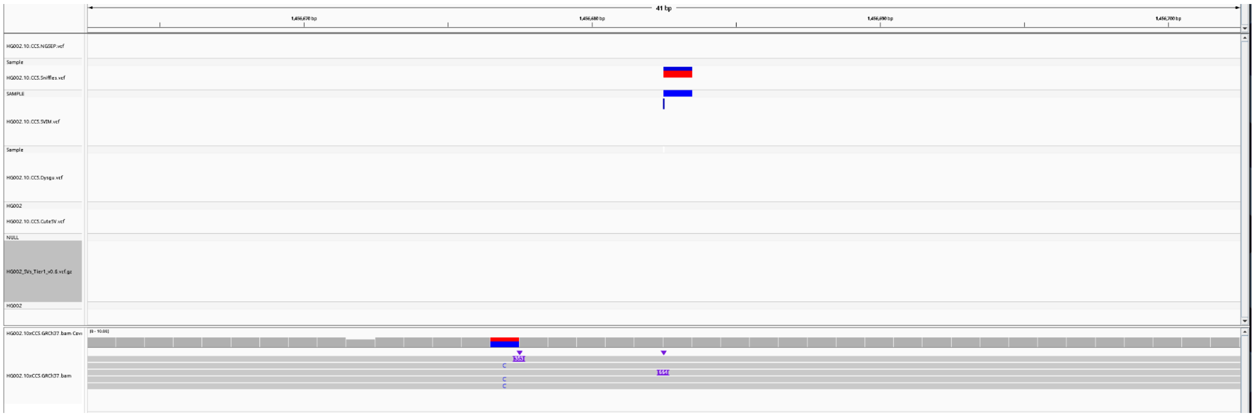

Example 2: Here, both Sniffles2, and SVIM call an insertion based on two discordant signals. Our clustering algorithm does not group these two signatures together.

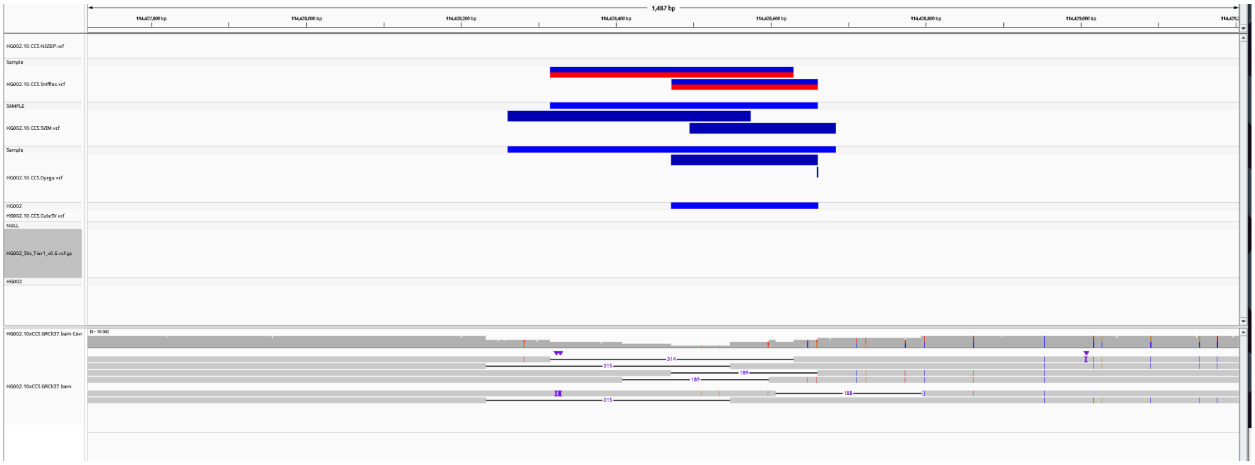

Example 3: Here, there seem to be two groups of deletion signatures considering their length, but their positions are somewhat inconsistent. This is a case where our clustering algorithm creates both clusters of signatures, avoiding merging discordant signals, but in the genotyping phase they are filtered due to their inconsistent positions, and the lack of evidence to produce both calls. This way, our algorithm avoids two false positives which are called by other tools.

3. I am also failing to fully understand how an improved clustering can improve the precision of the method, but maybe this comes from genotyping? It would be insightful if the authors give more details on how they filter their SV candidates.

R. You are right on your assessment that both phases are important for our algorithm to achieve high precision values. In the clustering phase, the DBSCAN algorithm allows for a better differentiation of clusters compared to other algorithms because it is capable of producing different cluster geometries. Even apparently similar points may border two different clusters. In the SV calling context for example, we can precisely call SVs that are adjacent in the genome but vary in length, avoiding false-merging events and a false positive call. In the genotyping phase, due to the power of our Bayesian model, if the posterior hypothesis results in a reference homozygote, we can filter that SV call from the output. We improved the results to describe this process more clearly.

4. It was interesting to me that the authors identify deletions, insertions and inversions, but didn't call duplications or translocations. Is there any reason for this?

R. We improved the results section to better explain that duplications are called after analyzing the variability of starting positions for insertion calls. We decided to not include translocations at this stage because signatures related to these events are more difficult to identify and to cluster, compared to other SV types. Also, we could not find gold standard datasets from real data to assess the reliability of translocation calls.

5. For the simulated data set, how did you evaluate the data set?

R. We improved the methods to explain better the simulation procedures, the number of simulated events and the evaluation procedure. Because the data is simulated, we have a perfect gold standard of structural variants that should be called, and hence we can calculate precision and recall.

6. For the GIAB I saw that you are using custom parameters for Truvari? I see the -p 0.0, which I think refers to the sequence identify comparison. What I am more wondering is the -r 1000. Could you justify this?

R. The -r 1000 parameter allows Truvari to compare SVs that are located at maximum 1000bp between each other, in terms of reference coordinates. This is specially important for bigger SVs, given that breakpoints are not consistently detected between callers. Using the default -r 500 could lead to underestimating the recall of some tools that may miss on the coordinates of the variant but are detecting it nonetheless.

7. I would have liked to test this SV caller myself but it seems that this is not so easily possible since I have to install the entire software analysis suit.. or is there a way around this?

R. To run NGSEP from the command line you just need to have the general purpose java virtual machine (v11 or superior) installed in your computer. You can either install the versions from Oracle or the openJDK in the case of linux. After that, you just need the jar file of NGSEP (currently version 4.3.2), which you can download from our website (https://sourceforge.net/projects/ngsep/files/Library/). Once downloaded, if you go to the download folder in the command line and type:

java -jar NGSEPcore_4.3.2.jar

You will see the different functionalities offered by the software. In particular, the functionality described in this manuscript can be executed typing:

java -jar NGSEPcore_4.3.2.jar SingleSampleVariantsDetector

Finally, the command we used for our experiments has been included in the Supplementary table 2.

8. I am missing also some details on the simulation. How many SV were simulated per data set, whats the sizes of SV simulated etc. I see some detail in the methods, but it only says indel were simulated and not inversion?

R. We improved the methods to better explain the simulation procedures, the number of simulated events and the evaluation procedure. We included the number of inversions in the text. You can find the exact breakdown by type of number of SVs for each gold standard dataset in the Supplementary Table 1.

Reviewer #3: The paper is well written and the results are good.
The methods described here remind me of those from Jasmine and Iris (Kirsche 2023), and I would like to authors to help me understand the differences between the two clustering approaches.

R. Thanks for your assessment of the manuscript. We went over the work of Kirsche et al., 2023 and we found that Jasmine solves a relatively different problem, namely the merging of individual SV calls. We are glad to see that they follow an alternative similar to that implemented in NGSEP. Going over the details of the algorithm, the Jasmine algorithm represents SVs as points in a two dimensional Euclidean space consisting of the first reference coordinate and length of the variants. Then, they produce a graph with the SVs as vertices by adding distance weighted-edges between them, complying with different restrictions. In our approach, we also include the end coordinate of each event and hence our graph represents signatures into a 3D Euclidean space, restricting the complete graph based on a distance threshold. The major divergence comes when Jasmine produces a Minimal Spanning Forest, to merge SVs into a single call if they are found in the same tree. In contrast, we use the DBSCAN algorithm to cluster the signatures into an individual SV call, processing the graph through a Breadth First Search. We included the paper in the references and made an appropriate citation in the discussion.

Also, it would be helpful to break up the results by SV type to see if the improvements seen are general or specific.

R. We improved the results to show clearly the behavior of the method separately for insertions and deletions in the human GIAB benchmark experiments (Supplementary figures 3-6). We only could assess inversions in the simulations with the Arabidopsis genome because the human gold standard datasets do not include inversions. The results for inversions are separated from those of insertions and deletions in figure 4.

Reviewer #4: In this manuscript named "A graph clustering algorithm for detection and genotyping of structural variants from long reads", Gaitán et al represented an algorithm that detect genomic structural variants (SV) from long reads. Albeit the existence of multiple long-read based SV discovery methods, I can see the value of this method to the field of genomic SV and long-read sequencing. However, the benchmarking data and methods described in this manuscript are outdated, and significant revision should be applied before this manuscript can qualify for publication. Below are my specific comments:

R. We thank the reviewer for the assessment of our work. We are glad to hear that the work is perceived as valuable by the reviewer. We performed further benchmark experiments and improved the results and discussion to address each comment of the reviewer. Please find below our specific answer to each comment.

1. Benchmarking methods: the authors included SVIM, Sniffles, CuteSV and Dysgu as benchmarking methods. However, there are newer and better algorithms available, including

Sniffles2 (which has significant improvements over Sniffles) and PBSV. In addition, assembly based long-read SV methods, such as PAV, could also be considered for benchmarking.

R. We mentioned the exact version numbers for the different tools in the results section. Although we cited the manuscript of Sniffles1, the results shown in the initial review were generated with the 2.0.6 version of Sniffles. In any case, we double checked that the benchmark was performed using the latest version of each tool. Regarding PBSV, we did not include it in the initial benchmark because we could not find a publication related to this tool. We tried to include this tool directly, but unfortunately it could not run with the minimap alignment files. Thus, we had to realign the reads of the original alignment files with the pbmm2 minimap2 wrapper. Since this would represent a different dataset for benchmarking, we ran all tools on these alignments. Please see the supplementary figure 7 for details. Overall, PBSV ranked below CuteSV and NGSEP,on precision, recall and GT accuracy.
Regarding comparisons with a de-novo assembly and contig mapping approach, we believe that results of de-novo assembly comparisons are generally better than those of read alignment based SV callers. However, they are more expensive given that larger depths are required to achieve a high quality *de-novo* genome assembly, in comparison to low-depth long-read sampling. Additionally, the two gold-standard datasets we used for real data benchmarking include calls from assembly based methods (especially HGSVC). In the second version, this consortium improved their callset by using the PAV algorithm. Hence, we believe that benchmarking against de-novo assembly methods will not make a fair comparison in these cases.

2. Simulation data benchmarking: it's useful to learn the performance of simulation data in the genome of Arabidopsis thaliana, but most important to simulate the human genome for benchmarking.

R. We did not do this before because we had the HG002 data. We performed the suggested simulation based on the T2T genome, and described the results (Supplementary figure 1).

3. Real data benchmarking: Gaitán et al compared the performance of NGSEP using HG002 base on the reference of GRCh37.Hhowever, in most of the current studies, both short-read and long-read data, are aligned against GRCh38 or T2T, and these data are of significantly more interest to the filed. Please either re-align the GIAB data against the newer reference genomes for the benchmarking or use other data such as those generated by the human genome structural variation consortium (HGSVC, Check Chaisson et al. 2019. and Ebert et al. 2021)

R. Although we agree that the GRCh38 genome, or even the T2T genome would be more interesting to perform these benchmark experiments, the public gold standard of GIAB to perform independent benchmark experiments is currently available only for GRCh37. Nevertheless, following this comment we found the dataset of the HGSVC consortium and we performed further benchmark experiments using this dataset, achieving very good performance. Please see the results in figure 6 and supplementary figures 9-11. We thank the reviewer for leading us to this asset, and allowing us to further improve our benchmark experiments.
