# Supplementary material for: A graph clustering algorithm for detection and genotyping of structural variants from long reads
Source: Gigascience. 2024 Jan 11;13:giad112. doi: 10.1093/gigascience/giad112 (PMC10783151; doi:10.1093/gigascience/giad112)
Supplement: giad112_GIGA-D-23-00070_Revision_2 [file giad112_giga-d-23-00070_revision_2.pdf]

GigaScience

A graph clustering algorithm for detection and genotyping of structural variants from long reads

--Manuscript Draft--

**Manuscript Number:**	GIGA-D-23-00070R2
**Full Title:**	A graph clustering algorithm for detection and genotyping of structural variants from long reads
**Article Type:**	Technical Note
**Funding Information:**	Ministerio de Ciencia, tecnología e innovación de Colombia (80740-441-2020) — Dr. Jorge Duitama
**Abstract:**	Structural variants (SV) are genomic polymorphisms defined by their length (>50 bp). The usual types of SVs are deletions, insertions, translocations, inversions, and copy number variants. SV detection and genotyping is fundamental given the role of SVs in phenomena such as phenotypic variation and evolutionary events. Thus, methods to identify SVs using long-read sequencing data have been recently developed. We present an accurate and efficient algorithm to predict germline SVs from long-read sequencing data. The algorithm starts collecting evidence (Signatures) of SVs from read alignments. Then, signatures are clustered based on a Euclidean graph with coordinates calculated from lengths and genomic positions. Clustering is performed by the DBSCAN algorithm, which provides the advantage of delimiting clusters with high resolution. Clusters are transformed into SVs and a Bayesian model allows to precisely genotype SVs based on their supporting evidence. This algorithm is integrated into the single sample variants detector of the Next Generation Sequencing Experience Platform (NGSEP), which facilitates the integration with other functionalities for genomics analysis. We performed multiple benchmark experiments including simulation, and real data, representing different genome profiles, sequencing technologies (PacBio HiFi, ONT), and read-depths. The results show that our approach outperformed state-of-the-art tools on germline SV calling and genotyping especially at low depths, and in error-prone repetitive regions. We believe this work significantly contributes to the development of bioinformatic strategies to maximize the use of long-read sequencing technologies.
**Corresponding Author:**	Jorge Duitama<br><br>COLOMBIA
**Corresponding Author Secondary Information:**	
**Corresponding Author's Institution:**	
**Corresponding Author's Secondary Institution:**	
**First Author:**	Nicolás Gaitán
**First Author Secondary Information:**	
**Order of Authors:**	Nicolás Gaitán
	Jorge Duitama
**Order of Authors Secondary Information:**	
**Response to Reviewers:**	6-Oct-2023<br>Dear editor Dr. Hans Zauner<br>Many thanks for your assessment of our manuscript 'A graph clustering algorithm for detection and genotyping of structural variants from long reads' and for giving us the opportunity to submit a revised version of the manuscript. We carefully read the follow up comments of the reviewer. We updated the additional benchmark experiments and made changes in the manuscript to address each comment. Please find our answers below for each specific comment. To facilitate the revision process, we marked in red

Powered by Editorial Manager® and ProduXion Manager® from Aries Systems Corporation

the changes performed from the previous version of the manuscript.
This revised version was seen and approved by all co-authors of this manuscript. We appreciate your consideration of this version and we look forward to your assessment.

Sincerely

Jorge Duitama Ph.D
Associate professor
Systems and Computing Engineering Department
Universidad de los Andes
Bogotá, Colombia
Tel: (+57) (1) 3394949 Ext 1686.
E-mail: ja.duitama@uniandes.edu.co

GIGA-D-23-00070R1
A graph clustering algorithm for detection and genotyping of structural variants from long reads
Nicolás Gaitán; Jorge Duitama
GigaScience

Dear Dr. Duitama,

Your revised manuscript "A graph clustering algorithm for detection and genotyping of structural variants from long reads" (GIGA-D-23-00070R1) has been assessed again by one of the reviewers. Based on the latest report, I feel the manuscript can be potentially acceptable for publication in GigaScience, once you have carried out some important follow-up revisions suggested by the reviewer.

The latest report is below.

Once you have made the necessary corrections, please submit a revised manuscript online at:

If you have forgotten your username or password please use the "Send Login Details" link to get your login information. For security reasons, your password will be reset.

Please include a point-by-point within the 'Response to Reviewers' box in the submission system. Please ensure you describe additional experiments that were carried out and include a detailed rebuttal of any criticisms or requested revisions that you disagreed with. Please also ensure that your revised manuscript conforms to the journal style, which can be found in the Instructions for Authors on the journal homepage. If the data and code has been modified in the revision process please be sure to update the public versions of this too.

The due date for submitting the revised version of your article is 20 Dec 2023.

We look forward to receiving your revised manuscript soon.

Best wishes,

Hans Zauner
GigaScience

Reviewer reports:
Reviewer #1: I wish to thank the authors for the detailed response, including more data, and for addressing most of the points I raised in the initial review. I have a few outstanding issues with the manuscript, which should not be too difficult to address.

1. I apologize for not finding the version numbers in the original submission. However, the versions of most of the tools are out of date by roughly a year. CuteSV, dysgu and sniffles have received substantial updates. The manuscript would be improved if these

were updated to recent versions.

R. We thank the reviewer for the follow up revision and comments. We updated the benchmark experiments using the newest versions of each tool at this moment (Dysgu v1.6.1, CuteSV v2.0.3, Sniffles v2.2, and SVIM v2.0.0) and adjusted the manuscript according to the new results. We made different observations when comparing the new versions against the older ones. The improvements of CuteSV in our updated experiments are mostly caused by the change in the value of the min_support parameter, rather than the version update. We follow this discussion in-depth in the response to the second comment. Regarding Dysgu, we found that the results on the GIAB dataset improved significantly in terms of precision for PacBio data, but this was not the case for the Tier 1+2 benchmark from the Nanopore data, even with the recommended parameters. In particular, for the GIAB pbmm2 remappings, we encountered segmentation fault errors that made it impossible to use v1.6.1, thus, we downgraded to Dysgu v1.5 for that specific dataset. Since Dysgu is in active development and this outcome is probably produced by a bug that could be easily fixed, we decided not to report in the paper this bug and the version downgrade that we had to do. Additionally, the recall of Dysgu on the HGSVC2 samples decreased significantly compared to v1.3.11, while the improvement of precision was not enough to compensate, generating a reduction in F1-score overall. The results of Sniffles did not change significantly, and the issue of reported doubled lengths for SVs discovered from the HGSVC2 samples remains with the updated version. Therefore, we applied the same previous solution to include a benchmark with a smaller reciprocal overlap parameter for Truvari (Supplementary Figures 9,11). Nevertheless, updating software versions allowed us to improve our benchmarking experiments and provided insights into the best practices for using each caller.

2. Going back to the point that cuteSV shows low sensitivity. As now pointed out by the authors, this is due to the default parameter for minimum read support, which is normally set at 10 for the tool. This probably explains the poor performance at low coverages and the excellent performance at high coverages. However, testing cuteSV with default settings on low coverage samples (5x, 10x) is not a fair test of the tool. Can I suggest making a precision-recall curve at one or two fixed coverage values but vary the minimum-support of the tools. This would be an interesting supplementary figure and would show how optimizing the minimum support parameter could affect performance.

R. We followed this suggestion and updated the experiments using the latest version of CuteSV, and reducing the minimum read support to 5x. However, we ran into an issue running the latest version of CuteSV (v2.0.3) on the original alignments downloaded from the GIAB repository. The tool produced low recall for all depths. This issue was fixed running this version with the pbmm2 realigned datasets. This unexpected behavior did not happen with version 1.0.13 of CuteSV.

Going back to the min support parameter, we also performed experiments varying the this parameter (1,5,10,15,20) on the PacBio 20x HG002 reads, realigned with pbmm2, and with the ONT reads for HG002 (See supplementary figure 13). We observed that lowering the min_support value to 5 produced the best performance metrics. In comparison to the results with the parameter set to 5, a value of 1 creates more false positives in exchange for better sensitivity, while bigger values significantly decrease recall with no precision improvement. Hence, we applied a min_support=5 to all runs of CuteSV in all the benchmark experiments, improving the performance of this software overall. In particular, this change produced a significant improvement in the performance of CuteSV on the HGSVC2 benchmark.

3. The performance of NGSEP seems to remain high at different coverages. Could the authors explain why this might be the case, for example are some dynamic thresholds used, or is this all down to the genotyping?

R. Your assessment is correct regarding the genotyping phase as the main cause for the consistent performance of NGSEP at different coverages, particularly regarding precision. Our Bayesian genotyping algorithm accurately predicts most false positive cases where inconsistent evidence supports an SV call according to the total amount

	of spanning read alignments, classifying the call as a Homozygous reference and filtering it out from the vcf output. In particular, when a low-depth sample is analyzed, each supporting read will have more weight in the probability of an alternate call, while keeping discordant or unique signals from being reported. Hence, we avoided the need to implement any dynamic thresholds or even a minimal read support parameter.<br><br>4. I would like to try and replicate the results in the paper, but the authors appear to be merging several different samples before down-sampling to the desired coverage value. I don't have an issue with this approach, but at the same time it makes repeating the results problematic. It would be useful for others in the field if a supplementary section was included, giving a series of commands to repeat some of these results, even if only for a single sample.<br><br>R. We added the supplementary file 2 with a thorough guide, including download links to each dataset, to ensure the reproducibility of our benchmarking procedure.
**Additional Information:**	
**Question**	**Response**
Are you submitting this manuscript to a special series or article collection?	No
**Experimental design and statistics**<br><br>Full details of the experimental design and statistical methods used should be given in the Methods section, as detailed in our Minimum Standards Reporting Checklist. Information essential to interpreting the data presented should be made available in the figure legends.<br><br>Have you included all the information requested in your manuscript?	Yes
**Resources**<br><br>A description of all resources used, including antibodies, cell lines, animals and software tools, with enough information to allow them to be uniquely identified, should be included in the Methods section. Authors are strongly encouraged to cite Research Resource Identifiers (RRIDs) for antibodies, model organisms and tools, where possible.<br><br>Have you included the information requested as detailed in our Minimum Standards Reporting Checklist?	Yes

Powered by Editorial Manager® and ProduXion Manager® from Aries Systems Corporation

**Availability of data and materials**<br><br>All datasets and code on which the conclusions of the paper rely must be either included in your submission or deposited in publicly available repositories (where available and ethically appropriate), referencing such data using a unique identifier in the references and in the "Availability of Data and Materials" section of your manuscript.<br><br>Have you have met the above requirement as detailed in our Minimum Standards Reporting Checklist?	Yes

Powered by Editorial Manager® and ProduXion Manager® from Aries Systems Corporation

Manuscript

Click here to access/download;Manuscript;NGSEPSVsLongReads_GigaSci

A graph clustering algorithm for detection and genotyping of structural variants from long reads

Nicolás Gaitán[1], Jorge Duitama[1,*].

[1]Systems and Computing Engineering Department, Universidad de Los Andes, Bogotá, Colombia.

* Corresponding author. E-mail: ja.duitama@uniandes.edu.co

ABSTRACT

Structural variants (SV) are genomic polymorphisms defined by their length (>50 bp). The usual types of SVs are deletions, insertions, translocations, inversions, and copy number variants. SV detection and genotyping is fundamental given the role of SVs in phenomena such as phenotypic variation and evolutionary events. Thus, methods to identify SVs using long-read sequencing data have been recently developed. We present an accurate and efficient algorithm to predict germline SVs from long-read sequencing data. The algorithm starts collecting evidence (Signatures) of SVs from read alignments. Then, signatures are clustered based on a Euclidean graph with coordinates calculated from lengths and genomic positions. Clustering is performed by the DBSCAN algorithm, which provides the advantage of delimiting clusters with high resolution. Clusters are transformed into SVs and a Bayesian model allows to precisely genotype SVs based on their supporting evidence. This algorithm is integrated into the single sample variants detector of the Next Generation Sequencing Experience Platform (NGSEP), which facilitates the integration with other functionalities for genomics analysis. We performed multiple benchmark experiments including simulation, and real data, representing different genome profiles, sequencing technologies (PacBio HiFi, ONT), and read-depths. The results show that our approach outperformed state-of-the-art tools

on germline SV calling and genotyping especially at low depths, and in error-prone repetitive regions. We believe this work significantly contributes to the development of bioinformatic strategies to maximize the use of long-read sequencing technologies.

INTRODUCTION

Structural variants (SV) are a type of genetic polymorphism, in both coding and non-coding sequences, which are usually defined by their length (>50 bp). The main types of SVs are deletions, insertions, translocations, inversions, and copy number variants (Alkan et al., 2011). The main genomic processes that cause the formation of structural variants are DNA recombination, replication, and repair-associated processes (Carvalho et al., 2016). For example, one common mechanism is Non-Allelic Homologous Recombination (NAHR) which is a genetic repair mechanism in which misalignment of previously duplicated regions called low copy repeats (LCR) occurs during meiosis. This subsequently causes a genomic rearrangement event on another locus that does not belong to the LCR gene, thus creating further deletions or duplications (Parks et al., 2015).

The interest in SVs comes mainly from the functional consequences of their genetic diversity. It has been proven that many SVs are involved in different gene expression patterns and influence different characteristics. SVs that are located adjacent to genes may structurally affect *cis*-regulatory regions by position or composition, leading to either silencing or increasing gene expression, which explains variation of Quantitative Trait Loci (QTL)(Chiang et al., 2017). For example, Alonge *et al.* (2020) found that at least 50% of the SVs found in an assessment of around 100 lines of tomato were associated with gene expression regulatory processes, mostly causing reductions or even silencing of gene products. Another case is when duplications increase the amount of overall transcript-protein production by gene dosage effect. This has proven beneficial for artificial selection in certain plant species where the average size

of fruits increased because the plant variant suffered a specific duplication in a cytochrome coding gene (Alonge et al., 2020).

Structural variants also provide fundamental information about evolutionary relationships between organisms and their natural history. Many Whole-Genome Sequencing (WGS) studies have been conducted to assess the prevalence of different SVs and their variation in organisms, populations, or species. In plants, analyzing structural variants allowed elucidation of the dynamics of whole-genome duplication (WGD) events and their evolutionary role (Qiao et al., 2019). WGDs are followed by a fast diploidization process, mainly because most of the duplicated genes become paralogs (Qiao et al., 2019). Furthermore, many components of the C4 metabolic pathway were brought by these WGD events and single duplication events. This is an interesting case of convergence throughout the evolution of different plant lineages (Wang et al., 2009). These changes are influenced by the synergistic effect of WGDs, transposed duplication, and dispersed gene duplication, evidenced by overlapping peaks in the rates of synonymous substitutions (Qiao et al., 2019). This shows how SVs can provide substantial amounts of evidence for evolutionary studies.

Given the importance of SVs, a large number of computational methods have been developed to identify and genotype SVs, based on high throughput sequencing (HTS) data. Most of these SV detection tools are based on short-read sequencing technologies (Cleal et.al., 2022; Sarwal et.al., 2022). This presents many limitations, mostly due to the length of structural variants, which usually exceeds the read length, which reduces the precision of both identification and genotyping (Luan et.al., 2020, Mahmoud et.al., 2019). Recently, new SV calling tools have adopted long reads as their input data, significantly increasing the accuracy of SV detection in comparison with short read-based callers (Mahmoud et.al., 2019; Schwarz et.al., 2021). This has allowed many researchers to increase their catalog of functionally relevant structural variants, including some that affect the pathophysiology of diseases such as human cancer

(Fujimoto et.al., 2021; Thibodeau et.al., 2020). However, further improvements could be achieved by novel algorithmic techniques. Some difficulties arise even when long reads are used. Since SV detection relies on accurate read alignment, dissimilar, partial, or inaccurate read alignments obscure the signal to perform a consistent detection and genotyping of SVs. Thus, the results also depend on the accuracy of the aligner software (Heller et.al., 2019). Additionally, from a software design point of view, our experience indicates that most current tools are difficult to operate because they require a large number of specific libraries and versions, their implementations are not debugged correctly and exceptions are not handled appropriately. For short read-based callers, these limitations have been described by a recent benchmark study by Sarwal et.al (2022).

Benchmarking SV detection is a difficult task. First, there are few independently validated gold standard datasets for real sequencing data because experimental validation is difficult to perform at a large scale. Consequently, there is no consensus on which of the existing tools produces the closest result to a gold standard set. Bolognini et.al (2020) addressed this issue by implementing a simulation software called VISOR, which produces a complete haplotype-resolved sample genome and simulates read alignments from a list of SVs, with either Oxford Nanopore or PacBio error profiles. Trying to optimize the SV calling pipeline, Jiang et.al (2021) evaluated the accuracy of different SV callers using VISOR simulations on real reported human SVs. For the 20x simulated dataset, they report that the best tools are CuteSV (F1=0.8), SVIM (F1=0.798), and Sniffles2 (0.769). Additionally, they provide recommendations for SV calling best practices such as sequencing experiments with read lengths of about 20 kb at 20x depth. Regarding real datasets, the most widely recognized and best-curated case is the high-confidence structural variant dataset (Sample HG002 on reference genome GRCh37) from the Genome In A Bottle human sample project (GIAB) crafted for benchmarking (Zook et.al.,

2020). The events reported in this file come from a mixture of sequencing technologies and have been predicted by using a pipeline integrating many different tools.

The HGSVC consortium also generated high confidence SV calls suitable for benchmarking. In the first version, a haplotype-resolved curated SV callset against the GRCh38 genome was produced for each of three samples from different ethnicities, including Han Chinese, Yoruban Nigerian, and Puerto Rican (HG00514, HG00733, NA19240) respectively. This provides SV variation profiles for individuals with a wide range of genetic diversities, including admixed individuals (Chaisson et.al., 2019). Similar to the GIAB effort, multiple sequencing platforms, and variant calling methods were used to produce these datasets, specially the reference guided assembly of the samples and their parents, which made it possible to determine the haplotype of the SVs. Furthermore, the HGSVC2 version improved these SV calls using *de-novo* assembly with the PAV algorithm (Chaisson et.al., 2019; Ebert et.al., 2021).

Structural variant detection provides the possibility of finding biological insights with many different functional consequences. In this manuscript, we developed a new software solution that improves the detection of germline SVs from long-read alignments using the DBSCAN algorithm to solve the clustering problem, and implements a new bayesian genotyping model. This functionality is integrated into the bioinformatic software suite (NGSEP) to further facilitate the analysis of genomic data.

RESULTS

A new clustering algorithm for detection and genotyping of Structural Variants

The process of structural variant detection and genotyping starts from reads aligned to a reference genome and is divided into three main stages described as follows.

1. Signature Collection

The main input to this algorithm is a set of read alignments in SAM or BAM format, obtained from mapping long reads to a reference genome. Signatures are individual signals of a structural variant that are contained within each read alignment or constructed from discordant partial alignments. They can be divided into intra-alignment and inter-alignment signatures. Intra-alignment signatures consist of evidence of deletions or insertions that are predicted as part of the read alignment process. Thus, these signatures are collected by reading the description of the alignment (encoded in the CIGAR field of the SAM format) to find signals of insertion or deletion. Conversely, reads with multiple discordant alignment segments, regarding their position or orientation, are selected to identify inter-alignment signatures.

Figure 1 shows the procedures that we implemented for the recollection of signatures for each SV type. Intra-alignment deletions and insertions are identified by parsing the CIGAR strings, and searching for their codes (e.g. D or I, respectively), The CIGAR code includes the length of each event within the alignment. Inter-alignment deletions are suspected when unmapped regions in the reference genome are flanked by partial alignments. For each read with two partial alignments within the same chromosome region, the reference distance between the end of the first partial alignment and the beginning of the second alignment in reference genomic coordinates is considered the length of the deletion signature. Inter-alignment insertion signatures are identified from reads with two adjacent alignments, having a soft clip starting from the presumed insertion point. For each read, the Longest Soft Clip (LSC) is calculated by taking the maximum of soft clips at the end of each alignment. The length of the partial alignment that does not contain the LSC is subtracted from the length of the LSC to estimate the length of the insertion signature. Inversions appear as three consecutive partial alignments where the middle alignment has an opposite orientation, compared to the two flanking alignments. The length of the inversion is predicted as the length of the middle alignment.

Signatures are filtered from the minimum SV length specified by the user (default ≥50 bp) and are added to a collection, which is sorted by chromosome and reference coordinates.

2. *Signature Clustering*

Given a set of SV signatures, we implemented a graph-based clustering in which each cluster becomes a candidate SV event. A graph is built independently for each signature type. The vertices of the graph correspond to the input collection of signatures identified in the previous step. Each signature is represented by a tridimensional vector with three numeric values: Start coordinate in the reference genome (B_i) end coordinate in the reference genome (E_i), and signature length (L_i). The cost m_{ij} of the edge between two signatures i and j corresponds to the Euclidean distance of their corresponding vectors:

$$FPD_{ij} = |B_j - B_i| \qquad LPD_{ij} = |E_j - E_i| \qquad LD_{ij} = |L_j - L_i|$$

$$m_{ij} = \sqrt{FPD_{ij}^2 + LPD_{ij}^2 + LD_{ij}^2}$$

The DBSCAN algorithm is a non-supervised clustering procedure for n-dimensional vectors (points) based on the principle of density-based grouping (Schubert et.al., 2017). The parameters of this algorithm are a threshold *epsilon* (ε) which limits the distance for considering two points as neighbors, and a minimum number of neighbors (minPts) that a point should have to be considered a *core point*. The lemma states that considering a cluster that contains certain *core points*, then any point which is density reachable from any of those *core points* (in the graph context, any point that has a path from any *core point*) will be considered as part of the cluster. Any point that is not reachable from any *core point* will be considered a noise signal. The procedure to implement this algorithm was as follows. Starting from an initially complete graph with *n* points, the algorithm eliminates the edges where m_{ij} is bigger than or equal to ε. Then, each point is visited to test if its number of neighbors is at least *minPts*, in which case it is labeled as a core point. Consequently, a new cluster is initialized with the core point and its

direct neighbors, and a Breadth First Search (BFS) is performed by pushing this neighborhood into a queue where each point will also be queried for its neighbors to assess the *core point* property presumption, repeating this process until all of the density reachable points from any core point in the cluster are visited. If there are unvisited points, the procedure continues until all points are visited. Figure 2 shows the main steps and restrictions of this procedure.

3. *Cluster to Genotyped SV*

Each signature cluster identified in the previous step becomes a candidate SV. The last step of the process is the genotyping of these candidates. To identify SV coordinates, the average of the first reference coordinates of the signatures within the cluster is estimated. The last coordinate is calculated likewise. The length is taken as the difference between both the last and first SV coordinates, except for insertions where the average length of the cluster signatures is estimated as the average of the insertion lengths of the signatures. Candidate SVs are stored in a collection sorted by reference coordinates. Then, a Bayesian genotyping process is performed for each candidate SV by reassessing the evidence that read alignments provide. To avoid having to reprocess the alignments file, a collection of compact alignment objects is kept in memory from the first stage, having the minimum possible information needed for this step. For each SV, intersecting read alignments are collected, and those containing clustered signatures are considered supporting evidence for the alternative allele hypothesis. If the spanning read alignment contains no signatures, it is counted as a supporting call for the reference allele. Figure 3 shows the estimation of the likelihood for the four possible scenarios, generated from the combination of the hypotheses, the two plausible alleles from which the read could be sequenced (SV or REF alleles), with calls from a read alignment that may or may not support these allele hypotheses. The distribution of lengths of the clustered signatures supporting the SV hypothesis is used to estimate the likelihood of a read alignment supporting this SV. In this case, it is assumed that the read was actually sequenced from a chromosome

affected by the SV (case 1). If a reference allele is assumed (case 2), a read with an SV signature is proposed to have happened by a misalignment or sequencing error and a fixed value (0.0001 by default) is used as likelihood. The likelihood of a read supporting the reference allele that is assumed to be sequenced from a haplotype affected by the SV is calculated as the probability of having an indel error that reverts the SV and is also a constant value (0.001 by default) (case 3). Finally, a fixed value (default 0.999) is used for the likelihood of a read supporting the reference allele assuming sequencing from a reference haplotype.

Read likelihoods for each allele hypothesis are transformed into posterior probabilities for each possible genotype following the same procedure implemented in NGSEP to perform SNP genotyping (Gil et al., 2021). The hypothesis having the largest posterior probability is assigned as the predicted genotype. If the genotype of an SV call is assigned as homozygous reference (0/0), this call will be considered as not well supported, and it will be filtered out of the output. Similar to SNP genotyping, the quality of such SV calls will be the phred score Q corresponding to the genotype posterior probability. Finally, duplications are identified after the three main steps from genotyped insertion SVs, if the supporting intra-alignment signatures differ significantly in reference coordinates.

Benchmarking with simulation experiments

We performed two simulations of structural variants in the genome of *Arabidopsis thaliana* using the tool VISOR (Bolognini et.al., 2020). 1718 insertions, 2532 deletions, and 2065 inversions were generated for benchmark experiments. Read alignment subsets were produced for 20x, 30x, 45x, and 60x. The precision-recall results of our NGSEP algorithm were compared to those of state-of-the-art tools, including SVIM (version 2.0.0) (Heller et.al., 2019), Sniffles2 (version 2.2) (Sedlazeck et.al., 2018; Smolka et.al., 2022), CuteSV (version 2.0.3) (Jiang et.al., 2020) and Dysgu (version 1.6.1) (Cleal et.al., 2022). After obtaining the metrics

for both simulation experiments, precision-recall curves and F-score against depth were plotted for each tool. Additionally, execution times for each depth dataset were evaluated for single-thread runs.

Figure 4A shows that the NGSEP algorithm presented above achieves an F-score value over 99, outperforming SVIM, Dysgu and Sniffles2 for all depths. (Values available at the Supplementary File 1). Only CuteSV achieves similar scores, reaching 99.5 in the 45x dataset. Figure 4B shows that NGSEP and CuteSV keep high performance for varying alignment depths in both precision and recall. In the inversion simulation benchmark, SVIM produced the highest F-score, closely followed by NGSEP and Dysgu (Figure 4C). The three tools showed almost perfect precision and between 50% and 60% recall. Conversely, CuteSV showed a precision of only 50% and Sniffles2 failed to detect most inversions.

Additionally, we generated a simulated SV gold-standard from the Human T2T genome (Nurk et.al., 2022) following the same pipeline, and increasing the amount of depth samples to 5x, 10x, 20x, 30x, 40x, and 60x including 5,000 insertions and 5,000 deletions, to evaluate the performance of the algorithms with bigger input data sizes and different genome features.

Results from these simulations are similar to those obtained with the Arabidopsis genome (Supplementary File 1). Our algorithm also generated good precision and recall values in this simulation. Comparing the results of the different tools, the outcome was similar to that obtained with Arabidopsis, with the exception of the F-scores of Sniffles2, which were the best in this simulation. NGSEP had slightly larger F-scores than CuteSV in the 10x and the 60x datasets (Supplementary Figure 1).

Single-thread runtimes were recorded for all experiments to compare the tools in terms of computational efficiency. As shown in Figure 4D, all of them follow a linearly increasing trend. Sniffles2 and NGSEP consistently required lower execution times compared to the other tools. It is worth clarifying that Sniffles2, CuteSV, and Dysgu support multithreading which

significantly reduces runtimes at the cost of processing resources. Dysgu was the worst-performing tool in terms of computational efficiency, requiring about three times more execution time than the NGSEP algorithm. For the T2T dataset, Dysgu ran faster than NGSEP, and CuteSV had the highest execution runtime (Supplementary figure 1).

Benchmarking with the Genome In a Bottle human genome

To assess the performance of our method on real datasets, we performed multiple experiments using reads from the Genome In a Bottle (GIAB) human individual HG002, for which a gold standard set of large indel calls is publicly available. Both 56x PacBio HiFi CCS (Circular Consensus Sequencing), and 47x ONT UL (Ultra Long Reads) reads sequenced from the HG002 subject were randomly sampled at average depths of 10x, 20x, 30x, and 40x to perform different experiments. Truvari (English et.al., 2022) was used to obtain precision and recall metrics of test calls against the gold standard, which was restricted either to the Tier 1 plus Tier 2 (T1+2) regions, or just Tier 1 (T1), and PASS-only SVs. Additionally, a F-score variation called GTF-score was estimated to assess their performance regarding the combination of genotyping accuracy and recall. Further details for this metric are provided in the methods section.

Figure 5A shows the results of the benchmark experiments detecting variants from PacBio HiFi alignments, using as gold-standard the T1+2 dataset, and varying read depth from 5x to 56x. Dysgu provided the best F-Score for low-depth mappings, closely followed by NGSEP (5,10 and 20x, see exact values in the Supplementary file 1). CuteSV had the best precision, but the worst recall. Similar to the simulation results, at increasing depths SVIM increased recall at a high cost on precision. Regarding GTF-Score, Dysgu produced the highest values. CuteSV produced the highest GT accuracy, but the low recall reduced its GTF-score (Figure 5B). The precision for all tools is low (up to 65%) mainly because the Tier 2 includes highly repetitive

regions in the human genome. If the gold standard is restricted to the T1 dataset, all tools improve precision, reaching values over 90% in almost all cases (Supplementary figure 2). Sniffles2 shows the most important increase in this comparison, reaching precision values slightly larger than those of NGSEP for depths above 30x.

Regarding ONT aligned reads and comparing against the T1+2 dataset, NGSEP is the most accurate tool comparing precision and recall. CuteSV, Sniffles2, and Dysgu achieve better recall and genotyping accuracy than NGSEP, at the cost of precision. Restricting the comparison to T1 regions, precision increases for all tools and Sniffles2 becomes the tool with the highest F-Score overall (Supplementary figure 2). The behavior of all callers is relatively consistent with the HiFi data, but the values obtained for the different metrics are consistently lower, probably due to the higher error rate of ONT reads.

A breakdown of these results in both deletion and insertion categories, shows that the major improvements in performance metrics for NGSEP over the other tools comes from the accurate detection of insertions, especially for the ONT data. However, the low GT accuracy of NGSEP for ONT reads is caused by a GT accuracy of insertions below 80% (Supplementary figures 3 and 4). Consistent with the global results, the improved precision of NGSEP is not evident if only T1 regions are included in the benchmark experiments (Supplementary figures 5 and 6).

We also tried to include in the benchmark experiments the tool PBSV (version 2.9.0)(https://github.com/PacificBiosciences/pbsv). However, this tool did not work with the original alignments, and hence we had to realign the HiFi reads with the pbmm2 mapper (available with PBSV). PBSV produced low recall values at low depths, improving as depth increases at a cost on precision. The overall accuracy of PBSV was inferior to that of NGSEP, both for the complete gold standard dataset and for the subset of Tier 1 SVs (Supplementary Figure 7). Both NGSEP and CuteSV benefited from improved accuracy using these realigned

reads. NGSEP became the tool with the best F-score for low read depths (below 20x), whereas CuteSV reported the best metrics above 20x.

Finally, the runtime of each tool behaves similar to the simulations. Sniffles2 was the fastest tool for all subsets, and PBSV was the slowest tool in most cases (Supplementary Figure 8A). We also analyzed the peak memory consumption for our algorithm. NGSEP takes less than 8Gb of RAM heap space to analyze the datasets up to 30x. For bigger inputs, although more space is used by the Java Virtual Machine, new objects maintain low memory consumption (Supplementary Figure 8B). All experiments could be performed with up to 16 Gb of RAM.

Benchmarking with the HGSVC2 samples

Taking advantage of the efforts made by the HGSVC consortium to produce accurate SV callsets, we included their three most refined samples (HG00514, HG00733, NA19240) into our benchmark experiments. These resulted in a truth set consisting of 74,467 indel SVs. The breakdown per sample and SV type is available in the Supplementary table 1. To evaluate the quality of SV callers for low-depth and varying genetic diversity inputs, we aligned PacBio HiFi sequencing reads from each of these samples to the GRCh38 genome, using minimap2 (Li et.al., 2018). Then, we randomly subsampled the mappings to evaluate the tools at 20x depth.

Figure 6 shows the performance metrics for SV discovery on the three samples. NGSEP achieved the second best F-score after CuteSV and the second best GTF-score after SVIM (All values are available at the Supplementary file 1). Dysgu and SVIM reported very low precision values, although they identified more than 75% of the indels. Conversely, CuteSV has high precision and genotyping accuracy, but it had between 2% and 5% less recall than NGSEP. In this experiment, the calls generated by Sniffles2 had surprisingly low performance metrics, taking into account the performance observed in the simulations and the GIAB data. After

manual inspection of the results, we discovered that Sniffles2 was reporting SVs in locations consistent with the gold-standards, but the reported SV length was about two times the SV length of the gold standard. Relaxing the reciprocal overlap for test-reference allele lengths (See methods for details), the precision and recall metrics of SVs reported by Sniffles2 improved to values similar to those observed in the previous experiments. However, the GT-accuracy is still affected, increasing only up to 45% (Supplementary Figure 9). Consistent with the experiments with the GIAB dataset, restricting the gold-standards to non-repetitive regions increases the performance of all callers (Supplementary Figures 10 and 11). In particular, NGSEP achieves the best genotyping accuracy for the admixed Puerto Rican individual (NA19240), suggesting that our genotyping procedure is very accurate even for samples with high heterozygosity (Figure 6C, Supplementary Figures 9C,10C and 11C).

DISCUSSION

The availability of long-read sequencing technologies represented a big step forward toward the accurate identification and genotyping of structural variants (Fujimoto et.al., 2021; Thibodeau et.al., 2020). Achieving this goal is becoming a requirement for current genomics, given the documented role of SVs as drivers of phenotyping variability and evolution (Alonge et al., 2020; Gorkovskiy et.al., 2021; Qiao et al., 2019; Wang et al., 2009). In this work we present the results of our efforts to develop novel algorithmic techniques, aiming to increase the accuracy of both discovery and genotyping of germline SVs. Transforming the problem of clustering SV signatures into a geometric clustering problem in an Euclidean space, allowed us to build a solution based on the well-known DBSCAN clustering algorithm (Schubert et.al., 2017) to identify SVs. A similar Euclidean space representation is implemented in the Jasmine algorithm to merge SVs from different samples into a refined call, which improved population level analyses (Kirsche et.al., 2023). Even though both works differ in the clustering algorithm,

they demonstrate the advantages of representing SV signals as n-dimensional euclidean points, and provide the groundwork for future SV analysis algorithms. Previous experiences implementing Bayesian models for SNV genotyping, allowed us to increase the accuracy of SV identification and provided a framework for SV genotyping.

Benchmarking experiments running simulations, and analyzing real data with the GIAB, and HGSVC2 datasets indicate that our algorithm achieves competitive accuracy compared to current software solutions. NGSEP consistently provided top-tier performance metrics across the experiments, showing a great balance between recall and precision for a variety of samples. The observed differences in the results obtained with Tier 1 and Tier 1+2 regions indicate that our solution provides accurate calls in repetitive regions, which remains as one of the main challenges for SV calling efforts. Compared to SVIM, our algorithm provided consistently better accuracy in all experiments. SVIM in particular ranked last in performance for ONT data. This result is consistent with previous experiments (Cleal et al., 2022) and could be explained by the tendency of the hierarchical clustering implemented in SVIM to separate signatures coming from the same variant if there is high variability in alignments due to sequencing error rates. Regarding Sniffles2, this tool was very competitive, achieving in some cases superior discovery and genotyping accuracy, compared to NGSEP, both in the simulations and in the experiments with the GIAB benchmark dataset. However, this behavior was not consistent in our experiments with the HGSVC2 datasets, mainly because in these cases Sniffles2 produced calls with about two times the length of the real calls. We could not identify a rationale for this behavior. Regarding CuteSV, we obtained SV calls with good accuracy running this tool, but only if the minimum read depth was set to 5x and if the testing read depth was superior to 20x. CuteSV implements a two step clustering procedure, making initial clusters based on coordinates and then identifying subclusters based on differences in event length. This process is controlled by a set of parameters which need to be tuned for

different event types and sequencing technologies. Although we acknowledge that further testing of each tool with different parameters on each specific dataset could yield improved outcomes, we argue that this indicates that our method adapts more naturally to changes in read depths and sequencing technologies, reducing the effort to perform parameter tuning for each experiment. Finally, Dysgu had the best F-score for HiFi reads of the GIAB dataset, but this outcome was not consistent testing other benchmark datasets. Since the initial submission of this paper we observed an important increase in accuracy for new versions of this tool, suggesting that the underlying algorithm is under active development. Continuous improvements are likely to be developed for all tools, including NGSEP and even including benchmark tools such as Truvari. This means that the current benchmark is only a snapshot of the current status of this field.

Given that even using long reads it is not easy to identify and cluster signatures for translocations, compared to other SV types, our current solution does not support discovery of translocations. We expect to implement this feature in future versions of NGSEP. We also plan to further improve on genotyping accuracy in future versions of the algorithm.

Researchers performing population genomic studies usually trade read depth by the number of samples sequenced, looking for a balance that maximizes the cost-benefit of the sequencing effort (Cericola et.al., 2018; Fumagalli et.al., 2013). Thus, it is extremely important for SV detection tools to be able to produce accurate results from a low-depth input. One of the biggest advantages of the NGSEP algorithm, when compared to the other state-of-the-art tools, is that it is robust to reductions of read depth. Even at 20x average read depth, the integration of the Bayesian model provided the best results for genotyping accuracy in the HGSVC2 experiments, also demonstrating reliability for samples with different genetic diversity profiles. Additionally, this probabilistic model improved precision, which was evidenced by the analysis of the 47x ONT GIAB reads, which have a bigger error rate than CCS reads. This

suggests that our algorithm is also robust to increased per-base error rates. Beyond tools comparison, our experiments indicate that an average read depth of around 20x is sufficient to achieve high detection and genotyping accuracy.

We believe that this work represents a significant contribution to current research on algorithms to analyze long DNA sequencing reads. We expect that the new functionality developed in NGSEP for SV detection from long reads will be useful for a large number of ongoing and upcoming research in population genomics for different species.

METHODS

Software development and integration within NGSEP

The algorithm described in this manuscript was implemented in Java 11 as a new option of the single sample variants detector functionality of the NGSEP software tool. The reuse of different NGSEP classes significantly decreased the development effort needed to code. Initially, for computing the input file a ReadAlignment iterator found in the ReadAlignmentFileReader class was used, given that it already collects all of the necessary information for each alignment. A Collection interface class named GenomicRegionSortedCollection allowed GenomicVariant interface implementing objects, such as Signature and CalledGenomicVariant objects, to be stored by sorted sequence, e.g chromosomes, and by genomic position. This also facilitated computed spanning alignments to specific variants. Additionally, the work made for genotyping SVs consisted mostly of programming the functionality to estimate likelihoods, given that the class CountsHelper allowed calculating the genotype posterior probabilities, as it was implemented before to genotype small indels and SNPs. The class diagram for the functionality inside of the NGSEP class context is shown in the supplementary figure 12.

Simulation experiments

In order to assess the behavior of our algorithm to identify and genotype SVs, a thorough benchmarking process was established to evaluate performance metrics of recall, precision, and efficiency. After an in-depth literature revision, four tools were included in the benchmark based on their performance and impact, including SVIM (version 2.0.0) (Heller et.al., 2019), Sniffles2 (version 2.2) (Sedlazeck et.al., 2018), CuteSV (version 2.0.3) (Jiang et.al., 2020) and Dysgu (version 1.6.1) (Cleal et.al., 2022). Both simulations and real cases were used to perform benchmark experiments. Output VCF files with SV calls were compared to Gold Standard files using the software Truvari (version 4.1.0) (English et.al., 2022), which provides recall, precision, F-score, and genotype accuracy of the evaluated SV genotype calls. This tool has been recommended by the GIAB consortium for benchmarking of SV callers (Zook et.al., 2020). Parameters for each dataset are provided in the supplementary table 2. In particular, we reduced the minimum read support parameter of CuteSV to 5x after performing parameter tuning experiments on the GIAB datasets (Supplementary figure 13).

SVs were simulated with the software VISOR (Bolognini et.al., 2020), based on the *Arabidopsis thaliana TAIR10* reference genome (Lamesch et.al., 2012). A total of 4330 structural variants with a minimum length of 50 bp were simulated (2500 deletions, 1830 insertions, and 2065 inversions), and a genome containing these variants was generated. Next, reads with the characteristics of the Oxford Nanopore Sequencing Technology (ONT), including the error profile, were simulated with VISOR from this altered genome. Reads were aligned to the original reference genome using minimap2 (Li et.al., 2018). This pipeline was repeated to simulate four datasets of varying depths, including 20x, 30x, 45x, and 60x. The resulting alignments were used as the input data for all tools. The Human simulation from the T2T genome (Nurk et.al., 2022) was produced following the exact same pipeline.

GIAB high-confidence dataset

The Genome in a Bottle (GIAB) consortium has produced a high-confidence curated SV dataset, consisting of indel SVs identified from many biotechnologies, and multiple bioinformatic methods on the Ashkenazi son sample (HG002) against the GRCh37 reference genome (Zook et.al., 2020). All callers, including NGSEP, were used to discover SVs from a PacBio HiFi read alignment dataset of 56x depth and an ONT UL dataset of 47x depth, both sequenced from the same HG002 subject. Minimap2 (Li et.al., 2018) was used as the mapping tool to the GRCh37 reference genome. These alignments were randomly subsetted to produce 10x, 20x, 30x, and 40x input files in addition to the initial full-depth datasets, to assess the effect of depth variance on the calling algorithms. In order to include PBSV (version 2.9.0) (https://github.com/PacificBiosciences/pbsv) we had to realign reads from the original HiFi HG002 sample using the pbmm2 mapper (available with PBSV). See step-by-step instructions in the supplementary file 2.

From the GIAB gold standard, we used two ground-truth benchmark datasets, one including repetitive regions called Tier 1+2 (T1+2), and another retaining only non-repetitive regions, called Tier 1 (T1). We filtered these datasets retaining only SVs flagged with a “PASS” in the filter field of the VCF files, and having length larger than 50 bp. The final number of SVs for each experiment can be found in the Supplementary table 1.

HGSVC2 high-confidence samples

The work made by the HGSVC2 consortium provided high-confidence haplotype resolved calls for three samples of different ethnicities against the GRCh38 genome (Chaisson et.al., 2019; Ebert et.al., 2021). The supplementary table 1 shows the number of SVs of each type within each gold-standard dataset. PacBio HiFi reads were extracted from publicly available alignments for each of the three samples and were realigned with minimap2 (Li et.al., 2018).

From each one, a 20x depth set of randomly chosen alignments was produced as input for the aforementioned callers. See step-by-step instructions in the supplementary file 2.

For benchmarking using Truvari, we compared the results obtained keeping the default value of reciprocal overlap (70%) with those obtained reducing this parameter to 35% (-pct flag). We adjusted this parameter based on the initial results produced by Sniffles2. Similar to the experiments with the GIAB dataset, we also calculated the metrics using the complete dataset, and compared them with those obtained including only SVs in non repetitive regions of the reference genome.

Benchmark metrics

Truvari (English et.al., 2022) was used to produce the benchmark metrics, using symbolic alleles only. Performance metric calculation is specified as follows:

$$Precision = \frac{TP}{TP+FP} \qquad Recall = \frac{TP}{TP+FN}$$

$$GTAccuracy = \frac{HOM_{TP}^{HOM} + HET_{TP}^{HET}}{HOM_{TP}^{HOM} + HOM_{TP}^{HET} + HET_{TP}^{HET} + HET_{TP}^{HOM}}$$

$$Fscore = 2\frac{Precision \times Recall}{Precision + Recall} \qquad GTFscore = 2\frac{GTAccuracy \times Recall}{GTAccuracy + Recall}$$

Where GT-Accuracy is a metric obtained by estimating the fraction of the correctly genotyped true positive SVs over the total amount of true positives. Superscripts indicate their true genotype, which may differ from the caller classification. GTF-score is a variation of F-score, to combine correct genotype classification with recall as the harmonic mean between both values.

Truvari also allows the inclusion of SVs in the truth and test call sets if they are located inside the genomic regions annotated in an input bed. This allowed us to produce the separate Tier1 and Tier1+2 benchmarks for GIAB and the non-repetitive-regions and all-regions for

HGSVC2. Finally, this software does not take into account SVs with homozygous reference (0/0) genotype calls.

Execution environments

Arabidopsis simulation software executions including running all SV callers were done on an 8-core Ryzen 7 5800H with 16Gb RAM Laptop. Analysis of the human T2T simulation, the GIAB benchmark and the HGSVC2 benchmark, was performed on an Intel Xeon Gold computing node with a capacity of 42 threads and 565 GB RAM. Most of this computing power was required to align reads to the reference genomes. Processes for variants detection were restricted to a single core and 16Gb of RAM.

ACKNOWLEDGEMENTS AND FUNDING

This work has been supported by the "Patrimonio autónomo del Fondo Nacional de Financiamiento para la ciencia, la tecnología y la innovación Francisco José de Caldas" with the contract number 80740-441-2020, awarded by the Colombian Ministry of Science to JD. We also acknowledge the high-performance computing unit of Universidad de Los Andes for their technical support to conduct the benchmark experiments presented in this manuscript.

DATA AVAILABILITY

The *A. thaliana* TAIR10 reference genome used for simulations is available in the phytozome v.12 database (https://phytozome-next.jgi.doe.gov). The GIAB SV gold standard VCF file can be downloaded from the GIAB website (https://www.nist.gov/programs-projects/genome-bottle) as well as the bed files containing tier information. The GHC37 human reference genome can be found in the NCBI Assembly database (accession number GCA_000001405.1).

PacBio HiFi reads are available at SRA BioProject accession number PRJNA586863. Oxford nanopore reads are located at the European Nucleotide Archive (ENA) under accession PRJEB37264.

Assets for the HGSVC2 benchmark are found in the project page at https://www.internationalgenome.org/data-portal/data-collection/hgsvc2. PB HiFi read files for the three samples are listed in this website, and deposited in the EBI ftp site at ftp://ftp.sra.ebi.ac.uk. Specifically, the GRCH38 reference genome can be found at http://ftp.1000genomes.ebi.ac.uk/vol1/ftp/data_collections/HGSVC2/technical/reference/20200513_hg38_NoALT/hg38.no_alt.fa.gz, and the vcf which contains the gold standard SVs for the three samples is available at http://ftp.1000genomes.ebi.ac.uk/vol1/ftp/data_collections/HGSVC2/release/v2.0/integrated_callset/variants_freeze4_sv_insdel_alt.vcf.gz.

SOFTWARE AVAILABILITY

The algorithm presented in this study can be executed through the Single sample Variants Detector functionality of the open-source software Next Generation Sequencing Experience Platform (NGSEP). Releases of NGSEP are available at SourceForge (http://ngsep.sf.net). Life development is available on Git Hub (https://github.com/NGSEP). These are full details of the availability of supporting source code and requirements:

Project name: Next Generation Sequencing Experience Platform (NGSEP)

Project home page: http://ngsep.sf.net

Operating system(s): Platform independent

Programming language: Java

Other requirements: Java 11 or higher

License: GNU GPL

RRID: SCR_012827

Biotools ID: NGSEP

COMPETING INTEREST STATEMENT

The authors declare that there are no competing interests related to the publication of this manuscript.

REFERENCES

Alkan, C., Coe, B. P., & Eichler, E. E. (2011). Genome structural variation discovery and genotyping. *Nature Reviews Genetics*, *12*(5), 363-376.

Alonge, M., Wang, X., Benoit, M., Soyk, S., Pereira, L., Zhang, L., ... & Lippman, Z. B. (2020). Major impacts of widespread structural variation on gene expression and crop improvement in tomato. *Cell*, *182*(1), 145-161. http://doi.org/10.1016/j.cell.2020.05.021

Bolognini, D., Sanders, A., Korbel, J. O., Magi, A., Benes, V., & Rausch, T. (2020). VISOR: a versatile haplotype-aware structural variant simulator for short-and long-read sequencing. *Bioinformatics*, *36*(4), 1267-1269. https://doi.org/10.1093/bioinformatics/btz719

Carvalho, C. M., & Lupski, J. R. (2016). Mechanisms underlying structural variant formation in genomic disorders. *Nature Reviews Genetics*, *17*(4), 224-238. https://doi.org/10.1038/nrg.2015.25

Cericola, F., Lenk, I., Fè, D., Byrne, S., Jensen, C. S., Pedersen, M. G., ... & Janss, L. (2018). Optimized use of low-depth genotyping-by-sequencing for genomic prediction among multi-parental family pools and single plants in perennial ryegrass (Lolium perenne L.). *Frontiers in plant science*, *9*, 369. https://doi.org/10.3389/fpls.2018.00369

Chaisson, M. J., Sanders, A. D., Zhao, X., Malhotra, A., Porubsky, D., Rausch, T., ... & Lee, C. (2019). Multi-platform discovery of haplotype-resolved structural variation in human genomes. *Nature communications*, *10*(1), 1784. https://doi.org/10.1038/s41467-018-08148-z

Chiang, C., Scott, A. J., Davis, J. R., Tsang, E. K., Li, X., Kim, Y., ... & Hall, I. M. (2017). The impact of structural variation on human gene expression. *Nature genetics*, *49*(5), 692-699. https://doi.org/10.1038/ng.3834

Cleal, K., & Baird, D. (2022). Dysgu: efficient structural variant calling using short or long reads. *Nucleic Acids Research* 50(9): e53. https://doi.org/10.1093/nar/gkac039

Ebert, P., Audano, P. A., Zhu, Q., Rodriguez-Martin, B., Porubsky, D., Bonder, M. J., ... & Eichler, E. E. (2021). Haplotype-resolved diverse human genomes and integrated analysis of structural variation. *Science*, *372*(6537), eabf7117. https://doi.org/10.1126/science.abf7117

English, A. C., Menon, V. K., Gibbs, R., Metcalf, G. A., & Sedlazeck, F. J. (2022). Truvari: Refined structural variant comparison preserves allelic diversity. *bioRxiv*. https://doi.org/10.1101/2022.02.21.481353

Fujimoto, A., Wong, J. H., Yoshii, Y., Akiyama, S., Tanaka, A., Yagi, H., ... & Shimada, M. (2021). Whole-genome sequencing with long reads reveals complex structure and origin of structural variation in human genetic variations and somatic mutations in cancer. *Genome medicine*, *13*(1), 1-15. https://doi.org/10.1186/s13073-021-00883-1

Fumagalli, M. (2013). Assessing the effect of sequencing depth and sample size in population genetics inferences. *PloS one*, *8*(11), e79667. https://doi.org/10.1371/journal.pone.0079667

Gil J, Andrade-Martínez JS and Duitama J (2021) Accurate, Efficient and User-Friendly Mutation Calling and Sample Identification for TILLING Experiments. Front. Genet. 12:624513. doi: 10.3389/fgene.2021.624513

Gorkovskiy, A., & Verstrepen, K. J. (2021). The Role of Structural Variation in Adaptation and Evolution of Yeast and Other Fungi. *Genes*, *12*(5), 699. http://doi.org/10.3390/genes12050699

Heller, D., & Vingron, M. (2019). SVIM: structural variant identification using mapped long reads. *Bioinformatics*, *35*(17), 2907-2915. https://doi.org/10.1093/bioinformatics/btz041

Kirsche, M., Prabhu, G., Sherman, R., Ni, B., Battle, A., Aganezov, S., & Schatz, M. C. (2023). Jasmine and Iris: population-scale structural variant comparison and analysis. *Nature Methods*, *20*(3), 408-417. https://doi.org/10.1038/s41592-022-01753-3

Jiang, T., Liu, S., Cao, S., Liu, Y., Cui, Z., Wang, Y., & Guo, H. (2021). Long-read sequencing settings for efficient structural variation detection based on comprehensive evaluation. *BMC bioinformatics*, *22*(1), 1-17. https://doi.org/10.1186/s12859-021-04422-y

Jiang, T., Liu, Y., Jiang, Y., Li, J., Gao, Y., Cui, Z., ... & Wang, Y. (2020). Long-read-based human genomic structural variation detection with cuteSV. *Genome biology*, *21*(1), 1-24. https://doi.org/10.1186/s13059-020-02107-y

Lamesch, P., Berardini, T. Z., Li, D., Swarbreck, D., Wilks, C., Sasidharan, R., ... & Huala, E. (2012). The Arabidopsis Information Resource (TAIR): improved gene annotation and new tools. *Nucleic acids research*, *40*(D1), D1202-D1210. http://doi.org/10.1093/nar/gkr1090

Li, H. (2018). Minimap2: pairwise alignment for nucleotide sequences. *Bioinformatics*, *34*(18), 3094-3100. https://doi.org/10.1093/bioinformatics/bty191

Luan, M. W., Zhang, X. M., Zhu, Z. B., Chen, Y., & Xie, S. Q. (2020). Evaluating structural variation detection tools for long-read sequencing datasets in saccharomyces cerevisiae. *Frontiers in genetics*, *11*, 159. http://doi.org/10.3389/fgene.2020.00159

Mahmoud, M., Gobet, N., Cruz-Dávalos, D. I., Mounier, N., Dessimoz, C., & Sedlazeck, F. J. (2019). Structural variant calling: the long and the short of it. *Genome biology*, *20*(1), 1-14. https://doi.org/10.1186/s13059-019-1828-7

Nurk, S., Koren, S., Rhie, A., Rautiainen, M., Bzikadze, A. V., Mikheenko, A., ... & Phillippy, A. M. (2022). The complete sequence of a human genome. *Science*, *376*(6588), 44-53. https://doi.org/10.1126/science.abj6987

Parks, M. M., Lawrence, C. E., & Raphael, B. J. (2015). Detecting non-allelic homologous recombination from high-throughput sequencing data. *Genome biology*, *16*(1), 1-19. https://doi.org/10.1186/s13059-015-0633-1

Qiao, X., Li, Q., Yin, H., Qi, K., Li, L., Wang, R., ... & Paterson, A. H. (2019). Gene duplication and evolution in recurring polyploidization–diploidization cycles in plants. *Genome biology*, *20*(1), 1-23. https://doi.org/10.1186/s13059-019-1650-2

Schubert, E., Sander, J., Ester, M., Kriegel, H. P., & Xu, X. (2017). DBSCAN revisited, revisited: why and how you should (still) use DBSCAN. *ACM Transactions on Database Systems (TODS)*, *42*(3), 1-21. https://doi.org/10.1145/3068335

Sarwal, V., Niehus, S., Ayyala, R., Kim, M., Sarkar, A., Chang, S., ... & Mangul, S. (2022). A comprehensive benchmarking of WGS-based deletion structural variant callers. *Briefings in Bioinformatics*, *23*(4), bbac221. http://doi.org/10.1093/bib/bbac221

Schwarz, J. M., Lüpken, R., Seelow, D., & Kehr, B. (2021). Novel sequencing technologies and bioinformatic tools for deciphering the non-coding genome. *Medizinische Genetik*, *33*(2), 133-145. https://doi.org/10.1515/medgen-2021-2072

Sedlazeck, F. J., Rescheneder, P., Smolka, M., Fang, H., Nattestad, M., Von Haeseler, A., & Schatz, M. C. (2018). Accurate detection of complex structural variations using single-molecule sequencing. *Nature methods*, *15*(6), 461-468. https://doi.org/10.1038/s41592-018-0001-7

Smolka, M., Paulin, L. F., Grochowski, C. M., Mahmoud, M., Behera, S., Gandhi, M., ... & Sedlazeck, F. J. (2022). Comprehensive structural variant detection: from mosaic to population-level. BioRxiv, 2022-04.

Thibodeau, M. L., O'Neill, K., Dixon, K., Reisle, C., Mungall, K. L., Krzywinski, M., ... & Jones, S. J. (2020). Improved structural variant interpretation for hereditary cancer susceptibility using long-read sequencing. *Genetics in Medicine, 22*(11), 1892-1897. https://doi.org/10.1038/s41436-020-0880-8

Wang, X., Gowik, U., Tang, H., Bowers, J. E., Westhoff, P., & Paterson, A. H. (2009). Comparative genomic analysis of C4 photosynthetic pathway evolution in grasses. *Genome biology*, *10*(6), 1-18. https://doi.org/10.1186/gb-2009-10-6-r68

Zook, J. M., Hansen, N. F., Olson, N. D., Chapman, L., Mullikin, J. C., Xiao, C., ... & Salit, M. (2020). A robust benchmark for detection of germline large deletions and insertions. *Nature biotechnology*, *38*(11), 1347-1355. https://doi.org/10.1038/s41587-020-0538-8

FIGURE LEGENDS

Figure 1. Procedures for intra-alignment and inter-alignment recollection of evidence (signatures) for indels, inversions, and duplications from reads aligned to a reference genome. Duplication assignment is performed only after insertion SVs have been called from signatures (SC=Soft Clip, LSC=Longest Soft Clip, ALN=Read alignment, POS=First position in the reference).

Figure 2. DBSCAN algorithm outlined in the context of variant calling applied to an example deletion event. A distance matrix is built from signatures using the euclidean distance of three numerical values: The difference between the first and last reference position and the length of each signature. BFS is used to build clusters based on core points (points with degrees larger than a given threshold) or points reachable from these core points.

Figure 3. Likelihood estimation for each of four possible scenarios for a diploid organism. In each case, the base 10 logarithm of the obtained value is calculated. For case 1, an example of the estimation of the log-likelihood value is shown, from a situation where the SV allele with a length of 85 bp is assumed, and a read alignment contains a call supporting the SV with a

length of 90 bp. The HTS factor is a normalization constant based on the sequencing technology and the according error rate.

Figure 4. Simulation benchmarking results. The shape of points represents different depths for values of 20x,30x,45x, and 60x. A. F-score as a function of sequencing depth. B and C precision-recall curves of SV detection for alignments at different depths B. indels and C. inversions. The indel 20x F-score values are as follows: NGSEP: 99.1, Sniffles: 98.09, SVIM: 97.3, Dysgu: 98.78, CuteSV: 99.16. For SVIM, a QS filter > 10 was applied given that this provides the best results for the tool, where 0 filter provides very low precision and >20 filters provide low recall. D. Single thread execution time of all callers as a function of the depth of the input alignments.

Figure 5. Performance metrics for PacBio HiFi and ONT data of HG002, using the T1+2 SV calls of GIAB as gold standard. A, C show precision-recall curves of SV discovery over varying depths for all callers on A. HiFi data, and C. ONT data. B, D show curves comparing genotyping accuracy with recall on B. HiFi data and D. ONT data. F-score values for the 20x (HiFi, ONT) depth mappings are: NGSEP: 69.93, 68.58; Sniffles: 68.2, 67.15; SVIM: 56.58, 31.58; Dysgu: 70.4, 67.75; CuteSV: 68.28, 66.28. SVIM and CuteSV had low values in some metrics, thus, some depth points for these tools are not included, but a trajectory line is left to indicate the results trend they followed over the different datasets.

Figure 6. HGSVC2 Benchmark experiments on 20x depth HiFi mappings for each sample (A. HG00514: Han Chinese, B. HG00733: Yoruba from Nigeria, C. NA19240: Puerto Rican). All performance metrics are shown based on the results of the tested variant callers, and their exact percentage values are portrayed over each column.

SUPPLEMENTARY FILES

Supplementary file 1. Accuracy and efficiency measures for the benchmark experiments presented in this study.

Supplementary file 2. Step-by-step instructions to prepare and run the experiments with human benchmark datasets

Supplementary file 3. Supplementary tables and figures

Figure 1

Click here to access/download;Figure;Figure1_signature_recollection.pdf

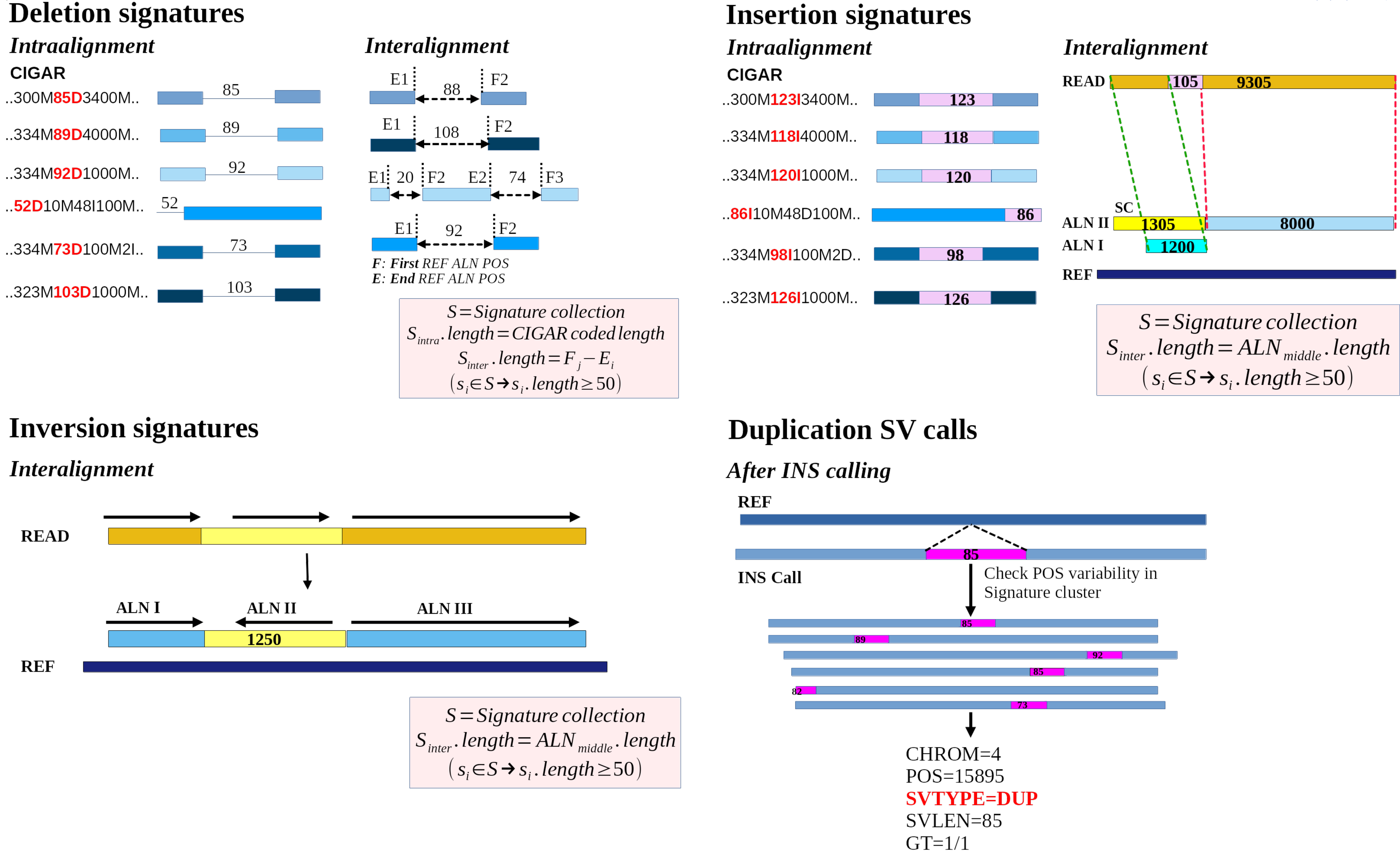

Click here to access/download;Figure;Figure2_DBSCAN_algorithm.pdf

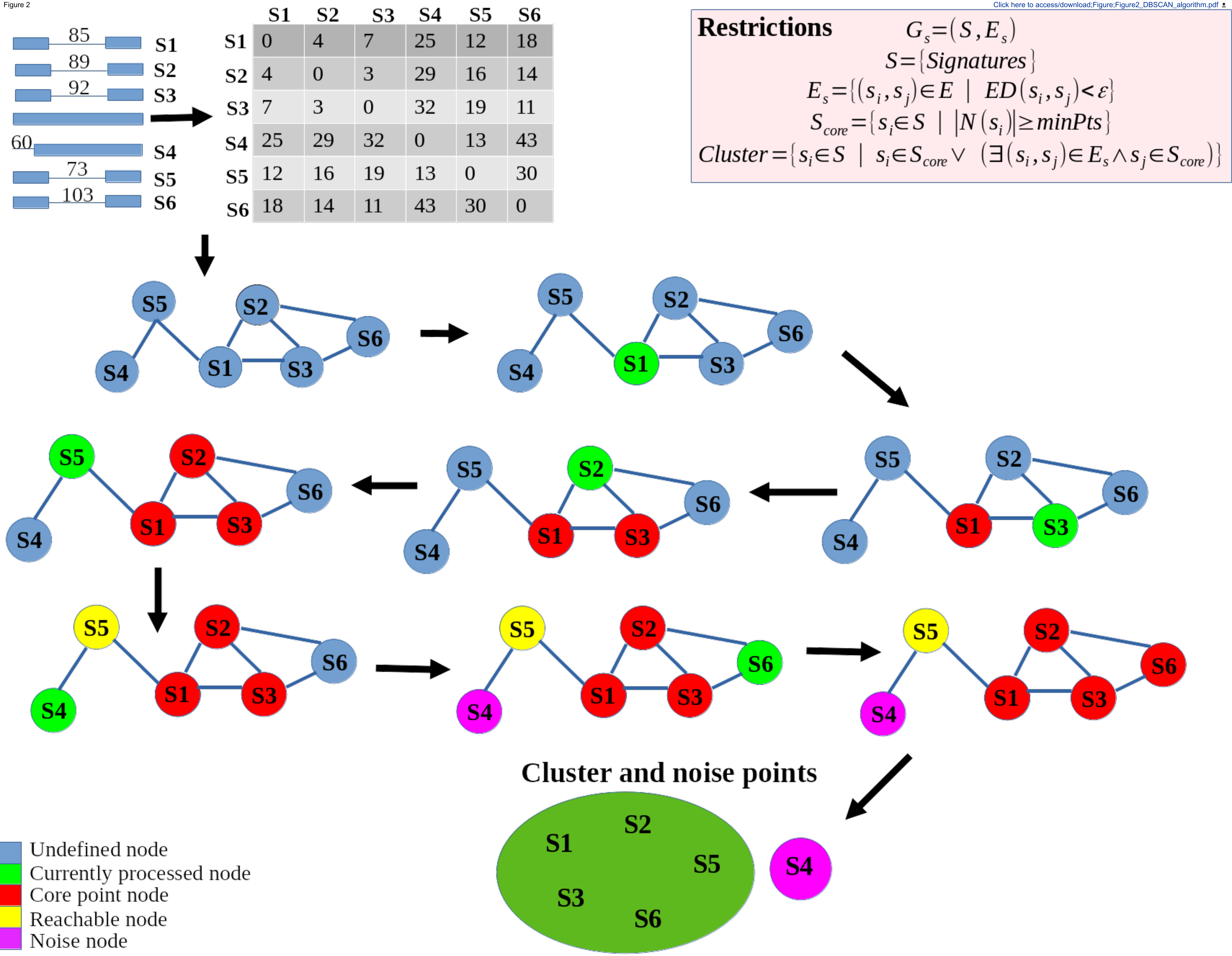

Figure 3
Click here to access/download;Figure;Figure3_likelihood_estimation.pdf

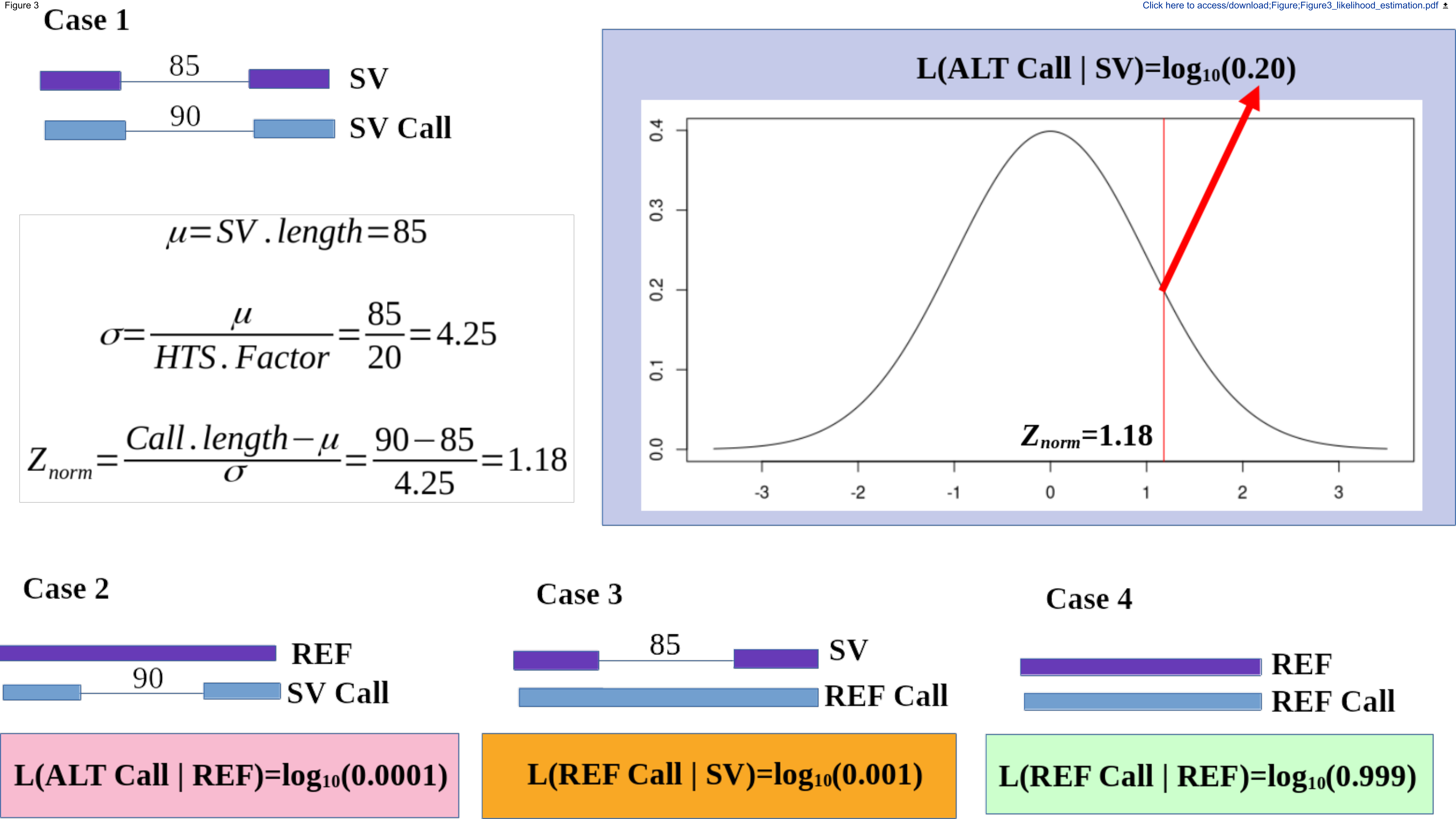

Figure 4

Click here to access/download;Figure;Figure4_SIMFigure.png

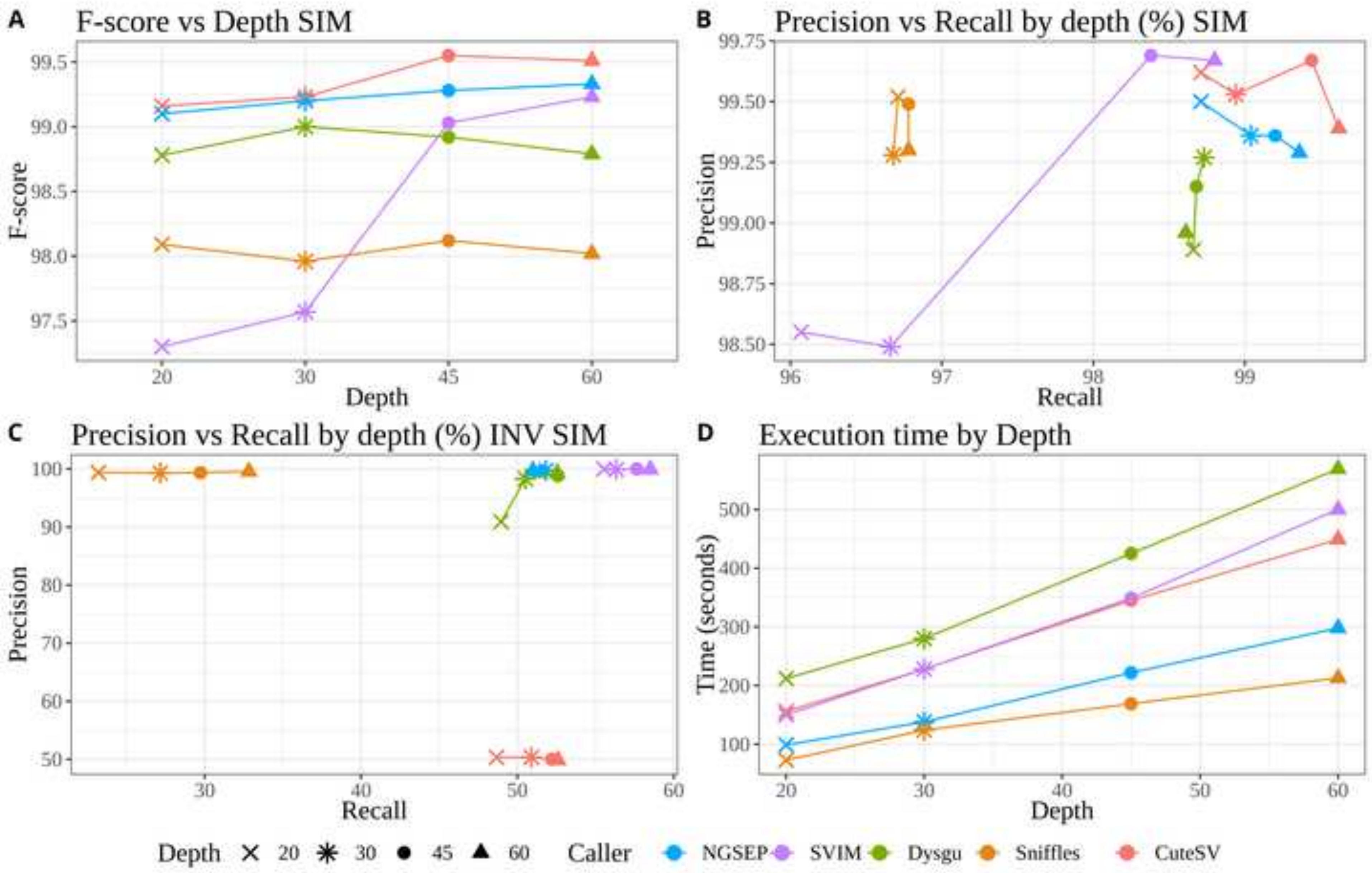

Figure 5

Click here to access/download;Figure;Figure5_HG002Tier1PlusTier2.png

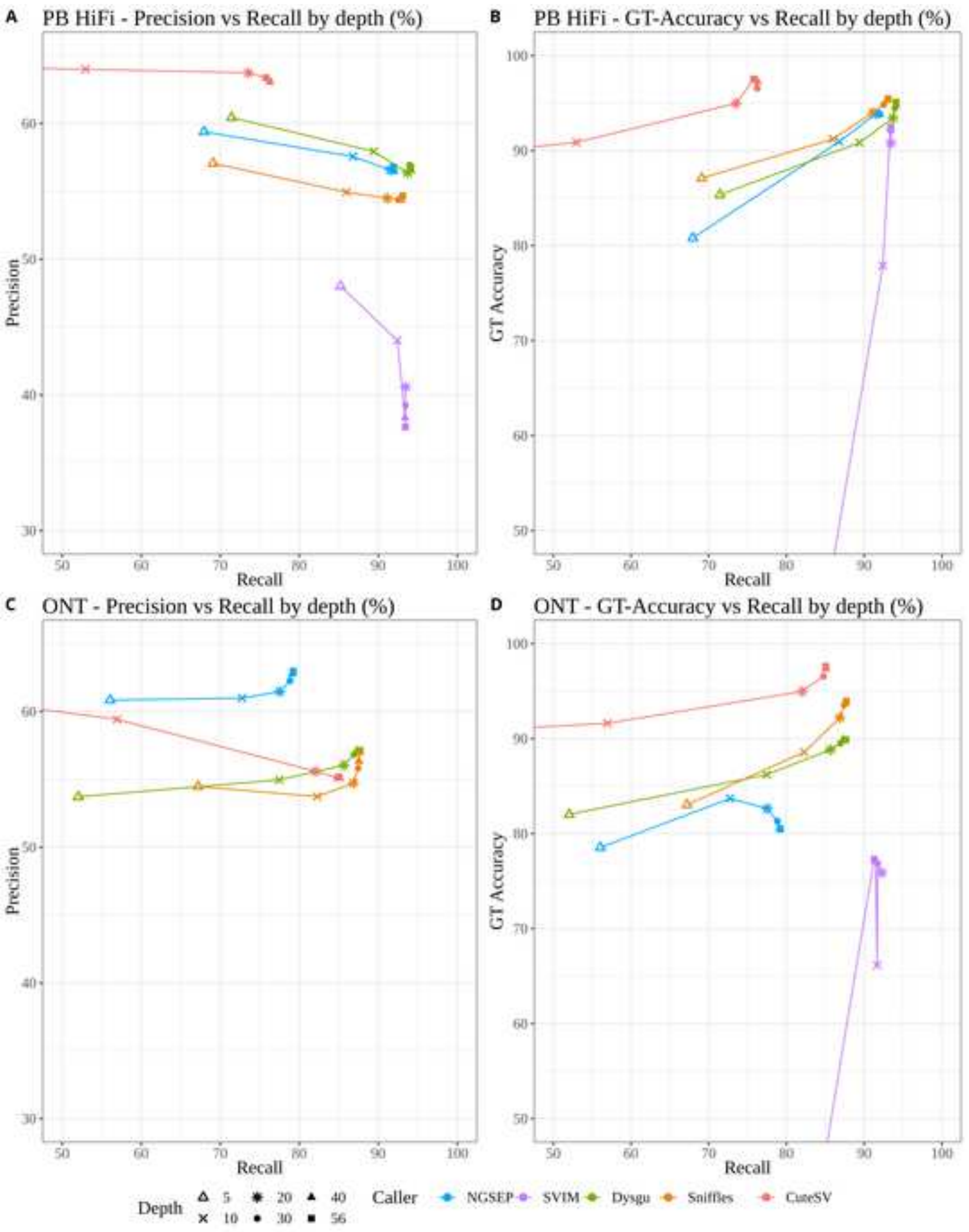

Figure 6

Click here to access/download;Figure;Figure6_HGSVC2.png

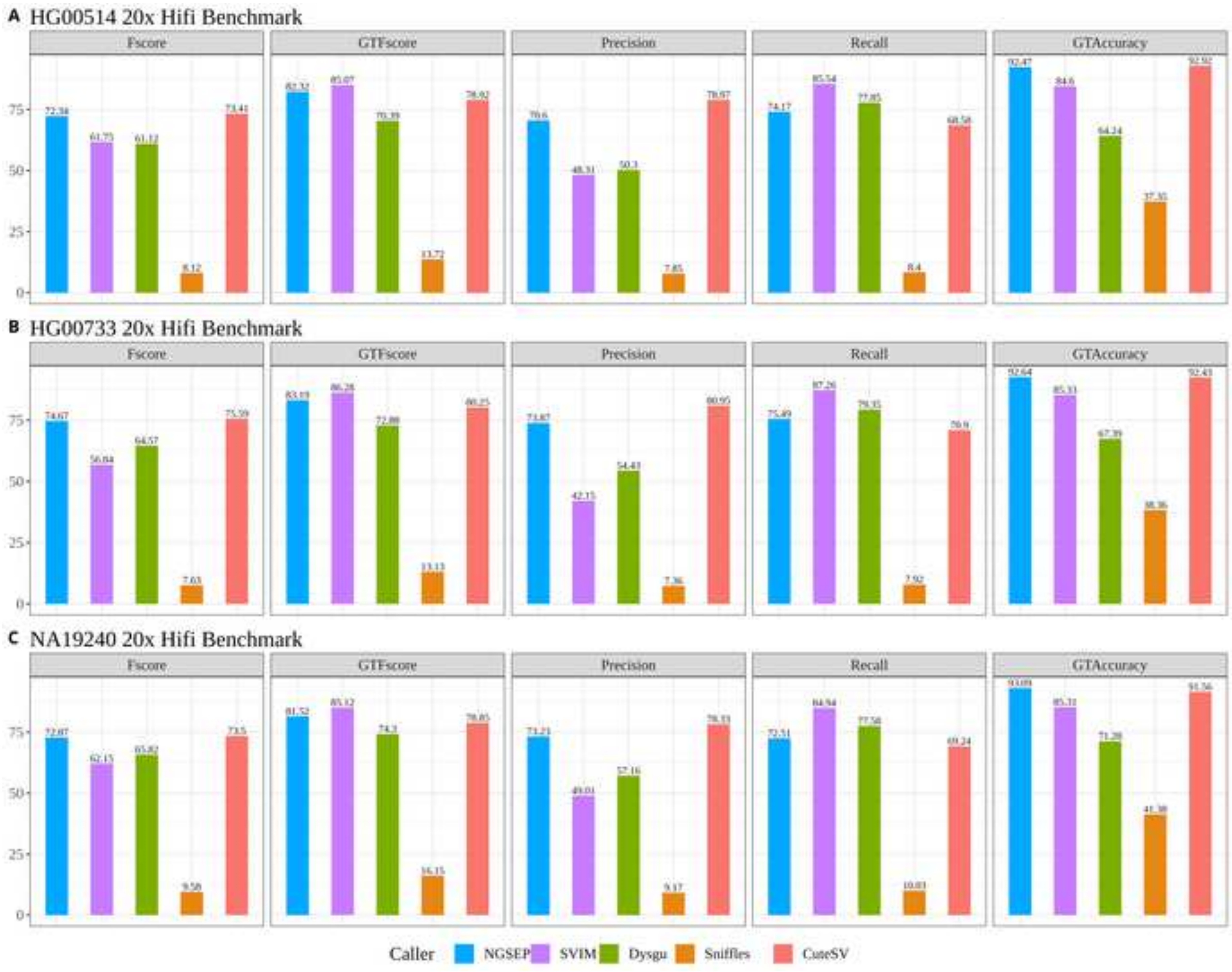

Supplementary File 1

Click here to access/download
Supplementary Material
SupplementaryFile1.xlsx

Supplementary File 2

Click here to access/download

Supplementary Material

SupplementaryFile2_benchmarkDetailedInstructions.pdf

Supplementary File 3

Click here to access/download
Supplementary Material
SupplementaryFile3_SupplementaryTablesAndFigures.pdf

Click here to access/download;Personal Cover;NGSEPSVsLongReads_GigaScience_rev2_answersRevi

6-Oct-2023

Dear editor Dr. Hans Zauner

Many thanks for your assessment of our manuscript 'A graph clustering algorithm for detection and genotyping of structural variants from long reads' and for giving us the opportunity to submit a revised version of the manuscript. We carefully read the follow up comments of the reviewer. We updated the additional benchmark experiments and made changes in the manuscript to address each comment. Please find our answers below for each specific comment. To facilitate the revision process, we marked in red the changes performed from the previous version of the manuscript.

This revised version was seen and approved by all co-authors of this manuscript. We appreciate your consideration of this version and we look forward to your assessment.

Sincerely

Jorge Duitama Ph.D
Associate professor
Systems and Computing Engineering Department
Universidad de los Andes
Bogotá, Colombia
Tel: (+57) (1) 3394949 Ext 1686.
E-mail: ja.duitama@uniandes.edu.co

GIGA-D-23-00070R1
A graph clustering algorithm for detection and genotyping of structural variants from long reads
Nicolás Gaitán; Jorge Duitama
GigaScience

Dear Dr. Duitama,

Your revised manuscript "A graph clustering algorithm for detection and genotyping of structural variants from long reads" (GIGA-D-23-00070R1) has been assessed again by one of the reviewers. Based on the latest report, I feel the manuscript can be potentially acceptable for publication in GigaScience, once you have carried out some important follow-up revisions suggested by the reviewer.

The latest report is below.

Once you have made the necessary corrections, please submit a revised manuscript online at:

If you have forgotten your username or password please use the "Send Login Details" link to get your login information. For security reasons, your password will be reset.

Please include a point-by-point within the 'Response to Reviewers' box in the submission system. Please ensure you describe additional experiments that were carried out and include a detailed rebuttal of any criticisms or requested revisions that you disagreed with. Please also ensure that your revised manuscript conforms to the journal style, which can be found in the Instructions for Authors on the journal homepage. If the data and code has been modified in the revision process please be sure to update the public versions of this too.

The due date for submitting the revised version of your article is 20 Dec 2023.

We look forward to receiving your revised manuscript soon.

Best wishes,

Hans Zauner
GigaScience

Reviewer reports:
Reviewer #1: I wish to thank the authors for the detailed response, including more data, and for addressing most of the points I raised in the initial review. I have a few outstanding issues with the manuscript, which should not be too difficult to address.

1. I apologize for not finding the version numbers in the original submission. However, the versions of most of the tools are out of date by roughly a year. CuteSV, dysgu and sniffles have received substantial updates. The manuscript would be improved if these were updated to recent versions.

R. We thank the reviewer for the follow up revision and comments. We updated the benchmark experiments using the newest versions of each tool at this moment (Dysgu v1.6.1, CuteSV v2.0.3, Sniffles v2.2, and SVIM v2.0.0) and adjusted the manuscript according to the new results. We made different observations when comparing the new versions against the older ones. The improvements of CuteSV in our updated experiments are mostly caused by the change in the value of the min_support parameter, rather than the version update. We follow this discussion in-depth in the response to the second comment. Regarding Dysgu, we found that the results on the GIAB dataset improved significantly in terms of precision for PacBio data, but this was not the case for the Tier 1+2 benchmark from the Nanopore data, even with the recommended parameters. In particular, for the GIAB pbmm2 remappings, we encountered segmentation fault errors that made it impossible to use v1.6.1, thus, we downgraded to Dysgu v1.5 for that specific dataset. Since Dysgu is in active development and this outcome is probably produced by a bug that could be easily fixed, we decided not to report in the paper this bug and the version downgrade that we had to do. Additionally, the recall of Dysgu on the HGSVC2 samples decreased significantly compared to v1.3.11, while the improvement of precision was not enough to compensate, generating a reduction in F1-score overall. The results of Sniffles did not change significantly, and the issue of reported doubled lengths for SVs discovered from the HGSVC2 samples remains with the updated version. Therefore, we applied

the same previous solution to include a benchmark with a smaller reciprocal overlap parameter for Truvari (Supplementary Figures 9,11). Nevertheless, updating software versions allowed us to improve our benchmarking experiments and provided insights into the best practices for using each caller.

2. Going back to the point that cuteSV shows low sensitivity. As now pointed out by the authors, this is due to the default parameter for minimum read support, which is normally set at 10 for the tool. This probably explains the poor performance at low coverages and the excellent performance at high coverages. However, testing cuteSV with default settings on low coverage samples (5x, 10x) is not a fair test of the tool. Can I suggest making a precision-recall curve at one or two fixed coverage values but vary the minimum-support of the tools. This would be an interesting supplementary figure and would show how optimizing the minimum support parameter could affect performance.

R. We followed this suggestion and updated the experiments using the latest version of CuteSV, and reducing the minimum read support to 5x. However, we ran into an issue running the latest version of CuteSV (v2.0.3) on the original alignments downloaded from the GIAB repository. The tool produced low recall for all depths. This issue was fixed running this version with the pbmm2 realigned datasets. This unexpected behavior did not happen with version 1.0.13 of CuteSV.

Going back to the min support parameter, we also performed experiments varying the this parameter (1,5,10,15,20) on the PacBio 20x HG002 reads, realigned with pbmm2, and with the ONT reads for HG002 (See supplementary figure 13). We observed that lowering the min_support value to 5 produced the best performance metrics. In comparison to the results with the parameter set to 5, a value of 1 creates more false positives in exchange for better sensitivity, while bigger values significantly decrease recall with no precision improvement. Hence, we applied a *min_support=5* to all runs of CuteSV in all the benchmark experiments, improving the performance of this software overall. In particular, this change produced a significant improvement in the performance of CuteSV on the HGSVC2 benchmark.

3. The performance of NGSEP seems to remain high at different coverages. Could the authors explain why this might be the case, for example are some dynamic thresholds used, or is this all down to the genotyping?

R. Your assessment is correct regarding the genotyping phase as the main cause for the consistent performance of NGSEP at different coverages, particularly regarding precision. Our Bayesian genotyping algorithm accurately predicts most false positive cases where inconsistent evidence supports an SV call according to the total amount of spanning read alignments, classifying the call as a Homozygous reference and filtering it out from the vcf output. In particular, when a low-depth sample is analyzed, each supporting read will have more weight in the probability of an alternate call, while keeping discordant or unique signals from being

reported. Hence, we avoided the need to implement any dynamic thresholds or even a minimal read support parameter.

4. I would like to try and replicate the results in the paper, but the authors appear to be merging several different samples before down-sampling to the desired coverage value. I don't have an issue with this approach, but at the same time it makes repeating the results problematic. It would be useful for others in the field if a supplementary section was included, giving a series of commands to repeat some of these results, even if only for a single sample.

R. We added the supplementary file 2 with a thorough guide, including download links to each dataset, to ensure the reproducibility of our benchmarking procedure.
