# Supplementary material for: A graph clustering algorithm for detection and genotyping of structural variants from long reads
Source: Gigascience. 2024 Jan 11;13:giad112. doi: 10.1093/gigascience/giad112 (PMC10783151; doi:10.1093/gigascience/giad112)
Supplement: giad112_Supplemental_Files [file giad112_supplemental_files.zip › SupplementaryFile2_benchmarkDetailedInstructions.pdf]

Benchmarking guide

This document contains step-by-step instructions to run the experiments with human benchmark samples presented in this study.

GIAB Benchmarking

1. **Download the data:**

PB CCS alignments:
$ wget
https://ftp.ncbi.nih.gov/giab/ftp/data/AshkenazimTrio/HG002_NA24385_son/PacBio_CCS_15kb_20kb_chemistry2/GRCh37/HG002.SequelII.merged_15kb_20kb.GRCh37.duplomap.bam

ONT alignments:
$ wget
https://ftp.ncbi.nih.gov/giab/ftp/data/AshkenazimTrio/HG002_NA24385_son/UCSC_Ultralong_OxfordNanopore_Promethion/HG002_GRCh37_ONT-UL_UCSC_20200508.phased.bam

Reference genome:
https://www.ncbi.nlm.nih.gov/assembly/GCF_000001405.13/

Find and download on the web page, rename it as **hg19.fa**

SV Gold Standard and bed files:

$ wget
https://ftp-trace.ncbi.nlm.nih.gov/ReferenceSamples/giab/release/AshkenazimTrio/HG002_NA24385_son/NIST_SV_v0.6/HG002_SVs_Tier1_v0.6.vcf.gz

$ wget
https://ftp-trace.ncbi.nlm.nih.gov/ReferenceSamples/giab/release/AshkenazimTrio/HG002_NA24385_son/NIST_SV_v0.6/HG002_SVs_Tier1_v0.6.vcf.gz.tbi

$ wget
https://ftp-trace.ncbi.nlm.nih.gov/ReferenceSamples/giab/release/AshkenazimTrio/HG002_NA24385_son/NIST_SV_v0.6/HG002_SVs_Tier1_v0.6.bed

$ wget
https://ftp-trace.ncbi.nlm.nih.gov/ReferenceSamples/giab/release/AshkenazimTrio/HG002_NA24385_son/NIST_SV_v0.6/HG002_SVs_Tier1plusTier2_v0.6.1.bed

2. **Remove alignments to alternative contigs:**

$ samtools view -h -b -o HG002.56.CCS.bam GIAB_GRCh37_Benchmark/alignments/HG002.SequelII.merged_15kb_20kb.GRCh37.duplomap.bam 1 2 3 4 5 6 7 8 9 10 11 12 13 14 15 16 17 18 19 20 21 22 X Y

$ samtools view -h -b -o HG002.47.ONT.bam https://ftp.ncbi.nih.gov/giab/ftp/data/AshkenazimTrio/HG002_NA24385_son/UCSC_Ultralong_OxfordNanopore_Promethion/HG002_GRCh37_ONT-UL_UCSC_20200508.phased.bam 1 2 3 4 5 6 7 8 9 10 11 12 13 14 15 16 17 18 19 20 21 22 X Y

3. **Downsample alignments to specific depth subsets**

Example for 20x from 56x HiFi:
$ samtools view -h -b -o HG002.20.CCS.bam -s *0.35 HG002.56.CCS.bam

*Result of 20/56=0.35. To get the proportion of reads wanted per sample, the desired depth has to be divided over the original depth of the dataset. In this case, it should be 56x for the CCS mappings and 47 for ONT_UL.

4. **Call variants with each caller for each set of mappings**

Example for 20x CCS:

NGSEP	java -Xmx16g -jar NGSEPcore_4.3.2.jar SingleSampleVariantsDetector -i HG002.20.CCS.bam -r hg19.fa -o HG002.20.CCS.NGSEP.vcf -runLongReadSVs -runOnlySVs -minSVQuality 0
Sniffles2	sniffles --input HG002.20.CCS.bam --vcf HG002.20.CCS.Sniffles.vcf --threads 1 --reference hg19.fa --minsvlen 50
CuteSV	cuteSV -s 5 --min_size 50 --threads 1 --max_cluster_bias_INS 1000 --diff_ratio_merging_INS 0.9 --max_cluster_bias_DEL 1000 --diff_ratio_merging_DEL 0.5 --genotype HG002.20.CCS.bam hg19.fa HG002.20.CCS.CuteSV.vcf /PATH/working_directory/
Dysgu	dysgu call --mode pacbio --min-size 50 -o HG002.20.CCS.Dysgu.vcf -p1 hg19.fa temp_dir HG002.20.CCS.bam
SVIM	svim alignment --min_sv_size 50 /PATH/output_directory HG002.20.CCS.bam hg19.fa<br>*Filter VCF output:*<br>$ awk '{if(substr($1,1,1)=="#" || (match($3, "DEL") || match($3, "INS") && $6>0)) print $0}' /PATH/output_directory/variants.vcf | sed 's/DUP:INT/INS/g' | sed 's/DUP:TANDEM/INS/g' > HG002.20.CCS.SVIM.vcf

pbsv	**Realignment step:*<br>$ pbmm2 align hg19.fa HG002.20.CCS.bam HG002.20.CCS.pbmm2.bam --sort --preset CCS --sample HG002 -j 16 -J 8<br>$ pbsv discover -s HG002 --ccs HG002.20.CCS.pbmm2.bam /PATH/sample.svsig.gz<br>$ pbsv call -m 50 --ccs hg19.fa /PATH/sample.svsig.gz HG002.20.CCS.pbsv.vcf

*The realignment was performed because pbsv could not be executed on the original CCS alignment file. Additionally, the latest version of CuteSV at the moment (2.0.3) shows decreased recall on the original CCS alignment compared to the pbmm2 realignment, which did not happen with the previously tested version (CuteSV v1.0.13).

5. **Filter inversions, translocations, and breakends, transform duplications into insertions**

$ awk -v FS="\t" -v OFS="\t" '!match($8, "SVTYPE=INV") && !match($8, "SVTYPE=TRA") && !match($8, "SVTYPE=BND")' HG002.20.CCS.$CALLER.vcf > HG002.20.CCS.$CALLER.filtered.vcf

$ sed -i 's/<DUP>/<INS>/g;s/SVTYPE=DUP/SVTYPE=INS/g;s/TYPE=DUP/TYPE=INS/g' HG002.20.CCS.$CALLER.filtered.vcf

6. **Produce performance metrics with Truvari**

Example for Tier1 + Tier2 regions:
$ bgzip -c HG002.20.CCS.$CALLER.filtered.vcf > HG002.20.CCS.$CALLER.filtered.vcf.gz

$ tabix -p vcf HG002.20.CCS.$CALLER.filtered.vcf.gz

$ truvari bench -b HG002_SVs_Tier1_v0.6.vcf.gz -c HG002.20.CCS.$CALLER.filtered.vcf.gz -f hg19.fa -r 1000 -p 0.00 -o benchmark_output_dir/ --passonly --includebed HG002_SVs_Tier1plusTier2_v0.6.1.bed

HGSVC2 Benchmarking

1. **Download all alignment files from the following list (with wget, for example):**

ftp://ftp.sra.ebi.ac.uk/vol1/run/ERR386/ERR3861382/HG00733-hifi-r54329U_20190607_183639-A01.bam
ftp://ftp.sra.ebi.ac.uk/vol1/run/ERR386/ERR3861383/HG00733-hifi-r54329U_20190615_010116-A01.bam
ftp://ftp.sra.ebi.ac.uk/vol1/run/ERR386/ERR3861384/HG00733-hifi-r54329U_20190617_231034-B01.bam
ftp://ftp.sra.ebi.ac.uk/vol1/run/ERR386/ERR3861385/HG00733-hifi-r54329U_20190617_231034-A01.bam
ftp://ftp.sra.ebi.ac.uk/vol1/run/ERR386/ERR3861386/HG00733-hifi-r54329U_20190701_221906-A01.bam
ftp://ftp.sra.ebi.ac.uk/vol1/run/ERR386/ERR3861387/HG00733-hifi-r54329U_20190629_175135-A01.bam
ftp://ftp.sra.ebi.ac.uk/vol1/run/ERR382/ERR3822935/HG00733-hifi-r54329U_20190827_172128-1_A01.bam
ftp://ftp.sra.ebi.ac.uk/vol1/run/ERR498/ERR4982327/HG00514-hifi-r54329U_20200715_193257-A01.bam
ftp://ftp.sra.ebi.ac.uk/vol1/run/ERR498/ERR4982328/HG00514-hifi-r54329U_20200717_234302-A01.bam
ftp://ftp.sra.ebi.ac.uk/vol1/run/ERR498/ERR4982329/HG00514-hifi-r54329U_20200717_234302-B01.bam
ftp://ftp.sra.ebi.ac.uk/vol1/run/ERR496/ERR4968413/NA19240_20191002_CLEE_m54336U_190827_013439.ccs.bam
ftp://ftp.sra.ebi.ac.uk/vol1/run/ERR496/ERR4968409/NA19240_20191002_CLEE_m64039_190921_152439.ccs.bam
ftp://ftp.sra.ebi.ac.uk/vol1/run/ERR496/ERR4968410/NA19240_20191002_CLEE_m54336U_190829_230546.ccs.bam
ftp://ftp.sra.ebi.ac.uk/vol1/run/ERR496/ERR4968412/NA19240_20191002_CLEE_m54336U_190828_075428.ccs.bam

2. **Download and prepare reference files**

Reference genome:
$ wget
http://ftp.1000genomes.ebi.ac.uk/vol1/ftp/data_collections/HGSVC2/technical/reference/20200513_hg38_NoALT/hg38.no_alt.fa.gz

High confidence multi-sample merged vcf file from HGSVC2:
$ wget
http://ftp.1000genomes.ebi.ac.uk/vol1/ftp/data_collections/HGSVC2/release/v2.0/integrated_callset/variants_freeze4_sv_insdel.tsv.gz

Extract calls as individual vcf files to obtain the reference calls of the three samples in this benchmark (HG00514, HG00733, NA19240)

```
$ bcftools query -l variants_freeze4_sv_insdel.tsv.gz > query.txt

$ for sample in `cat query.txt`;do bcftools view -c1 -Oz -s $sample -o $sample.vcf
variants_freeze4_sv_insdel.tsv.gz;done
```

*IMPORTANT: The resulting vcf sample must be corrected. A properly formatted header must be added, as well as some INFO fields. <u>In particular:</u>

- The END tag is not present in the original samples, so it must be added depending on the SV type.
- The GT field format may not work with truvari, so it has to be changed (e.g 0|1 -> 0/1).

We created a custom script to fix these issues using some of NGSEP API classes, but other solutions may be successful in producing the gold standard vcf files for benchmarking with truvari.

Bgzip compression and tabix indexing for benchmarking with Truvari (HG00514 example):

```
$ bgzip -c HG00514.reference_fixed.vcf > HG00514.reference_fixed.vcf.gz

$ tabix -p vcf HG00514.reference_fixed.vcf.gz
```

3. **Extract the reads from the mappings**

```
$ for $file in /PATH_to_mappings/*.bam;
do
	bedtools bamtofastq -i $file -fq ${file%.bam}.fastq
done
```

4. **Append the reads into a merged fastq file for each one of the three samples**

```
$ cat HG00514*.fastq > HG00514.hifi.fastq
$ cat HG00733*.fastq > HG00733.hifi.fastq
$ cat NA19240*.fastq > NA19240.hifi.fastq
```

5. **Map and sort each of the three merged read samples with minimap2 to the GRCh38 reference genome**

```
$ minimap2 -ax asm20 -R ‘$RG’ hg38.no_alt.fa.gz HG00514.hifi.fastq | samtools sort -o
HG00514.hifi.bam
$ minimap2 -ax asm20 -R ‘$RG’ hg38.no_alt.fa.gz HG00733.hifi.fastq | samtools sort -o
HG00733.hifi.bam
$ minimap2 -ax asm20 -R ‘$RG’ hg38.no_alt.fa.gz NA19240.hifi.fastq  | samtools sort -o
NA19240.hifi.bam
```

6. **Downsample each of the three bam files to 20x:**

Example for HG00514:
$ samtools view -h -b -o HG00514.20.hifi.bam -s $percentage HG002.hifi.bam

7. **Run all callers on this sample, same as step 4 of the GIAB procedure, but changing the reference to the GRCh38 genome.**
8. **Filtering VCF step, same as step 5 of the GIAB benchmark**
9. **Produce performance metrics with Truvari**

Example for HG00514 with half pct:

$ bgzip -c HG00514.20.$CALLER.filtered.vcf > HG00514.20.$CALLER.filtered.vcf.gz

$ tabix -p vcf HG00514.20.$CALLER.filtered.vcf.gz

$ truvari bench -b HG00514.reference_fixed.vcf.gz -c HG00514.20.$CALLER.filtered.vcf.gz
-f hg38.no_alt.fa.gz -r 1000 -p 0.00 --pctsize 0.35 -o benchmark_output_dir/
