# Supplementary material for: A graph clustering algorithm for detection and genotyping of structural variants from long reads
Source: Gigascience. 2024 Jan 11;13:giad112. doi: 10.1093/gigascience/giad112 (PMC10783151; doi:10.1093/gigascience/giad112)
Supplement: giad112_Supplemental_Files [file giad112_supplemental_files.zip › SupplementaryFile3_SupplementaryTablesAndFigures.pdf]

Supplementary tables and figures for:

A graph clustering algorithm for detection and genotyping of structural variants from long reads

Nicolás Gaitán[1], Jorge Duitama[1,*].

[1]Systems and Computing Engineering Department, Universidad de Los Andes, Bogotá, Colombia.

* Corresponding author. E-mail: ja.duitama@uniandes.edu.co

Supplementary Tables

Supplementary Table 1. Number of SVs included in each gold-standard dataset.

Sample / SV Type	*Deletion*	*Insertion*	*Inversion*	*Total*
Arabidopsis Simulation	2,532	1,718	2,065	6,315
T2T Human Simulation	5,000	5,000	0	10,000
GIAB Tier1+2	5,457	7,266	0	12,723
GIAB Tier1	4,199	5,442	0	9,641
HG00514 all-regions	9,109	14,279	0	23,388
HG00733 all-regions	9,308	14,469	0	23,777
NA19240 all-regions	10,926	16,376	0	27,302
HG00514 non-repeat-regions	1,529	3,883	0	5,412
HG00733 non-repeat-regions	1,543	3,990	0	5,533
NA19240 non-repeat-regions	1,846	4,783	0	6,629

Supplementary Table 2. Commands executed to test each tool evaluated in this study. For all callers, we filtered the output vcf files with the commands specified in the second to last row. This prevented Truvari from counting duplications as false positives instead of true positive insertions. We also filtered out inversions in the benchmarks with real data because the gold standard sets do not include inversions.

<table>
<tr><th>Tool</th><th>Command</th></tr>
<tr><td>NGSEP</td><td>$ java -Xmx16g -jar NGSEPcore_4.3.2.jar SingleSampleVariantsDetector -i /PATH/input_alignments.bam -r /PATH/ReferenceGenome -o /PATH/output.vcf -runLongReadSVs -runOnlySVs -minSVQuality 0</td></tr>
<tr><td>Sniffles2</td><td>$ sniffles --input /PATH/input_alignments.bam --vcf /PATH/output.vcf --threads 1 --reference /PATH/ReferenceGenome/ --minsvlen 50</td></tr>
<tr><td>CuteSV</td><td>**PB: $** cuteSV -s 5 --min_size 50 --threads 1 --max_cluster_bias_INS 1000 --diff_ratio_merging_INS 0.9 --max_cluster_bias_DEL 1000 --diff_ratio_merging_DEL 0.5 --genotype /PATH/input_alignments.bam /PATH/ReferenceGenome /PATH/output.vcf /PATH/working_directory/
ONT: $ cuteSV -s 5 --min_size 50 --threads 1 --max_cluster_bias_INS 100 --diff_ratio_merging_INS 0.3 --max_cluster_bias_DEL 100 --diff_ratio_merging_DEL 0.3 --genotype /PATH/input_alignments.bam /PATH/ReferenceGenome /PATH/output.vcf /PATH/working_directory/</td></tr>
<tr><td>Dysgu</td><td>**PB:** $ dysgu call --mode pacbio --min-size 50 -o /PATH/output.vcf -p1 /PATH/ReferenceGenome temp_dir /PATH/input_alignments.bam
ONT: $ dysgu call --divergence auto --mode nanopore --min-size 50 -o /PATH/output.vcf -p1 /PATH/ReferenceGenome temp_dir /PATH/input_alignments.bam</td></tr>
<tr><td>SVIM</td><td>$ svim alignment --min_sv_size 50 /PATH/output_directory /PATH/input_alignments.bam /PATH/ReferenceGenome/
Filter VCF output:
$ awk '{if(substr($1,1,1)=="#" || (match($3, "DEL") || match($3, "INS") && $6>0)) print $0}' /PATH/output_directory/variants.vcf | sed 's/DUP:INT/INS/g' | sed 's/DUP:TANDEM/INS/g' > output.vcf</td></tr>
<tr><td>pbsv</td><td>*Realignment step:*
$ pbmm2 align /PATH/ReferenceGenome /PATH/original_alignments.bam /PATH/input_alignments.bam --sort --preset CCS --sample “sample” -j 16 -J 8
$ pbsv discover -s “sample” --ccs /PATH/input_alignments.bam /PATH/sample.svsig.gz
$ pbsv call -m 50 --ccs /PATH/ReferenceGenome /PATH/sample.svsig.gz output.vcf</td></tr>
<tr><td>Filtering all callers for benchmark</td><td>$ awk -v FS="\t" -v OFS="\t" '!match($8, "SVTYPE=INV") && !match($8, "SVTYPE=TRA") && !match($8, "SVTYPE=BND")' output.vcf > output.filtered.vcf
$ sed -i 's/<DUP>/<INS>/g;s/SVTYPE=DUP/SVTYPE=INS/g;s/TYPE=DUP/TYPE=INS/g' output.filtered.vcf</td></tr>
<tr><td>Truvari</td><td>$ truvari bench -b /PATH/gold_standard_sv_calls.vcf -c /PATH/output.vcf.gz -f /PATH/ReferenceGenome -r 1000 -p 0.00 -o /PATH/benchmark_output_dir/ --passonly --includebed /PATH/included_regions.bed</td></tr>
</table>

Supplementary Figures

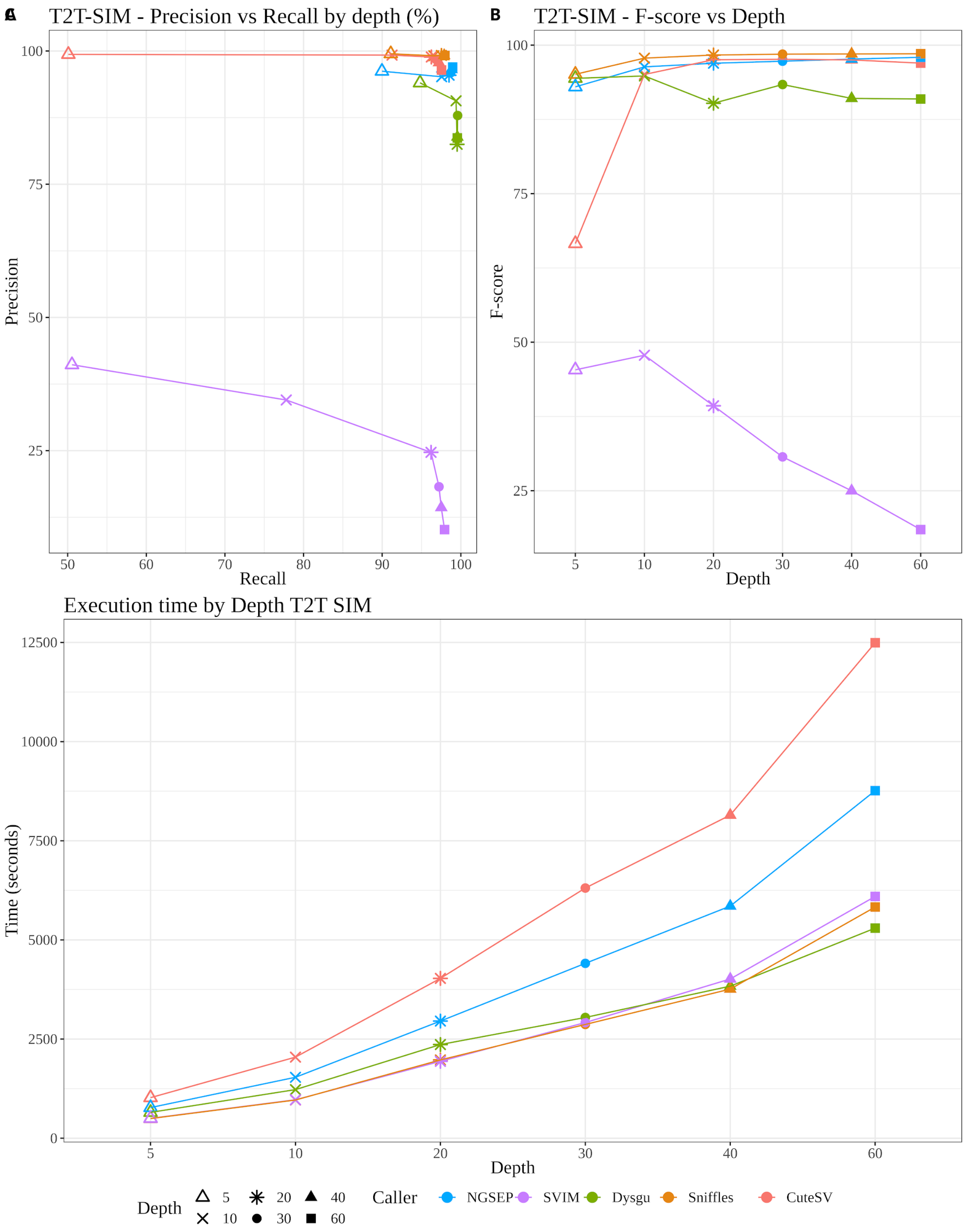

Supplementary figure 1. Human T2T Indel Simulation The shape of points represents depths for fixed values of 5x, 10x, 20x, 30x, 40x, and 60x. A. Precision-recall curves of SV detection for alignments at different depths. B. F-score as a function of sequencing depth. The 20x F-score values are as follows: NGSEP: 96.96, Sniffles: 98.34, SVIM: 39.3, Dysgu: 90.22, CuteSV: 97.55. For SVIM, a QS filter > 0 was applied. C. Single thread execution time of all callers as a function of the depth of the input alignments. An apparent linear time complexity is observed for each dataset of increasing sizes (5x: 21Gb, 10x: 42Gb, 20x: 84Gb, 30x: 126Gb, 40x: 167G, 60x: 250Gb), except for Dysgu from 40x to 60x.

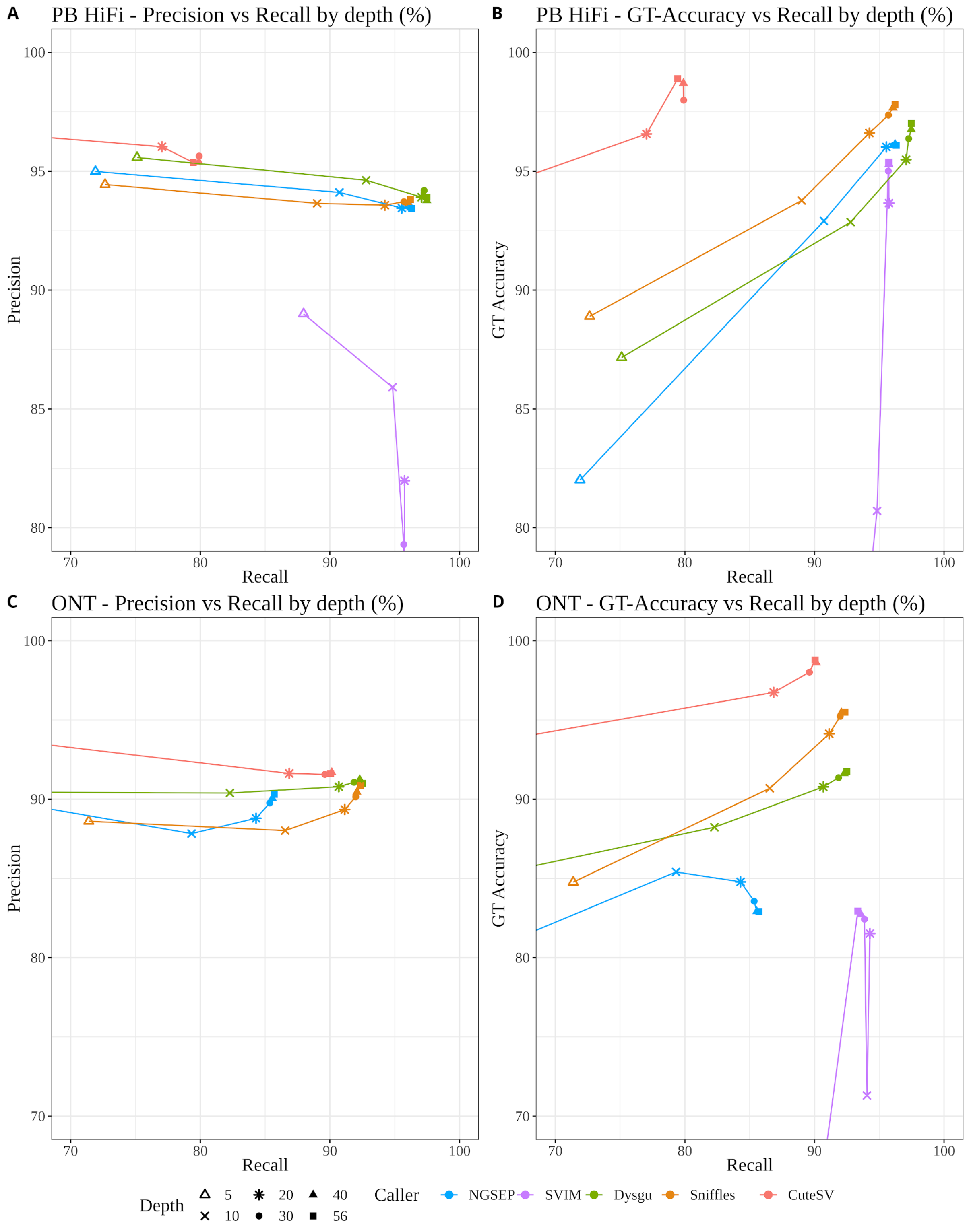

Supplementary figure 2. Benchmark HG002. Only tier 1. A, C Precision-recall curves of SV discovery over varying depths for all callers restricted to T1 regions on A. HiFi data, and C. ONT data. B, D show curves comparing genotyping accuracy with recall on B. HiFi data and D. ONT data. F-score values for the 20x (HiFi, ONT) depth mappings are: NGSEP: 94.49, 86.49; Sniffles: 93.91, 90.24; SVIM: 88.33, 11.92; Dysgu: 95.47, 90.73; CuteSV: 85.49, 89.18

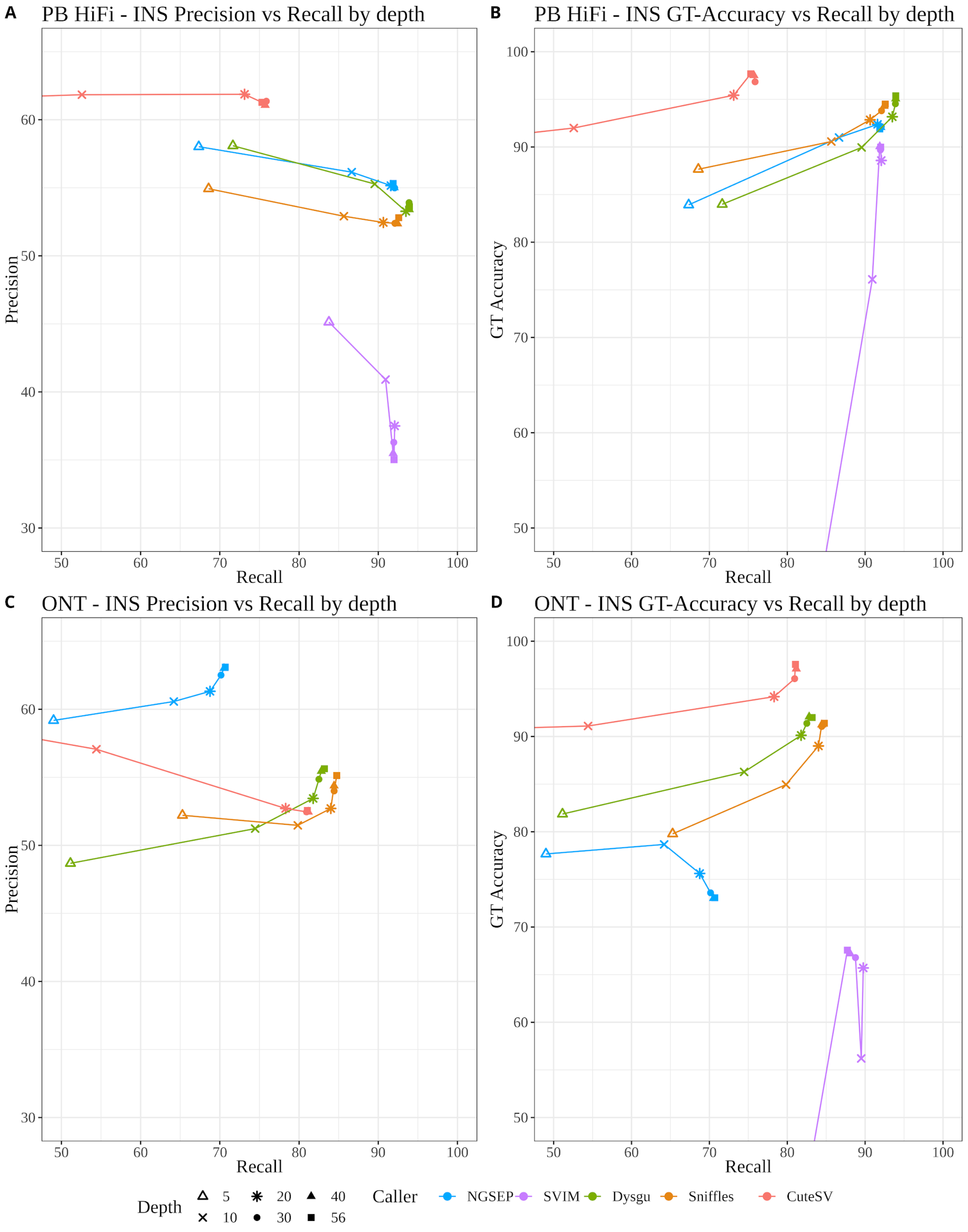

Supplementary figure 3. Benchmark HG002. Only Insertions Tier 1 + 2. A, C Insertion only precision-recall curves of SV discovery over varying depths for all callers restricted to T1+2 regions on A. HiFi data, and C. ONT data. B, D show curves comparing genotyping accuracy with recall on B. HiFi data and D. ONT data.

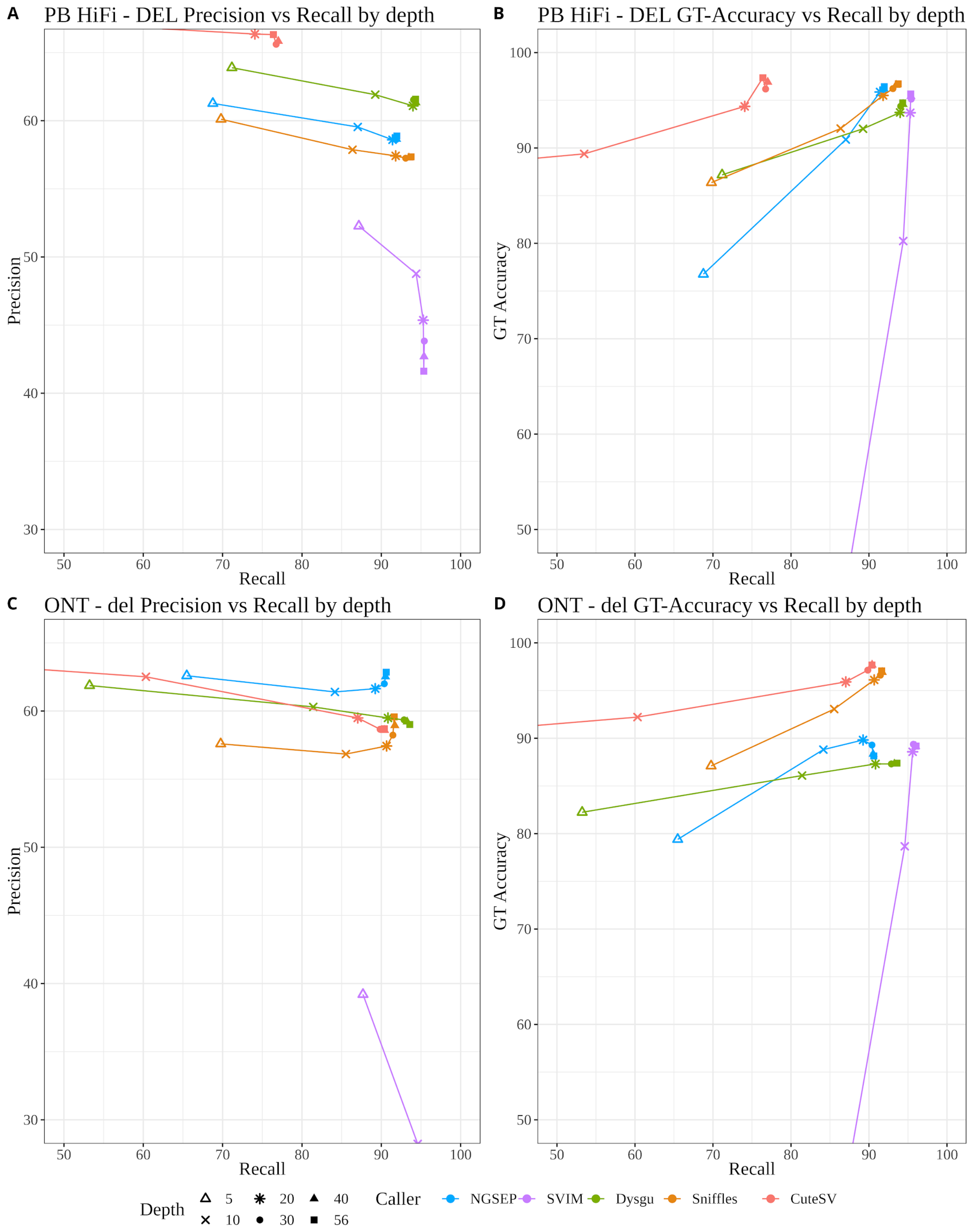

Supplementary figure 4. Benchmark HG002. Only deletions Tier 1 + 2. A, C Deletion only precision-recall curves of SV discovery over varying depths for all callers restricted to T1+2 regions on A. HiFi data, and C. ONT data. B, D show curves comparing genotyping accuracy with recall on B. HiFi data and D. ONT data.

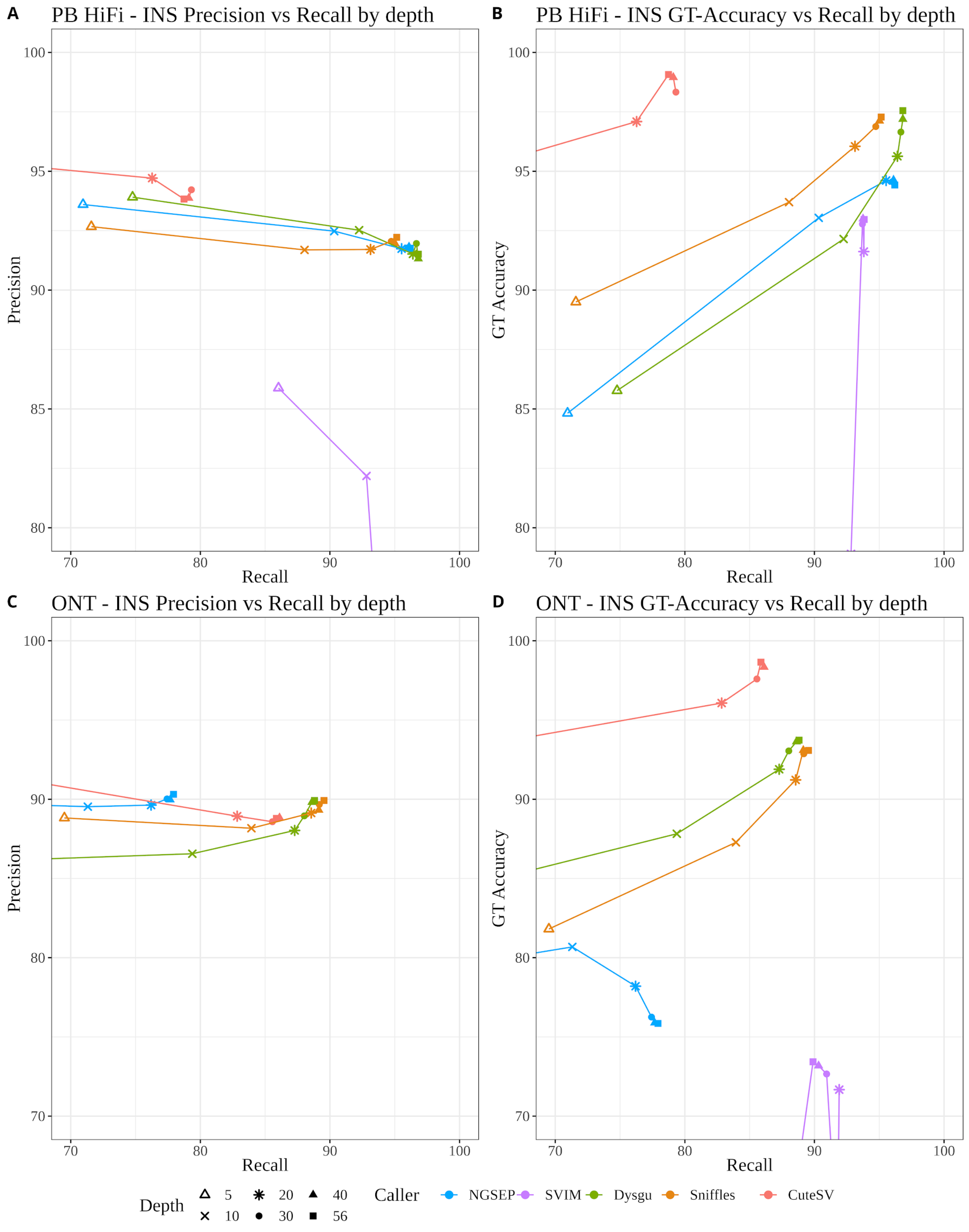

Supplementary figure 5. Benchmark HG002. Only Insertions Tier 1
A, C Insertion only precision-recall curves of SV discovery over varying depths for all callers restricted to T1 regions on A. HiFi data, and C. ONT data. B, D show curves comparing genotyping accuracy with recall on B. HiFi data and D. ONT data.

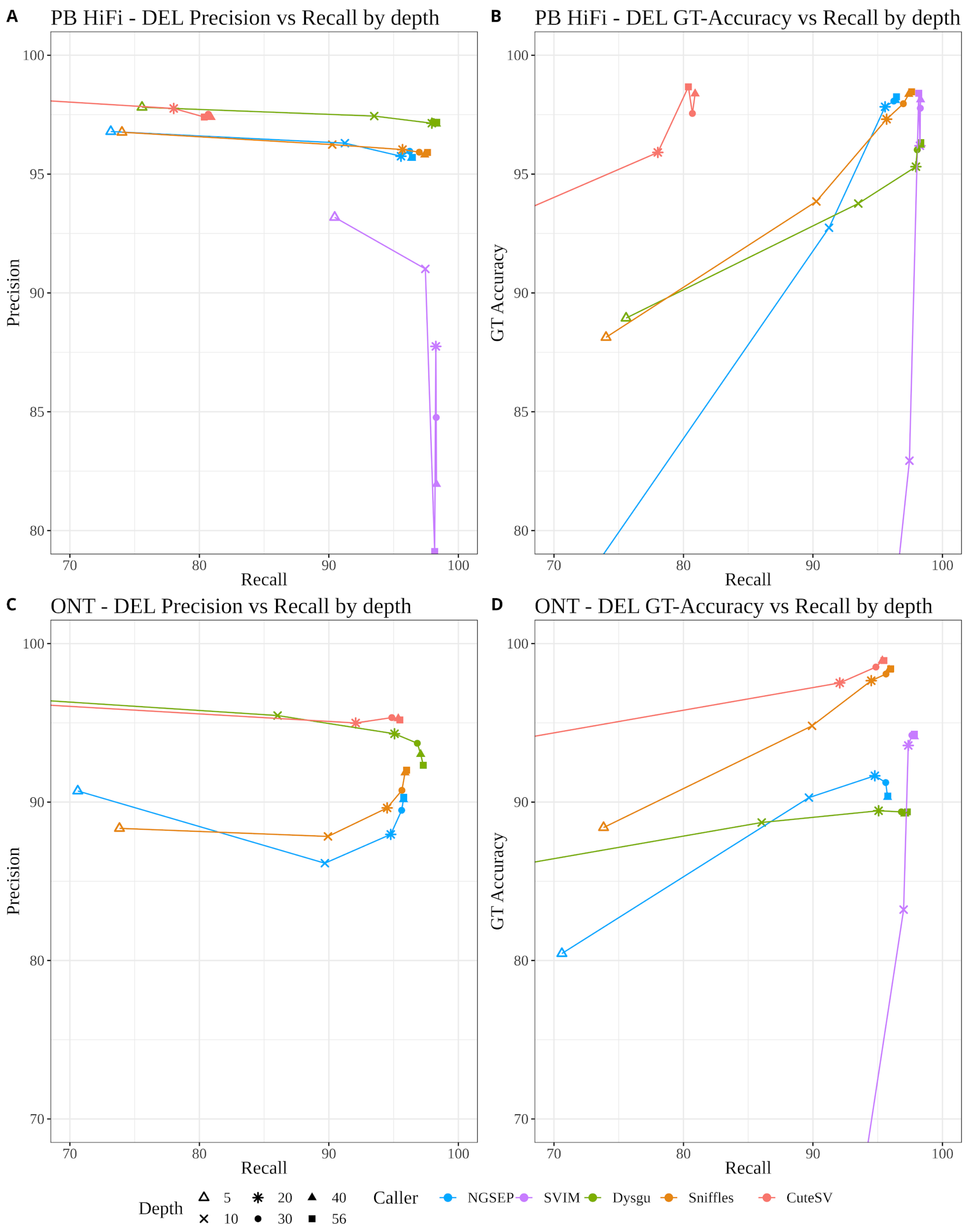

Supplementary figure 6. Benchmark HG002. Only deletions Tier 1. A, C Deletion only precision-recall curves of SV discovery over varying depths for all callers restricted to T1 regions on A. HiFi data, and C. ONT data. B, D show curves comparing genotyping accuracy with recall on B. HiFi data and D. ONT data.

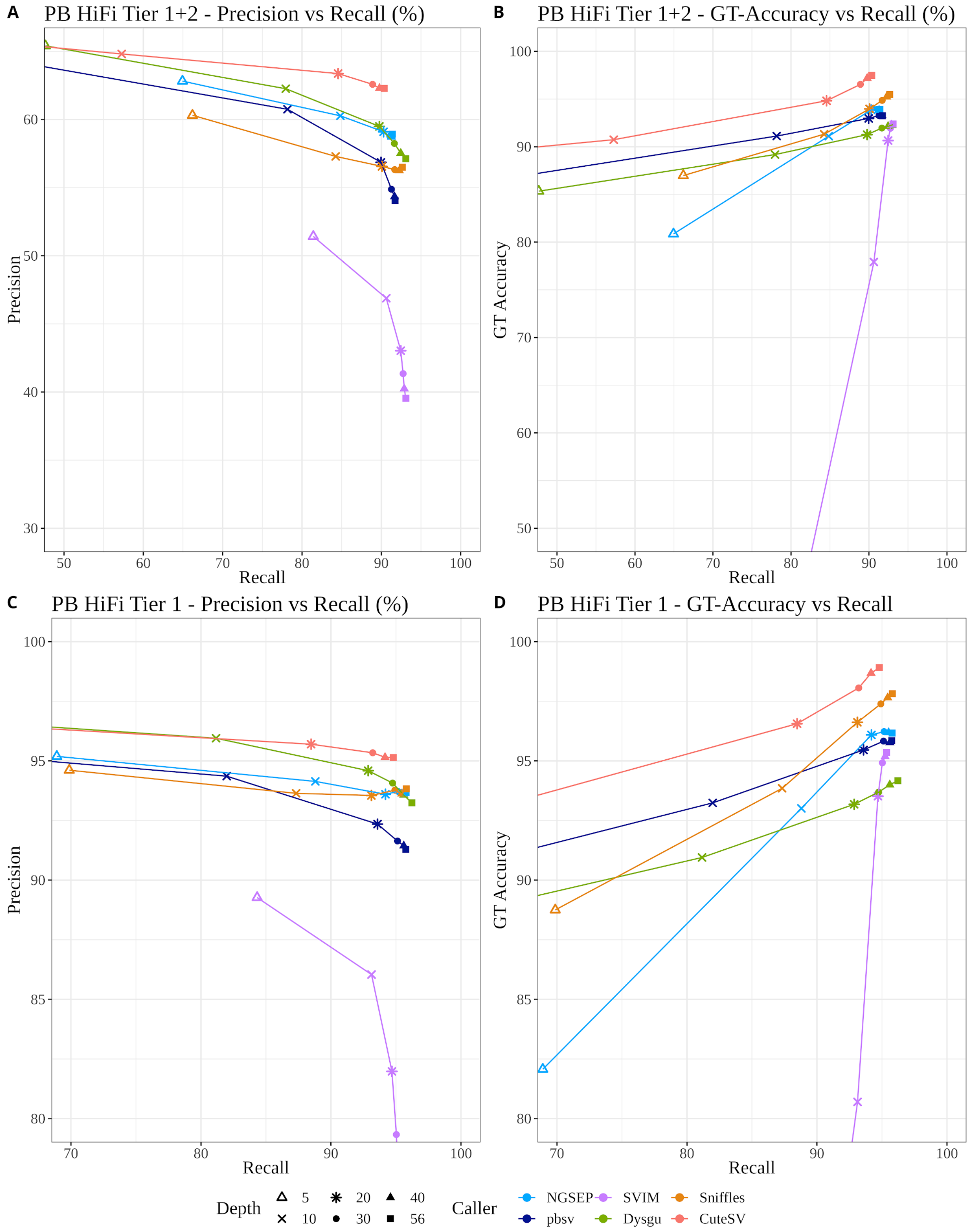

Supplementary figure 7. Benchmark HiFi HG002. Including pbsv. A, C Precision-recall curves of SV discovery over varying depths of HiFi mappings, for all callers including pbsv restricted to A. T1+2, and C. T1 regions. B, D show curves comparing genotyping accuracy with recall on B. T1+2, and D. T1. F-score values for the 20x (T1+2, T1) depth mappings are: NGSEP: 71.44, 93.89; Sniffles: 69.49, 93.33; SVIM: 58.72, 87.88; Dysgu: 71.55, 93.72; CuteSV: 72.45, 91.94; pbsv: 69.68, 92.96.

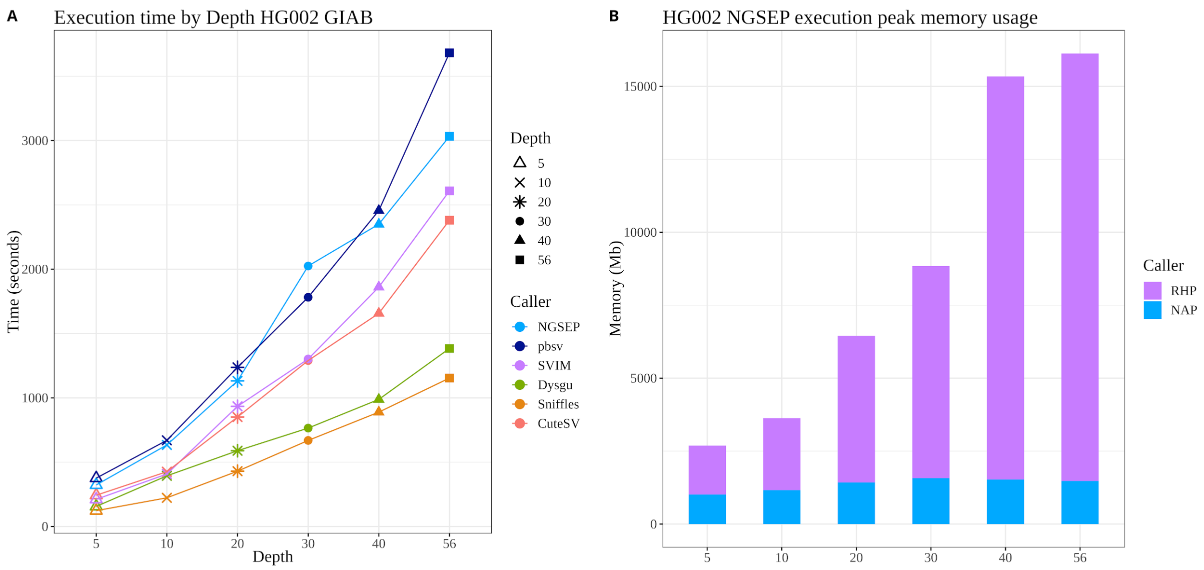

Supplementary figure 8. Time and peak memory usage from HG002 benchmarking A. Execution time of all callers under the pbmm2 aligned reads at varying input depths. Different from the recorded times of the simulations, all tools perform similarly, increasing at a linear rate. Only pbsv shows a sudden spike from 40 to 56x. B. Java Virtual Machine assessment of peak memory consumption for NGSEP caller executions at different depths of input. Peak memory usage was recorded through executions of the software, and dissected into two types. NAP stands for New allocations peak, which showcases the heap memory assigned to recently created objects. RHP for Remaining heap peak, representing objects that remain in memory, and have not been removed by the garbage collector. As can be seen, for low-depth inputs (5, 10, 20x) total memory consumption is below 8 Gb. From 30x onwards, old objects occupy most of the memory and even reach total memory consumption for the complete mappings. Nonetheless, new objects still consume minimal memory, showcasing that the algorithm is able to handle inputs close to 60x depth for human genomes with a 16Gb memory limit, which is consistent with the executions on simulated human reads.

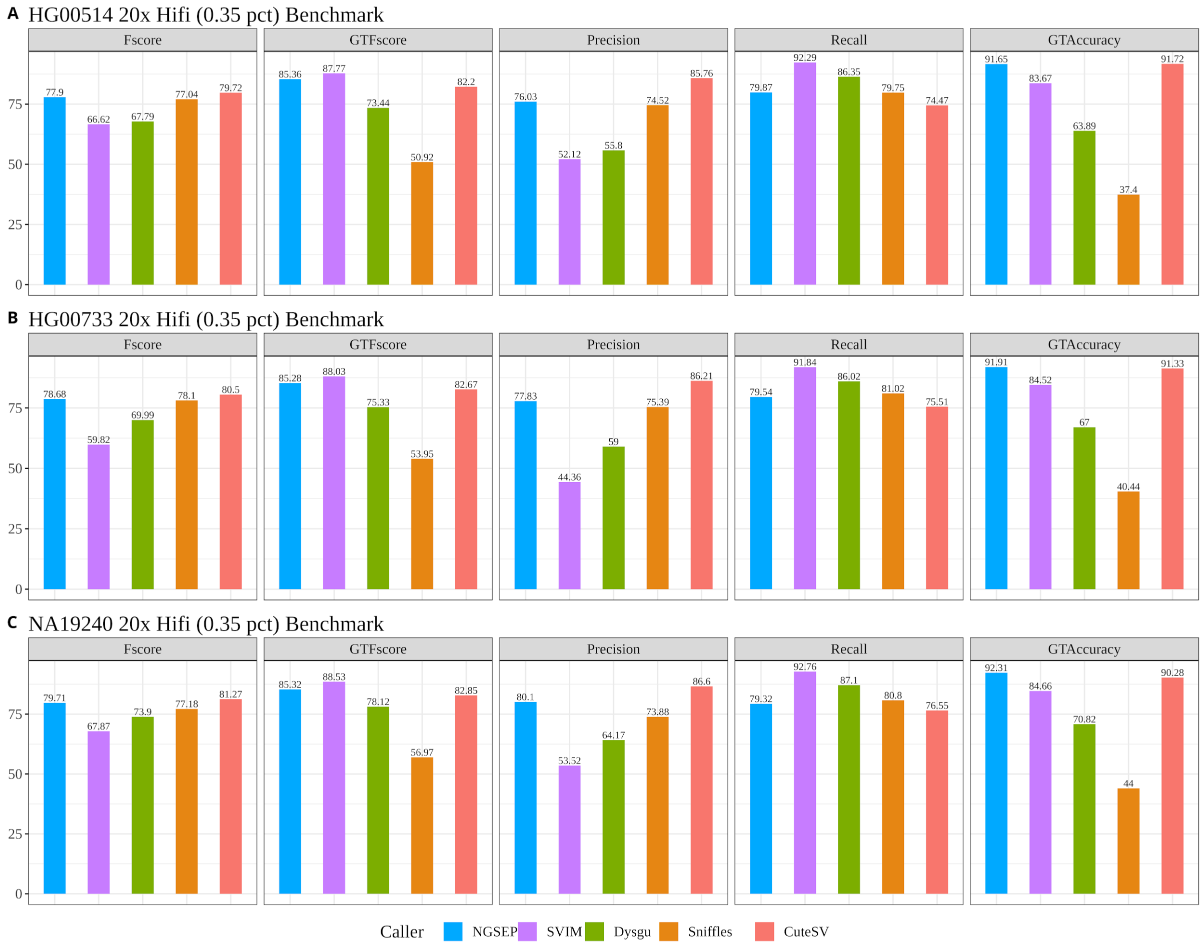

Supplementary figure 9. Benchmark HGSVC2 half pct (0.35) parameter. HGSVC2 Benchmark experiments on 20x depth HiFi mappings for each sample (A. HG00514: Han Chinese, B. HG00733: Yoruban Nigerian, C. NA19240: Puerto Rican). These results come from altering the reciprocal overlap parameter (-pct 0.35) in Truvari to favor Sniffles2, due to an error in their output. All performance metrics are shown based on the results of the tested variant callers, and their exact percentage values are portrayed over each column.

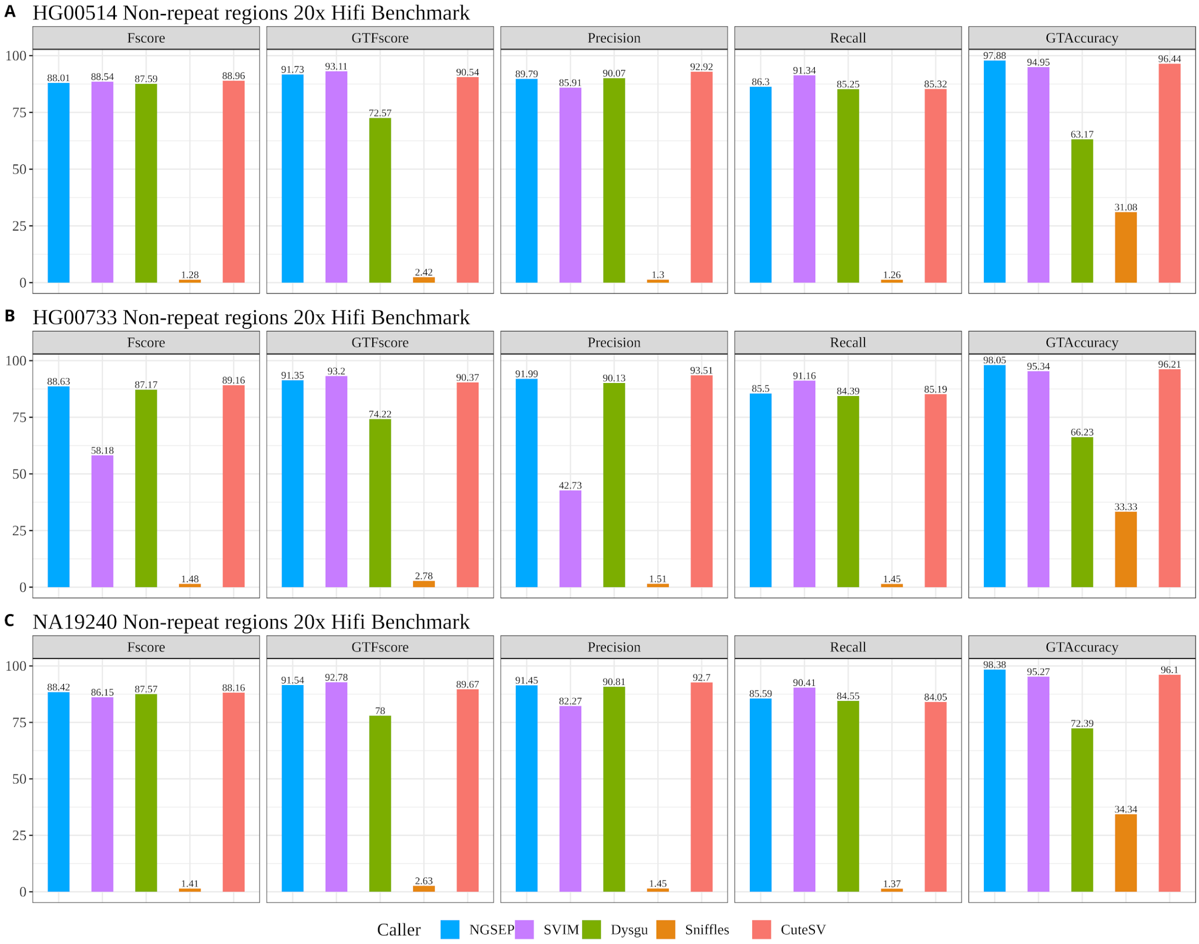

Supplementary figure 10. Benchmark HGSVC2 default parameter non-repetitive regions
HGSVC2 Benchmark experiments on 20x depth HiFi mappings for each sample (A. HG00514: Han Chinese, B. HG00733: Yoruban Nigerian, C. NA19240: Puerto Rican). Tested SVs are restricted to non-repetitive regions. All performance metrics are shown based on the results of the tested variant callers, and their exact percentage values are portrayed over each column.

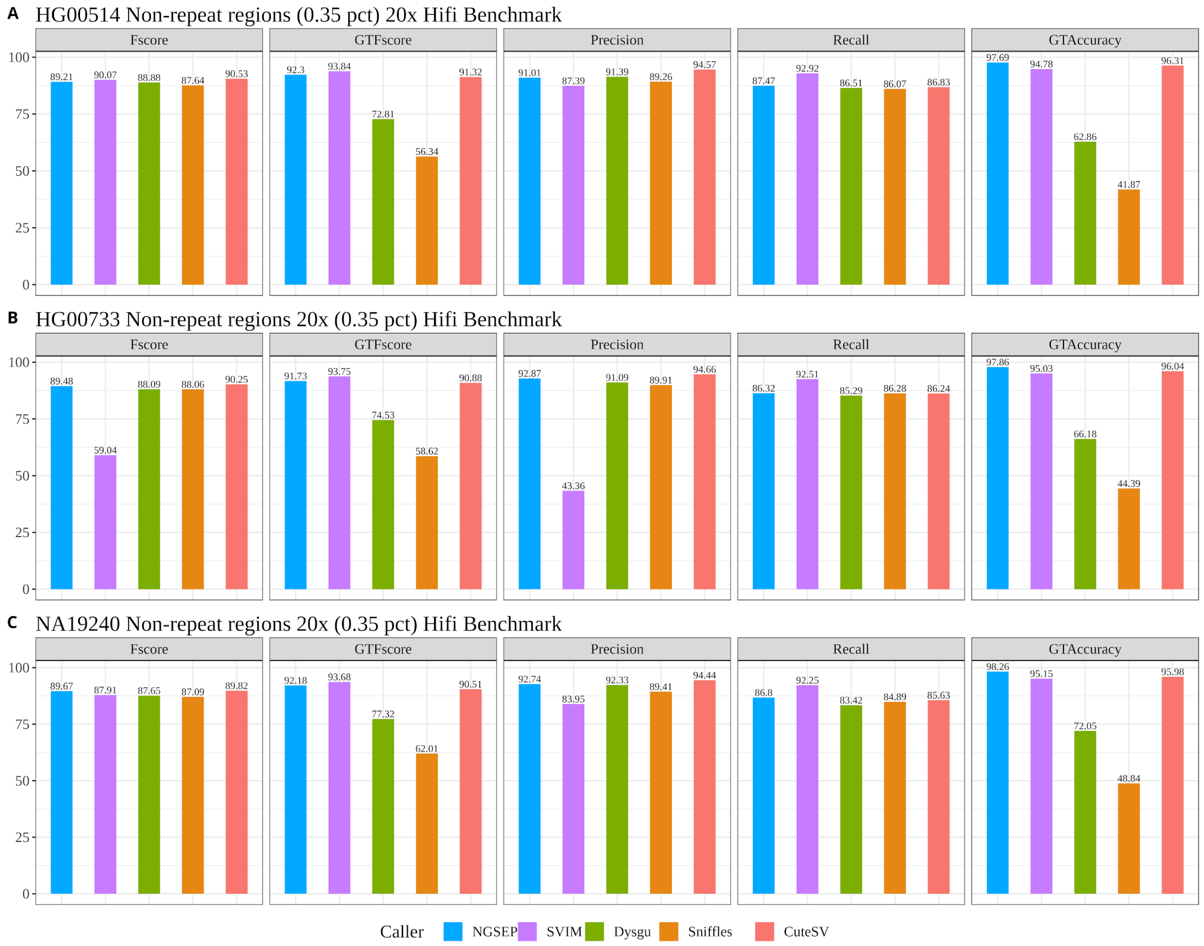

Supplementary figure 11. Benchmark HGSVC2 hal pct (0.35) parameter non-repetitive regions HGSVC2 Benchmark experiments on 20x depth HiFi mappings for each sample (A. HG00514: Han Chinese, B. HG00733: Yoruban Nigerian, C. NA19240: Puerto Rican). These results come from altering the reciprocal overlap parameter (-pct 0.35) in Truvari to favor Sniffles2, due to an error in their output. Additionally, SVs are restricted to non-repetitive genome regions.

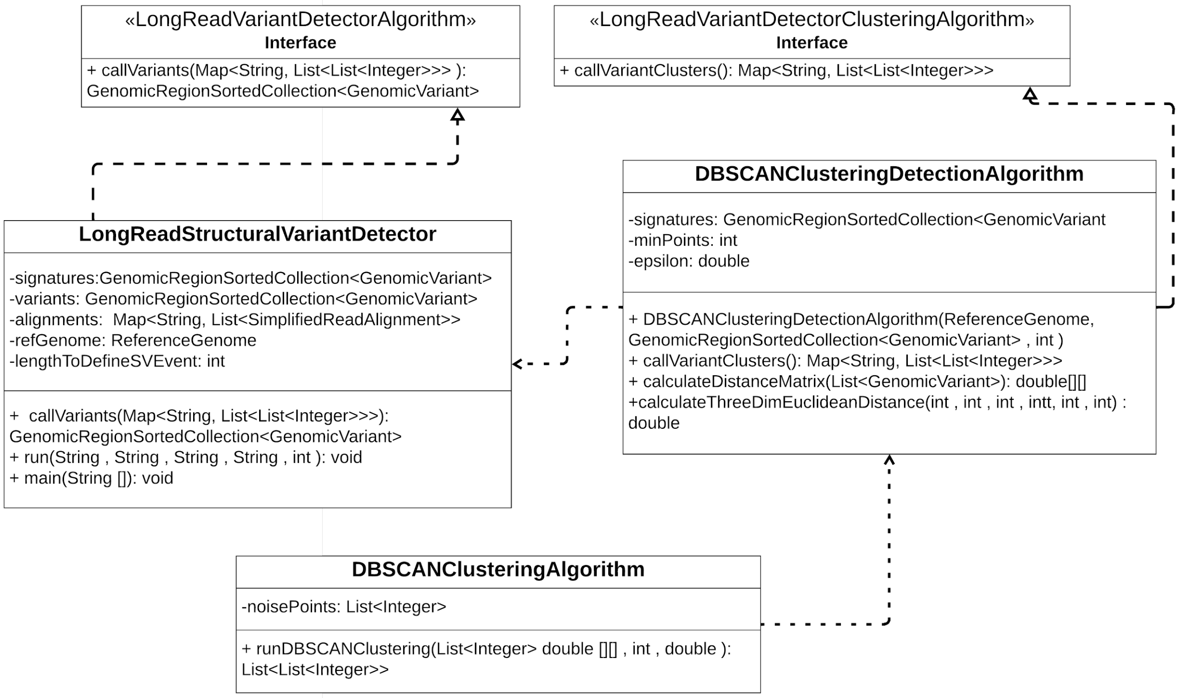

Supplementary figure 12. NGSEP SVCaller functionality UML diagram. Class diagram of the functionality as a UML integrated into NGSEP. The generic algorithm classes are designed so that they can be used for other problems and applications. The clustering detection classes adapt them for the SV clustering functionality dictated by an interface, designed to guide the solution to this problem.

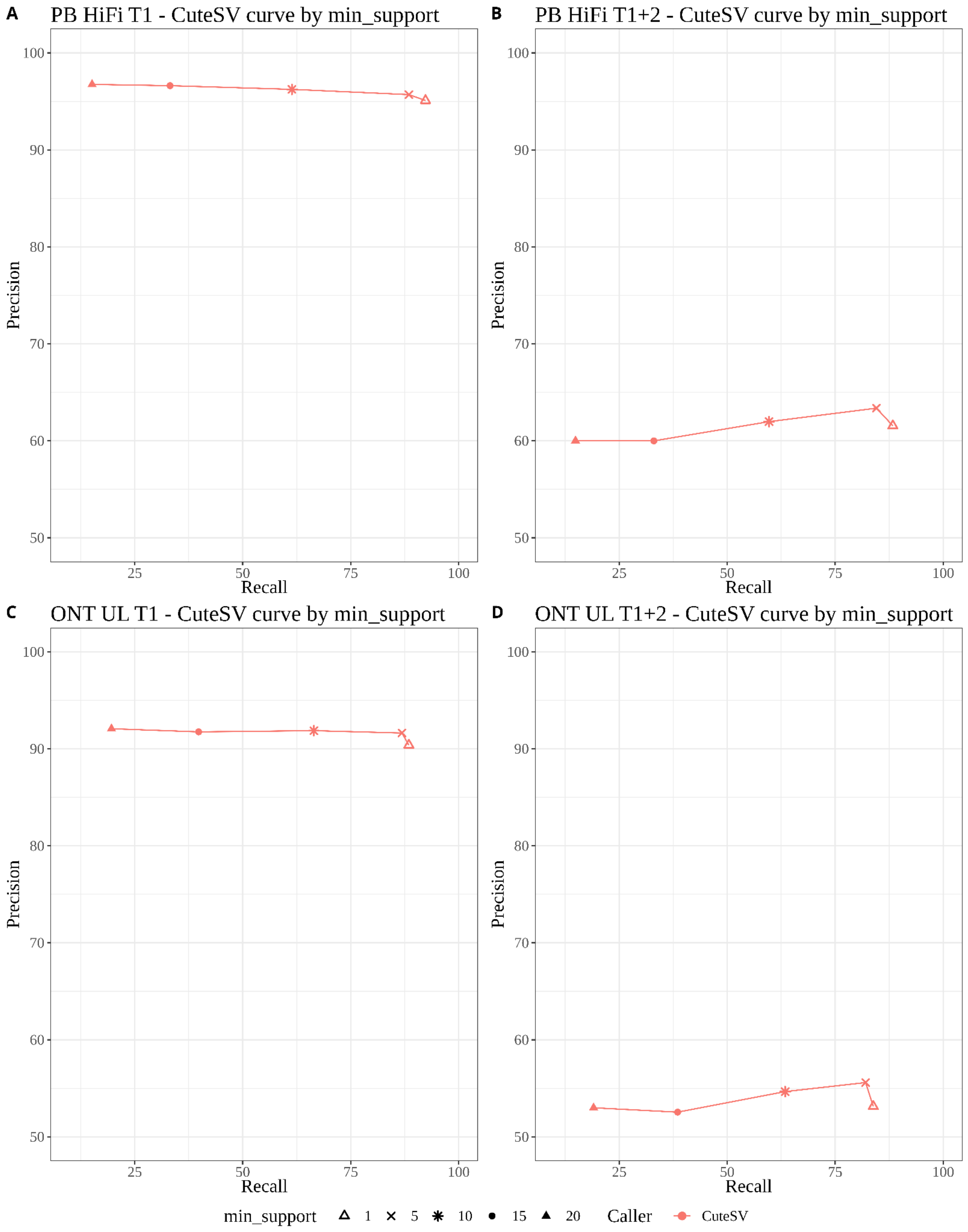

Supplementary figure 13. Experiments changing min read support for CuteSV. The experiments were performed on (A,B) HiFi and (C,D) ONT reads of the benchmark sample HG002, varying the min read support from 1x to 20x. HiFi reads were realigned using pbmm2. Panels A and C. show the results for T1 regions and panels B and D show the results for T1+2 regions.
