# Supplementary material for: A graph clustering algorithm for detection and genotyping of structural variants from long reads
Source: Gigascience. 2024 Jan 11;13:giad112. doi: 10.1093/gigascience/giad112 (PMC10783151; doi:10.1093/gigascience/giad112)
Supplement: giad112_Response_to_Reviewer_Comments_Original_Submission [file giad112_response_to_reviewer_comments_original_submission.pdf]

Author's Response To Reviewer Comments

Please see the complete answer with supporting figures in the personal cover letter file. Here we transcribe the answers to the comments of the reviewers.

Reviewer #1: The submitted study presents an innovative new software for calling de novo structural variants using long-read data. The novelty of the work lies in the application of the DBSCAN clustering algorithm to the problem of identifying SVs from a collection of SV signatures. The paper is well written an easy to follow, and the authors present some good performance metrics for their new software. However, I will highlight some short comings with the manuscript in its current form:

R. We thank the reviewer for the evaluation of our work. We are glad to hear that the main innovation of our work was properly communicated. We performed new benchmark experiments and modified significant parts of the manuscript to properly address the comments of the reviewer. Please find below our answers to each specific comment.

Major points:

The results are currently not reproducible due to missing information, and perhaps missing scripts. In the supplementary, the command used to run NGSEP is not given. I was able to repeat a run on PacBio HiFi data (HG002 sample, 8X coverage) using the following command, and will use the results of this run to raise some further points:

'/usr/bin/time java -jar NGSEPcore_4.3.1.jar SingleSampleVariantsDetector -runOnlySVs -i HG002.pacbio.cram -r ucsc.hg19.fasta -o HG002.ngsep -runLongReadSVs'

The output of this command was a gff file with 17277 SVs which is in line with the Supplementary Data file NGSEP_SVCaller_output_PBHifi_10x.vcf which contained around 20800 SVs using 10X coverage. However, the program only produced a gff file rather than a vcf file, so I was not able to analyze results further. Please can the authors state how to use the software to produce a vcf output and include any further scripts necessary for this.

R. We apologize for this issue with the output format. We also thank the reviewer for taking the time to test the software. On the positive side, the command line tried by the reviewer is accurate, according to the current manual. We included the SVs in the VCF file if SNVs were also called, but unfortunately we were not generating a VCF if SNVs were not called. We fixed this usability issue and, moreover, version 4.3.2 now produces a separate VCF for structural variants identified from long reads. We added to the Supplementary material the command line to run NGSEP and the procedure to replicate the benchmarking using Truvari

The runtime and memory usage of the above command was higher than expected compared to the presented benchmark on the simulated reads (gnu-time command was used to test). The test command appeared to use several threads during execution and finished in 24mins 25s and used 16.7 Gb of memory. The input cram file was only 8.9 Gb in size, so the memory usage may cause issues on some systems. The manuscript would be improved by running the benchmark on the real datasets (PacBio and ONT), and reporting memory usage alongside time.

R. We performed further testing experiments and we believe that both the usage of more than one core and the reports of high memory peaks are more related to the behavior of the Java Virtual machine, which is able to use resources that it sees idle. We have been able to run the functionality on 60x human data using up to 16 Gb of RAM in a laptop having only 4 processors by limiting the available memory using the java option -Xmx. We included in the manuscript the use of memory and time for the GIAB benchmark (see supplementary figure 8). Additionaly, we included the java option in the description of the command line.

The manuscript would be improved by presenting data on the Tier2 HG002 GIAB benchmark dateset, not just Tier 1 regions. Although Tier1 regions are the highest accuracy regions, these also present an 'easy case' for long read callers, as demonstrated by the results - there are relatively small differences among callers in these regions. A key use-case of long-reads is to analyse more difficult genomic regions; therefore, it would be of interest to readers to assess Tier1+2 regions. A quick comparison of NGSEP, dysgu, svim and sniffles indicated large differences in the total number of deletion SVs >=50 bp called and most of these differences probably arise outside of Tier1 regions.

R. We actually had comparisons including tier1+tier2 in an earlier version of the work but we used only tier1 based on a suggestion of a previous reviewer. We finally decided to report both results. The new results are shown in Figure 5 and the new supplementary figures 2 to 6.

The total numbers of SVs called per genome should be presented and discussed. The numbers of called SV that intersect the Tier1+2 and regions should be shown.

R. Following the suggestion, we now refer in the methods section to the supplementary table 1, which contains a thorough description of the number of SV types included in all the datasets used for benchmarking.

Minor points:

The software versions of tools are not given.

R. We improved the results to show the exact version of each tool included in the benchmark experiments, given that it was only specified in the methods section.

Please could the authors also include details of the computer system used for benchmarking.

R. We added a methods section to describe the execution environment used for each experiment.

The software does not report translocations/BND, in contrast to the comparison tools, please could a sentence be included to indicate this.

R. We clarified in the discussion that at this stage the software does not call translocations

The use of a star in the plots to indicate an F1 score is confusing to the reader as it looks like the star is an extra data point rather than an annotation. The stars would be easier to read if they were moved to a legend.

R. We followed the suggestion and moved the F1 scores to the legend

CuteSV appeared to show unexpectedly poor performance at low coverage values, could the authors comment on why this was the case in their experiments.

R. We took a close look at the manuscript to understand the method implemented in CuteSV. The clustering algorithm is relatively simple. They first create clusters based on distance between genomic coordinates, and then they create subclusters based on length differences. The process to define clusters and subclusters is based on simple decision rules tuned by fixed parameters for each event type (which can be changed by user options), such as the distance between signatures, the absolute number of reads needed to support a variant, and the percentage of the average event length within a cluster after which a new subcluster is formed. Given that a large space of parameter options are needed to be tuned for each experiment, we only ran the experiments with default values. In particular, the default value of the "--min_support" parameter (10) is likely to explain the outcome that we obtained. After this analysis, we believe that our method adapts more naturally to different event types and sequencing technologies because we did not have to tune parameters for each particular experiment. We improved the discussion adding a brief summary of this rationale.

SVIM showed very poor precision on nanopore data, could the authors comment on this finding - previous studies suggest SVIM performance suffers on ONT data due to a high number of duplicated true-positive SVs (Cleal et al., 2021).

R. In our experience with SVIM, we observed that they changed their clustering algorithm from a Maximal Clique finder to a hierarchical clustering process. This algorithm produces flat clusters that may fail to resolve close calls. It seems this leads to duplicated SV calls from individual or few nearby signatures, as evidenced by the ratio of duplicate true-positive calls to the number of true-positive calls in Cleal et.al (2022). Additionally, discordant signals may be grouped, and called as a single SV, as the example attached below where only SVIM calls the insertion in the picture from the 10x GIAB benchmark. This is consistent with our results. Thus, we decided to filter their output for comparison as explained in the supplementary table 2 to improve their precision. Further filtering by quality score leads to significant decreases in recall.

The manuscript would be improved by including a lower coverage test on real data, e.g. 5X coverage, as many researchers are interested in utilizing lower coverages.

R. We included the results at 5x in the new simulation of a human individual and in the results with HG002.

Reviewer #2: The authors of the manuscript "A graph clustering algorithm for detection and genotyping of structural variants from long reads" introduce a new method for long read based SV analysis integrated in their NGSEP framework. The main advancement of what they are presenting is the clustering and genotyping methodology, which they show it improves SV detection based on simulated and GIAB benchmark data. The manuscript is overall easy to understand and to follow the points made by the authors. Nevertheless, I feel like I am missing many details on why the algorithm should perform better than existing methodologies that also uses similar concepts on genotyping. In the following I list my questions and concerns in no particular order:

R. We thank the reviewer for the evaluation of our work. We are glad to hear that the text was easy to follow. We performed changes to the methods, results and discussion to address the comments of the reviewer. Please find below our specific answer to each comment.

1. There are overall not many details given that helps me to understand why your algorithm should perform better than the others. This is a bit tricky for me, because I like to understand this to better understand the results that you are showing. For example, you give great detail about why you cluster but then you are not giving much on concrete examples where this clustering matters.

R. We improved the discussion to provide a rationale on why the algorithm could work better than other solutions. Specific reasons for the observed differences against SVIM and CuteSV are described in the answers to the minor comments of reviewer 1. Below we provide examples in which our clustering algorithm provides improved analysis of candidate SV signatures.

2. Their approach of clustering might be novel, but I fail to understand the differences this makes from traditional clustering approaches. Like it is often hard to cluster signals accurately as SV often occur in repeat regions and thus over or under merging can happen. It would be great if you could show some examples of this. It would be interesting to see (since it is one of the main novelties) how it behaves under different circumstances. Like some methods take the SV size into account when clustering the reads to the individual breakpoint. Is that also the case here?

R. The event length is included as one of the dimensions for DBSCAN. Signature false merging is in fact one of the main challenges, especially in repeat regions. We include in the cover letter three examples from the 10x GIAB benchmark in which our algorithm performed a better signal clustering, compared to other approaches.

3. I am also failing to fully understand how an improved clustering can improve the precision of the method, but maybe this comes from genotyping? It would be insightful if the authors give more details on

how they filter their SV candidates.

R. You are right on your assessment that both phases are important for our algorithm to achieve high precision values. In the clustering phase, the DBSCAN algorithm allows for a better differentiation of clusters compared to other algorithms because it is capable of producing different cluster geometries. Even apparently similar points may border two different clusters. In the SV calling context for example, we can precisely call SVs that are adjacent in the genome but vary in length, avoiding false-merging events and a false positive call. In the genotyping phase, due to the power of our Bayesian model, if the posterior hypothesis results in a reference homozygote, we can filter that SV call from the output. We improved the results to describe this process more clearly.

4. It was interesting to me that the authors identify deletions, insertions and inversions, but didn't call duplications or translocations. Is there any reason for this?

R. We improved the results section to better explain that duplications are called after analyzing the variability of starting positions for insertion calls. We decided to not include translocations at this stage because signatures related to these events are more difficult to identify and to cluster, compared to other SV types. Also, we could not find gold standard datasets from real data to assess the reliability of translocation calls.

5. For the simulated data set, how did you evaluate the data set?

R. We improved the methods to explain better the simulation procedures, the number of simulated events and the evaluation procedure. Because the data is simulated, we have a perfect gold standard of structural variants that should be called, and hence we can calculate precision and recall.

6. For the GIAB I saw that you are using custom parameters for Truvari? I see the -p 0.0, which I think refers to the sequence identify comparison. What I am more wondering is the -r 1000. Could you justify this?

R. The -r 1000 parameter allows Truvari to compare SVs that are located at maximum 1000bp between each other, in terms of reference coordinates. This is specially important for bigger SVs, given that breakpoints are not consistently detected between callers. Using the default -r 500 could lead to underestimating the recall of some tools that may miss on the coordinates of the variant but are detecting it nonetheless.

7. I would have liked to test this SV caller myself but it seems that this is not so easily possible since I have to install the entire software analysis suit.. or is there a way around this?

R. To run NGSEP from the command line you just need to have the general purpose java virtual machine (v11 or superior) installed in your computer. You can either install the versions from Oracle or the openJDK in the case of linux. After that, you just need the jar file of NGSEP (currently version 4.3.2), which you can download from our website (https://sourceforge.net/projects/ngsep/files/Library/). Once downloaded, if you go to the download folder in the command line and type:

java -jar NGSEPcore_4.3.2.jar

You will see the different functionalities offered by the software. In particular, the functionality described in this manuscript can be executed typing:

java -jar NGSEPcore_4.3.2.jar SingleSampleVariantsDetector

Finally, the command we used for our experiments has been included in the Supplementary table 2.

8. I am missing also some details on the simulation. How many SV were simulated per data set, whats the sizes of SV simulated etc. I see some detail in the methods, but it only says indel were simulated and not inversion?

R. We improved the methods to better explain the simulation procedures, the number of simulated events and the evaluation procedure. We included the number of inversions in the text. You can find the exact breakdown by type of number of SVs for each gold standard dataset in the Supplementary Table 1.

Reviewer #3: The paper is well written and the results are good.
The methods described here remind me of those from Jasmine and Iris (Kirsche 2023), and I would like to authors to help me understand the differences between the two clustering approaches.

R. Thanks for your assessment of the manuscript. We went over the work of Kirsche et al., 2023 and we found that Jasmine solves a relatively different problem, namely the merging of individual SV calls. We are glad to see that they follow an alternative similar to that implemented in NGSEP. Going over the details of the algorithm, the Jasmine algorithm represents SVs as points in a two dimensional Euclidean space consisting of the first reference coordinate and length of the variants. Then, they produce a graph with the SVs as vertices by adding distance weighted-edges between them, complying with different restrictions. In our approach, we also include the end coordinate of each event and hence our graph represents signatures into a 3D Euclidean space, restricting the complete graph based on a distance threshold. The major divergence comes when Jasmine produces a Minimal Spanning Forest, to merge SVs into a single call if they are found in the same tree. In contrast, we use the DBSCAN algorithm to cluster the signatures into an individual SV call, processing the graph through a Breadth First Search. We included the paper in the references and made an appropriate citation in the discussion.

Also, it would be helpful to break up the results by SV type to see if the improvements seen are general or specific.

R. We improved the results to show clearly the behavior of the method separately for insertions and deletions in the human GIAB benchmark experiments (Supplementary figures 3-6). We only could assess inversions in the simulations with the Arabidopsis genome because the human gold standard datasets do not include inversions. The results for inversions are separated from those of insertions and deletions in figure 4.

Reviewer #4: In this manuscript named "A graph clustering algorithm for detection and genotyping of structural variants from long reads", Gaitán et al represented an algorithm that detect genomic structural variants (SV) from long reads. Albeit the existence of multiple long-read based SV discovery methods, I can see the value of this method to the field of genomic SV and long-read sequencing. However, the benchmarking data and methods described in this manuscript are outdated, and significant revision should be applied before this manuscript can qualify for publication. Below are my specific comments:

R. We thank the reviewer for the assessment of our work. We are glad to hear that the work is perceived as valuable by the reviewer. We performed further benchmark experiments and improved the results and discussion to address each comment of the reviewer. Please find below our specific answer to each comment.

1. Benchmarking methods: the authors included SVIM, Sniffles, CuteSV and Dysgu as benchmarking methods. However, there are newer and better algorithms available, including Sniffles2 (which has significant improvements over Sniffles) and PBSV. In addition, assembly based long-read SV methods, such as PAV, could also be considered for benchmarking.

R. We mentioned the exact version numbers for the different tools in the results section. Although we cited the manuscript of Sniffles1, the results shown in the initial review were generated with the 2.0.6 version of Sniffles. In any case, we double checked that the benchmark was performed using the latest version of each tool. Regarding PBSV, we did not include it in the initial benchmark because we could not find a publication related to this tool. We tried to include this tool directly, but unfortunately it could not run with the minimap alignment files. Thus, we had to realign the reads of the original alignment files with the pbmm2 minimap2 wrapper. Since this would represent a different dataset for benchmarking, we ran all tools on these alignments. Please see the supplementary figure 7 for details. Overall, PBSV ranked below CuteSV and NGSEP,on precision, recall and GT accuracy.

Regarding comparisons with a de-novo assembly and contig mapping approach, we believe that results of de-novo assembly comparisons are generally better than those of read alignment based SV callers. However, they are more expensive given that larger depths are required to achieve a high quality de-novo genome assembly, in comparison to low-depth long-read sampling. Additionally, the two gold-standard datasets we used for real data benchmarking include calls from assembly based methods (especially HGSVC). In the second version, this consortium improved their callset by using the PAV algorithm. Hence, we believe that benchmarking against de-novo assembly methods will not make a fair comparison in these cases.

2. Simulation data benchmarking: it's useful to learn the performance of simulation data in the genome of Arabidopsis thaliana, but most important to simulate the human genome for benchmarking.

R. We did not do this before because we had the HG002 data. We performed the suggested simulation based on the T2T genome, and described the results (Supplementary figure 1).

3. Real data benchmarking: Gaitán et al compared the performance of NGSEP using HG002 base on the reference of GRCh37.Hhowever, in most of the current studies, both short-read and long-read data, are aligned against GRCh38 or T2T, and these data are of significantly more interest to the filed. Please either re-align the GIAB data against the newer reference genomes for the benchmarking or use other data such as those generated by the human genome structural variation consortium (HGSVC, Check Chaisson et al. 2019. and Ebert et al. 2021)

R. Although we agree that the GRCh38 genome, or even the T2T genome would be more interesting to perform these benchmark experiments, the public gold standard of GIAB to perform independent benchmark experiments is currently available only for GRCh37. Nevertheless, following this comment we found the dataset of the HGSVC consortium and we performed further benchmark experiments using this dataset, achieving very good performance. Please see the results in figure 6 and supplementary figures 9-11. We thank the reviewer for leading us to this asset, and allowing us to further improve our benchmark experiments.
