# Supplementary material for: A graph clustering algorithm for detection and genotyping of structural variants from long reads
Source: Gigascience. 2024 Jan 11;13:giad112. doi: 10.1093/gigascience/giad112 (PMC10783151; doi:10.1093/gigascience/giad112)
Supplement: giad112_Response_to_Reviewer_Comments_Revision_1 [file giad112_response_to_reviewer_comments_revision_1.pdf]

Author's Response To Reviewer Comments

6-Oct-2023
Dear editor Dr. Hans Zauner
Many thanks for your assessment of our manuscript 'A graph clustering algorithm for detection and genotyping of structural variants from long reads' and for giving us the opportunity to submit a revised version of the manuscript. We carefully read the follow up comments of the reviewer. We updated the additional benchmark experiments and made changes in the manuscript to address each comment. Please find our answers below for each specific comment. To facilitate the revision process, we marked in red the changes performed from the previous version of the manuscript.
This revised version was seen and approved by all co-authors of this manuscript. We appreciate your consideration of this version and we look forward to your assessment.

Sincerely

Jorge Duitama Ph.D
Associate professor
Systems and Computing Engineering Department
Universidad de los Andes
Bogotá, Colombia
Tel: (+57) (1) 3394949 Ext 1686.
E-mail: ja.duitama@uniandes.edu.co

GIGA-D-23-00070R1
A graph clustering algorithm for detection and genotyping of structural variants from long reads
Nicolás Gaitán; Jorge Duitama
GigaScience

Dear Dr. Duitama,

Your revised manuscript "A graph clustering algorithm for detection and genotyping of structural variants from long reads" (GIGA-D-23-00070R1) has been assessed again by one of the reviewers. Based on the latest report, I feel the manuscript can be potentially acceptable for publication in GigaScience, once you have carried out some important follow-up revisions suggested by the reviewer.

The latest report is below.

Once you have made the necessary corrections, please submit a revised manuscript online at:

If you have forgotten your username or password please use the "Send Login Details" link to get your login information. For security reasons, your password will be reset.

Please include a point-by-point within the 'Response to Reviewers' box in the submission system. Please ensure you describe additional experiments that were carried out and include a detailed rebuttal of any criticisms or requested revisions that you disagreed with. Please also ensure that your revised manuscript conforms to the journal style, which can be found in the Instructions for Authors on the journal homepage. If the data and code has been modified in the revision process please be sure to update the public versions of this too.

The due date for submitting the revised version of your article is 20 Dec 2023.

We look forward to receiving your revised manuscript soon.

Best wishes,

Hans Zauner
GigaScience

Reviewer reports:
Reviewer #1: I wish to thank the authors for the detailed response, including more data, and for addressing most of the points I raised in the initial review. I have a few outstanding issues with the manuscript, which should not be too difficult to address.

1. I apologize for not finding the version numbers in the original submission. However, the versions of most of the tools are out of date by roughly a year. CuteSV, dysgu and sniffles have received substantial updates. The manuscript would be improved if these were updated to recent versions.

R. We thank the reviewer for the follow up revision and comments. We updated the benchmark experiments using the newest versions of each tool at this moment (Dysgu v1.6.1, CuteSV v2.0.3, Sniffles v2.2, and SVIM v2.0.0) and adjusted the manuscript according to the new results. We made different observations when comparing the new versions against the older ones. The improvements of CuteSV in our updated experiments are mostly caused by the change in the value of the min_support parameter, rather than the version update. We follow this discussion in-depth in the response to the second comment. Regarding Dysgu, we found that the results on the GIAB dataset improved significantly in terms of precision for PacBio data, but this was not the case for the Tier 1+2 benchmark from the Nanopore data, even with the recommended parameters. In particular, for the GIAB pbmm2 remappings, we encountered segmentation fault errors that made it impossible to use v1.6.1, thus, we downgraded to Dysgu v1.5 for that specific dataset. Since Dysgu is in active development and this outcome is probably produced by a bug that could be easily fixed, we decided not to report in the paper this bug and the version downgrade that we had to do. Additionally, the recall of Dysgu on the HGSVC2 samples decreased significantly compared to v1.3.11, while the improvement of precision was not enough to compensate, generating a reduction in F1-score overall. The results of Sniffles did not change significantly, and the issue of reported doubled lengths for SVs discovered from the HGSVC2 samples remains with the updated version. Therefore, we applied the same previous solution to include a benchmark with a smaller reciprocal overlap parameter for Truvari (Supplementary Figures 9,11). Nevertheless, updating software versions allowed us to improve our benchmarking experiments and provided insights into the best practices for using each caller.

2. Going back to the point that cuteSV shows low sensitivity. As now pointed out by the authors, this is due to the default parameter for minimum read support, which is normally set at 10 for the tool. This probably explains the poor performance at low coverages and the excellent performance at high coverages. However, testing cuteSV with default settings on low coverage samples (5x, 10x) is not a fair test of the tool. Can I suggest making a precision-recall curve at one or two fixed coverage values but vary the minimum-support of the tools. This would be an interesting supplementary figure and would show how optimizing the minimum support parameter could affect performance.

R. We followed this suggestion and updated the experiments using the latest version of CuteSV, and reducing the minimum read support to 5x. However, we ran into an issue running the latest version of CuteSV (v2.0.3) on the original alignments downloaded from the GIAB repository. The tool produced low recall for all depths. This issue was fixed running this version with the pbmm2 realigned datasets. This unexpected behavior did not happen with version 1.0.13 of CuteSV.

Going back to the min support parameter, we also performed experiments varying the this parameter (1,5,10,15,20) on the PacBio 20x HG002 reads, realigned with pbmm2, and with the ONT reads for HG002 (See supplementary figure 13). We observed that lowering the min_support value to 5 produced the best performance metrics. In comparison to the results with the parameter set to 5, a value of 1 creates more false positives in exchange for better sensitivity, while bigger values significantly decrease recall with no precision improvement. Hence, we applied a min_support=5 to all runs of CuteSV in all the benchmark experiments, improving the performance of this software overall. In particular, this change produced a significant improvement in the performance of CuteSV on the HGSVC2 benchmark.

3. The performance of NGSEP seems to remain high at different coverages. Could the authors explain why this might be the case, for example are some dynamic thresholds used, or is this all down to the genotyping?

R. Your assessment is correct regarding the genotyping phase as the main cause for the consistent performance of NGSEP at different coverages, particularly regarding precision. Our Bayesian genotyping algorithm accurately predicts most false positive cases where inconsistent evidence supports an SV call according to the total amount of spanning read alignments, classifying the call as a Homozygous reference and filtering it out from the vcf output. In particular, when a low-depth sample is analyzed, each supporting read will have more weight in the probability of an alternate call, while keeping discordant or unique signals from being reported. Hence, we avoided the need to implement any dynamic thresholds or even a minimal read support parameter.

4. I would like to try and replicate the results in the paper, but the authors appear to be merging several different samples before down-sampling to the desired coverage value. I don't have an issue with this approach, but at the same time it makes repeating the results problematic. It would be useful for others in the field if a supplementary section was included, giving a series of commands to repeat some of these results, even if only for a single sample.

R. We added the supplementary file 2 with a thorough guide, including download links to each dataset, to ensure the reproducibility of our benchmarking procedure.
