# Supplementary material for: A graph clustering algorithm for detection and genotyping of structural variants from long reads
Source: Gigascience. 2024 Jan 11;13:giad112. doi: 10.1093/gigascience/giad112 (PMC10783151; doi:10.1093/gigascience/giad112)
Supplement: giad112_Response_to_Reviewer_Comments_Revision_2 [file giad112_response_to_reviewer_comments_revision_2.pdf]

# Author's Response To Reviewer Comments

Dear editor Hans Zauner

Many thanks for your assistance through this process. We performed the requested changes to make the manuscript acceptable for publication in GigaScience. This are our answers to each specific request:

1) Please add ORCIDs for all authors to the title page (we have these on our file: ORCID iDs: Nicolás Gaitán [0009-0009-2979-2703]; Jorge Duitama [0000-0002-9105-6266];)

R. We added the ORCIDs before the abstract

2) Please structure your abstract ("Background" - "Findings" - "Conclusions".)

R. We separated the abstract in three sections as requested

3) Bibliography: please use numbered references, not by alphabetical order. Refer to our instructions for "Technical Notes" for details on the article format.

R. We numbered the references and followed the corresponding guidelines for technical notes.

4) Add your new GigaDb dataset as a numbered reference to the bibliography, including the doi link. The citation is:

[xx] Gaitán N, Duitama J Supporting data for "A graph clustering algorithm for detection and genotyping of structural variants from long reads" GigaScience Database. 2023. http://dx.doi.org/10.5524/102475

Please cite the GigaDB set by number from the data availability section. For example, write something like: "An archival copy of the code and supporting data is also available via the GigaScience database GigaDB [xx]".

R. We added the reference and the citation at the end of the data availability section as requested.

5) Please check the bibliograpy if any preprints have meanwhile be published - if so, please cite the journal citation instead of the preprint (e.g. the Truvari paper is now published in Genome Biology, if I'm not mistaken).

R. We modified the reference to Truvari. Only the reference to the preprint of the Sniffles2 paper remains because we could not find evidence of publication of this paper in any journal.

6) Move all URLs / dois for data sources, home pages etc. from the main text to the bibliography, and cite them by reference number - analogous to the GigaDB example above. (We treat databases, software repos and other internet sources as citable items, which should have their own numbered reference in the bibliography. Please refer to our instructions for authors for details on the format).

R. We moved all URLs to the references section and cited them in the manuscript as specified in the instructions for technical notes

Best regards

Jorge Duitama Ph.D.
Associate professor
Systems and Computing Engineering Department
Universidad de los Andes
Bogotá, Colombia
