# Supplementary material for: A graph clustering algorithm for detection and genotyping of structural variants from long reads
Source: Gigascience. 2024 Jan 11;13:giad112. doi: 10.1093/gigascience/giad112 (PMC10783151; doi:10.1093/gigascience/giad112)
Supplement: giad112_Reviewer_1_Report_Original_Submission — Kez Cleal -- 4/17/2023 Reviewed [file giad112_reviewer_1_report_original_submission.pdf]

Reviewer Report

Title: A graph clustering algorithm for detection and genotyping of structural variants from long reads

Version: Original Submission **Date:** 4/17/2023

Reviewer name: Kez Cleal

Reviewer Comments to Author:

The submitted study presents an innovative new software for calling de novo structural variants using long-read data. The novelty of the work lies in the application of the DBSCAN clustering algorithm to the problem of identifying SVs from a collection of SV signatures. The paper is well written an easy to follow, and the authors present some good performance metrics for their new software. However, I will highlight some short comings with the manuscript in its current form:Major points:The results are currently not reproducible due to missing information, and perhaps missing scripts. In the supplementary, the command used to run NGSEP is not given. I was able to repeat a run on PacBio HiFi data (HG002 sample, 8X coverage) using the following command, and will use the results of this run to raise some further points:'/usr/bin/time java -jar NGSEPcore_4.3.1.jar SingleSampleVariantsDetector -runOnlySVs -i HG002.pacbio.cram -r ucsc.hg19.fasta -o HG002.ngsep -runLongReadSVs'The output of this command was a gff file with 17277 SVs which is in line with the Supplementary Data file NGSEP_SVCaller_output_PBHifi_10x.vcf which contained around 20800 SVs using 10X coverage. However, the program only produced a gff file rather than a vcf file, so I was not able to analyze results further. Please can the authors state how to use the software to produce a vcf output and include any further scripts necessary for this.The runtime and memory usage of the above command was higher than expected compared to the presented benchmark on the simulated reads (gnu-time command was used to test). The test command appeared to use several threads during execution and finished in 24mins 25s and used 16.7 Gb of memory. The input cram file was only 8.9 Gb in size, so the memory usage may cause issues on some systems. The manuscript would be improved by running the benchmark on the real datasets (PacBio and ONT), and reporting memory usage alongside time.The manuscript would be improved by presenting data on the Tier2 HG002 GIAB benchmark dateset, not just Tier 1 regions. Although Tier1 regions are the highest accuracy regions, these also present an 'easy case' for long read callers, as demonstrated by the results - there are relatively small differences among callers in these regions. A key use-case of long-reads is to analyse more difficult genomic regions; therefore, it would be of interest to readers to assess Tier1+2 regions. A quick comparison of NGSEP, dysgu, svim and sniffles indicated large differences in the total number of deletion SVs >=50 bp called and most of these differences probably arise outside of Tier1 regions.The total numbers of SVs called per genome should be presented and discussed. The numbers of called SV that intersect the Tier1+2 and regions should be shown.Minor points:The software versions of tools are not given.Please could the authors also include details of the computer system used for benchmarking.The software does not report translocations/BND, in contrast to the comparison tools, please could a sentence be included to indicate this.The use of a star in the plots to indicate an F1 score is confusing to the reader as it looks like the star is an extra data point rather than an annotation. The stars would be easier to read if they

were moved to a legend.CuteSV appeared to show unexpectedly poor performance at low coverage values, could the authors comment on why this was the case in their experiments.SVIM showed very poor precision on nanopore data, could the authors comment on this finding - previous studies suggest SVIM performance suffers on ONT data due to a high number of duplicated true-positive SVs (Cleal et al., 2021).The manuscript would be improved by including a lower coverage test on real data, e.g. 5X coverage, as many researchers are interested in utilizing lower coverages.

Level of Interest

Please indicate how interesting you found the manuscript: Choose an item.

Quality of Written English

Please indicate the quality of language in the manuscript: Choose an item.

Declaration of Competing Interests

Please complete a declaration of competing interests, considering the following questions:

- Have you in the past five years received reimbursements, fees, funding, or salary from an organisation that may in any way gain or lose financially from the publication of this manuscript, either now or in the future?
- Do you hold any stocks or shares in an organisation that may in any way gain or lose financially from the publication of this manuscript, either now or in the future?
- Do you hold or are you currently applying for any patents relating to the content of the manuscript?
- Have you received reimbursements, fees, funding, or salary from an organization that holds or has applied for patents relating to the content of the manuscript?
- Do you have any other financial competing interests?
- Do you have any non-financial competing interests in relation to this paper?

If you can answer no to all of the above, write 'I declare that I have no competing interests' below. If your reply is yes to any, please give details below.

I declare that I have no competing interests

I agree to the open peer review policy of the journal. I understand that my name will be included on my report to the authors and, if the manuscript is accepted for publication, my named report including any attachments I upload will be posted on the website along with the authors' responses. I agree for my report to be made available under an Open Access Creative Commons CC-BY license (http://creativecommons.org/licenses/by/4.0/). I understand that any comments which I do not wish to

be included in my named report can be included as confidential comments to the editors, which will not be published.

Choose an item.

To further support our reviewers, we have joined with Publons, where you can gain additional credit to further highlight your hard work (see: https://publons.com/journal/530/gigascience). On publication of this paper, your review will be automatically added to Publons, you can then choose whether or not to claim your Publons credit. I understand this statement.

Yes Choose an item.
