# Supplementary material for: A graph clustering algorithm for detection and genotyping of structural variants from long reads
Source: Gigascience. 2024 Jan 11;13:giad112. doi: 10.1093/gigascience/giad112 (PMC10783151; doi:10.1093/gigascience/giad112)
Supplement: giad112_Reviewer_1_Report_Revision_1 — J Kez Cleal -- 8/14/2023 Reviewed [file giad112_reviewer_1_report_revision_1.pdf]

Reviewer Report

Title: A graph clustering algorithm for detection and genotyping of structural variants from long reads

Version: Revision 1 **Date:** 8/14/2023

Reviewer name: Kez Cleal

Reviewer Comments to Author:

I wish to thank the authors for the detailed response, including more data, and for addressing most of the points I raised in the initial review. I have a few outstanding issues with the manuscript, which should not be too difficult to address.1. I apologize for not finding the version numbers in the original submission. However, the versions of most of the tools are out of date by roughly a year. CuteSV, dysgu and sniffles have received substantial updates. The manuscript would be improved if these were updated to recent versions.2. Going back to the point that cuteSV shows low sensitivity. As now pointed out by the authors, this is due to the default parameter for minimum read support, which is normally set at 10 for the tool. This probably explains the poor performance at low coverages and the excellent performance at high coverages. However, testing cuteSV with default settings on low coverage samples (5x, 10x) is not a fair test of the tool. Can I suggest making a precision-recall curve at one or two fixed coverage values but vary the minimum-support of the tools. This would be an interesting supplementary figure and would show how optimizing the minimum support parameter could affect performance.3. The performance of NGSEP seems to remain high at different coverages. Could the authors explain why this might be the case, for example are some dynamic thresholds used, or is this all down to the genotyping?4. I would like to try and replicate the results in the paper, but the authors appear to be merging several different samples before down-sampling to the desired coverage value. I don't have an issue with this approach, but at the same time it makes repeating the results problematic. It would be useful for others in the field if a supplementary section was included, giving a series of commands to repeat some of these results, even if only for a single sample.

Level of Interest

Please indicate how interesting you found the manuscript: Choose an item.

Quality of Written English

Please indicate the quality of language in the manuscript: Choose an item.

Declaration of Competing Interests

Please complete a declaration of competing interests, considering the following questions:

- Have you in the past five years received reimbursements, fees, funding, or salary from an organisation that may in any way gain or lose financially from the publication of this manuscript, either now or in the future?
- Do you hold any stocks or shares in an organisation that may in any way gain or lose financially from the publication of this manuscript, either now or in the future?
- Do you hold or are you currently applying for any patents relating to the content of the manuscript?
- Have you received reimbursements, fees, funding, or salary from an organization that holds or has applied for patents relating to the content of the manuscript?
- Do you have any other financial competing interests?
- Do you have any non-financial competing interests in relation to this paper?

If you can answer no to all of the above, write 'I declare that I have no competing interests' below. If your reply is yes to any, please give details below.

I declare that I have no competing interests

I agree to the open peer review policy of the journal. I understand that my name will be included on my report to the authors and, if the manuscript is accepted for publication, my named report including any attachments I upload will be posted on the website along with the authors' responses. I agree for my report to be made available under an Open Access Creative Commons CC-BY license (http://creativecommons.org/licenses/by/4.0/). I understand that any comments which I do not wish to be included in my named report can be included as confidential comments to the editors, which will not be published.

Choose an item.

To further support our reviewers, we have joined with Publons, where you can gain additional credit to further highlight your hard work (see: https://publons.com/journal/530/gigascience). On publication of this paper, your review will be automatically added to Publons, you can then choose whether or not to claim your Publons credit. I understand this statement.

Yes Choose an item.
