# Supplementary material for: A graph clustering algorithm for detection and genotyping of structural variants from long reads
Source: Gigascience. 2024 Jan 11;13:giad112. doi: 10.1093/gigascience/giad112 (PMC10783151; doi:10.1093/gigascience/giad112)
Supplement: giad112_Reviewer_2_Report_Original_Submission — Fritz J Sedlazeck -- 5/2/2023 Reviewed [file giad112_reviewer_2_report_original_submission.pdf]

Reviewer Report

Title: A graph clustering algorithm for detection and genotyping of structural variants from long reads

Version: Original Submission **Date:** 5/2/2023

Reviewer name: Fritz J Sedlazeck

Reviewer Comments to Author:

The authors of the manuscript "A graph clustering algorithm for detection and genotyping of structural variants from long reads" introduce a new method for long read based SV analysis integrated in their NGSEP framework. The main advancement of what they are presenting is the clustering and genotyping methodology, which they show it improves SV detection based on simulated and GIAB benchmark data. The manuscript is overall easy to understand and to follow the points made by the authors. Nevertheless, I feel like I am missing many details on why the algorithm should perform better than existing methodologies that also uses similar concepts on genotyping. In the following I list my questions and concerns in no particular order:1. There are overall not many details given that helps me to understand why your algorithm should perform better than the others. This is a bit tricky for me, because I like to understand this to better understand the results that you are showing. For example, you give great detail about why you cluster but then you are not giving much on concrete examples where this clustering matters.2. Their approach of clustering might be novel, but I fail to understand the differences this makes from traditional clustering approaches. Like it is often hard to cluster signals accurately as SV often occur in repeat regions and thus over or under merging can happen. It would be great if you could show some examples of this. It would be interesting to see (since it is one of the main novelties) how it behaves under different circumstances. Like some methods take the SV size into account when clustering the reads to the individual breakpoint. Is that also the case here?3. I am also failing to fully understand how an improved clustering can improve the precision of the method, but maybe this comes from genotyping? It would be insightful if the authors give more details on how they filter their SV candidates.4. It was interesting to me that the authors identify deletions, insertions and inversions, but didn't call duplications or translocations. Is there any reason for this?5. For the simulated data set, how did you evaluate the data set?6. For the GIAB I saw that you are using custom parameters for Truvari? I see the -p 0.0, which I think refers to the sequence identify comparison. What I am more wondering is the -r 1000. Could you justify this?7. I would have liked to test this SV caller myself but it seems that this is not so easily possible since I have to install the entire software analysis suit.. or is there a way around this?8. I am missing also some details on the simulation. How many SV were simulated per data set, whats the sizes of SV simulated etc. I see some detail in the methods, but it only says indel were simulated and not inversion?

Level of Interest

Please indicate how interesting you found the manuscript: Choose an item.

Quality of Written English

Please indicate the quality of language in the manuscript: Choose an item.

Declaration of Competing Interests

Please complete a declaration of competing interests, considering the following questions:

- Have you in the past five years received reimbursements, fees, funding, or salary from an organisation that may in any way gain or lose financially from the publication of this manuscript, either now or in the future?
- Do you hold any stocks or shares in an organisation that may in any way gain or lose financially from the publication of this manuscript, either now or in the future?
- Do you hold or are you currently applying for any patents relating to the content of the manuscript?
- Have you received reimbursements, fees, funding, or salary from an organization that holds or has applied for patents relating to the content of the manuscript?
- Do you have any other financial competing interests?
- Do you have any non-financial competing interests in relation to this paper?

If you can answer no to all of the above, write 'I declare that I have no competing interests' below. If your reply is yes to any, please give details below.

I am getting some research support from Pacbio and ONT.

I agree to the open peer review policy of the journal. I understand that my name will be included on my report to the authors and, if the manuscript is accepted for publication, my named report including any attachments I upload will be posted on the website along with the authors' responses. I agree for my report to be made available under an Open Access Creative Commons CC-BY license (http://creativecommons.org/licenses/by/4.0/). I understand that any comments which I do not wish to be included in my named report can be included as confidential comments to the editors, which will not be published.

Choose an item.

To further support our reviewers, we have joined with Publons, where you can gain additional credit to further highlight your hard work (see: https://publons.com/journal/530/gigascience). On publication of this paper, your review will be automatically added to Publons, you can then choose whether or not to claim your Publons credit. I understand this statement.

Yes Choose an item.
