# Supplementary material for: A graph clustering algorithm for detection and genotyping of structural variants from long reads
Source: Gigascience. 2024 Jan 11;13:giad112. doi: 10.1093/gigascience/giad112 (PMC10783151; doi:10.1093/gigascience/giad112)
Supplement: giad112_Reviewer_4_Report_Original_Submission — Xuefang Zhao -- 5/9/2023 Reviewed [file giad112_reviewer_4_report_original_submission.pdf]

Reviewer Report

Title: A graph clustering algorithm for detection and genotyping of structural variants from long reads

Version: Original Submission **Date:** 5/9/2023

Reviewer name: Xuefang Zhao

Reviewer Comments to Author:

In this manuscript named "A graph clustering algorithm for detection and genotyping of structural variants from long reads", GaitÃ¡n et al represented an algorithm that detect genomic structural variants (SV) from long reads. Albeit the existence of multiple long-read based SV discovery methods, I can see the value of this method to the field of genomic SV and long-read sequencing. However, the benchmarking data and methods described in this manuscript are outdated, and significant revision should be applied before this manuscript can qualify for publication. Below are my specific comments:1. Benchmarking methods: the authors included SVIM, Sniffles, CuteSV and Dysgu as benchmarking methods. However, there are newer and better algorithms available, including Sniffles2 (which has significant improvements over Sniffles) and PBSV. In addition, assembly based long-read SV methods, such as PAV, could also be considered for benchmarking.2. Simulation data benchmarking: it's useful to learn the performance of simulation data in the genome of Arabidopsis thaliana, but most important to simulate the human genome for benchmarking.3. Real data benchmarking: GaitÃ¡n et al compared the performance of NGSEP using HG002 base on the reference of GRCh37.Hhowever, in most of the current studies, both short-read and long-read data, are aligned against GRCh38 or T2T, and these data are of significantly more interest to the filed. Please either re-align the GIAB data against the newer reference genomes for the benchmarking or use other data such as those generated by the human genome structural variation consortium (HGSVC, Check Chaisson et al. 2019. and Ebert et al. 2021)

Level of Interest

Please indicate how interesting you found the manuscript: Choose an item.

Quality of Written English

Please indicate the quality of language in the manuscript: Choose an item.

Declaration of Competing Interests

Please complete a declaration of competing interests, considering the following questions:

- Have you in the past five years received reimbursements, fees, funding, or salary from an organisation that may in any way gain or lose financially from the publication of this manuscript, either now or in the future?

- Do you hold any stocks or shares in an organisation that may in any way gain or lose financially from the publication of this manuscript, either now or in the future?
- Do you hold or are you currently applying for any patents relating to the content of the manuscript?
- Have you received reimbursements, fees, funding, or salary from an organization that holds or has applied for patents relating to the content of the manuscript?
- Do you have any other financial competing interests?
- Do you have any non-financial competing interests in relation to this paper?

If you can answer no to all of the above, write 'I declare that I have no competing interests' below. If your reply is yes to any, please give details below.

I declare that I have no competing interests

I agree to the open peer review policy of the journal. I understand that my name will be included on my report to the authors and, if the manuscript is accepted for publication, my named report including any attachments I upload will be posted on the website along with the authors' responses. I agree for my report to be made available under an Open Access Creative Commons CC-BY license (http://creativecommons.org/licenses/by/4.0/). I understand that any comments which I do not wish to be included in my named report can be included as confidential comments to the editors, which will not be published.

Choose an item.

To further support our reviewers, we have joined with Publons, where you can gain additional credit to further highlight your hard work (see: https://publons.com/journal/530/gigascience). On publication of this paper, your review will be automatically added to Publons, you can then choose whether or not to claim your Publons credit. I understand this statement.

Yes Choose an item.
